# Phosphasilene mediated CO activation and deoxygenative homo coupling of CO molecules in reactions with metal carbonyls†

**DOI:** 10.1039/d4sc05491a

**Published:** 2024-10-10

**Authors:** Zohreh Hendi, Renuka Pradhan, Katharina Rachuy, Soheil Mahmoudi, Madhusudan K. Pandey, Saroj Kumar Kushvaha, Regine Herbst-Irmer, Upakarasamy Lourderaj, Dietmar Stalke, Herbert W. Roesky

**Affiliations:** a Institut für Anorganische Chemie, Georg-August-Universität Göttingen Göttingen 37077 Germany hroesky@gwdg.de dstalke@chemie.uni-goettingen.de; b School of Chemical Sciences, National Institute of Science Education and Research (NISER) Bhubaneswar Bhubaneswar India u.lourderaj@niser.ac.in; c University of Vienna, Faculty of Chemistry, Institute of Inorganic Chemistry Waehringer Str. 42 Vienna 1090 Austria; d University of Vienna, Vienna Doctoral School in Chemistry (DoSChem), Waehringer Str. 42 1090 Vienna Austria

## Abstract

Herein, we report the synthesis of a new sterically demanding hyper-coordinate phosphasilene (Mes*PSi(SiMe_3_)(PhC(N^*t*^Bu)_2_) (1) and its unprecedented reactivity with metal carbonyls (M = Fe, Mo, W). The reaction of 1 with Fe(CO)_5_ involves the deoxygenative homocoupling of two CO molecules, forming a rare ketene (μ-CCO) inserted Fe complex 2. Contrastingly, reactions with M(CO)_6_ (M = Mo, W) entail the deoxygenated activation of one CO molecule, with the second CO molecule being trapped between Si and P atoms. All the compounds including their crystal structures, are thoroughly characterized and potential energy profiles for the reaction mechanisms are also explored.

## Abstract 2

This paper describes the unique reactivity of phosphasilene (1) with metal carbonyls (M = Fe, Mo and W) resulting in the deoxygenative homo-coupling of two CO molecules and CO activation upon treatment with Fe (2) and W/Mo (3/4), respectively.

## Graphical abstract

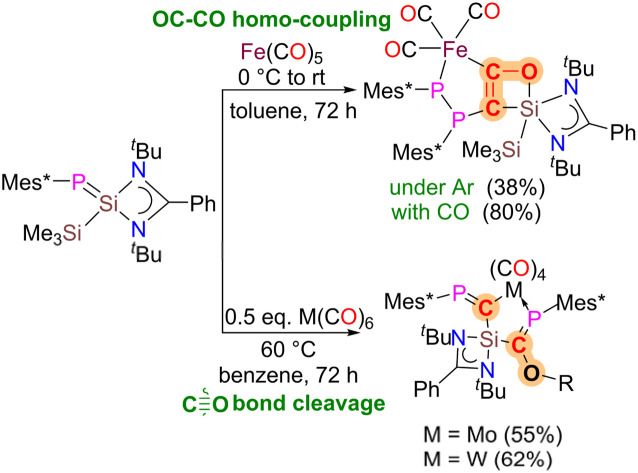

## Introduction

**Scheme 1:**
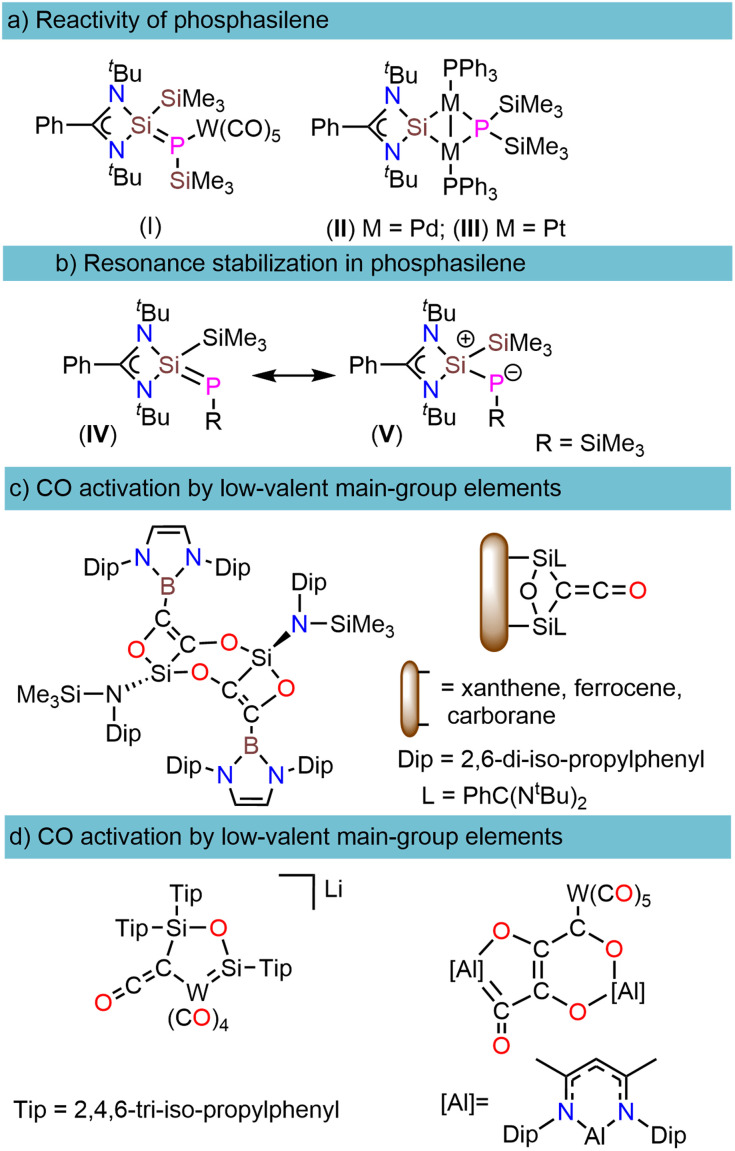
(a) Reactivity of phosphasilenes with early and late transition metals. (b) Resonance stabilization in phosphasilene (c) CO activation by low-valent main group B and Si elements. (d) CO activation by low-valent main group Al and Si elements assisting metal carbonyls and a reductant.

Phosphasilenes (RP=SiR_2_), heavier analogs of imines, are highly reactive compounds that show unique reactivity with the main-group elements and transition metals.^1,2^ Following the first spectroscopic analysis by Bickelhaupt in 1984 (ref. 3) and the subsequent structural determination by Niecke in 1993,^4^ several efforts have been undertaken to isolate stable phosphasilenes and investigate their reactivity.^2,5–11^ These investigations mainly concentrate on two types: (i) with the main-group and transition metals such as Au, Zn, Cr, Mo, W, and Pb, phosphasilenes often exhibit κ^1^-P-coordination to the metal center^12–14^ whereas in the case of M(0) salts (M = Ni, Pd, Pt), rearrangement occurs through cleavage of the phosphorus-silicon double bond (Scheme 1a), and (ii) with small organic molecules such as ketones, aldehydes, P_4_, acetylenes, nitriles, and azides which resulted in the insertion of these molecules into the Si=P double bond.^2,15–18^

The unusual reactivities of phosphasilenes can be attributed to the polarization of the (–P^(*δ*−)^=Si^(*δ*+)^<) double bond (Scheme 1b)^16^ and small HOMO–LUMO energy gap, which is achieved through modifications of the substituents at the P and Si atoms, and by the coordination of a P lone pair to transition metals or main-group elements.^2^ However, to the best of our knowledge, reactivity studies of phosphasilenes with small molecules such as CO, CO_2,_ N_2,_ and H_2_ are currently lacking. Among these small molecules, CO activation/functionalization represents one of the most important strategies for utilizing CO molecule as a C1 source in producing bulk and fine chemicals.^19^

It is common knowledge that transition metal complexes play a major role in the activation of the CO bond due to its high C≡O bond dissociation energy (BDE = 1077 kJ mol^−1^).^20,21^ In recent years, low-valent main-group elements have shown tremendous development in CO activation chemistry.^20^ Among different types of main group compounds that demonstrate the capability of CO activation, such as B, Si, and Al, silicon in the form of silylenes have exhibited exceptional efficiency that can be attributed to the small HOMO–LUMO energy gap, a lone pair, and a free p-orbital at the silicon center.^22–26^ As depicted in (Scheme 1c), in such reactions, carbon monoxide (CO) reduction is succeeded by CO homologation, leading to the formation of (C_*n*_O_*x*_) chains, which play a pivotal role in generating carbon building blocks.^24–26^ It is worth noting that CO homologation can be achieved not only through the use of transition metal complexes or low-valent main group elements but also in conjunction with auxiliary metal carbonyls and a reductant (Scheme 1d).^27,28^

These reductants encompass a spectrum ranging from metallocenes (M = Zr, Hf, Sm) to KC_8_.^27,29^ For these types of reactions, there are reports documenting the use of M(I) (M = Al, Mg) complexes and compounds with Si=Si bond, which pose considerable synthetic challenges.^28,30,31^ To the best of our knowledge, there have been no reports of using phosphasilenes in this type of reaction, either directly or in conjunction with another transition metal.

Given the fact that the polarization of the double bond in phosphasilenes can be adjusted and since they have important characteristics for CO activation, such as a low HOMO–LUMO energy gap, an electrophilic silicon center, and the ability to interact with transition metals, we synthesized a sterically demanding base-stabilized phosphasilene (Mes*PSi(SiMe_3_)(PhC(N^*t*^Bu)_2_) (1) and studied its reactivity with CO (gas) and metal carbonyls (M = Fe, Mo and W). Although base-stabilized phosphasilene 1 does not show any reaction with CO (gas), it interestingly facilitates CO activation and deoxygenative homo coupling of CO molecules when treated with metal carbonyls, leading to the formation of the rare complexes 2–4. The details are described.

## Results and discussion

**Scheme 2:**
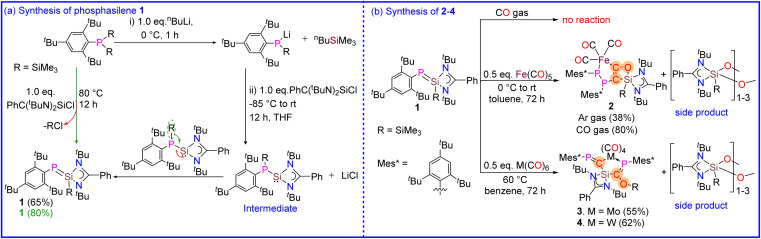
(a) Synthesis of 1. (b) Synthesis of 2–4.

The heterolyptic chlorosilylene [(PhC(N^*t*^Bu)_2_SiCl]^32^ and Mes*PLi(SiMe_3_) (Mes* = 2,4,6-^*t*^Bu_3_C_6_H_2_) were first chosen as the starting precursors. A 1 : 1 salt metathesis reaction of [(PhC(N^*t*^Bu)_2_SiCl] with an *in situ* generated Mes*PLi(SiMe_3_) salt in THF unexpectedly resulted in the formation of phosphasilene (Mes*PSi(SiMe_3_)(PhC(N^*t*^Bu)_2_) (1) as orange-yellow solid in good yield, in place of phosphinosilylene (Mes*P(SiMe_3_)Si(PhC(N^*t*^Bu)_2_) (Scheme 2a). This contrasts with the previous report, where phosphasilene formation could only be achieved by heating the phosphinosilylene over 100 °C for approximately four days, facilitating the transfer of the SiMe_3_ group from phosphorus to the Si atom.^33^ This interesting difference in reactivity might be due to the presence of the bulky Mes* group at the phosphorus center. Interestingly, phosphasilene 1 can be readily synthesized in a single step by heating a toluene solution of (Mes*)P(SiMe_3_)_2_ and [(PhC(N^*t*^Bu)_2_SiCl] in a 1 : 1 molar ratio for 8 hours (see ESI† for details). This straightforward synthesis of 1 contrasts prior studies, where phosphasilene formation was typically achieved under harsh reaction conditions, using harsh reducing agents or with the help of catalysts and bases during the reaction.^10,34–36^ It should be noted that 1 is a highly air and moisture-sensitive compound. However, it is stable in solid and solution states under an inert atmosphere at room temperature for months, and no decomposition was observed. Furthermore, the phosphasilene 1 exhibits very high thermal stability and shows no signs of decomposition when heated up to 120 °C. Considering the mentioned properties, we decided to treat compound 1 with transition metal carbonyl complexes (Scheme 2b) and compare its reactivity with previously reported investigations. Surprisingly, unlike most reported reactions involving phosphasilenes that primarily exhibit simple κ^1^-P-coordination, this compound undergoes a unique reaction with CO groups released during the reaction with metal carbonyls, resulting in the formation of compounds 2–4 and a side product, which according to NMR and IR spectroscopies and mass spectrometry data, is considered to be an oxygenated silylene compound which will be discussed later. Although phosphasilene 1 does not react with CO (gas), interestingly, when treated with Fe(CO)_5,_ it afforded a rare Fe complex 2. In this reaction, two CO molecules released during the reaction couple together to form a ketene μ-(CCO). This ketene is trapped between Fe and Si atoms in 2. Additionally, silanone derivative either in the form of monomer or dimer/trimer {PhC(N^*t*^Bu)_2_Si(SiMe_3_)O–}_*n*_ (*n* = 1–3) is eliminated as a side product during the reaction.

In the case of -treatments with M(CO)_6_ (M = Mo and W), the reactions were carried out at 60 °C, resulted in the formation of two interesting complexes (3 and 4) where the deoxygenation of one CO molecule without CO–CO homocoupling is observed, which is in contrast with the previous reports that the complete deoxygenation has been afforded with the help of homologation with another CO molecule.

**Fig. 1:**
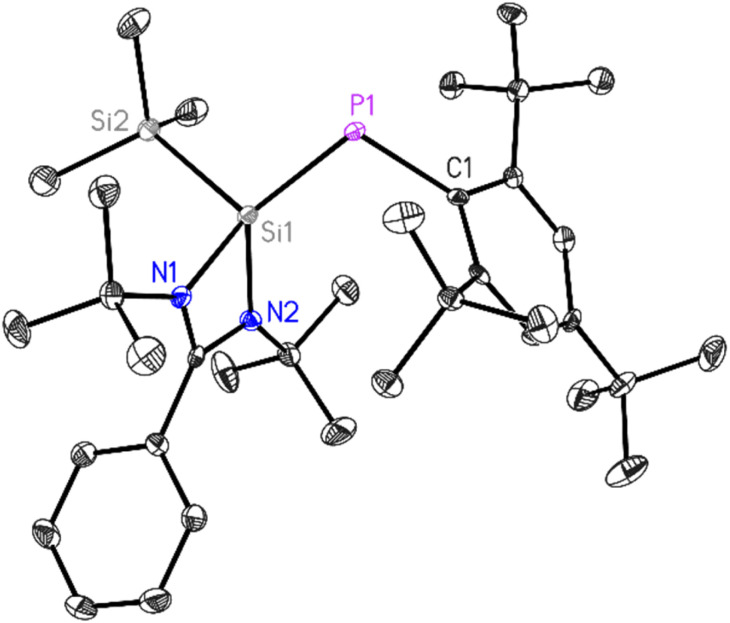
Molecular structure of 1. The anisotropic displacement parameters are depicted at the 50% probability level. Hydrogen atoms have been omitted for clarity. Selected bond lengths (Å) and bond angles (°). P1–C1 1.874(2), P1–Si1 2.1250(10), N1–Si1 1.888(2), N2–Si1 1.833(2), Si1–Si2 2.3501(12), N1–Si1–N2 70.39(9), N1–Si1–Si2 106.67(7), N2– Si1–Si2 110.16(8), P1–Si1–Si2 104.80(4), C1–P1–Si1 109.30(8).

**Fig. 2:**
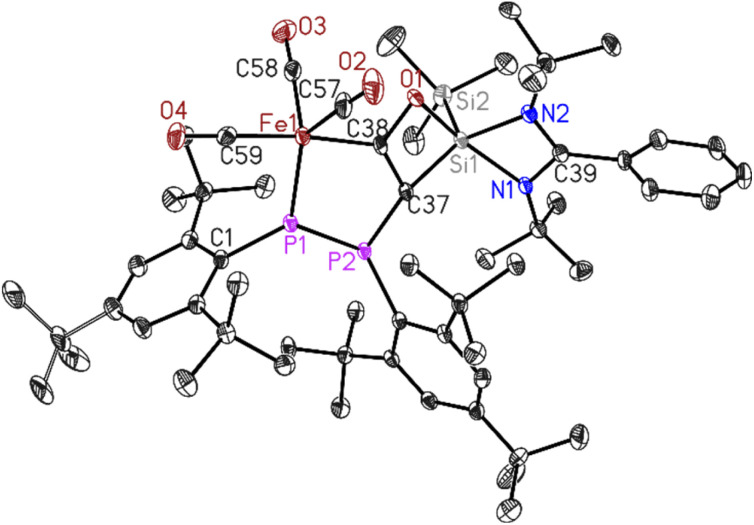
Molecular structure of 2. The anisotropic displacement parameters are depicted at the 50% probability level. Hydrogen atoms have been omitted for clarity. Selected bond lengths (Å) and bond angles (°). Fe1–P1 2.1133(7), Fe1–C38 1.982(3), Fe1–C57 1.803(3), C57–O2 1.134(3), O1–Si1 1.8093(19), Si1–Si2 2.3453(10), C37–C38 1.371(4), C38–O1 1.366(3)Å, P1–P2 2.2028(9), C1–P1–P2 116.60(8).

**Fig. 3:**
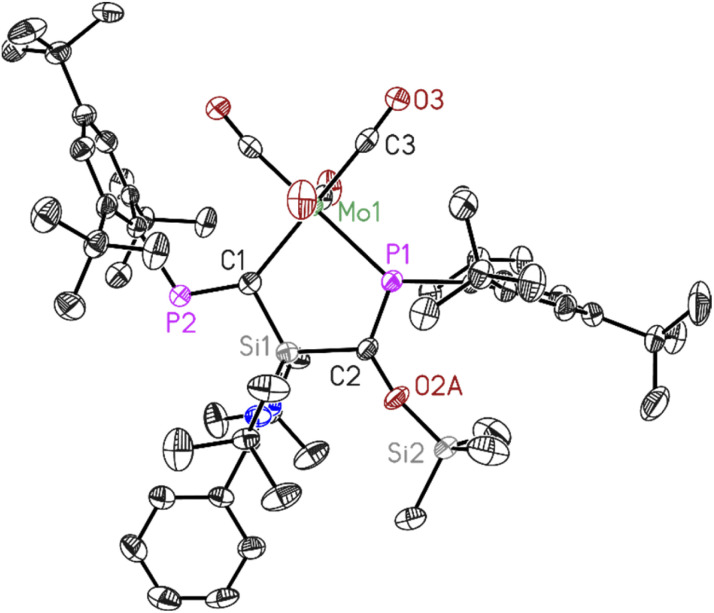
Molecular structure of 3. The anisotropic displacement parameters are depicted at the 50% probability level. Hydrogen atoms have been omitted for clarity. Selected bond lengths (Å) and bond angles (°). Mo1–P1 2.5051(11), Mo1–C1 2.323(4), C1–P2 1.679(4), C2–P1 1.678(4), C1–Si1 1.817(4), C2–Si1 1.868(4), C2–O2A 1.401(10), O2A–Si2 1.653(9), P1–Mo1–C1 86.24(10), C1–Si1–C2 115.66(18), P1–C2–Si1 112.7(2), Mo1–C1–Si1 111.93(19), Mo1–P1–C2 113.43(14).

**Fig. 4:**
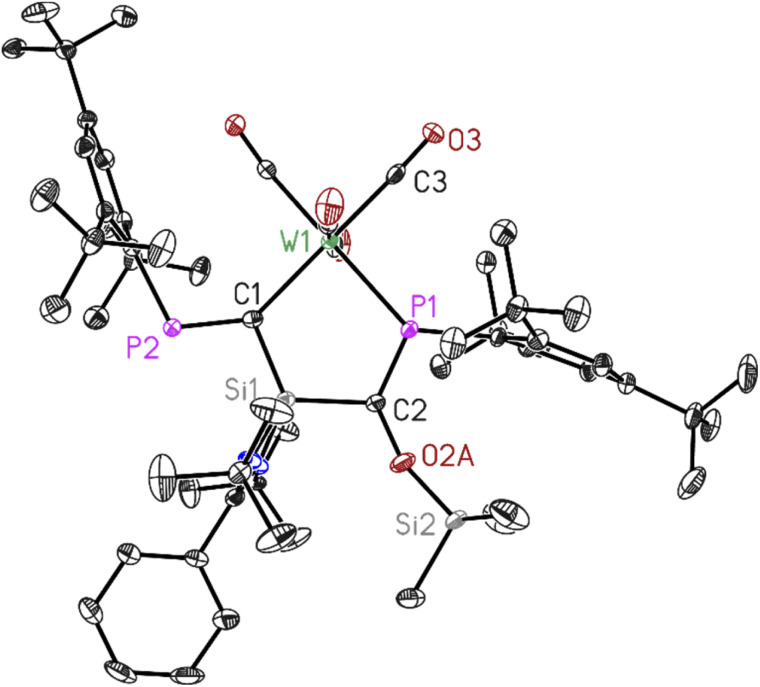
Molecular structure of 4. The anisotropic displacement parameters are depicted at the 50% probability level. Hydrogen atoms have been omitted for clarity. Selected bond lengths (Å) and bond angles (°). W1–P1 2.4933(7), W1–C1 2.294(2), C1–P2 1.690(3), C2–P11.686(3), C1–Si1 1.821(2), C2–Si1 1.861(3), C2–O2A 1.389(9), O2A–Si2 1.657(8), P1–W1–C1 86.12(6), C1–Si1–C2 115.01(11), P1–C2–Si1 112.28(13), W1–C1–Si1 112.86(12), W1–P1–C2 113.65(9).

To further investigate the effect of CO on the reaction yield, the reaction of 1 with metal carbonyls were conducted in the presence of CO gas, which resulted in the enhancement of the product yields. Compounds (1–4) were thoroughly characterized using NMR spectroscopies, mass spectrometry, elemental analysis, and X-ray diffraction analysis^37–41^(Fig. 1–4).

The ^31^P NMR spectrum of 1 (Fig. S3†) displayed a singlet resonance at *δ* −92 ppm with two types of characteristic ^29^Si satellites attributed to the coupling with Si1 [PhC(^*t*^BuN)_2_Si], (^1^*J*_PSi_; 241.37 Hz) and Si2 [SiMe_3_], (^3^*J*_PSi_; 54.26 Hz) atoms. Whereas, the ^29^Si NMR spectrum of 1 (Fig. S4†) revealed two doublets centered at *δ* 16.59 ppm (Si1 [PhC(^*t*^BuN)_2_Si], ^1^*J*_SiP_; 241.37 Hz) and *δ* −16.04 ppm (Si2, [SiMe_3_], ^3^*J*_SiP_; 54.26 Hz)) resulting from the coupling with the phosphorus atom. The LIFDI mass spectrum (Fig. S7†), showed molecular ion for [M]^+^ at *m*/*z* = 608.5, which is well-matched with its simulated isotopic pattern confirming phosphasilene 1 formation. The UV-vis spectrum of 1 in pentane demonstrated a broad absorption band with a maximum at *λ* = 353 nm, a value that is similar to those observed for polarized phosphasilenes.^42^

The block-shaped orange-yellow color crystals of 1 suitable for X-ray diffraction analysis were grown from a saturated diethyl ether solution of 1 at room temperature over 24 h. Compound 1 crystallizes in the monoclinic *P*2_1_/*n* space group (see Table S1†), with one molecule in the asymmetric unit. The P1–Si1 bond length in 1 is 2.1250(10) Å, slightly longer compared to the same bond (2.095(3) Å) in ((SiMe_3_)P=Si(SiMe_3_)(PhC(N^*t*^Bu)_2_) and shorter than the P–Si bond lengths (2.2264(13) and 2.2321(12) Å) in ((SiMe_3_)_2_P–Si(PhC(N^*t*^Bu)_2_).^33^ This provides additional evidence for double bond formation in phosphasilene 1. The P1–Si1 bond length of 2.1250(10) Å is consistent with values reported for phosphasilenes with three-coordinate silicon atoms.^5–7^

Dark green crystals of 2 suitable for X-ray diffraction analysis were grown from saturated *n*-hexane solution at room temperature over three weeks. Compound 2 crystallizes in the triclinic space group *P*1̄ (see ESI Table S1†) with one molecule in the asymmetric unit. The molecular structure of 2 confirms the deoxygenated homo-coupling of two CO molecules resulting in the formation of unique five- and four-membered rings containing P–P–Fe–C–C and C–C–O–Si atoms, respectively. The bond length of C37–C38 is 1.371(4) Å, falling within the range typical for C=C double bonds, while the C38–O1 bond length of 1.366(3) Å corresponds to C–O single bonds. The ^31^P NMR spectrum of 2 (Fig. S11†) revealed two doublets centered at *δ* 54.5 and 387.6 ppm with ^1^*J*_PP_ coupling of 460 Hz for (Mes*P–PMes*) group. This significant coupling aligns with reported compounds containing phosphorus–phosphorus bonds.^43^ Whereas, the ^29^Si NMR spectrum of 2 (Fig. S12†) showed two shielded singlets around *δ* −14.33 and −86.43 ppm, assigned to the (SiMe_3_) group and [PhC(N^*t*^Bu)_2_Si] group respectively.

The up-field shift is attributed to the change in the oxidation state of Si1 from (II) to (IV) and the presence of electron-withdrawing atoms, such as oxygen and carbon, around this five-coordinate silicon center. The LIFDI mass spectrum of 2 (Fig. S16†) showed molecular ion peak at *m*/*z* 1064.3 confirming the formation of 2.

Dark red crystals of 3 and 4 suitable for X-ray diffraction analysis were grown from saturated benzene-d_6_ solution at room temperature over one day. Both compounds 3 and 4 crystallize isomorphously in the orthorhombic space group *P*2_1_2_1_2_1_ (see ESI Table S1†) with one molecule and five benzene molecules as lattice solvent in the asymmetric unit. The molecular structures confirm the deoxygenation of one CO molecule followed by trapping another CO molecule in between the Si–P bond resulting in the formation of unique five-membered ring containing M–C–Si–C–P atoms (M = Mo, W). The important bond lengths for 3 and 4 are summarised in (Table 1). According to the data presented there, the P–C bond lengths align with the typical range observed for P=C double bonds, whereas the C–O bond lengths are consistent with C–O single bonds.

**Table 1:** Important bond lengths of compound 3 and 4

Bond length (Å)	P1–C2	P2–C1	C2–O2A	M–P1
3	1.678(4)	1.679(4)	1.401(10)	2.5051(11)
4	1.686(3)	1.690(3)	1.389(9)	2.4933(7)

Both compounds 3 and 4 are stable in an inert atmosphere for a prolonged period. However, they undergo rapid decomposition in polar solvents such as tetrahydrofuran (THF) and toluene, within 4–5 hours. Due to their limited solubility in deuterated benzene (C_6_D_6_), nuclear magnetic resonance (NMR) spectroscopy was conducted using toluene (d_8_) and THF (d_8_) (Fig. S18–S21 and S24–S30†).

Despite the suboptimal solubility of compound 3 in toluene-d_8_, it was selected as the solvent due to the rapid decomposition observed in other deuterated solvents such as THF-d_8_ and dichloromethane (d_2_) (CD_2_Cl_2_). The ^31^P NMR spectrum of 3 (Fig. S20†) revealed two peaks centered at *δ* 481.41 and 249.7 ppm, and ^29^Si-NMR spectrum displayed two peaks at *δ* 14.30 (attributed to Si2, OSiMe_3_) and −16.43 (attributed to Si1 [(Si(PhC(N^*t*^Bu)_2_])). The phosphorous–phosphorous and silicon-phosphorous couplings were not clearly observed in the ^31^P and ^29^Si NMR spectra due to poor solubility of 3.

With compound 4, the ^31^P-NMR spectrum showed two doublets about *δ* 466.9 and 216.8 ppm with a ^3^*J*_PP_ coupling of 7.8 Hz. The peak at *δ* 216.8 ppm exhibited two characteristic satellites due to coupling with the silicon atoms (^2^*J*_PSi_ = 247.53 Hz and ^3^*J*_PSi_ = 112.23 Hz) (Fig. S29†).

The ^29^Si NMR spectrum of 4 (Fig. S30†) showed two peaks, one singlet at *δ* 16.28 ppm and a doublet of doublets centred at *δ* −11.48 ppm, assigned to the (SiMe_3_) group and [PhC(N^*t*^Bu)_2_Si] group, respectively.

The LIFDI mass spectra of compounds 3 and 4 (Fig. S22 and S31†) revealed molecular ion peaks at *m*/*z* 1106.3 and 1192.3, respectively, indicating the loss of one CO molecule during ionization for both compounds. As it is obvious from the characteristic results, this reactivity pattern diverges from the reaction of 1 with Fe(CO)_5_ and the reactions of the same category, where CO deoxygenation typically proceeds *via* CO–CO homogenization.^17,23^ Importantly, our investigations with M(CO)_6_ (M = Mo, W) did not manifest homocoupling of CO molecules.

Given the initial compounds and the reaction products, it is evident that one oxygen atom from the metal carbonyls and the [(PhC(N^*t*^Bu)_2_Si(TMS)] fragment of the phosphasilene compound are missing. To characterize the side product, we analyzed the residual reaction mixture using ^29^Si-NMR spectroscopy. Compression of the^29^ Si-NMR (Fig. S33†) of all three residual mixtures revealed two signals in the same region in all the cases suggesting the similar side product in all of them. Fortunately, we could purify the side product from the residual reaction mixture of the iron complex and characterize it using NMR, IR spectroscopies and mass spectrometry (Fig. S34–S37†). The ^29^Si NMR data of the residual mixture suggest that the side product is a silanone derivative, which was further confirmed by ^1^H NMR spectrum and mass spectrometry data.^44–46^ However, all attempts to grow a single crystal for X-ray diffraction analysis were unsuccessful; we consistently obtained a white solid that was amorphous and, therefore, non-diffracting. Although we are not very sure about it, we rationalize that this most probably is due to the polymerization of the silanone side product during the reaction. Silanones lacking bulky substituents are challenging to stabilize and tend to polymerize readily to form polysiloxane. However in the following reaction, the bulky substituents on the silicon in PhC(N^*t*^Bu)_2_Si(SiMe_3_)O likely hinder extensive polymerization, favoring the formation of dimers or trimers of the silanone {PhC(N^*t*^Bu)_2_Si(SiMe_3_)O–}*n* (*n* = 1–3), rather than a polymer with *n* = ∞.^47–49^

To understand the detailed atomic-level mechanisms for the formation of products 2, 3, and 4 from the reaction of 1 with Fe(CO)_5_, Mo(CO)_6_, and W(CO)_6_, respectively, free energy profiles were mapped at the B3LYP-D3/6-31G* Mo, W (LanL2DZ) level of theory (refer to the ESI† for computational details).^50,51^ The free energies for the reaction of 1 with Fe(CO)_5_ were calculated at room temperature, whereas for the reaction of 1 with W(CO)_6_ and Mo(CO)_6_, they were calculated at 333.15 K. For simplicity of the calculations, the *tert*-butyl substituents of the aryl groups attached to the phosphorus atoms of 1 and nitrogen atoms of the benzamidinate ligand were replaced by methyl groups, and the resulting structure was labelled as 1′. The final products thus formed are denoted as 2′, 3′, and 4′, respectively. The free energy profile for the reaction of 1′ with Fe(CO)_5_ is shown in (Fig. S45†). The HOMO (−4.11 eV) molecular orbital of 1 corresponds to a Si=P π-type orbital, while the LUMO (−0.83 eV) is a π*-type orbital of the phenyl ring of the benzamidinate ligand (Fig. S45(b)).† NBO analysis of 1 reveals that the HOMO orbital has a predominant electron density at the phosphorus atom, which is available for bond formation with incoming Fe(CO)_5_. Initially, 1′ and Fe(CO)_5_ combine to form the phosphasilene–iron complex, Fe-int1, releasing one CO molecule.

The CO molecule released from the previous step can be inserted into the P–Si bond of Fe-int1 through the transition state Fe-ts1′, producing Fe-int2′. However, this process requires a high energy barrier of 66.88 kcal mol^−1^, and hence is unfeasible under the experimental condition. An alternative scenario, in which Fe-int1 reacts with a second 1′ molecule to generate Fe-int2 while releasing another CO molecule, has been studied. It is worth noting that the *in situ* monitoring of the reaction by ^31^P NMR spectroscopy showed the formation of Fe complex 2 together with a new peak at *δ* 200 ppm, which can be attributed to Fe-int2 (Fig. S38†). The large downfield shift in the ^31^P NMR resonance signal compared to phosphasilene 1 might be due to the coordination of P lone pair to the Fe(CO)_3_ moiety which results in reduced electron density on the phosphorus atom. A similar trend is observed in the ^31^P NMR spectrum for the M(0) complexes of phosphasilenes, further suggesting Fe-int2 formation.^5,52^ Although monitoring of ^31^P-NMR suggests the formation of Fe-int1 and Fe-int2, the high energy barrier between Fe-int1 and Fe-ts1 (+43.68 kcal mol^−1^) led us to include the mechanism diagram and its discussion in the ESI (see Fig. S45†).

**Fig. 5:**
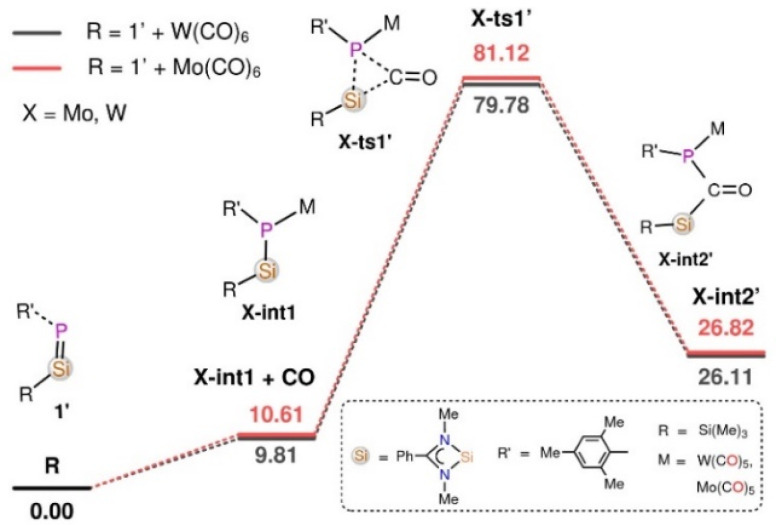
The Gibbs free energy profile for the reaction of 1′ with Mo(CO)_6_ and W(CO)_6_ obtained at the B3LYP-D3/6-31G* Mo, W (LanL2DZ) level of theory for the direct insertion of CO molecules into the Si–P bonds. The energies are given in kcal mol^−1^.

**Fig. 6:**
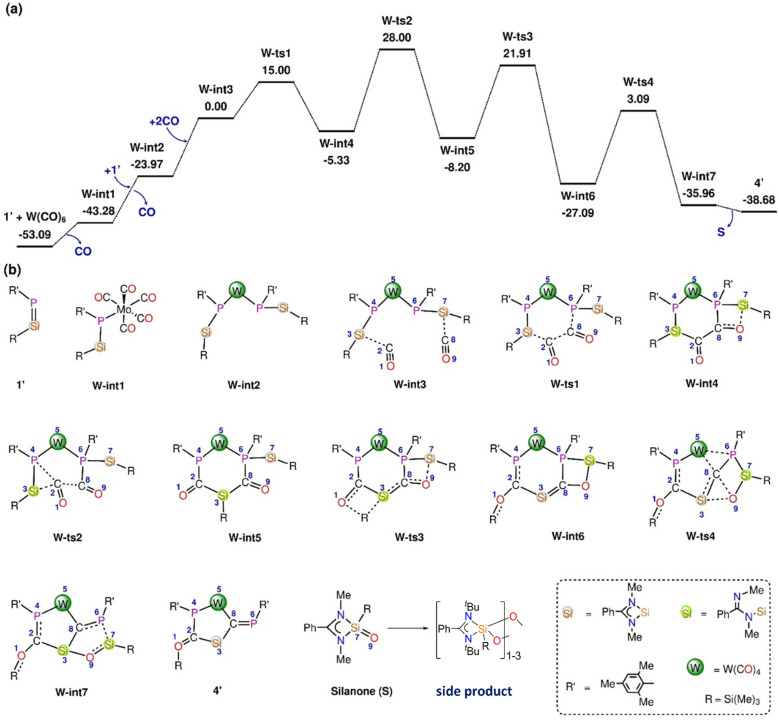
(a) The Gibbs free energy profile for the reaction of 1′ with W(CO)_6_ was obtained at the B3LYP-D3/6-31G* level of theory. (b) Different stationary point structures existing along the reaction pathway. The substituents connected to Si, W, and P are omitted in the images for clarity. The energies are given in kcal mol^−1^.

In the reaction of 1′ with Mo(CO)_6_ and W(CO)_6_, deoxygenative CO activation of one carbonyl group and the trapping of another CO molecules in between the Si and P atoms was observed which results in the products 3 and 4, respectively. In order to understand the atomic-level mechanisms for the formation of the products 3 and 4, the free energy profiles for the CO insertion between the P–Si bonds were mapped (Fig. 5 and 6) for different pathways. As can be seen from Fig. 5, the approach of 1′ and Mo(CO)_6_ or W(CO)_6_ leads to the formation of the phosphasilene-X complex, X-int1, with the release of one CO molecule, where X = Mo and W. The CO molecule released from the previous step may be inserted into the P–Si bond of X-int1 through the transition state X-ts1′ to form X-int2′. The CO insertion energy barrier for Mo and W is 70.51 and 69.97 kcal mol^−1^, respectively. The energy barrier for the insertion of CO into the P–Si bond is very high, and this reaction is not viable under the experimental conditions. An alternative multi-step mechanism involving a homo-coupling of CO moieties followed by CO insertion was also mapped (Fig. 6) This mechanism involves the initial formation of an activated complex X-int3, similar to that obtained for the reaction of 1′ with Fe(CO)_5_, by the reaction of X-int1 with a second molecule of 1′. It is important to note that the X-int3 structure formed here is quite different from that of Fe-int3. In X-int3, the P–X–P angle is about 77°, and the Si groups are well-separated due to crowding (see ESI Table S6†). However, in Fe-int3, the P–Fe–P angle is 170°, and a CO group attached to the Si(7) atom has weak interactions with the Fe atom. These structural differences in X-int3 result in different pathways being followed in the reactions involving Fe and Mo/W. From X-int3, due to the close proximity of the P(6) to the Si(3) group, a six-membered intermediate X-int4 is formed by the homo-coupling of CO moieties *via*X-ts1. From X-int4, CO insertion into the P(4)–Si(3) happens *via*X-ts2 to result in the bicylic X-int5. Then the migration of the –Si(Me)_3_ group from the Si(3) moiety to the carbonyl O(1) results in the formation of X-int6. This is followed by the rearrangement of X-int6*via* the breaking of the W(5)–P(6) and C(8)–O(9) bonds and the formation of W(5)–C(8) and O(9)–Si(3) bonds to result in X-int7. In X-int7, the P(6)–Si(7) bond is stretched (2.37 Å), leading to the elimination of the silanone (S) in the following step. The elimination of silanone (S) from X-int7 gives the product 4′. The rate-determining transition state is X-ts2, which corresponds to the insertion of CO into the P(4)–Si(3) bond, which has a barrier of ∼28 kcal mol^−1^ with respect to the activated complex X-int3. The reaction of 1′ with Mo(CO)_6_ follows a route similar to that of the reaction of 1′ with W(CO)_6_ to form the product 3′ (Fig. S46†).

Bubbling CO gas into the THF solution of compound 1 was undertaken to assess its capability to activate CO molecules. The ^31^P-NMR monitoring revealed no discernible changes in the spectrum. This underscores that the activation of CO occurs exclusively in the presence of two adjacent silicone atoms, consistent with observations in the reaction mechanism. The formation of int2 is imperative for this reaction, providing additional evidence supporting the validity of the proposed mechanism.

## Conclusions

In summary, this work presents a new sterically demanding phosphasilene (Mes*PSi(SiMe_3_)(PhC(N^*t*^Bu)_2_)) and its unexpected reaction of Si=P double bond with metal carbonyls (M = Fe, Mo and W). The coordination of P lone pair to M center resulted in polarization of Si=P double bond which in turn increased the electrophilicity of the Si center, therefore in the case of treatment with Fe(CO)_5_, it mediates the deoxygenative homocoupling of two CO molecules, yielding the ketene-inserted rare Fe complex 2. Contrastingly, reaction with Mo(CO)_6_ and W(CO)_6_ result in the deoxygenation of one carbonyl group while the second carbonyl group placed in between silicon and phosphorous atoms without any homocoupling with the first CO molecule, resulting in the formation of complexes 3 and 4, respectively. Density Functional Theory (DFT) calculations support a mechanism involving the formation of bis-phosphasilene metal complexes, M-int2 (M = Fe, Mo, W). The variation in geometrical arrangement of the two adjacent phosphasilene molecules within M-int2 leads to the formation of distinct products, with the iron complex (2) differing from the molybdenum and tungsten complexes (3 and 4).

## Data availability

The data that support the finding of this article has been included as part of the ESI.† Crystallographic data for compounds 1–4 has been deposited as the CCDC 2311389, 2279235, 2370052 and 2370053.

## Author contributions

Z. H.: conceptualization, methodology, formal analysis, investigation, writing – original draft, review & editing, visualization, R. P.: DFT calculation, writing – review & editing, K. R.: single-crystal measurement, S. M.: single-crystal measurement, M. K. P.: writing – review & editing, S. K. K.: writing – review & editing, R. H.-I.: supervision, U. L.: supervision, writing – review & editing, D. S.: supervision, writing – review & editing, H. W. R.: writing – review & editing, supervision, funding acquisition.

## Conflicts of interest

There are no conflicts to declare.

## Supplementary Material

SC-OLF-D4SC05491A-s001

SC-OLF-D4SC05491A-s002
