# Supplementary material for: Phosphasilene mediated CO activation and deoxygenative homo coupling of CO molecules in reactions with metal carbonyls
Source: Chem Sci. 2024 Oct 10;15(45):18888–95. doi: 10.1039/d4sc05491a (PMC11485134; doi:10.1039/d4sc05491a)
Supplement: SC-015-D4SC05491A-s001 [file SC-015-D4SC05491A-s001.pdf]

Supplementary Information (SI) for Chemical Science.
This journal is © The Royal Society of Chemistry 2024

Supplementary Information

Phosphasilene Mediated CO Activation and Deoxygenative Homo Coupling of CO Molecules in Reactions with Metal Carbonyls

Zohreh Hendi[a], Renuka Pardhan[b], Katharina Rachuy[a], Soheil Mahmoudi[c], Madhusudan K. Pandey[a], Saroj Kumar Kushvaha[a], Regine Herbst-Irmer[a], Upakarasamy Lourderaj[b]*, Dietmar Stalke[a]*, Herbert W. Roesky[a]*

[a] Dr. Z. Hendi, K. Rachuy, Dr. M. K. Panday, Dr. S. K. Kushvaha, Dr. R. Herbst-Irmer, Prof. Dr. D. Stalke, Prof. Dr. H. W. Roesky
Institut für Anorganische Chemie
Georg-August-Universität Göttingen
Göttingen, 37077, Germany
E-mail: hroesky@gwdg.de, dstalke@chemie.uni-goettingen.de

[b] Dr. Renuka Pakdhan, Prof. Upakarasamy Lourderaj
School of Chemical Sciences,
National Institute of Science Education and Research (NISER) Bhubaneswar,
HBNI, Khurda, Odisha 752050, India

[c] S. Mahmoudi
Institute of Inorganic Chemistry, Faculty of Chemistry
University of Vienna
Währinger Strasse 42, 1090 Vienna (Austria).

University of Vienna, Vienna Doctoral School in Chemistry. (DoSChem), Waehringer Str. 42, 1090 Vienna, Austria

Contents

I. General Information	S3
II. Synthesis and Characterization Data of **1 - 4**	S3–S40
III. Characterization Data of the side product	S40-46
IV. Crystallographic Details for Compounds **1 - 4**	S47–S70
V. Computational details	S71-S229
VI. References	S230

EXPERIMENTAL SECTION

General procedures. All air and moisture-sensitive compounds were handled and stored in an MBRAUN Glove box under an atmosphere of high purity of N_2. All manipulations were performed under an inert atmosphere of dry nitrogen or argon using standard Schlenk techniques. Commercial reagents were purchased from Sigma-Aldrich or TCI and used as received. Toluene and hexane were distilled over Na/K alloy (25: 75), and diethyl ether was distilled over potassium mirror. C_6D_6 and fluorobenzene were dried by stirring for two days over Na/K alloy and CaH_2, respectively. After three freeze-pump-thaw cycles for degassing, it was finally distilled and stored in the glove box. [Mes*PH_2] and [Mes*P$(TMS)_2$] were prepared according to the published procedures.[1,2] Other reagents were obtained from commercial sources and used after purification.

Instrumentation. The solution NMR spectra were recorded on Bruker FT spectrometers (Advance-400 or 500 MHz) at ambient probe temperatures. $^{13}C\{^1H\}$ and $^{29}Si\{^1H\}$ NMR spectra were acquired using the broadband decoupling method. The spectra were recorded in C_6D_6 solutions with C_6D_6 as an internal lock. Chemical shifts of ^1H and $^{13}C\{^1H\}$ NMR spectra are reported in ppm downfield from TMS, used as an internal standard. Positive values indicate downfield shifts. LIFDI measurements were performed on a Joel AccuTOF spectrometer under an inert atmosphere.

II. Synthesis and Characterization data of 1-2

(A) Synthesis of Mes*PSi(PhC(N^{t}Bu)$_2$)SiMe$_3$ (1).

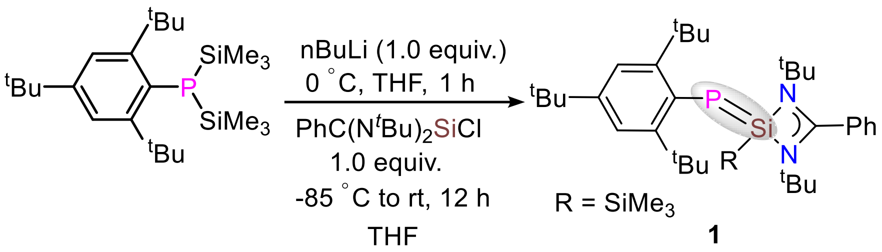

nBuLi (0.851 mL, 2.5 M in hexanes) was added dropwise to a solution of Mes*P(TMS)$_2$ (900 mg, 2.12 mmol) in tetrahydrofuran (40 mL) at 0 °C and the reaction mixture was allowed to warm to room temperature in 1 hour. Following this, a solution of [(PhC(N^{t}Bu)$_2$SiCl] (625.2 mg, 2.12 mmol) in THF (30 mL) was added dropwise at -80 °C and the reaction mixture was allowed to come to room temperature and stirred for over-night. THF was slowly reduced under vacuum to give the crude product. 40 mL of freshly dried diethyl ether was transferred to the resulting precipitate by using cannula to separate the dissolved product from LiCl salt which was produced during the reaction. The reaction mixture was filtered using a *P4* frit to remove LiCl. Diethyl ether was slowly reduced under *vaccuo* to give the pure product. The orange crystals of **1** suitable for single crystal X-ray diffraction study were obtained from a concentrated diethyl ether solution of **1** at room temperature.

Yield 65 % (842 mg).

Another alternative approach of synthesis **1** was carried out using 1;1 molar ratio of Mes*P(SiMe)$_3$ (900mg) and [(PhC(N^{t}Bu)$_2$SiCl] (625.2 mg) in a 100 mL RB flask, 40 mL freshly dried toluene was added to the flask, and it was heated at 80 °C for 8 hours. After cooling the reaction mixture to room temperature, the solvent was removed under vacuum and the product was washed with hexane. Yield 78% (1015 mg).

!!^{1}H NMR (400 MHz, C_6D_6) δ = 6.80 – 7.00 (m, 4H, ArH), 7.18 (t, J=1.57 Hz, 1H, ArH), 7.48 (d, J=1.63 Hz, 2H, ArH), 2.70 (s, 18H, –meta-$C(CH_3)_3$), 1.40 (s, 9H, para- $C(CH_3)_3$), 0.93 (s, 18H, N-^{t}Bu), 0.49 (s, 9H, $SiMe_3$).

^{13}C NMR (400 MHz, C_6D_6) δ = 170.49 (s), 155.28 (d, J=3.70), 143.96 (s), 138.63 (d, J=91.64), 131.62 (s), 129.88 (s), 128.47 (s), 120.33 (s), 65.54 (s), 53.94 (s), 38.82 (s), 34.22 (s), 33.95 (d, J=9.00 Hz), 31.61 (s), 31.25 (s), 15.23 (s), 1.03 (d, J=5.84).

^{31}P{^{1}H} NMR (400 MHz, C_6D_6) δ = -92.88 (s, with 2 silicone satellites, $^{1}J_{Psi}$= 240.16 Hz, $^{2}J_{Psi}$= 55.14 Hz)

^{29}Si{^{1}H} NMR (400 MHz, C_6D_6) δ = 16.59 (d, $^{1}J_{SiP}$= 240.16 Hz), -15.11 (d, $^{2}J_{SiP}$= 55.14 Hz)

MS (LIFDI, toluene): m/z for $C_{36}H_{61}N_2PSi_2$ $(M)^+$: 608.5.

EA experimental (calculated): C 70.82 (71.00), H 10.22 (10.10), N 4.73 (4.60).

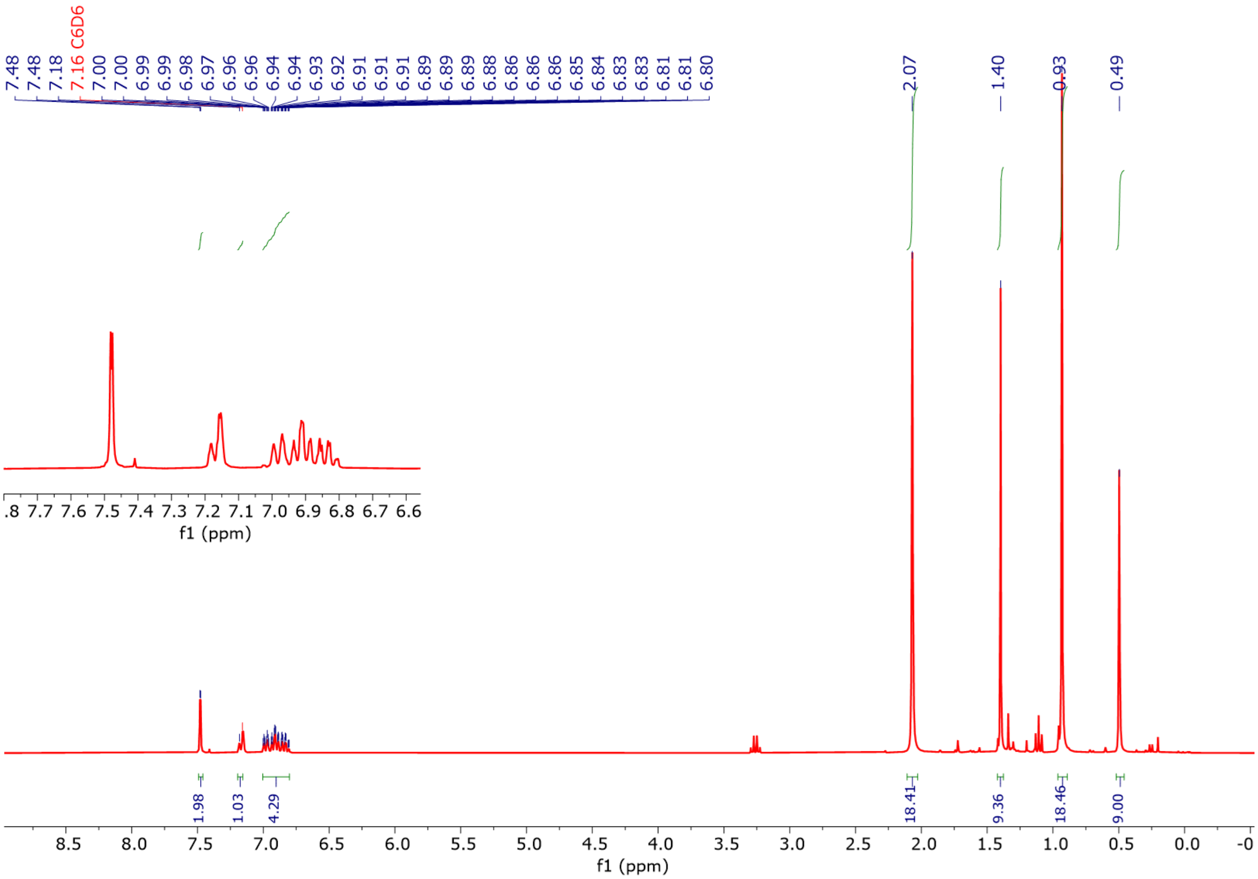

Figure S1. ^{1}H NMR spectrum of **1** in C_6D_6

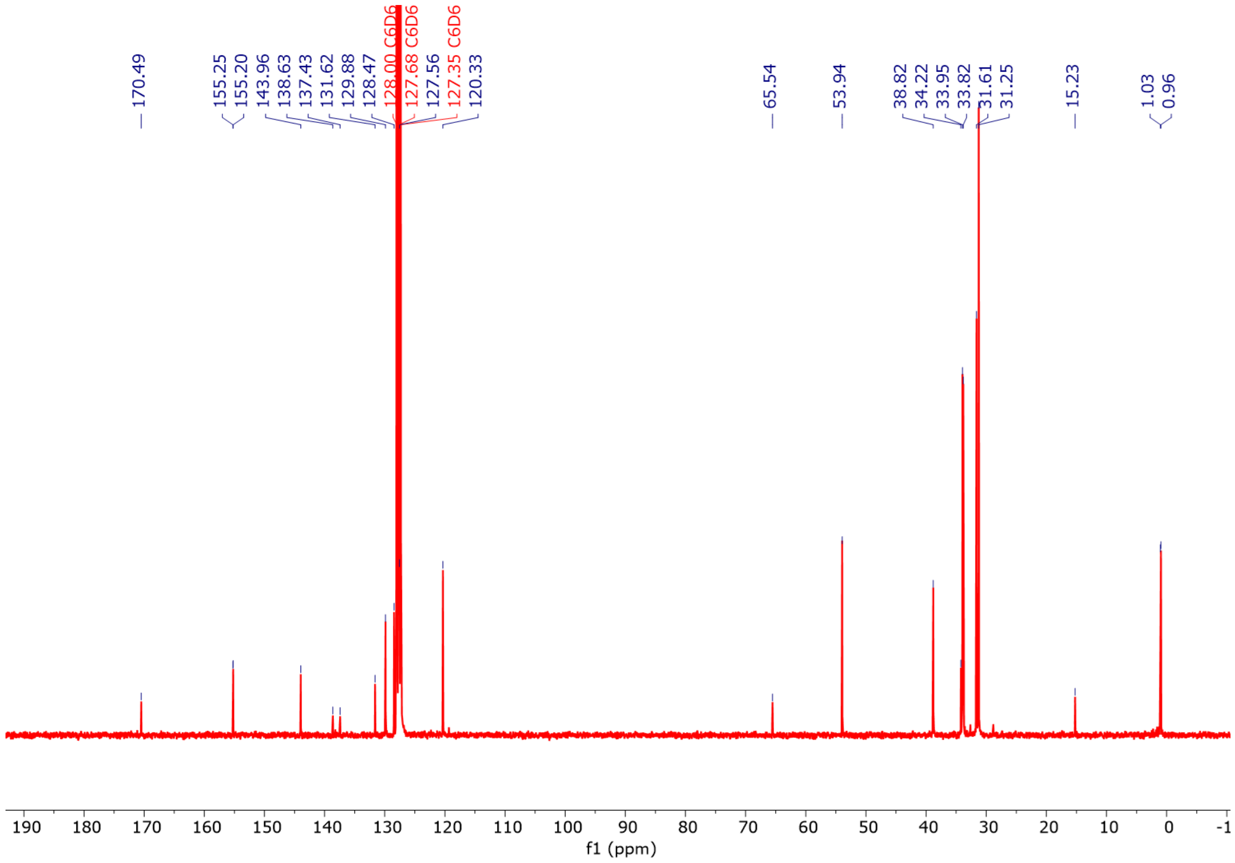

Figure S2. ^{13}C NMR spectrum of **1** in C_6D_6

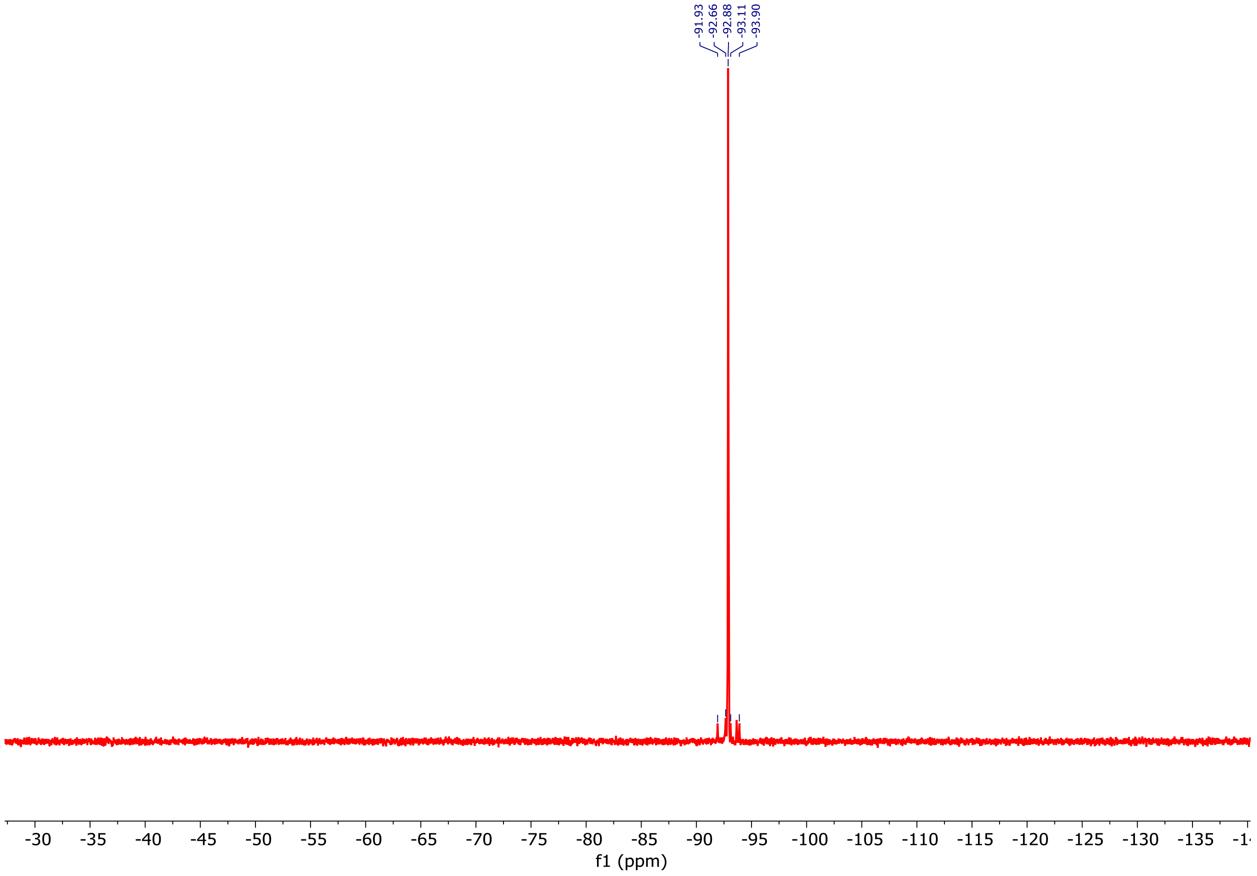

Figure S3. $^{31}P\{^{1}H\}$ NMR spectrum of **1** in C_6D_6

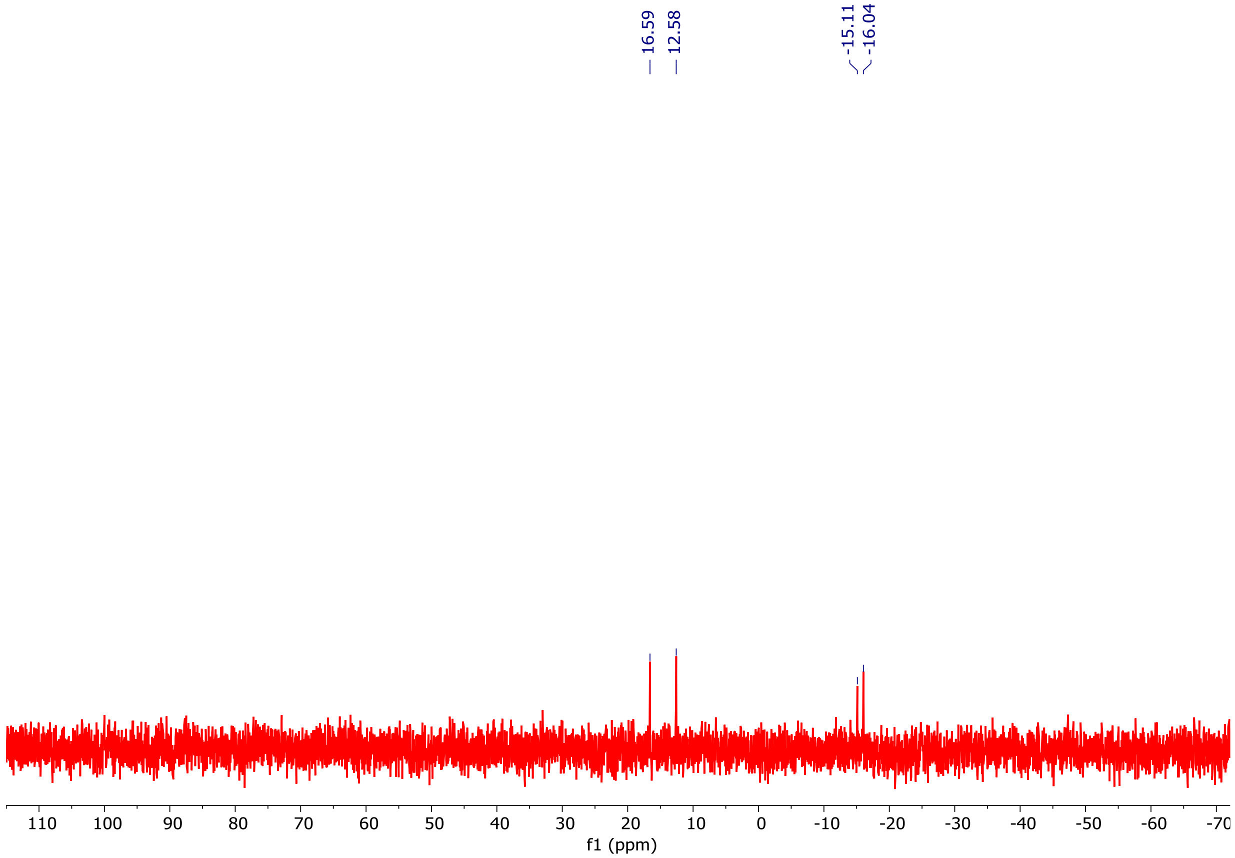

Figure S4. $^{29}Si\{^{1}H\}$ NMR spectrum of **1** in C_6D_6

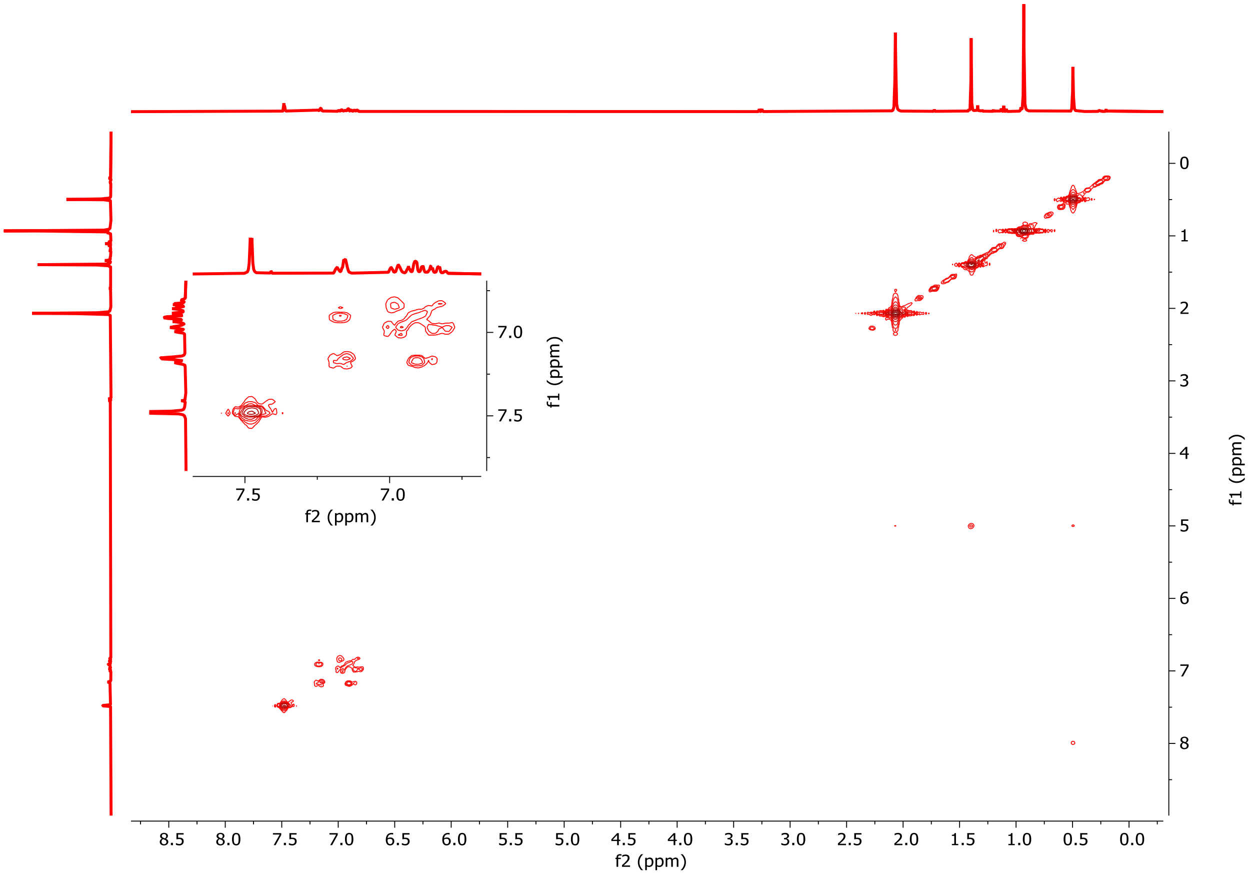

Figure S5. 2D ^1H-^1H COSY NMR spectrum of **1** in C_6D_6

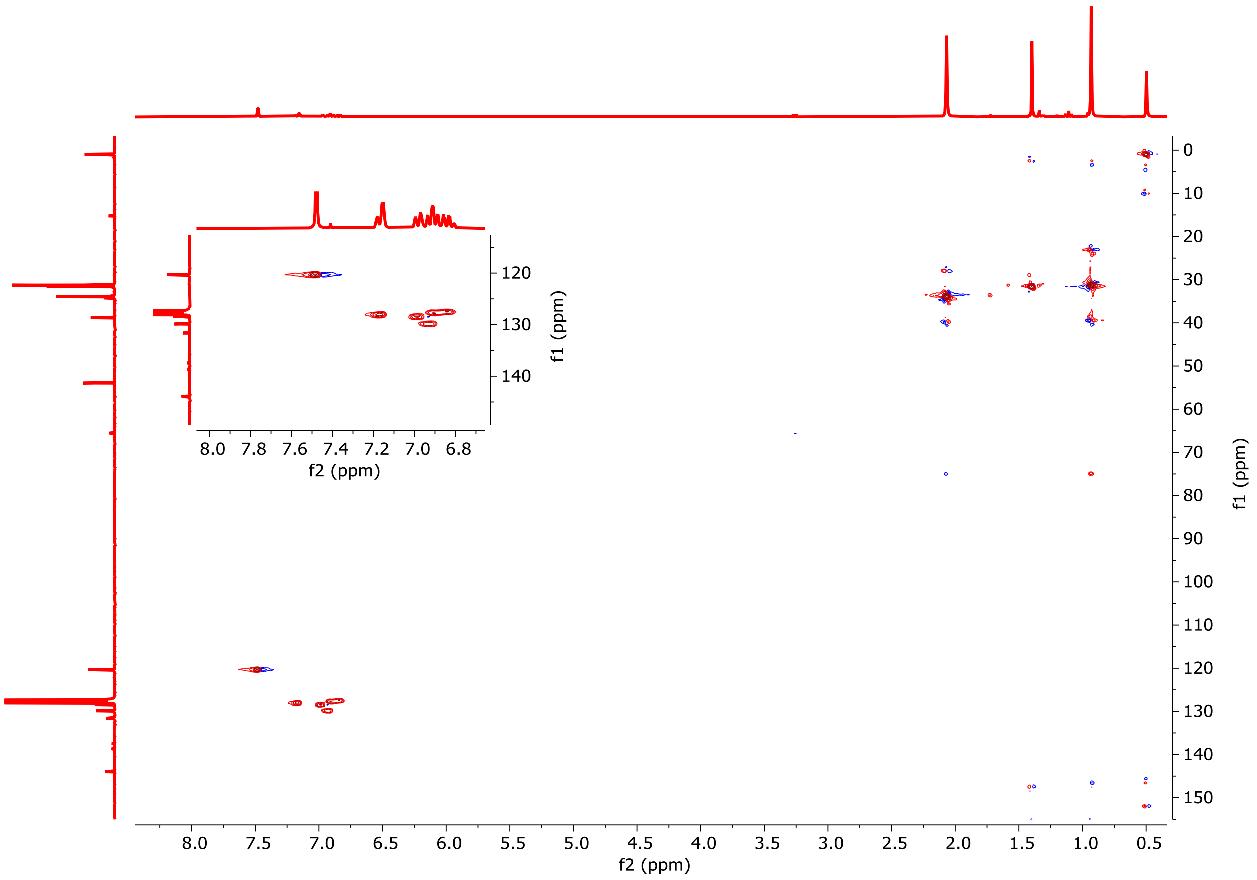

Figure S6. 2D ^{1}H-^{13}C HSQC NMR spectrum of **1** in C_6D_6

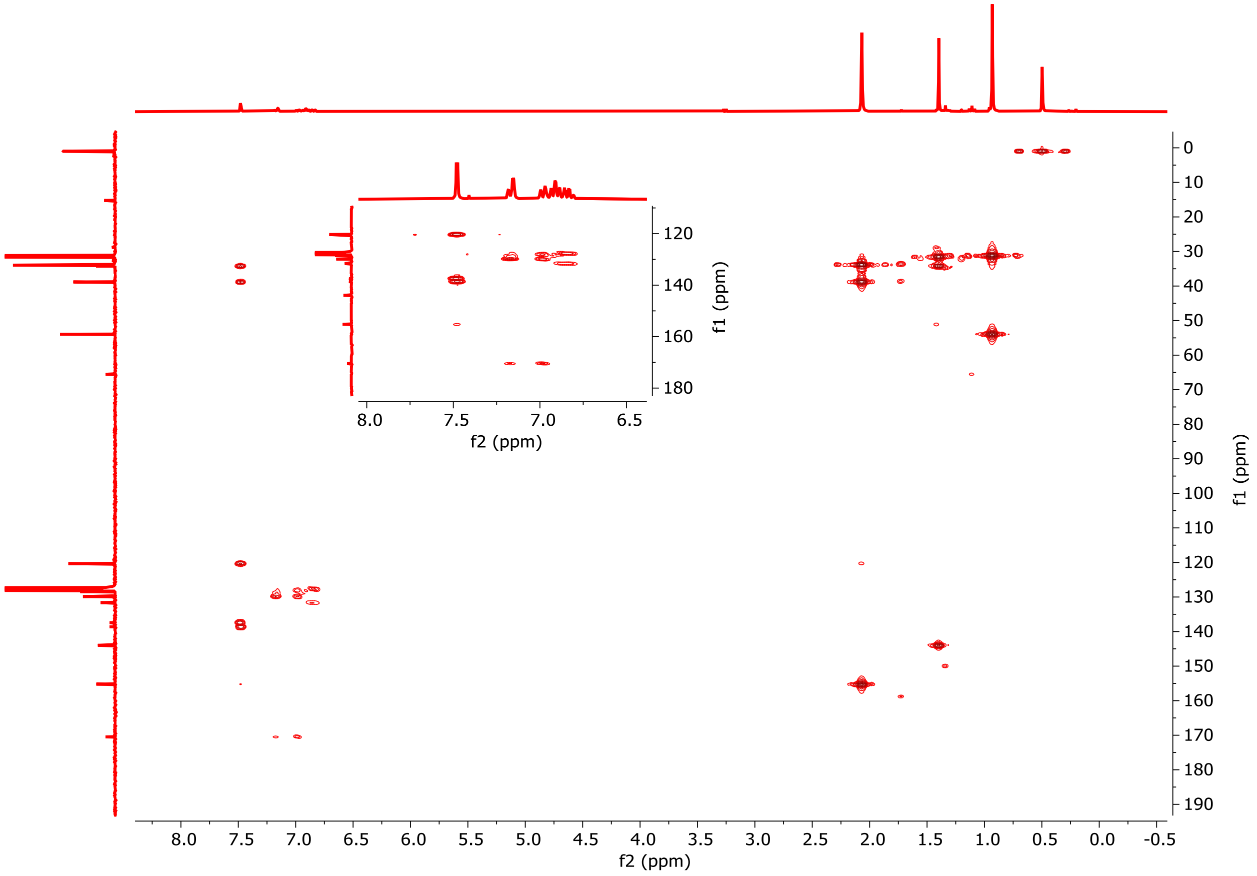

Figure S7. 2D ^{1}H-^{13}C HMBC NMR spectrum of **1** in C_6D_6.

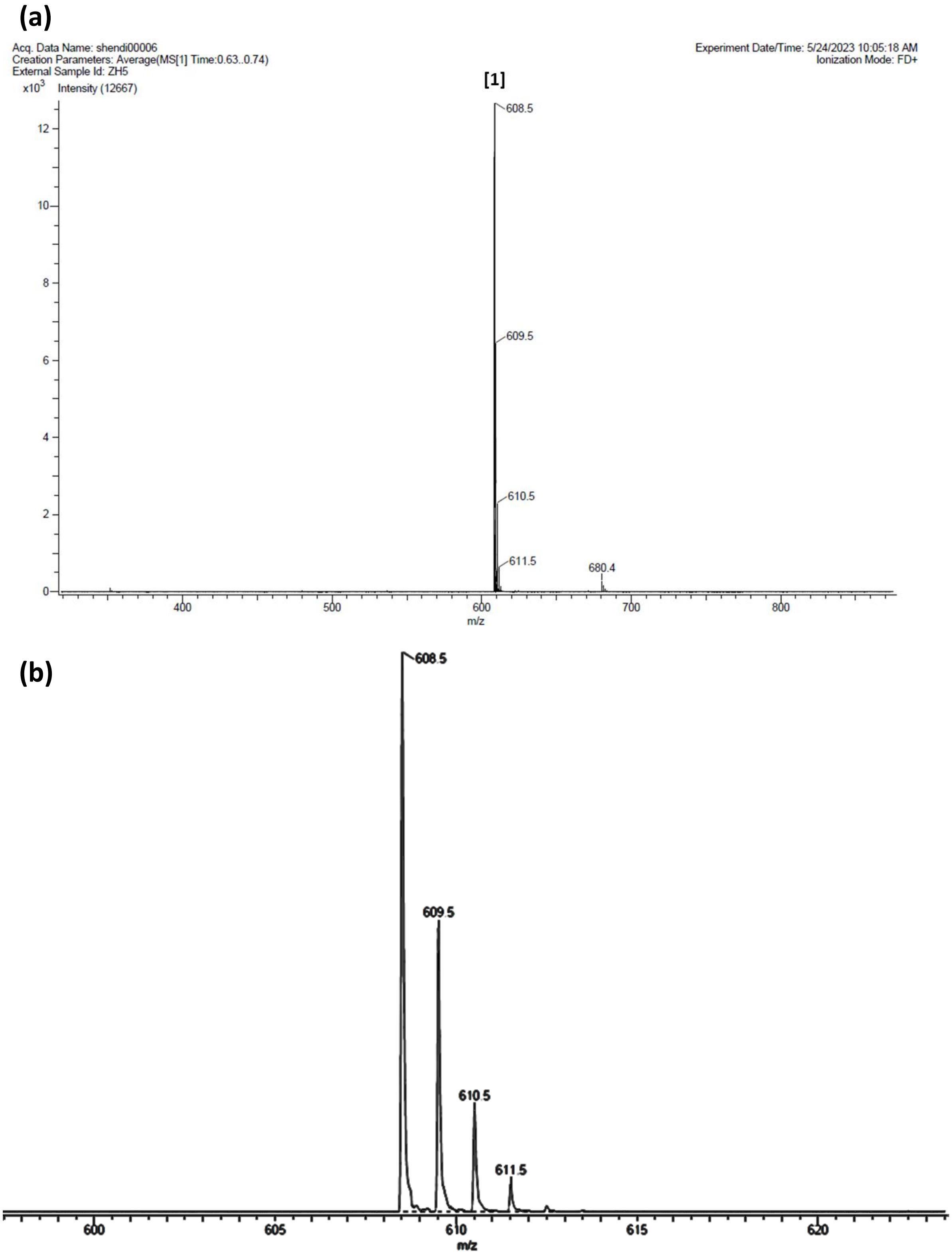

Figure S8: (a) LIFDI spectrum of (**1**), (b) expansion of the pattern.

(B) Synthesis of (Mes*P)$_2$(CCO)FeSi(PhC(N^{t}Bu)$_2$)TMS (2).

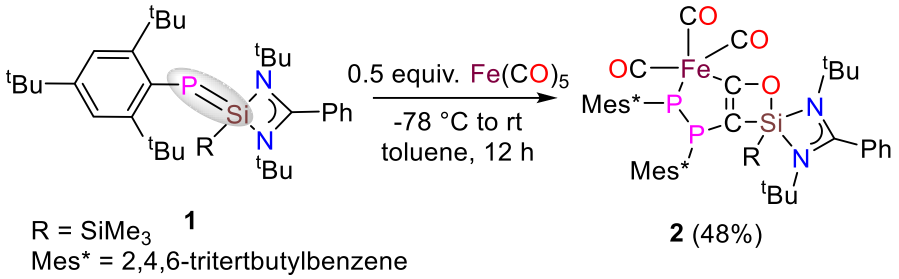

Fe(CO)$_5$ (45 µL, 0.82 mmol) was added dropwise to a solution of (**1**) (100 mg, 0.164 mmol) in toluene (30 mL) at 0 °C. The flask was sealed, and the reaction mixture was allowed to warm to room temperature and stired for 24 hours which resulted in the formation of a dark green solution. After that, toluene was slowly reduced under a vacuum to give the crude product. 2-3 mL of dried hexane was added to the crude product and stored for 3 weeks at room temperature to afford green crystals of the desired compound (**2**). The experimental and calculated IR spectra of **2** is demonstrated in fig S17. According to the calculated IR spectrum the IR values for Fe(CO)$_3$ units are in the range of 2055-2109 cm^{-1} and for C=C-O unit is about 1044 cm^{-1} which are in agreement with the observed values in the experimental IR spectrum.

Yield 38 % (34 mg). mp 240-243 °C.

^{1}H NMR (400 MHz, C$_6$D$_6$) δ = 7.74 (s, 1H,m-PMes*H), 7.63 (s, 1H, m-PMes*H), 7.53 (s, 1H, m-PMes*H), 7.48 (d, *J*=3.6, 1H, m-PMes*H), 7.25 (d, *J*=6.83, 1H, ArH), 7.12 (s, 1H, ArH), 6.95-6.86 (m, 3H, ArH), 1.72 (s, 9H, ^{t}Bu-PMes*), 1.68 (s, 9H, ^{t}Bu-PMes*), 1.59 (s, 9H, ^{t}Bu-PMes*), 1.55 (s, 9H, ^{t}Bu-PMes*), 1.47 (s, 9H, ^{t}Bu-PMes*), 1.27 (s, 9H,N-^{t}Bu), 1.19 (s, 9H, N-^{t}Bu), 0.76 (s, 9H, ^{t}Bu-PMes*), 0.55 (s, 9H, SiMe$_3$).

^{13}C NMR (101 MHz, C$_6$D$_6$) δ = 167.89 (s), 158.20 (d, *J*= 43.05), 154.21 (d, *J*= 10.79), 153.88 (s), 152.18 (s), 151.72 (s), 150.47 (s), 139.34 (d, *J*=25.34), 134.21 (s), 129.17 (d, *J*=6.29), 127.01 (s),

126.13 (s), 125.27 (s), 123.41 (d, *J*=12.42), 122.10 (d, *J*= 6.13), 40.91(s), 40.47 (s), 39.13 (d, *J*= 6.92), 38.83 (s), 35.14 (d, *J*=6.92), 34.80 (s), 34.52 (s), 34.35 (s), 34.22 (s), 33.39 (s), 32.63 (s), 31.71 (s), 31.57 (s), 30.84 (d, *J*=7.94), 0.67 (s).

$^{31}P\{^1H\}$ NMR (400 MHz, C_6D_6) δ = 387.66 (d, $^1J_{PP}$= 459.60), 54.50 (d, $^1J_{PP}$= 459.60)

$^{29}Si\{^1H\}$ NMR (500 MHz, C_6D_6) δ = -14.33 (s, $SiMe_3$), -86.43 (s, Si [(PhC(NtBu)$_2$SiOCC])

MS (LIFDI, toluene): m/z for $C_{59}H_{90}FeN_2O_4P_2Si_2$ $(M)^+$: 1064.3

EA experimental (calculated): C 66.14 (66.52), H 9.03 (8.52), N 2.31 (2.63) %.

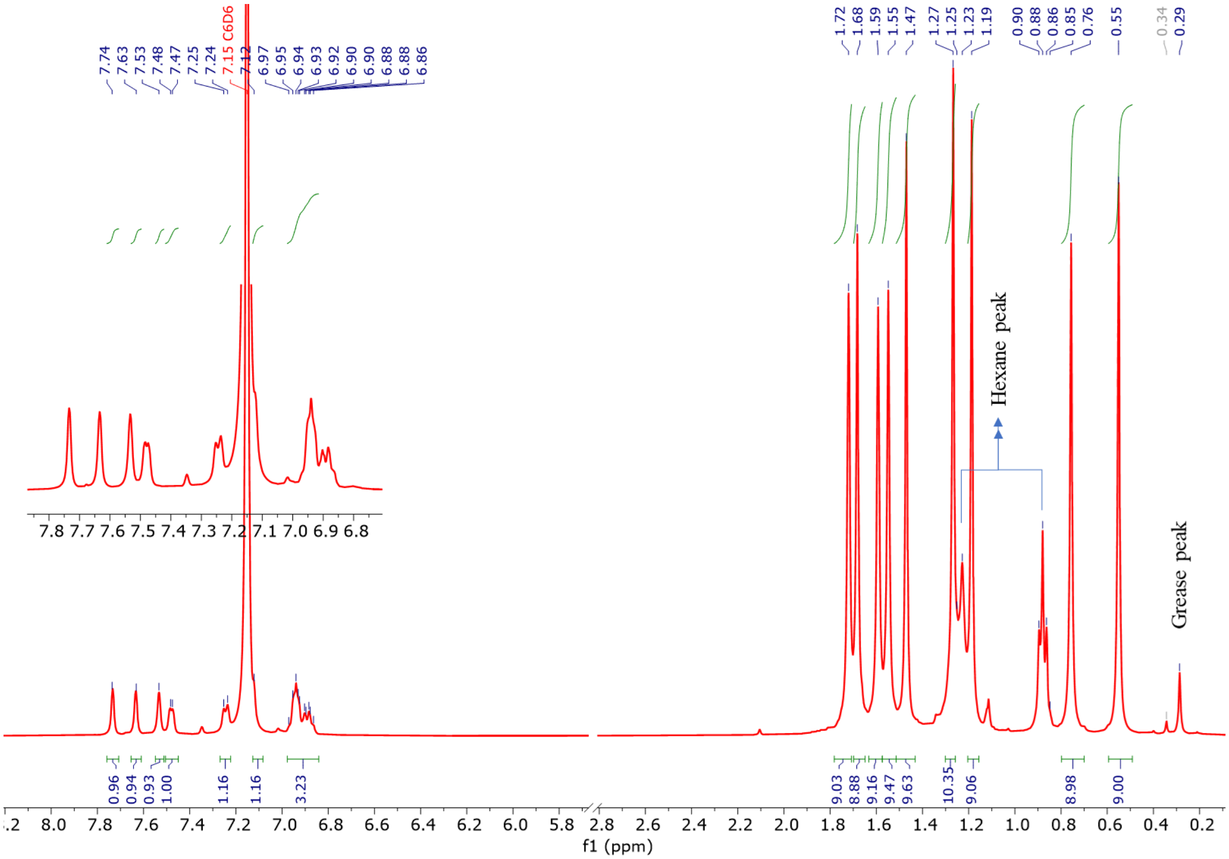

Figure S9. ^1H NMR spectrum of **2** in C_6D_6.

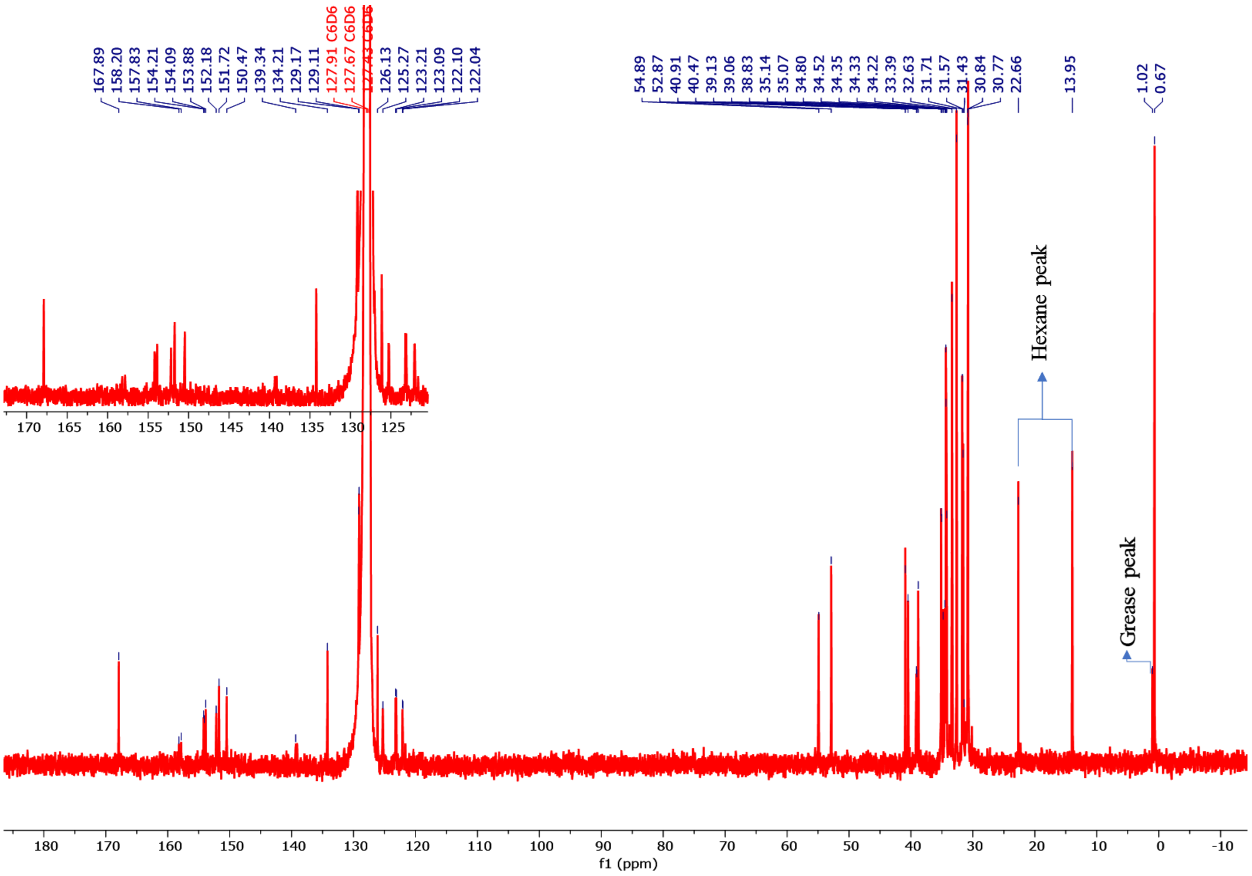

Figure S10. ^{13}C NMR spectrum of **2** in C_6D_6

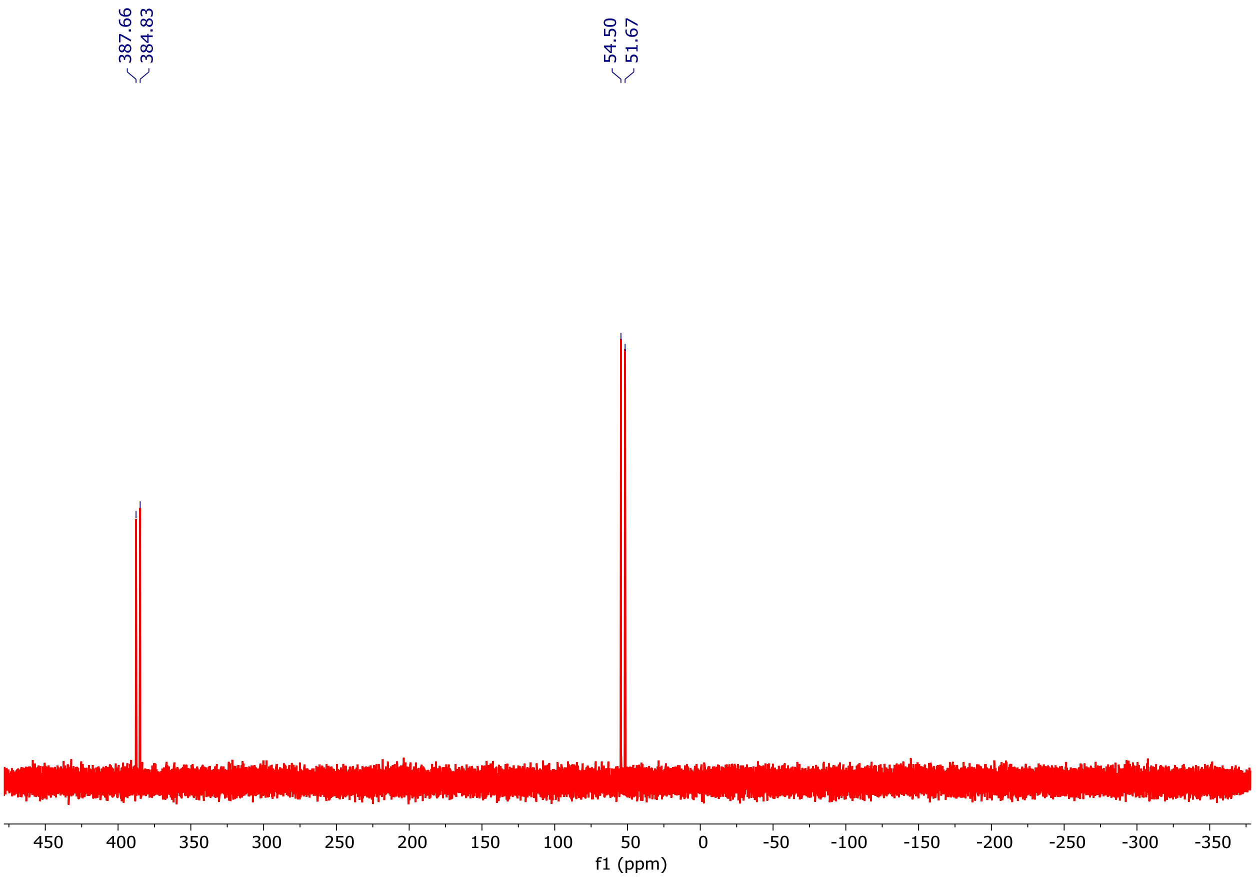

Figure S11. $^{31}P\{^{1}H\}$ NMR spectrum of **2** in C_6D_6.

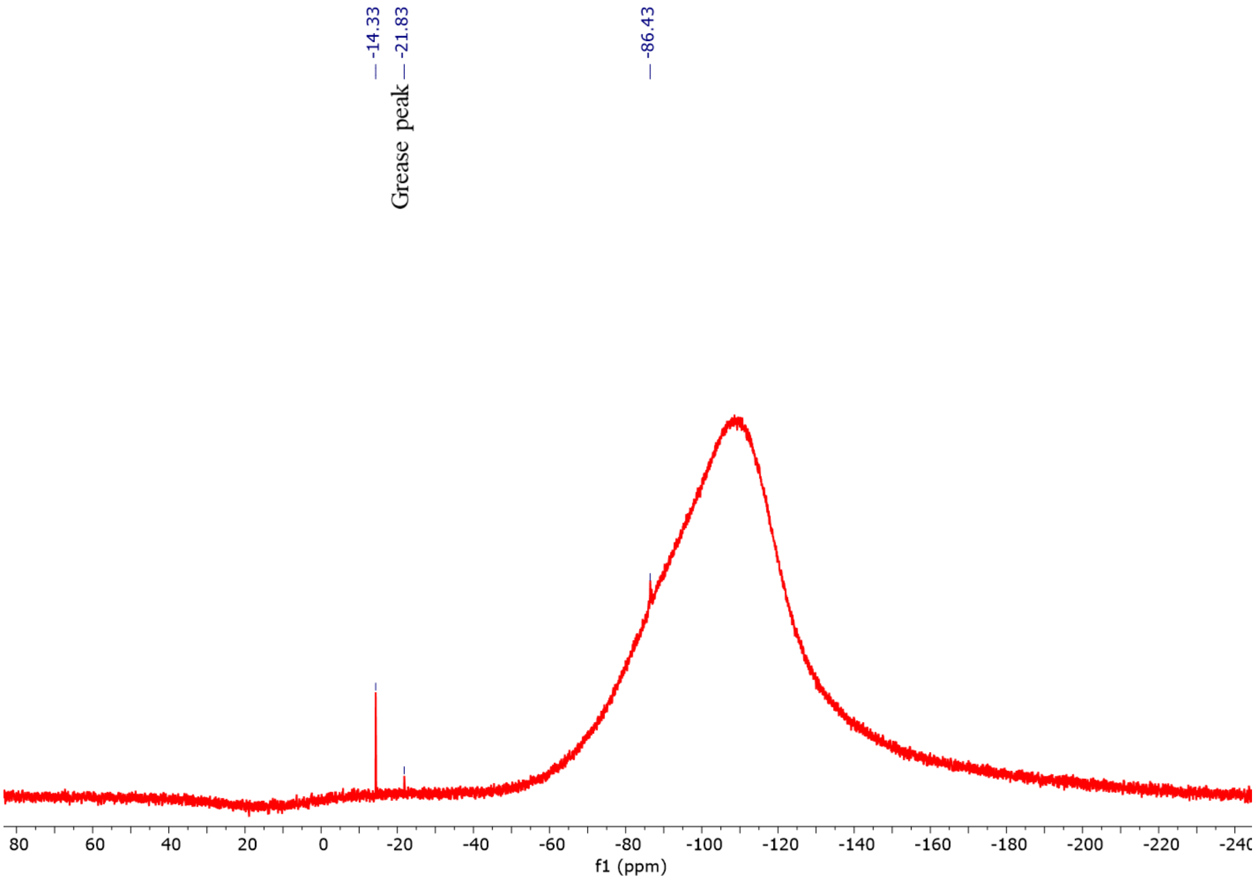

Figure S12. $^{29}Si\{^1H\}$ NMR spectrum of **2** in C_6D_6.

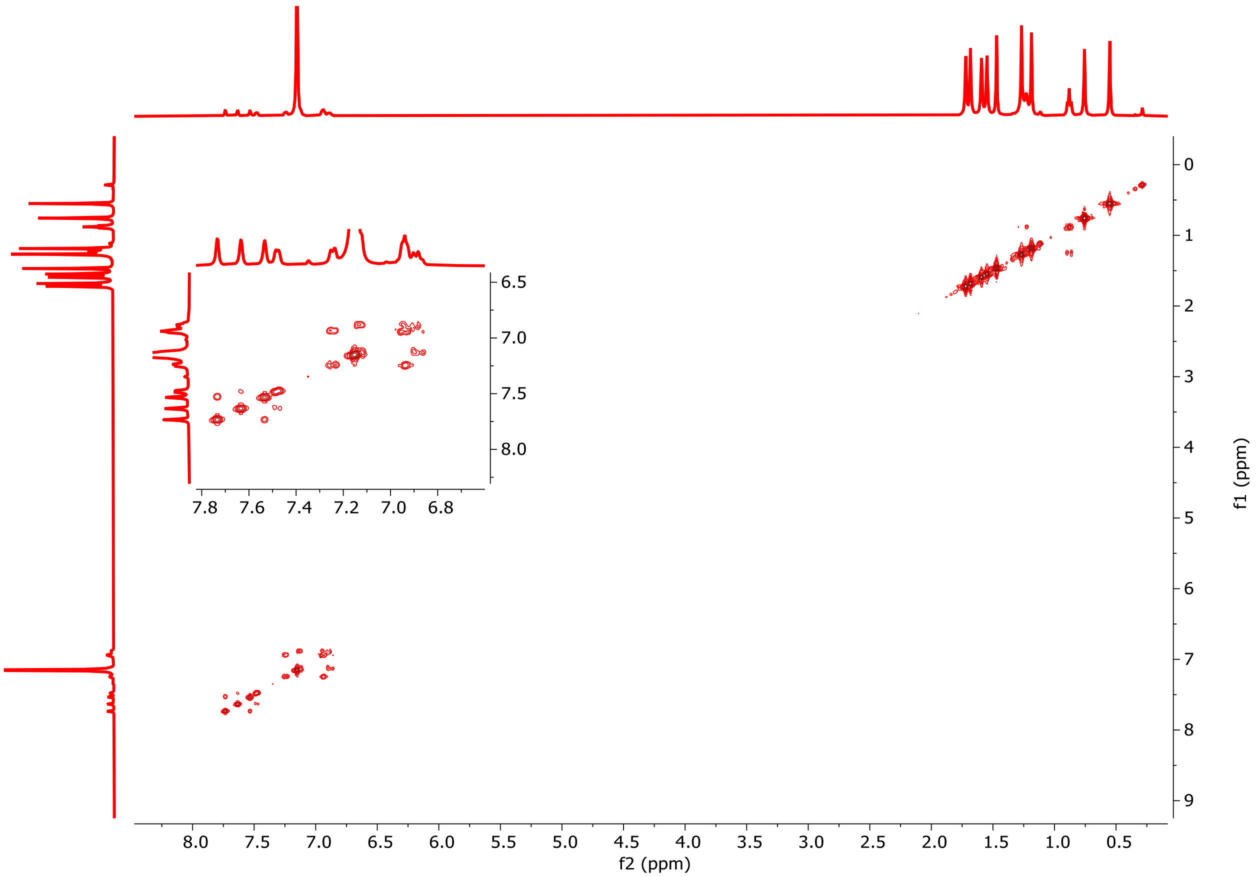

Figure S13. 2D ^{1}H-^{1}H COSY NMR spectrum of **2** in C_6D_6.

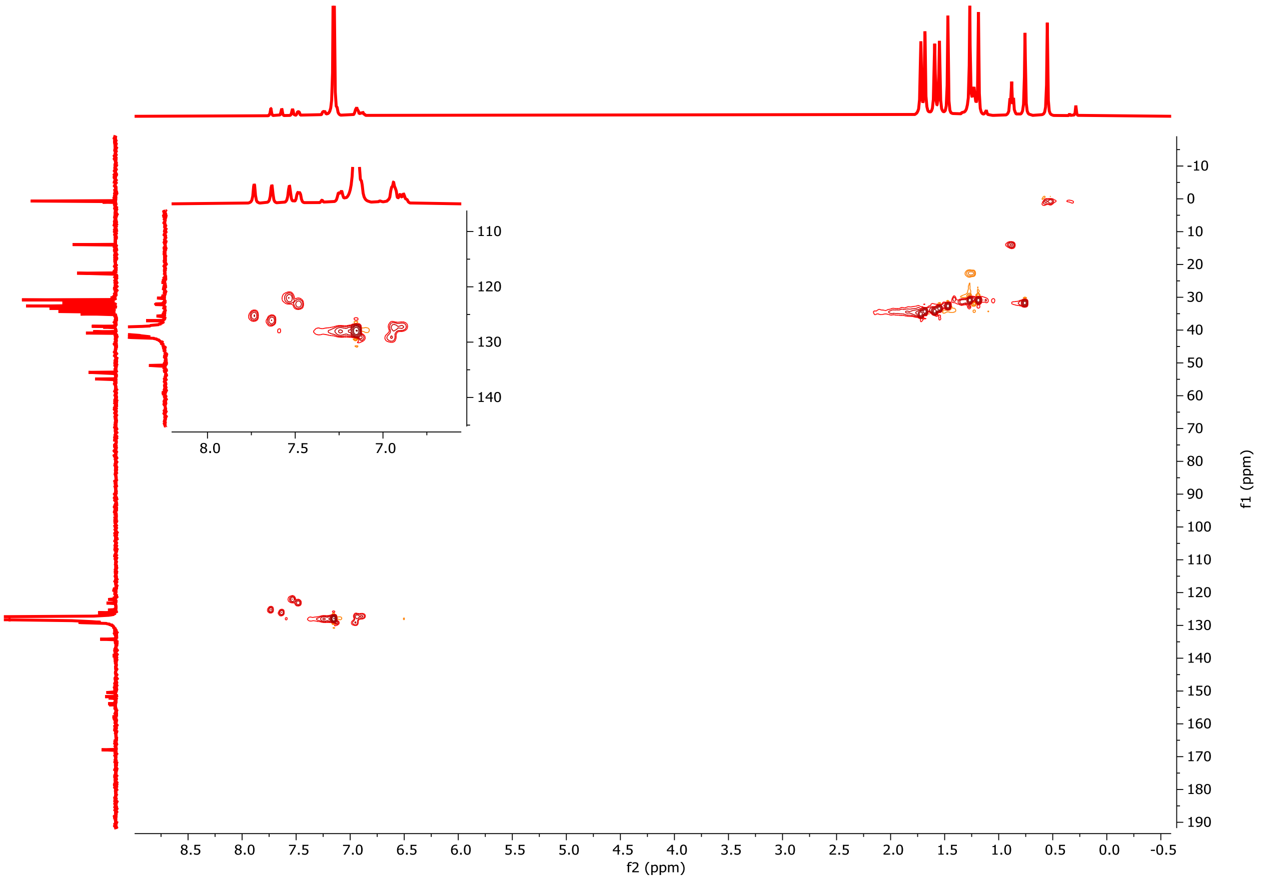

Figure S14. 2D ^{1}H-^{13}C HSQC NMR spectrum of **2** in C_6D_6.

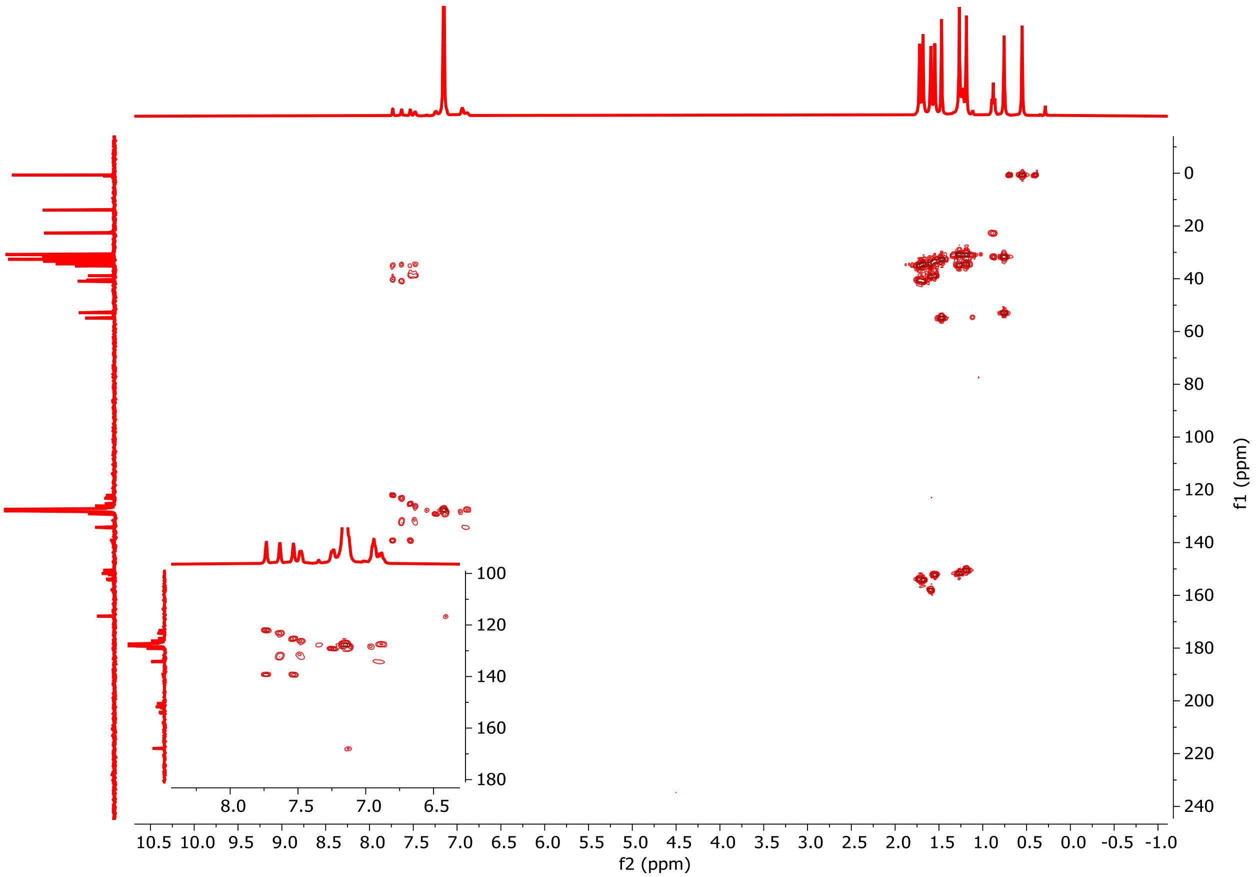

Figure S15. 2D ^1H-^{13}C HMBC NMR spectrum of **2** in C_6D_6.

(a)

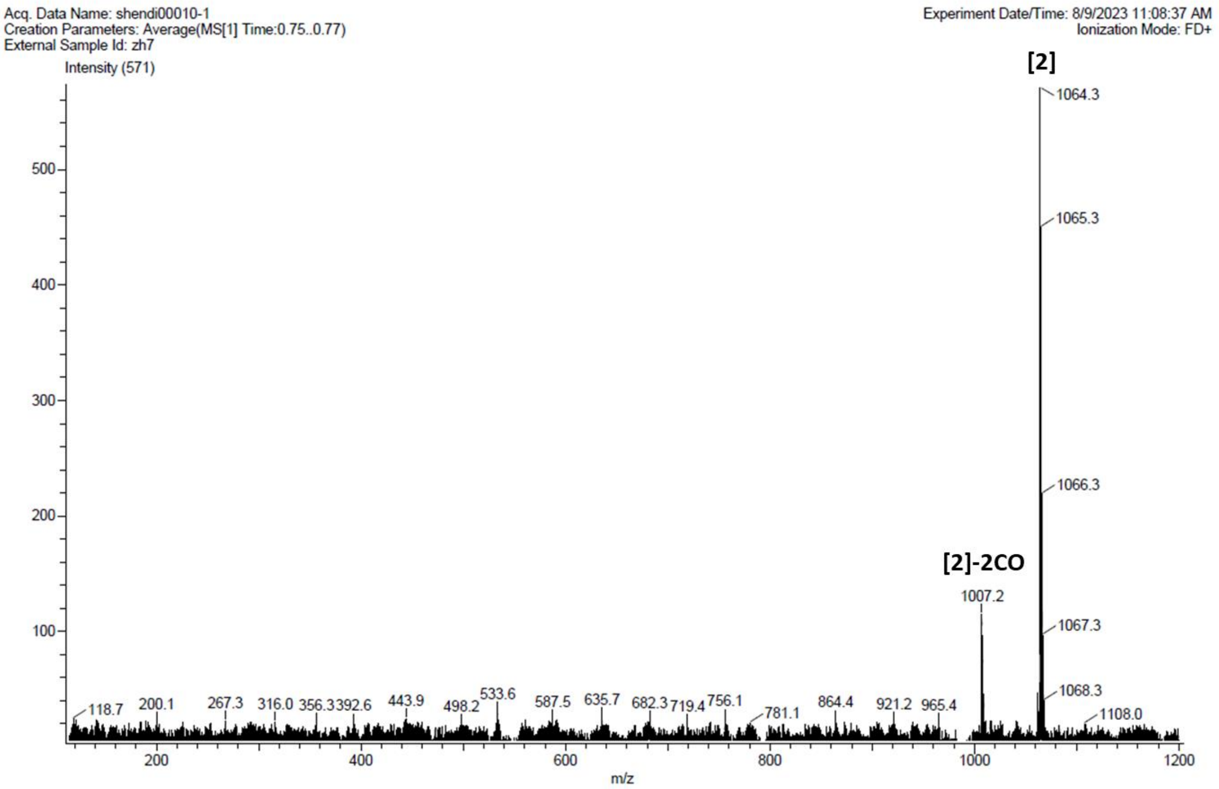

(b)

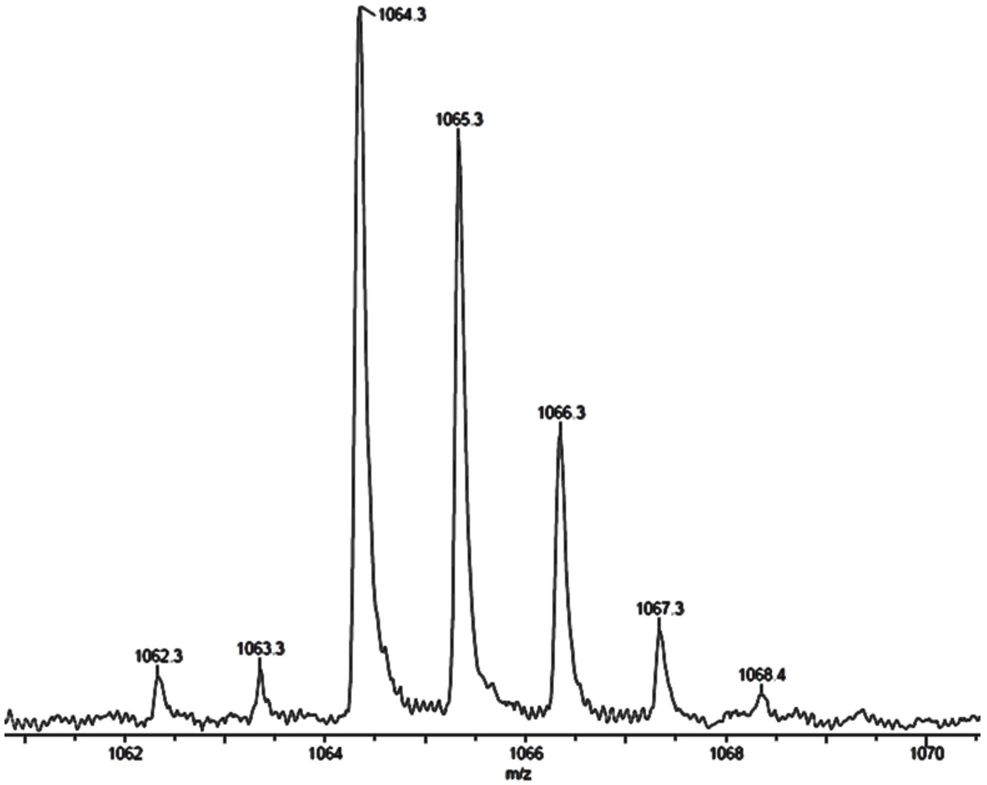

(Figure S16: (a) LIFDI spectrum of **(2)**, (b) expansion of the pattern

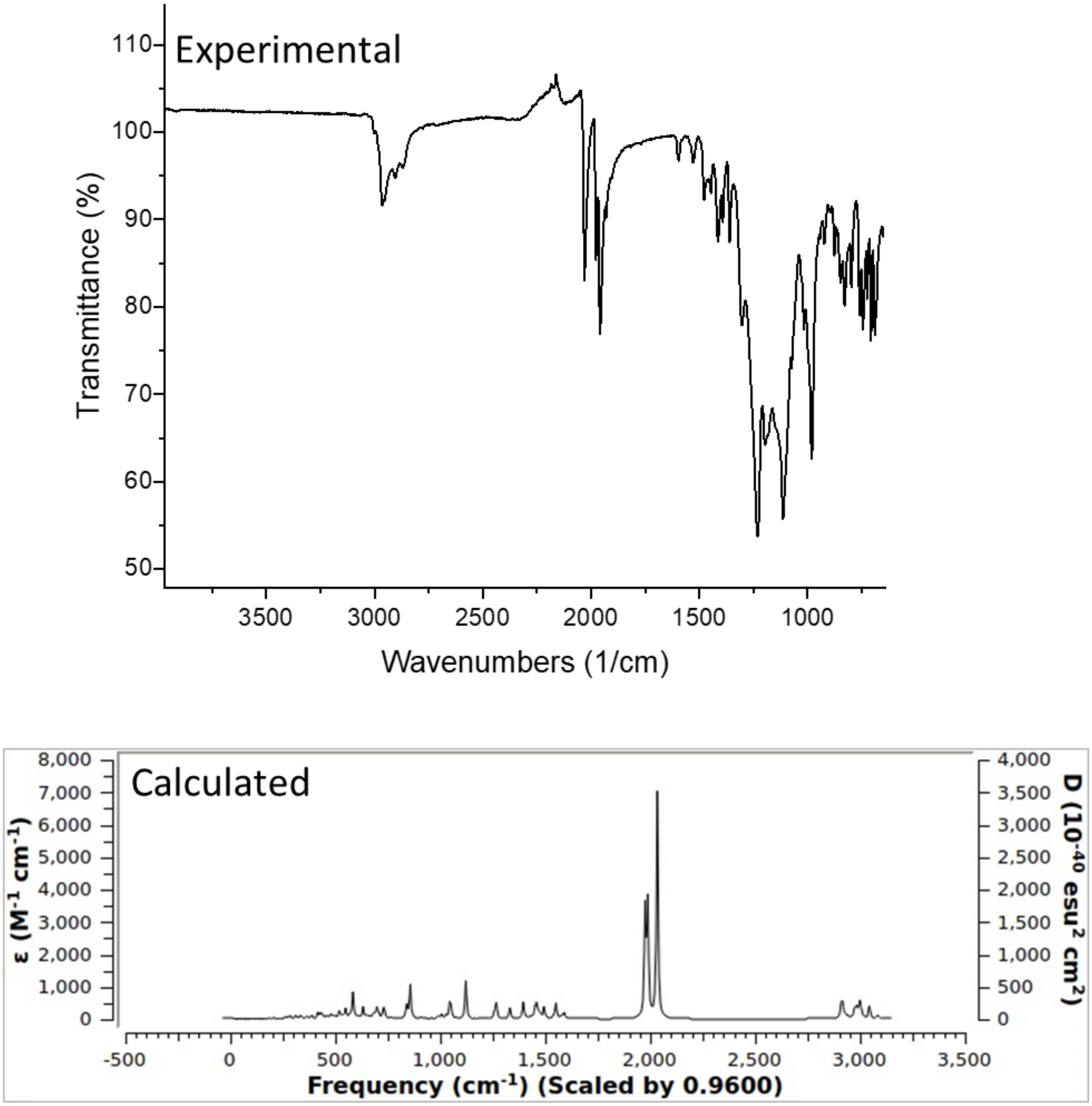

Figure S17. Experimental and calculated IR spectra of comound **2.**

(C) Synthesis of $(Mes^*P)_2$ (C) Si(PhC(N^{t}Bu)$_2$)(COTMS)Mo (3).

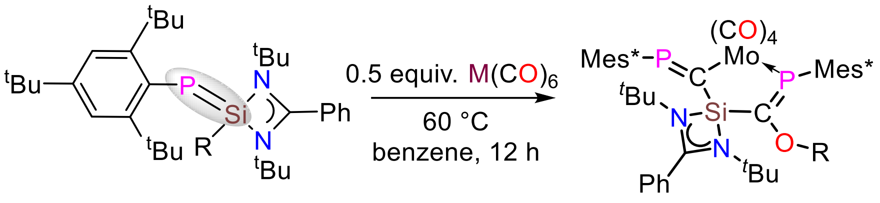

1 **3**

R = $SiMe_3$
Mes* = 2,4,6-tritertbutylbenzene

$Mo(CO)_6$ (26mg, 0.099 mmol) was added to a solution of (**1**) (120 mg, 0.197 mmol) in dry benzene (5 mL) at room temperature. The flask was sealed, and the reaction mixture was allowed to stire at 60°C for 12 hours which resulted in the formation of a dark red solution. After cooling to the room temperature red crystals of **3** were formed. The solution was separated from the crystals and the crystals were washed 3 times with dry benzene. The experimental and calculated IR spectra of **2** is demonstrated in fig S23. According to the calculated IR spectrum the IR values for $Mo(CO)_4$ units are in the range of 1950-2070 cm^{-1} , for C-O-$SiMe_3$ unit is about 1320-1330 cm^{-1} , P-C-O-SiMe3 1285 cm^{-1}, and for P-C-Si[(PhC(NtBu)$_2$] is around 1034 cm^{-1} which are in agreement with the observed values in the experimental IR spectrum.

Yield 55 % (123 mg). mp 232-235 °C.

^{1}H NMR (400 MHz, Tol(d_8)) δ = 7.72 (s, 2H, PMes*H), 7.64 (d, J=1.74, 2H, m-PMes*H), 7.55 (d, J=7.62, 1H, ArH), 7.47-7.44 (m, 1H, ArH), 6.98-6.66 (m, 2H, ArH), 6.80-6.76 (m, 1H, ArH), 1.96 (s, 18H, ^{t}Bu-PMes*), 1.89 (s, 18H, ^{t}Bu-PMes*), 1.52 (s, 9H, ^{t}Bu-PMes*), 1.36 (s, 9H, ^{t}Bu-PMes*), 1.25 (s, 18H, N-^{t}Bu), 0.02 (s, 9H, $SiMe_3$).

^{13}C NMR (400 MHz, Tol(d_8)) δ = 219.88 (s), 213.13 (s), 178.47 (s), 156.24 (s), 153.07 (s), 151.07 (s), 149.96 (s), 131.03 (s), 130.84 (s), 130.58 (d, J=25.56), 130.20 (s), 128.37 (s), 127.43 (s),

127.10 (s), 125.58 (d, *J*= 5.00), 124.28 (s), 123.15 (d, *J*= 4.25), 122.35 (s), 54.50(s), 39.60 (s), 39.33 (s), 35.22 (d, *J*=5.36), 34.98 (s), 34.17 (s), 32.00 (s), 31.58 (s), 31.34 (s), 31.22 (s), 30.28 (s), 23.06 (s), 14.29 (s), 2.88 (s).

$^{31}P\{^1H\}$ NMR (400 MHz, Tol(d_8)) δ = 483.41(d, $^3J_{PP}$= 8.78), 249.70 (s)

$^{29}Si\{^1H\}$ NMR (500 MHz, Tol(d_8)) δ = -16.41 (m, Si [(PhC(NtBu)$_2$]), 14.30 (s, $SiMe_3$)

MS (LIFDI, THF): m/z for $C_{59}H_{90}N_2O_4P_2Si_2Mo$ {[(M)$^+$]-CO}: 1106.3

EA experimental (calculated): C 63.08 (63.58), H 8.29 (8.00), N 2.52 (2.47) %.

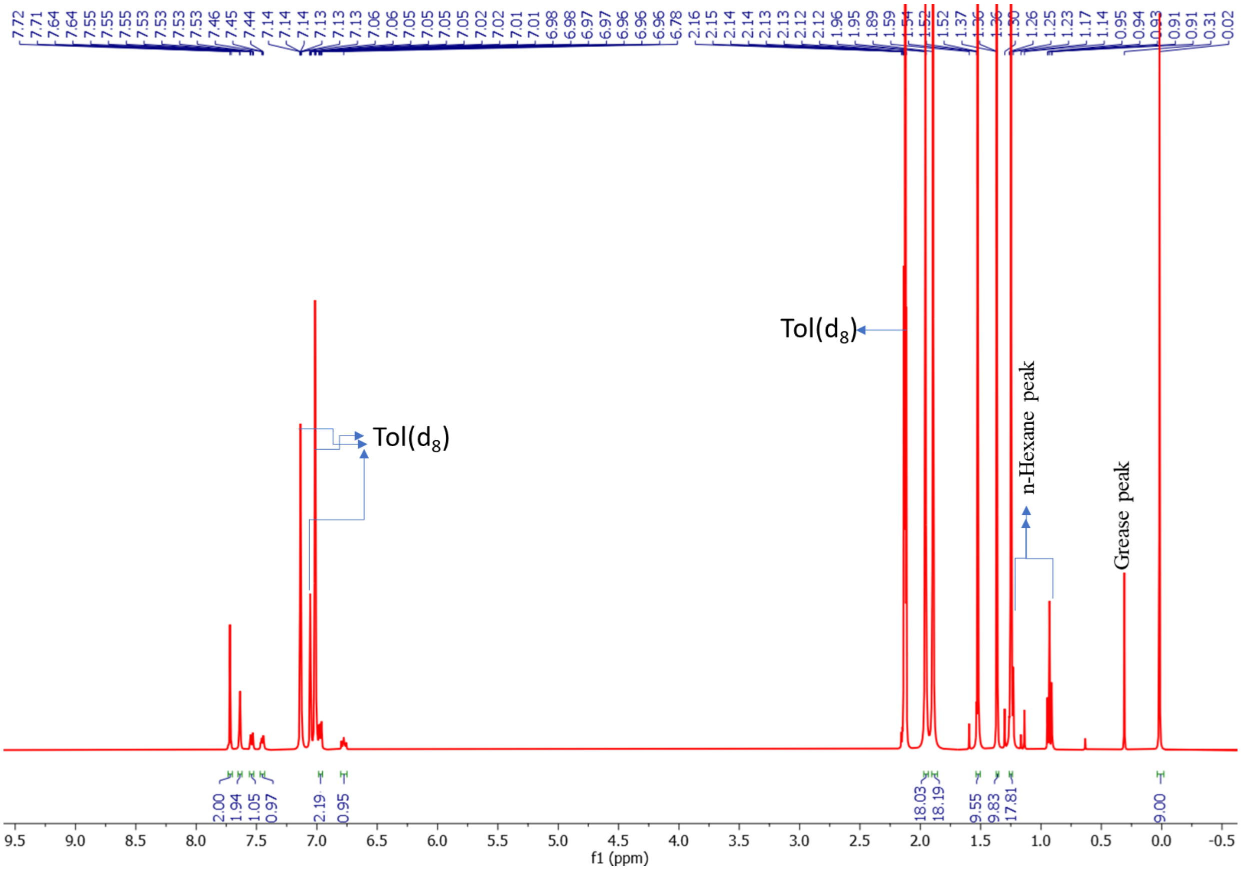

Figure S18. ^{1}H NMR spectrum of compound **(3)** in Toluene (d_8).

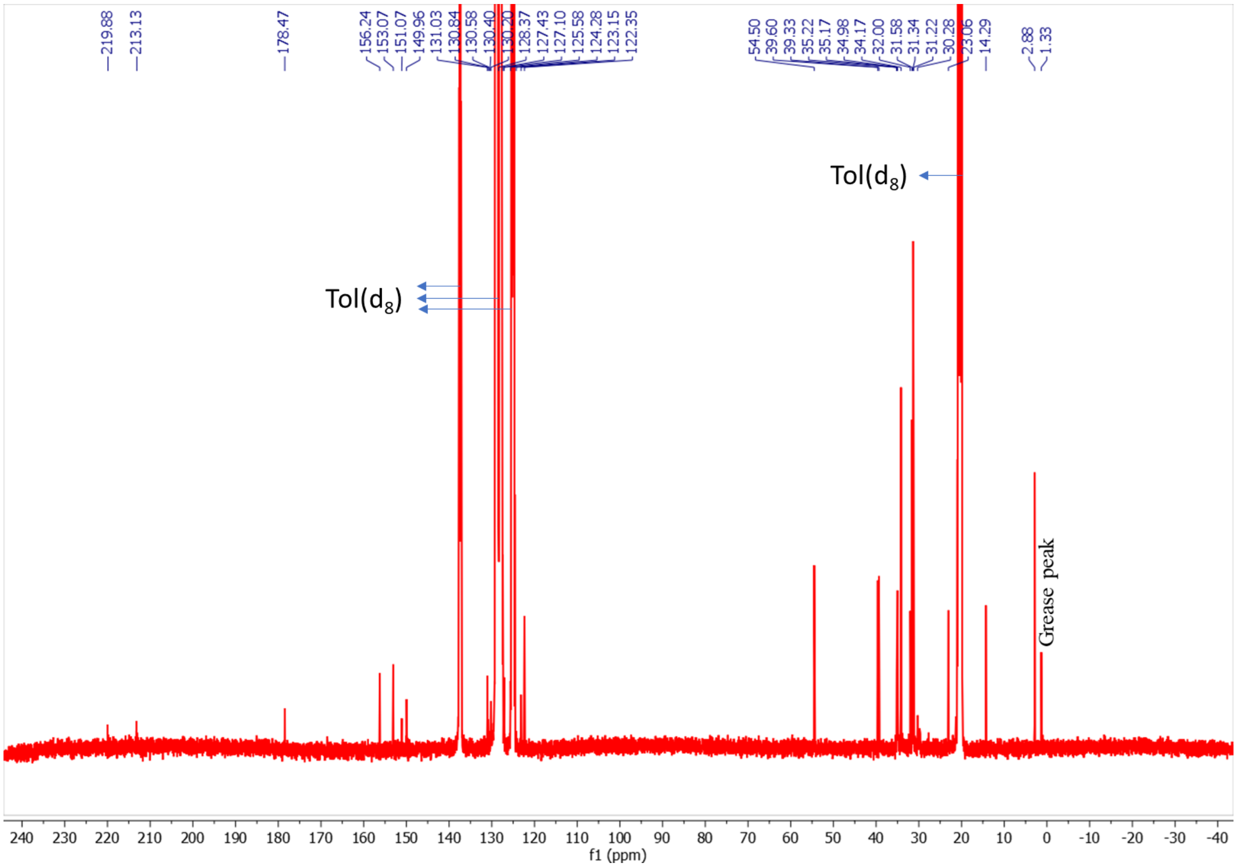

Figure S19. ^{13}C NMR spectrum of compound **(3)** in Toluene (d_8).

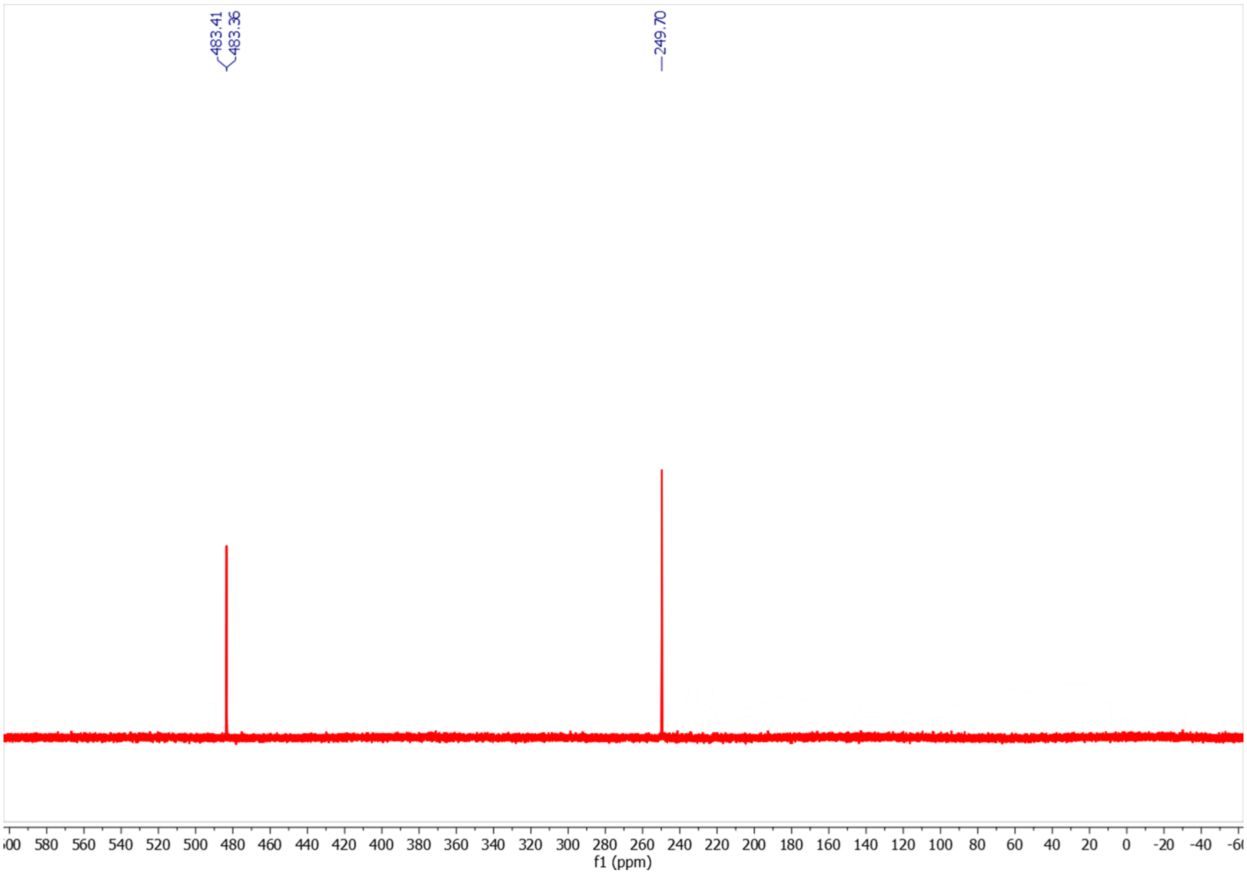

Figure S20. ^{31}P NMR spectrum of compound **(3)** in Toluene (d_8).

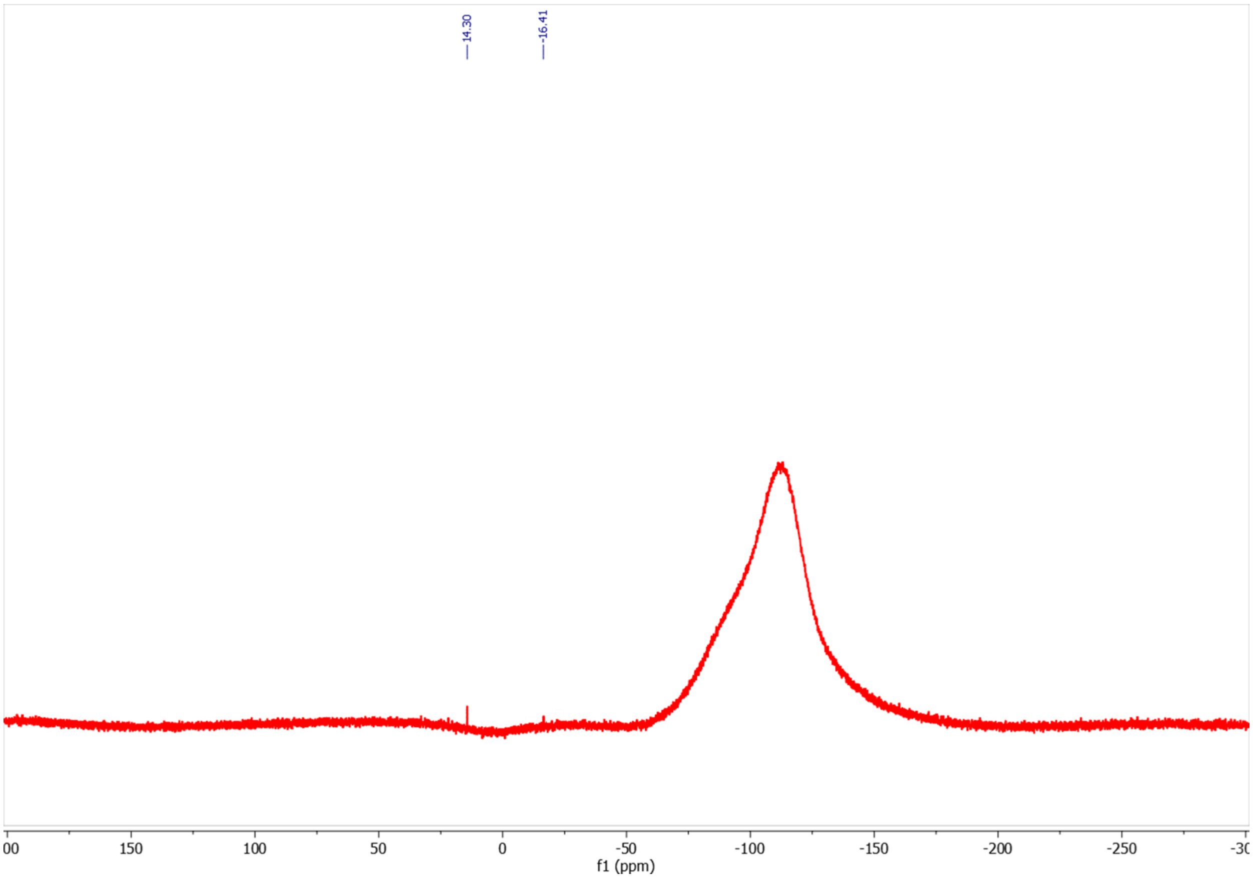

Figure S21. ^{29}Si NMR spectrum of compound **(3)** in Toluene (d_8).

(a)

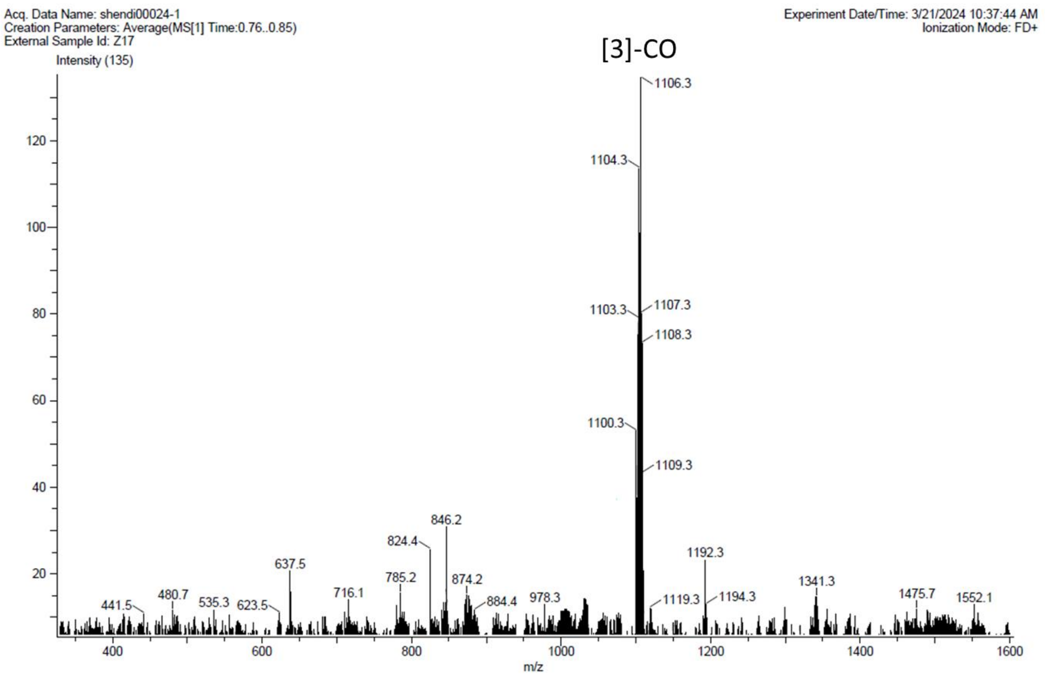

(b)

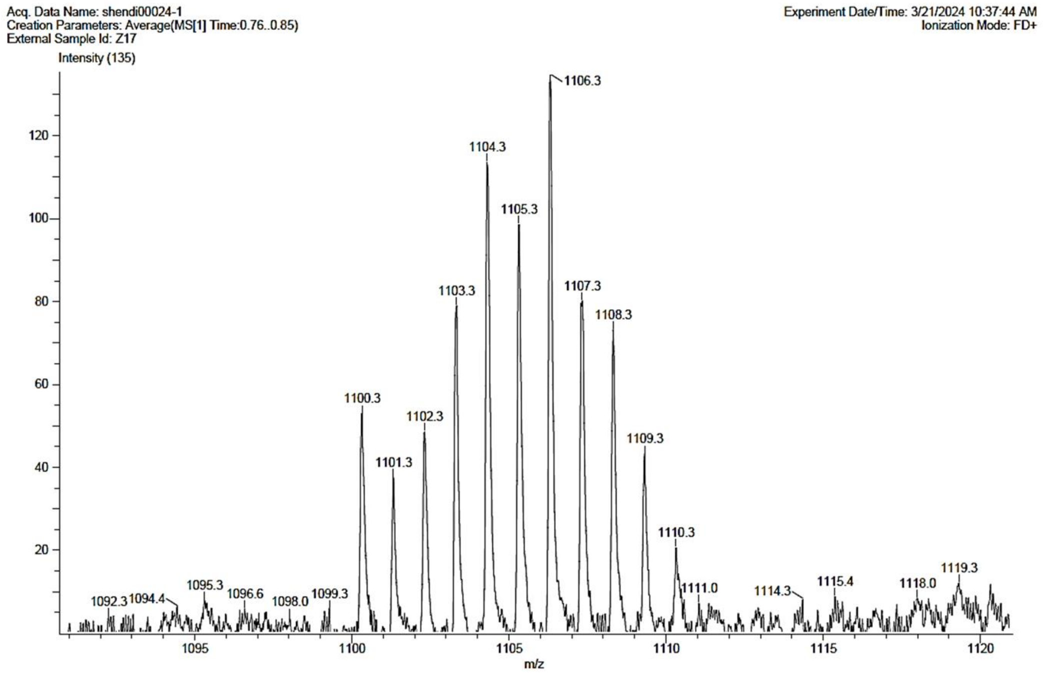

Figure S22: (a) LIFDI spectrum of compound **(3)**, (b) expansion of the pattern.

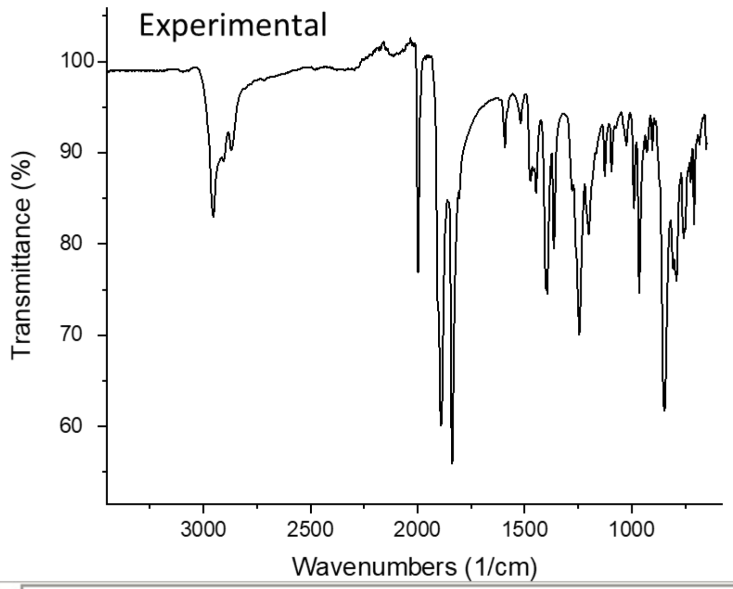

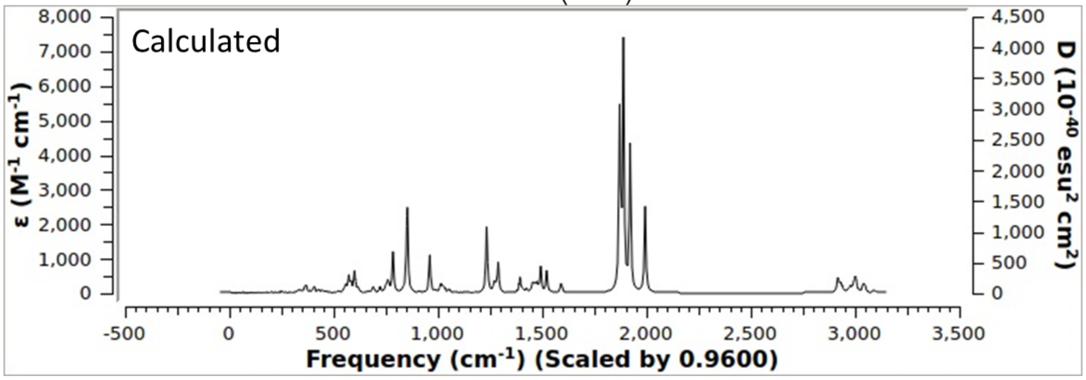

Figure S23: Experimental and calculated IR spectrum of the compound (**3**) (cm^{-1}).

(D) Synthesis of (Mes*P)$_2$ (C) Si(PhC(N^{t}Bu)$_2$)(CO(TMS))W (4).

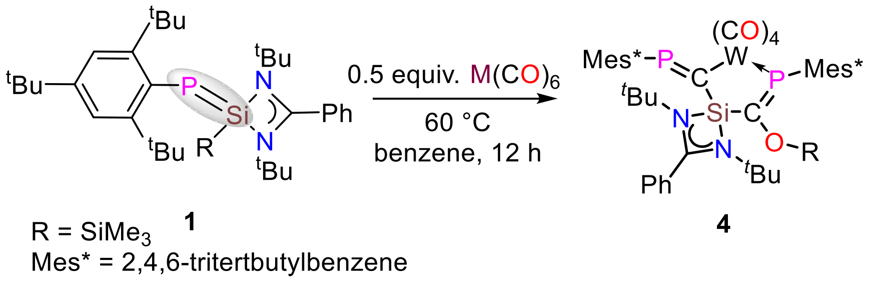

$W(CO)_6$ (35 mg, 0.099 mmol) was added to a solution of (**1**) (120 mg, 0.197 mmol) in dry benzene (5 mL) at room temperature. The flask was sealed, and the reaction mixture was allowed to stire at 60°C for 12 hours which resulted in the formation of a dark red solution. After cooling to the room temperature red crystals of **4** were formed. The solution was separated from the crystals and the crystals were washed three times with dry benzene. The calculated IR spectrum of 4 is in good agreement with the experimental one Fig S32.

Yield 62 % (150 mg). mp 248-252 °C.

^{1}H NMR (400 MHz, THF(d_8)) δ = 7.68-7.63 (m, 7H, ArH), 7.41 (d, 2H, m-PMes*H), 1.76 (s, 18H, m-^{t}Bu-PMes*), 1.69 (s, 18H, m-^{t}Bu -PMes*), 1.40 (s, 9H, p-^{t}Bu-PMes*), 1.38 (s, 9H, p-^{t}Bu-PMes*), 1.31 (s, 18H, N-^{t}Bu), 0.76 (s, 9H, ^{t}Bu-PMes*), 0.08 (s, 9H, $SiMe_3$).

^{13}C NMR (101 MHz, C_6D_6) δ = 209.70 (d, *J*=5.26), 207.41 (d, *J*=10.79), 179.22 (s), 155.58 (s), 152.42 (s), 151.21 (s), 148.96 (s), 131.44 (s), 130.14 (s), 129.58 (s), 128.64 (s), 127.77 (s), 127.53 (s), 127.34 (d, *J*=5.45) 123.10 (d, *J*=5.45), 121.52 (s), 54.40 (s), 39.14 (s), 38.59 (s), 34.88 (s), 34.54 (s), 34.18 (d, *J*= 6.16), 33.66(s), 33.40 (s), 31.56 (s), 30.81 (s), 30.77 (s), 30.66 (s), 30.48 (s), 22.56 (s), 13.47 (s), 2.17 (s).

^{31}P{^{1}H} NMR (400 MHz, C_6D_6) δ = 466.95 (d, $^{3}J_{PP}$= 7.8), 216.75 (d, $^{3}J_{PP}$= 7.8).

^{29}Si{^{1}H} NMR (500 MHz, C_6D_6) δ = 16.28 (s, $SiMe_3$), -10.82 to -12.62 (dd, $^{2}J_{SiP}$= 114.44, $^{2}J_{SiP}$= 62.56, Si [(PhC(NtBu)$_2$SiOCC]).

MS (LIFDI, THF): m/z for $C_{59}H_{90}N_2O_4P_2Si_2W$ {[(M)$^+$]-CO}: 1192.3

EA experimental (calculated): C 58.73 (59.01), H 7.02 (7.43), N 2.43 (2.29) %.

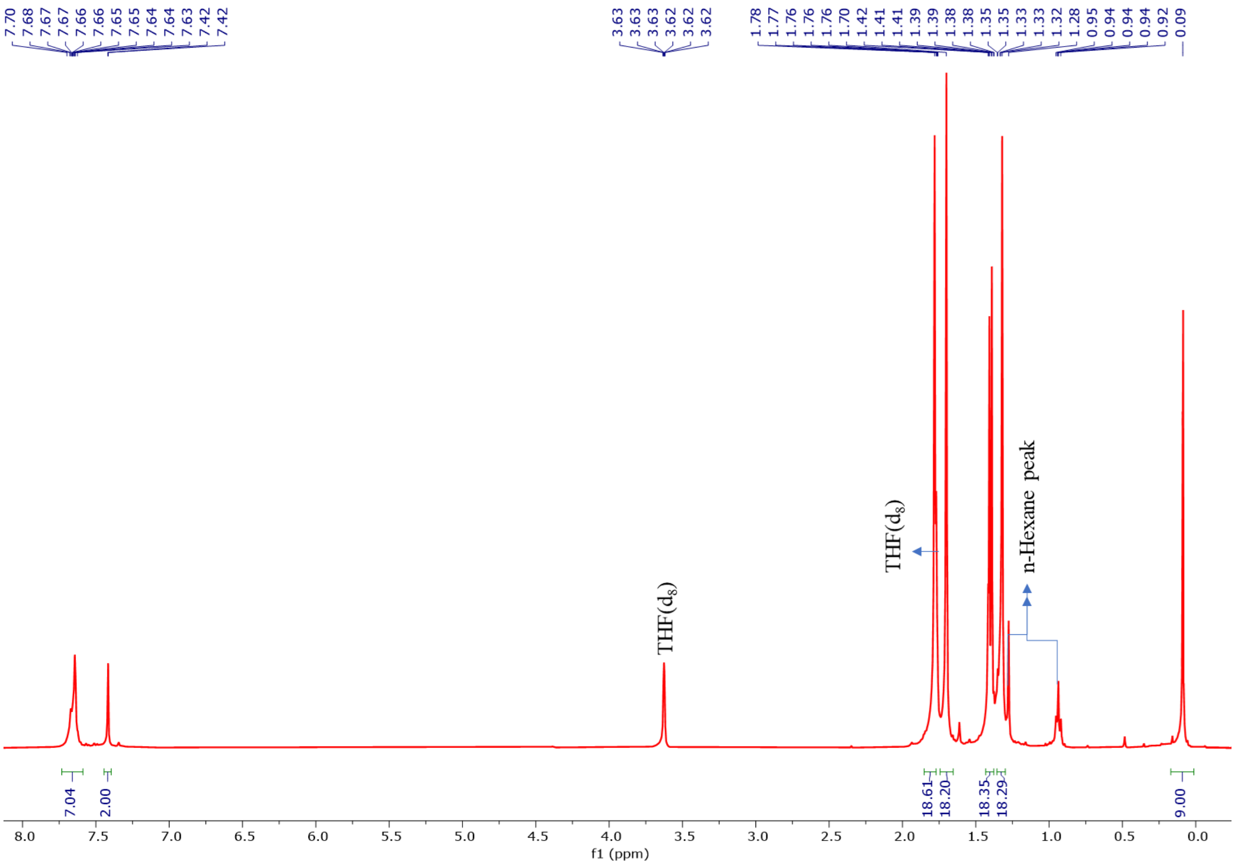

Figure S24. ^{1}H NMR spectrum of compound **(4)** in THF (d_8).

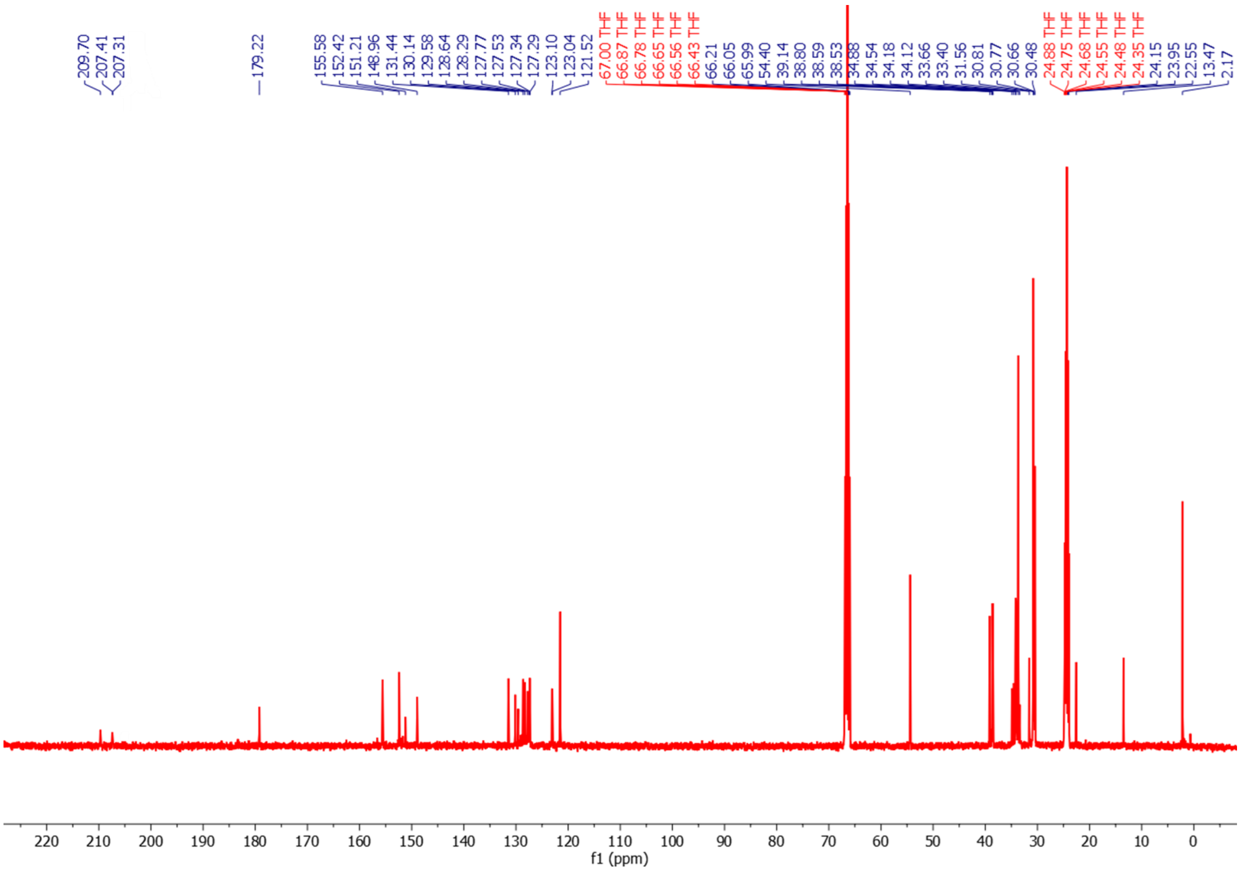

Figure S25. ^{13}C NMR spectrum of compound **(4)** in THF (d_8).

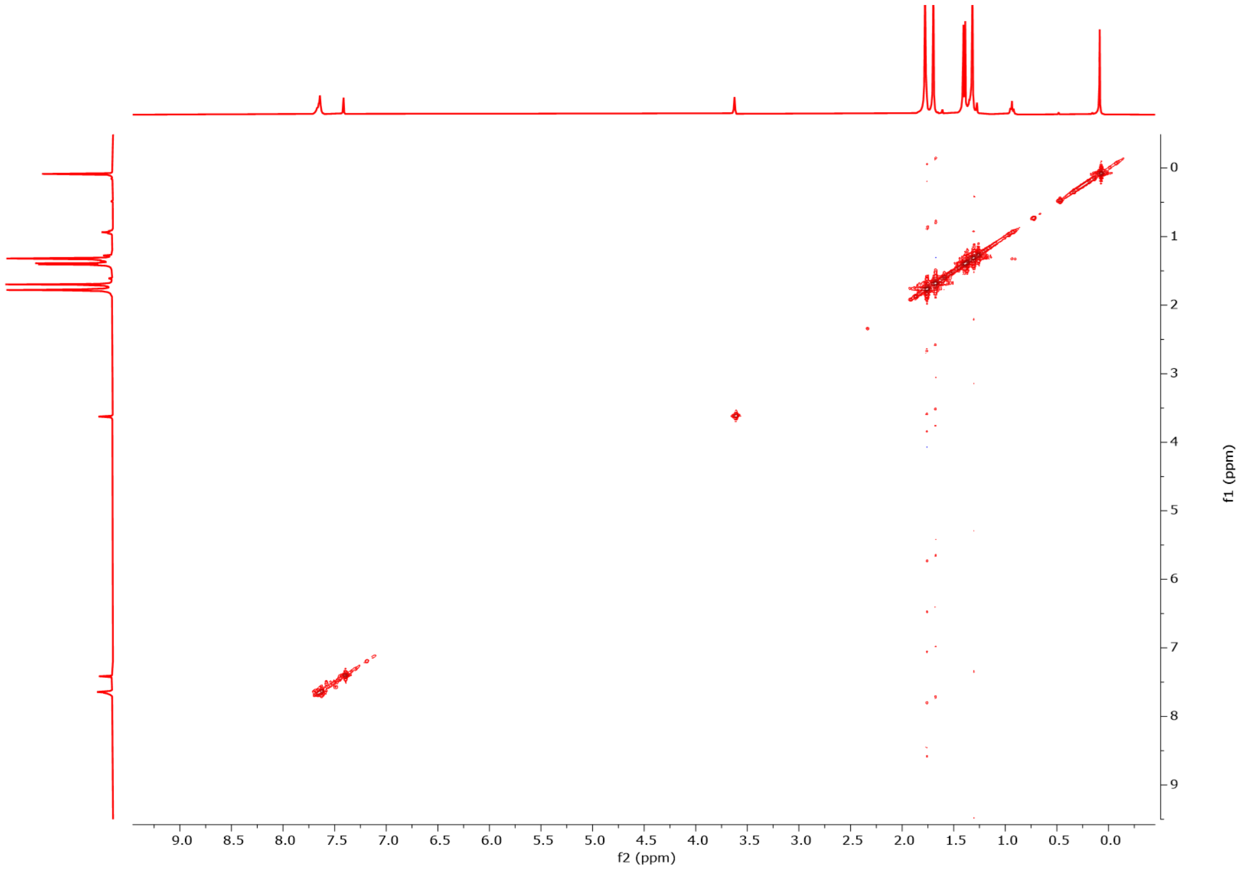

Figure S26. 2D ^{1}H - ^{1}H COSY NMR spectrum of compound **(4)** in THF (d_8).

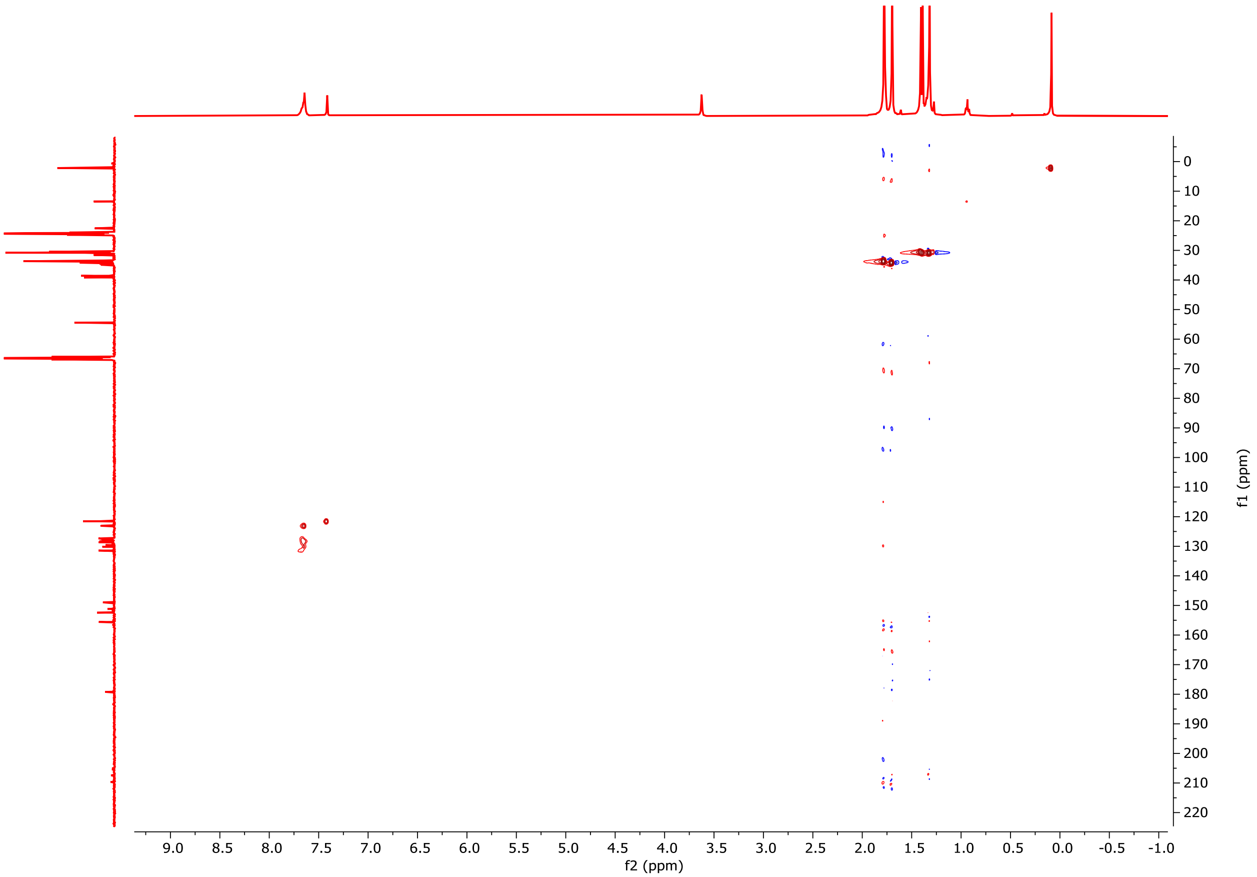

Figure S27. 2D ^{1}H - ^{13}C HSQC NMR spectrum of compound **(4)** in THF (d_8).

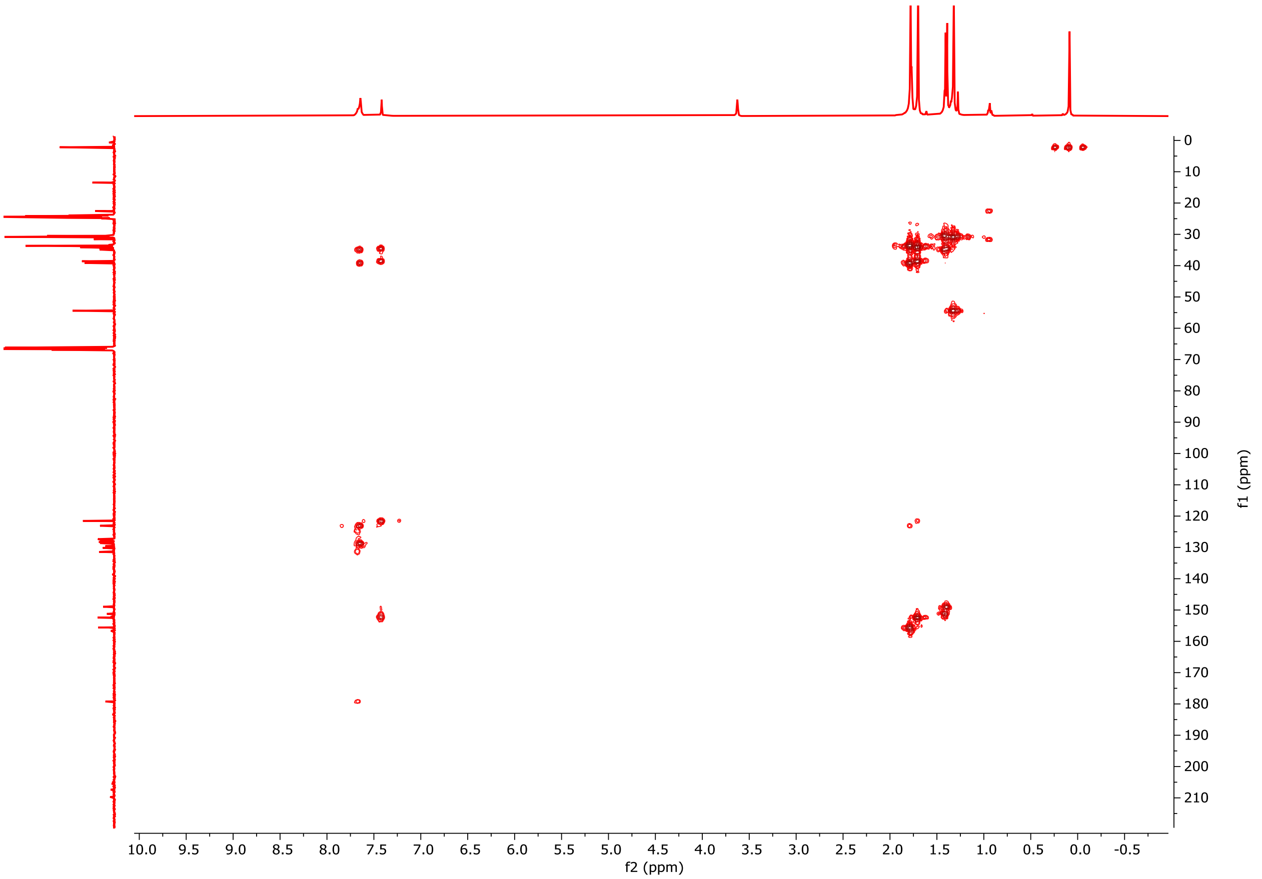

Figure S28. 2D ^{1}H - ^{13}C HMBC NMR spectrum of compound **(4)** in THF (d_8).

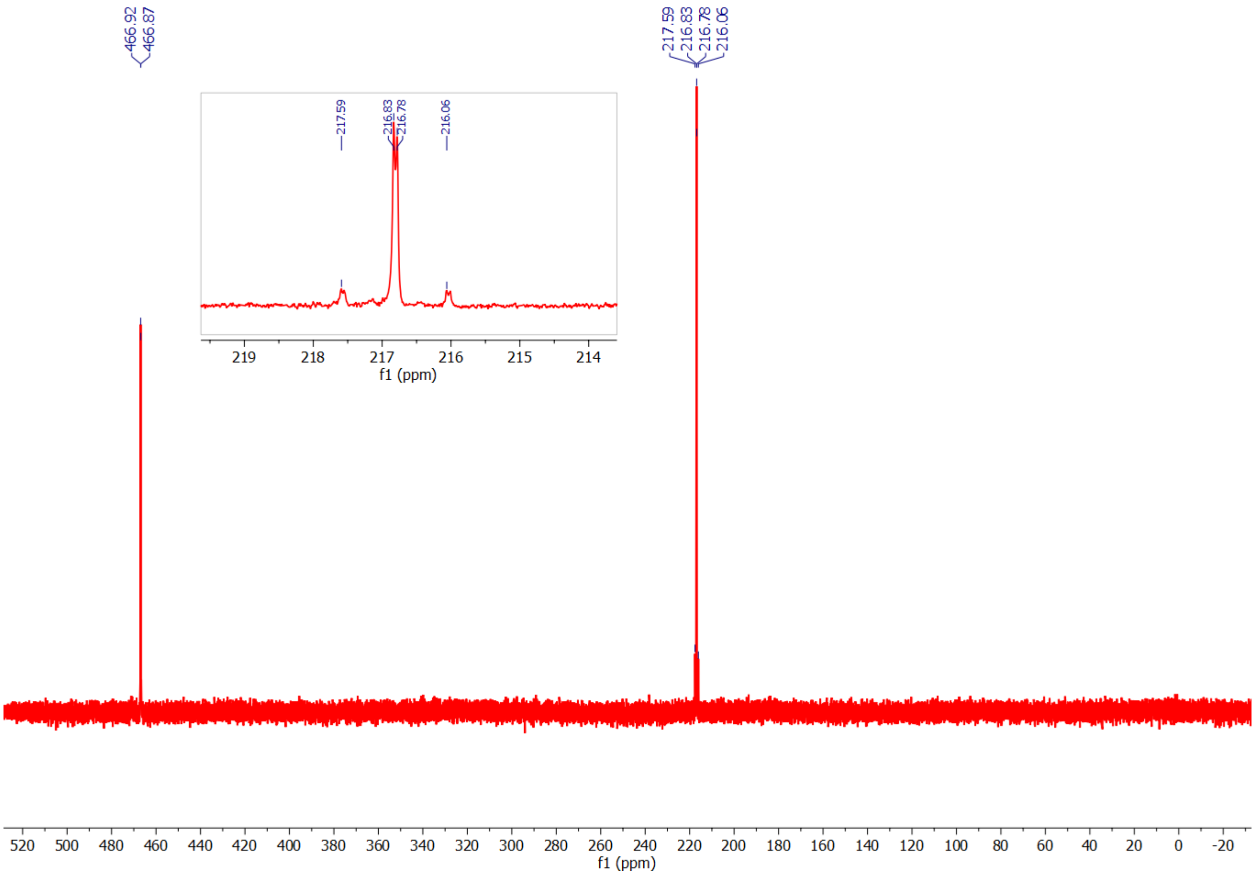

Figure S29. ^{31}P NMR spectrum of compound **(4)** in THF (d_8).

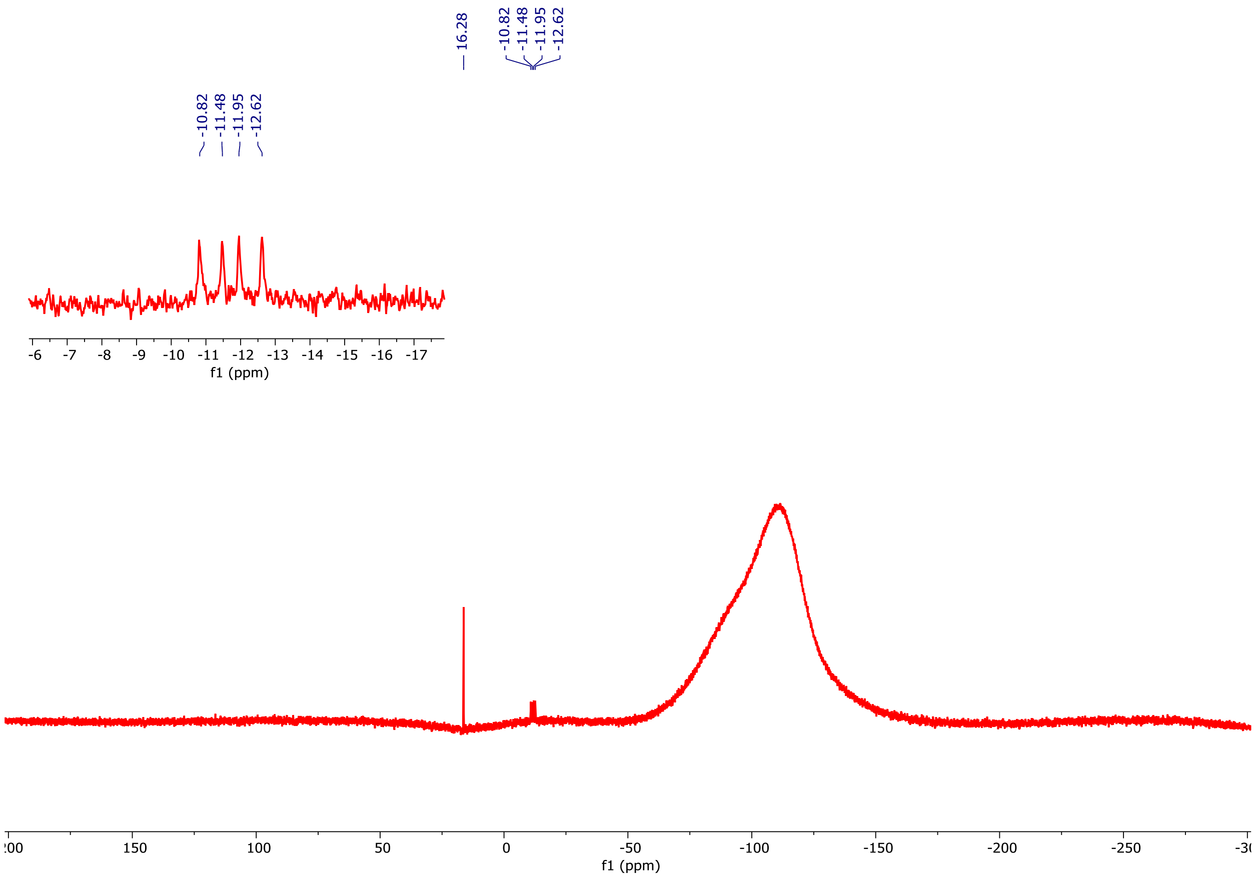

Figure S30. ^{29}Si NMR spectrum of compound **(4)** in THF (d_8).

(a)

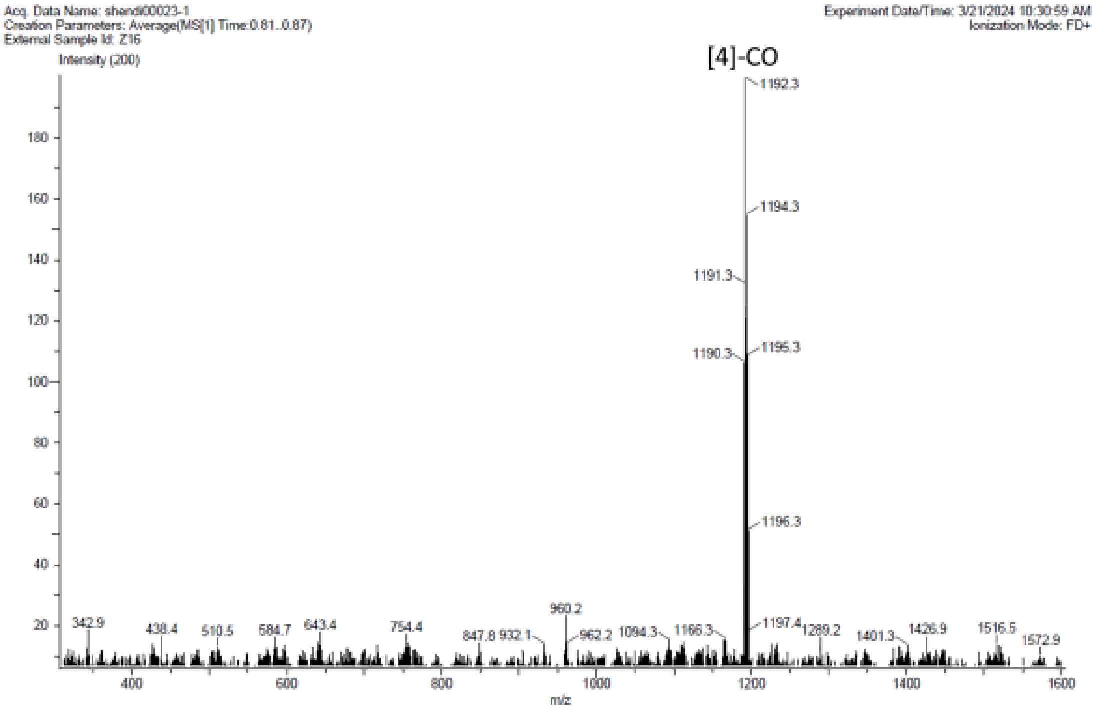

(b)

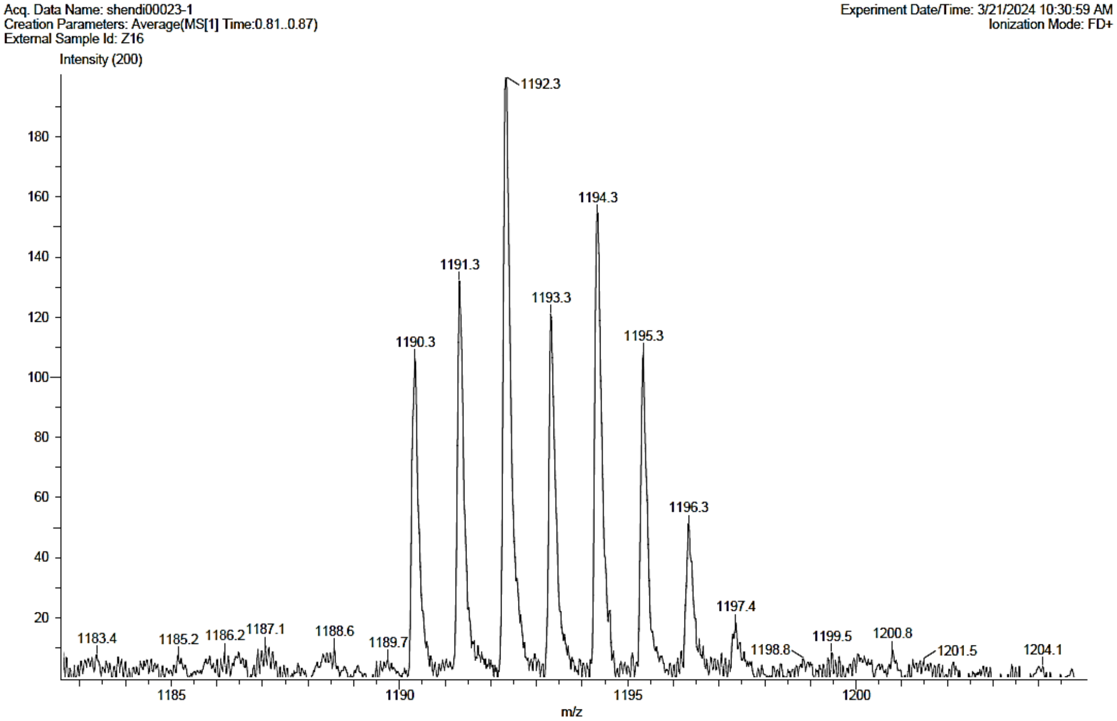

Figure S31: (a) LIFDI spectrum of compound (**4**), (b) expansion of the pattern.

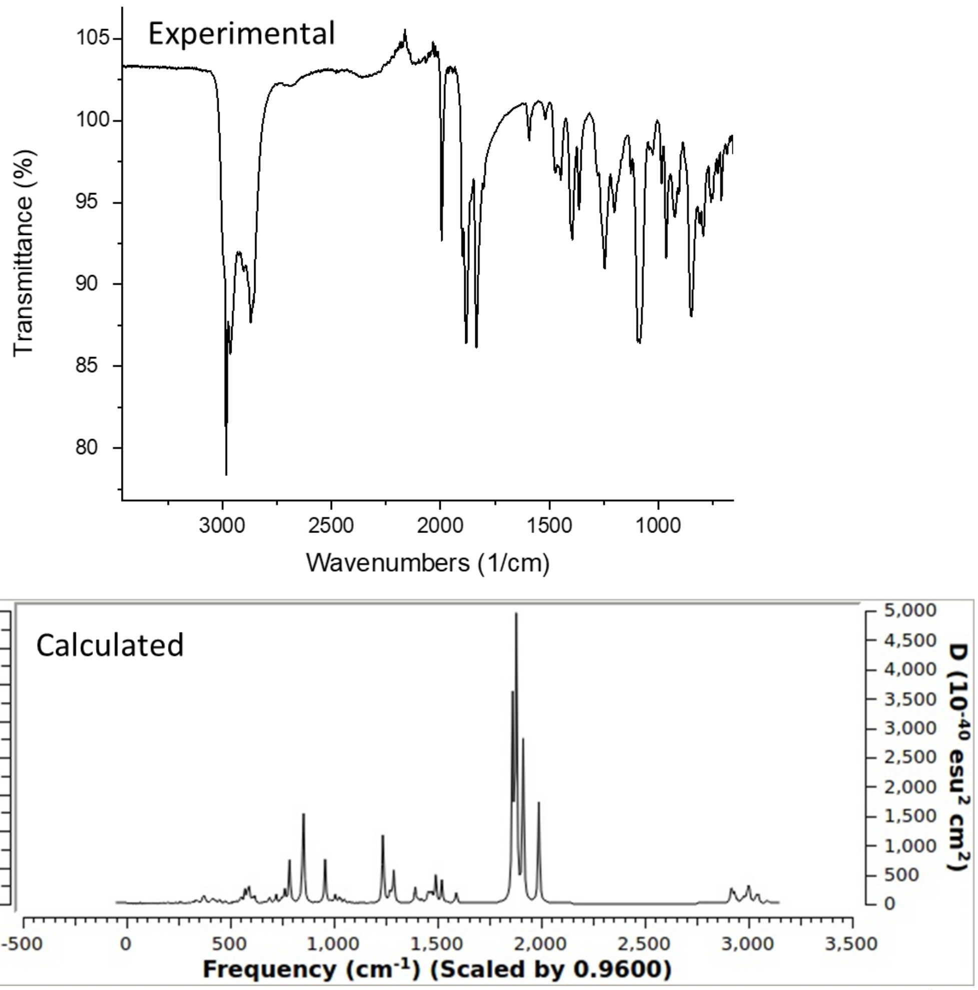

Figure S32: Experimental and calculated IR spectra of the compound (**4**) (cm^{-1}).

(E) Side product characterization

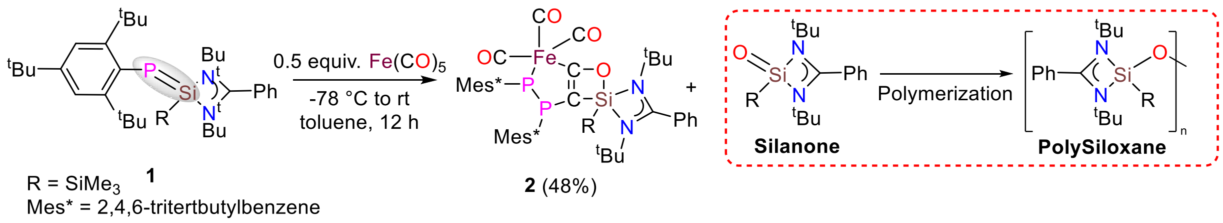

In order to separate the side product from the reaction mixture, after crystallization of compound **2** the hexane part was transferred to another RB. The RB was sealed and it was kept at -18 °C for one night which resulted in precipitation of the side product, the hexane part was separated from

the precipitate and the precipitate was washed with cold hexane to afford a white powder. The latter was dried under vacuum for 2 hours. The ^{29}Si-NMR is showing two resonances for the silicon atoms, one around δ 10.84 which was assigned to $SiMe_3$ group and the second one around δ 47.33, assigned to the centred oxygenated Si atom. This chemical shift agrees with the oxygenated silicone atoms (please see the references in the manuscript). The characteristic Si=O stretching vibration was found at 1196 cm^{-1} for both compounds (**Figure S20**), which is in good agreement with the calculated value ($\nu_{Si=O}$ =1183 cm^{-1}) and comparable to other Si=O containing structures.[9-11] The GIAO-derived ^{29}Si NMR chemical shift for silanone showed a chemical shift of 362.998-315.789 = 47.209 ppm related to Si=O motive (with respect to tetramethylsilane), which aligns well with the experimental value. For the $SiMe_3$ group, a shift of 23.553 ppm (339.342 - 315.789 ppm) was observed, which is slightly higher than the experimentally determined value.

^{1}H NMR (400 MHz, C_6D_6) δ = 7.44-7.40 (m, 1H, ArH), 6.91-6.87 (m, 1H, ArH), 6.82-6.77 (m, 3H, ArH), 1.12 (s, 18H, N-^{t}Bu), 0.35 (s, 9H, $SiMe_3$).

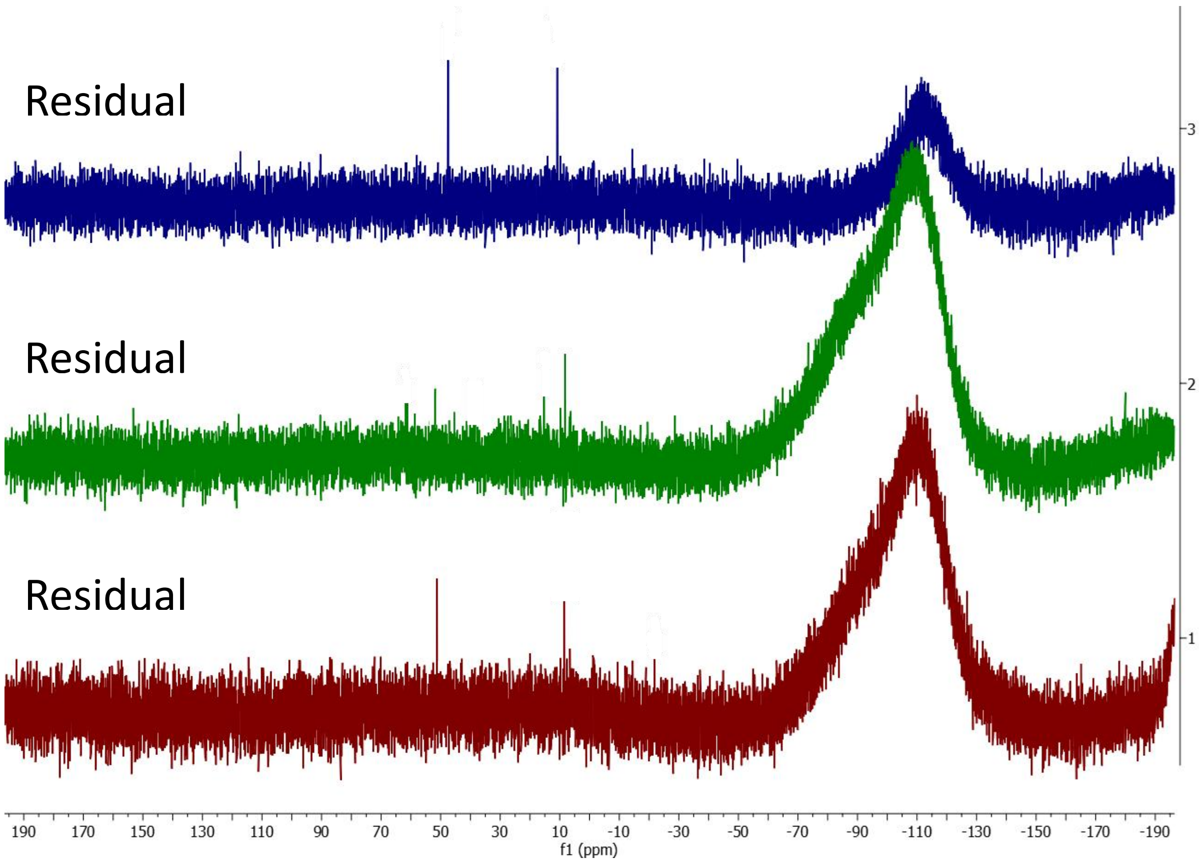

Figure S33. ^{29}Si NMR spectrum of the residual reaction mixture of the products in C_6D_6.

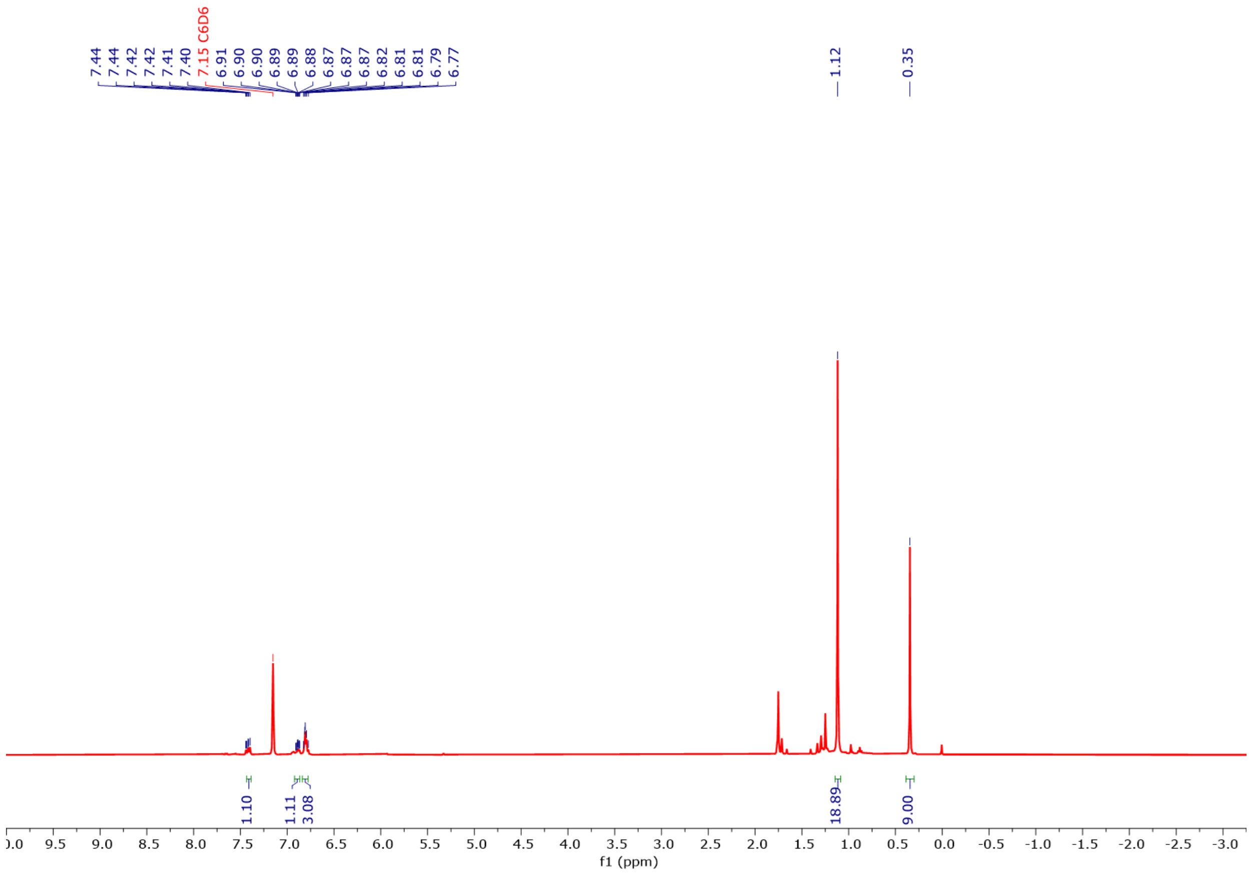

Figure S34. ^{1}H NMR spectrum of **Side product** in C_6D_6

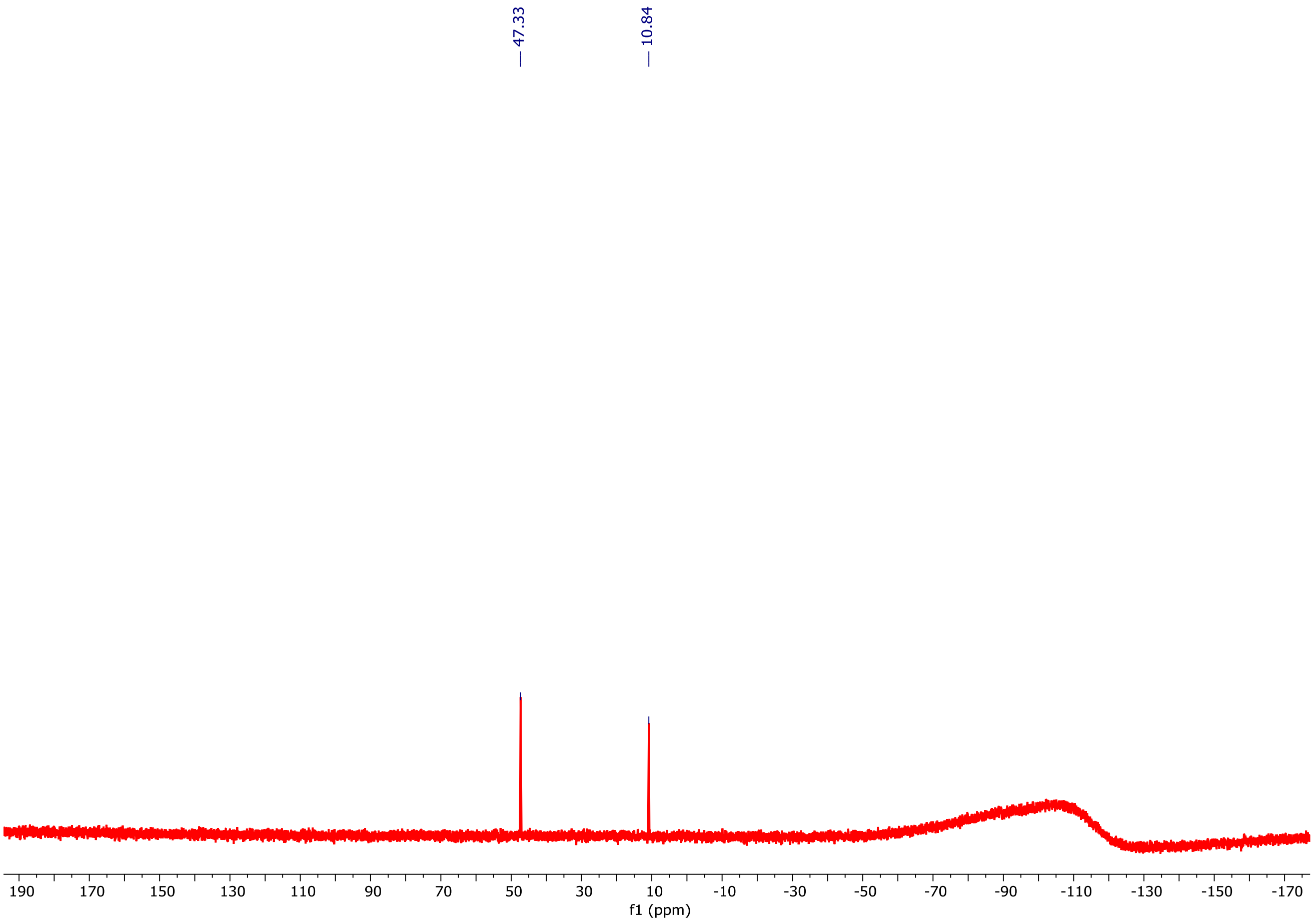

Figure S35. ^{29}Si NMR spectrum of **Side product** in C_6D_6

(a)

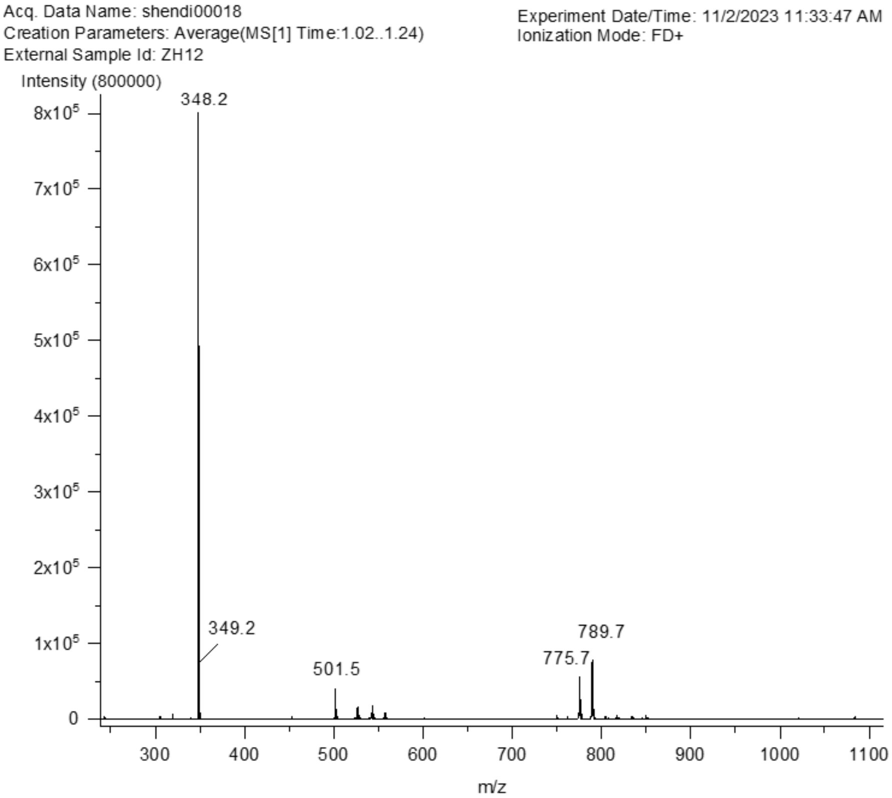

(b)

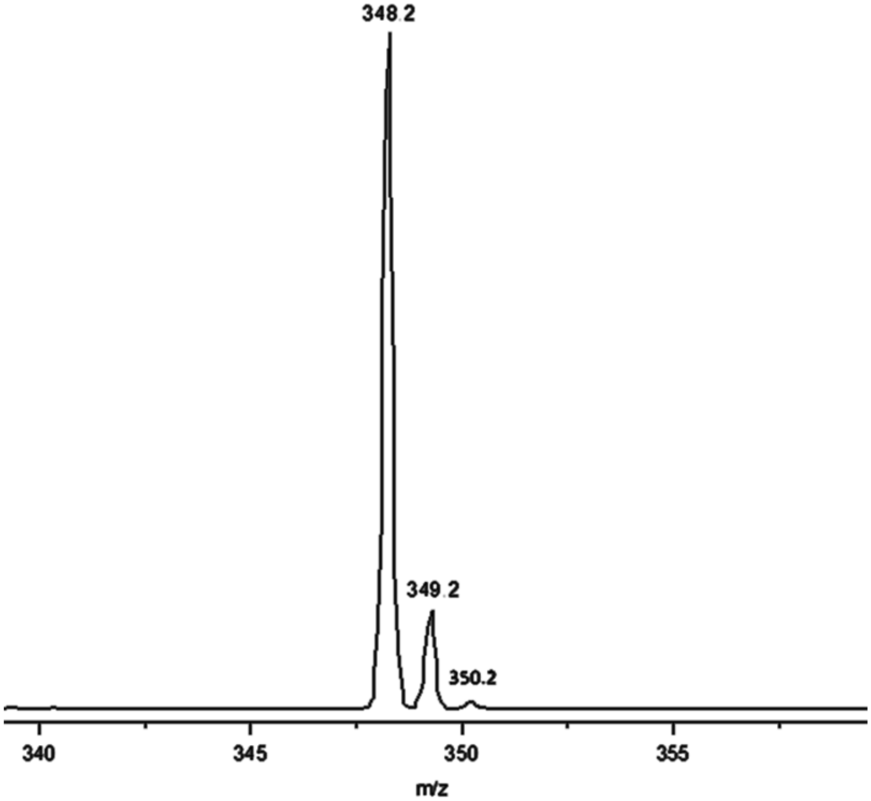

Figure S36: (a) LIFDI spectrum of the side product, (b) expansion of the pattern

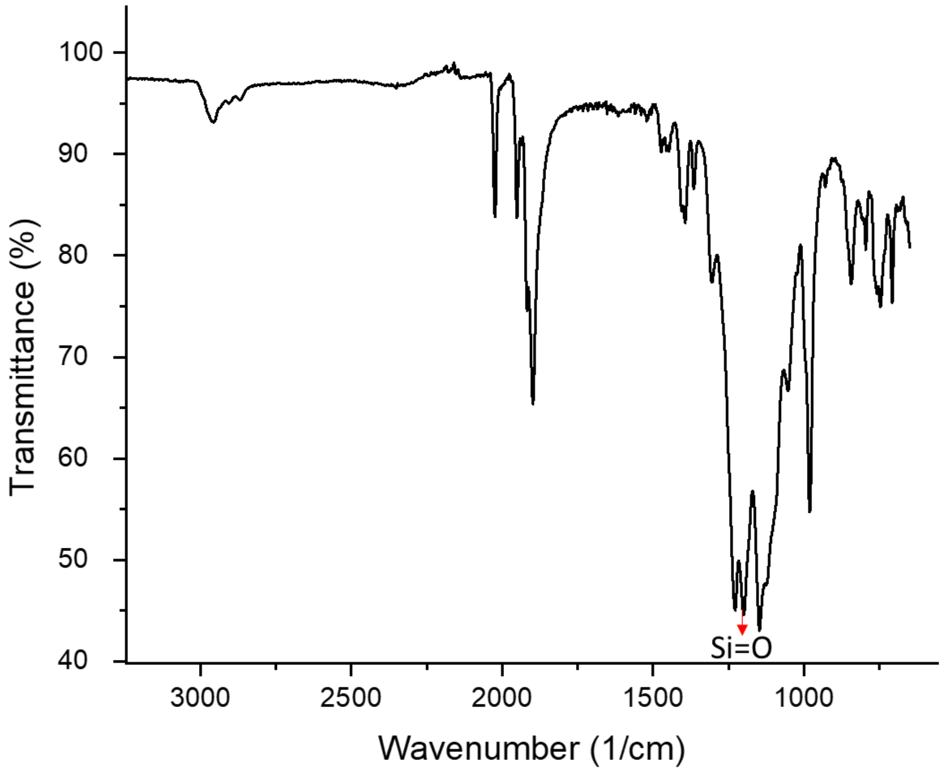

Figure S37: IR spectrum of the side product (cm^{-1}). Exp. ν [cm^{-1}] Si=O; 1196, Cal. ν [cm^{-1}] Si=O; 1183.

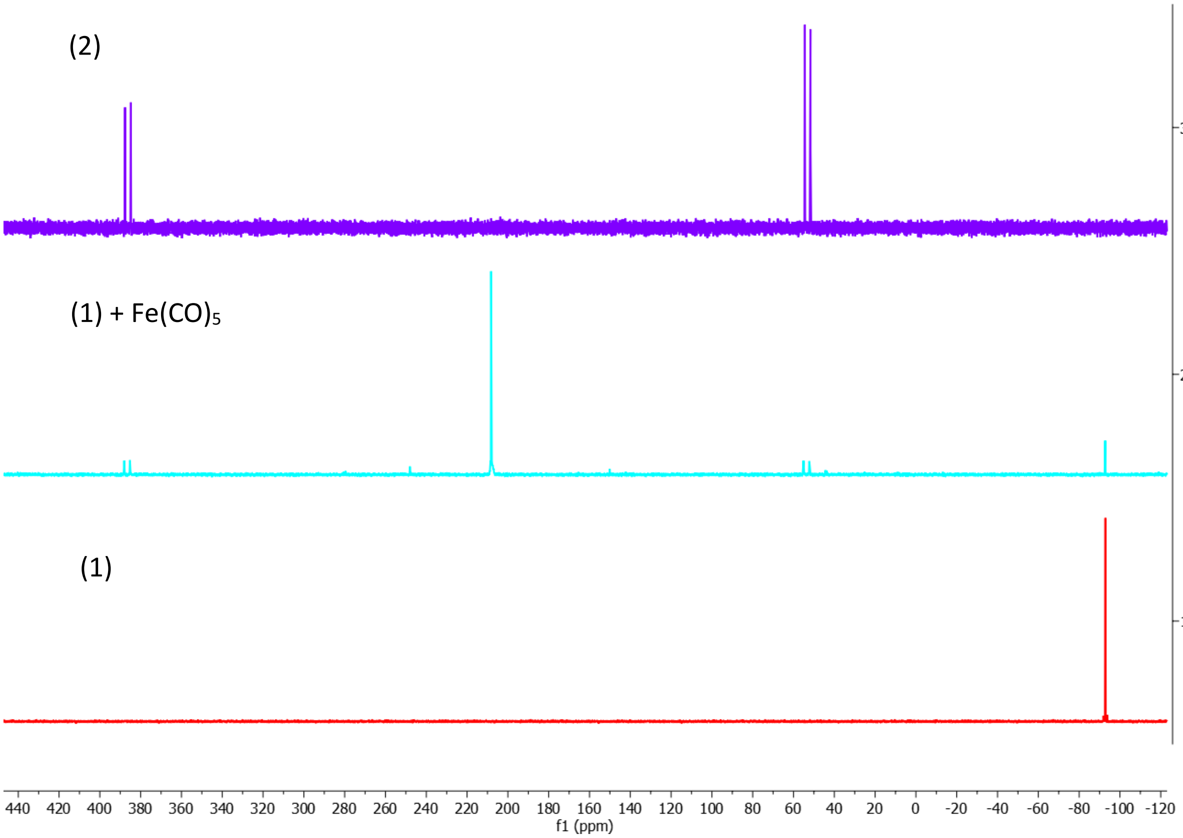

Figure **S38**. ^{31}P-NMR monitoring the reaction of **1** with $Fe(CO)_5$.

X-ray Structure Determination.

I. General Data Acquisition and Processing for 1, 3 and 4.

The diffraction data were collected using Mo Kα radiation and a Bruker Photon II detector. The data were integrated with SAINT.[3] A multi-scan absorption correction was applied using SADABS.[4] The structures were solved by SHELXT[5] and refined on F^2 using SHELXL[6] in the graphical user interface ShelXle.[7] All hydrogen atoms were placed with a riding model. An overview of the crystallographic data for **1** can be found in **Table S1**, while individual bond lengths and angles are listed in **Table S2, S4** and **S5**.

II. General Data Acquisition and Processing for 2.

A single-crystal of **2** suitable for X-ray diffraction analysis was mounted on a Cryoloop with a drop of Paratone oil and placed in the cold nitrogen stream on the STADIVARI diffractometer. The data was collected at 100 K using a Mo K*α* radiation source with the ω-scan technique and a EIGER2 R500 detector (Dectris Ltd, Switzerland). The data were integrated with STOE.[8] A multi-scan absorption correction was applied using STOE LANA.[8] The structures were solved by SHELXT[5] and refined on F^2 using SHELXL[6] in the graphical user interface ShelXle.[7] All hydrogen atoms were placed with a riding model. An overview of the crystallographic data for **2** can be found in **Table S1**, while individual bond lengths and angles are listed in **Table S3.**

Table S1: Crystal data and structure refinement details for 1-4.

	**1**	**2**	**3**	**4**
Formula	$C_{36}H_{61}N_2PSi_2$	$C_{59}H_{90}FeN_2O_4P_2Si_2$	$C_{90}H_{120}N_2O_5P_2Si_2Mo$	$C_{90}H_{120}N_2O_5P_2Si_2W$
no CCDC	2311389	2279235	2370052	2370053
Molecular weight	609.01	1065.29	1523.93	1611.84
Temperature (K)	100(2)	100	100(2)	100(2)
Wavelength	0.71073 Å	0.71073 Å	0.71073 Å	0.71073 Å
Crystal System	Monoclinic	Triclinic	Orthorhombic	Orthorhombic
Crystal size [mm]	0.132 x 0.118 x 0.053	0.300 x 0.173 x 0.070	0.189 x 0.109 x 0.035	0.61 x 0.23 x 0.22
Space group	$P2_1/n$	$P\bar{1}$	$P2_12_12_1$	$P2_12_12_1$
a (Å)	18.994(2)	13.3341(7)	14.748(2)	14.772(2)
b (Å)	11.672(2)	15.4736(9)	18.855(2)	18.812(2)
c (Å)	19.431(3)	16.8028(9)	31.225(4)	31.181(4)
α (°)	-	89.148(4)	90	90
β (°)	118.39(2)	68.098(4)	90	90
γ (°)	-	69.912(4)	90	90
V (Å^3)	3789.7(11)	2995.3(3)	8682.8(19)	8664.9(19)
Z	4	2	4	4
D_{calc} (g/cm^3)	1.067	1.181	1.166	1.236
μ (mm^{-1})	0.161	0.390	0.265	1.447
F(000)	1336.0	1148.0	3256	3384.0
Index ranges	$-22 \leq h \leq 22$<br>$-13 \leq k \leq 13$<br>$-23 \leq l \leq 23$	$-14 \leq h \leq 16$<br>$-19 \leq k \leq 17$<br>$-20 \leq l \leq 20$	$-18 \leq h \leq 18$<br>$-23 \leq k \leq 23$<br>$-39 \leq l \leq 38$	$-19 \leq h \leq 19$<br>$-25 \leq k \leq 25$<br>$41 \leq l \leq 41$
Data / restraints / parameters	6704/147/398	11773/1213/698	17772 / 5608 / 1266	21441/5608/1259
Angle range 2θ (°)	2.070 to 25.090°	2.534 to 26.022	1.693 to 26.414	1.264 to 28.341°
Reflection Collected	60239	102143	198112	205991
Independent Reflection	6704	11773	17772	21441
R_{int}	0.1570	0.1050	0.0836	0.0357
Refinement method	Full-matrix least-squares on F^2	Full-matrix least-squares on F^2	Full-matrix least-squares on F^2	Full-matrix least-squares on F^2
Completeness to theta = 25.242°/25.090°(for **1**)	99.5%	99.7%	99.9%	99.9%
R_1 ($I < 2\sigma(I)$))	0.0514	0.0576	0.0436	0.0207
wR_2 (all data)	0.1232	0.1975	0.0994	0.0504
GOOF	1.050	1.000	1.043	1.078
Absolute structure parameter	-	-	0.47(3)	0.0057(11)
ρ_{min}, ρ_{max}, e/Å^3	0.393/-0.287	1.227/-1.272	0.682/ -0.497	1.692/-0.700

$R_1 = \Sigma||F_o| - |F_c||/\Sigma|F_o|$; $wR_2 = \{\Sigma[w(F_o^2 - F_c^2)^2]/\Sigma[w(F_o^2)^2]\}^{1/2}$; $w = 1/[\sigma^2(F_o^2)+(aP)^2 + bP]$, where $P = (F_o^2 + 2F_c^2)/3$; $s = \{\Sigma[w(F_o^2 - F_c^2)]/(n - p)\}^{1/2}$ where n is the number of reflections and p is the number of refinement parameters.

Crystal structure of 1

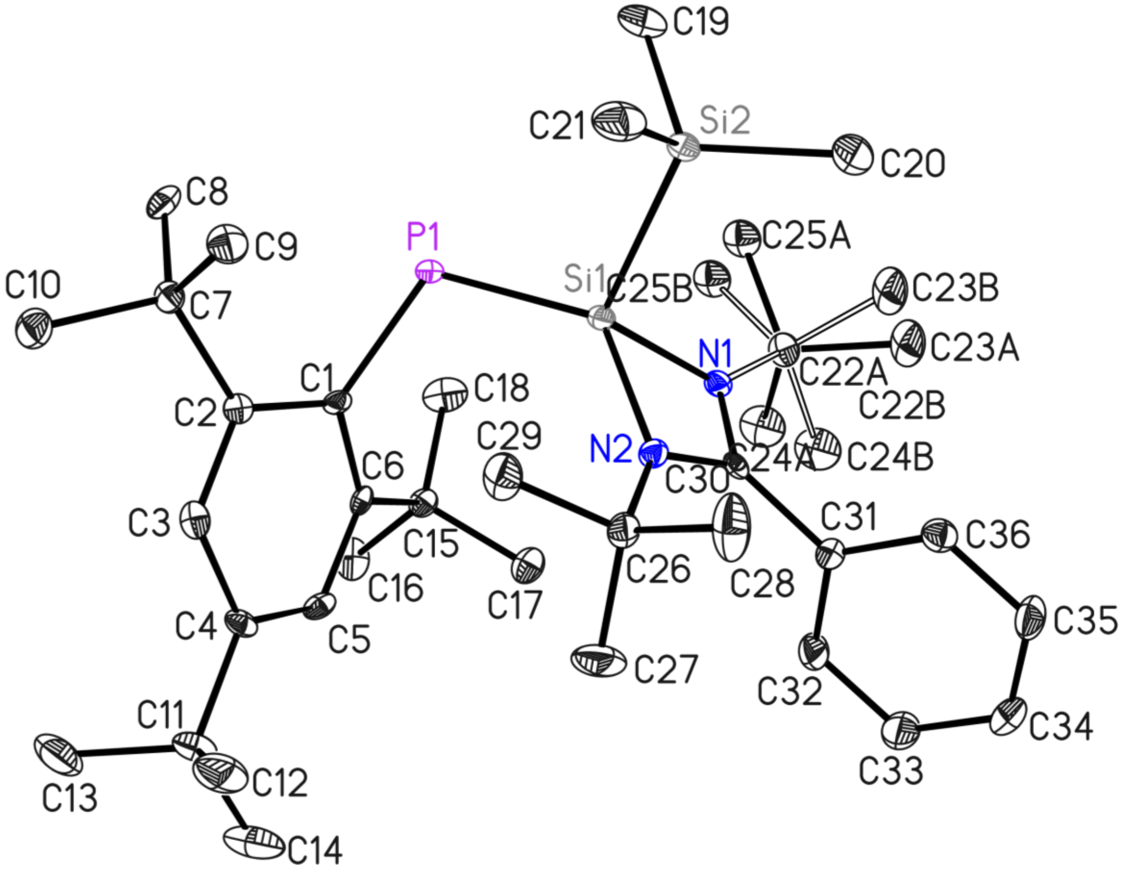

Figure S39: Asymmetric unit of **1** with thermal ellipsoids shown at 50% probability level. Hydrogen atoms are omitted for clarity. One *tert*-butyl group is disordered over two positions and was refined with distance restraints and restraints for the anisotropic displacement parameters. The occupancy of the minor component refined to 0.152(6). Selected bond lengths (Å) and bond angles (°). P1–C1 1.874(2), P1–Si1 2.1250(10), N1–Si1 1.888(2), N2–Si1 1.833(2), Si1–Si2 2.3501(12), N1–Si1–N2 70.39(9), N1–Si1–Si2 106.67(7), N2– Si1–Si2 110.16(8), P1–Si1–Si2 104.80(4), C1–P1–Si1 109.30(8).

Table S2. Bond length [Å] and angles [°] for **1**.

P(1)-C(1)	1.874(2)	C(22B)-C(25B)	1.498(11)
P(1)-Si(1)	2.1250(10)	C(22B)-C(24B)	1.527(11)
Si(1)-N(2)	1.833(2)	C(22B)-C(23B)	1.546(12)
Si(1)-N(1)	1.888(2)	C(26)-C(27)	1.524(4)
Si(1)-C(30)	2.313(3)	C(26)-C(29)	1.526(4)
Si(1)-Si(2)	2.3501(12)	C(26)-C(28)	1.527(4)
Si(2)-C(20)	1.871(3)	C(30)-C(31)	1.489(4)
Si(2)-C(21)	1.875(3)	C(31)-C(36)	1.385(4)
Si(2)-C(19)	1.877(3)	C(31)-C(32)	1.388(4)
N(2)-C(30)	1.344(3)	C(32)-C(33)	1.384(4)
N(2)-C(26)	1.482(3)	C(33)-C(34)	1.376(4)
C(1)-C(6)	1.422(3)	C(34)-C(35)	1.383(4)
C(1)-C(2)	1.439(3)	C(35)-C(36)	1.384(4)
C(2)-C(3)	1.386(3)		
C(2)-C(7)	1.557(3)	C(1)-P(1)-Si(1)	109.30(8)
C(3)-C(4)	1.396(4)	N(2)-Si(1)-N(1)	70.39(9)
C(4)-C(5)	1.381(4)	N(2)-Si(1)-P(1)	127.74(7)
C(4)-C(11)	1.534(3)	N(1)-Si(1)-P(1)	133.12(8)
C(5)-C(6)	1.403(3)	N(2)-Si(1)-C(30)	35.48(9)
C(6)-C(15)	1.552(3)	N(1)-Si(1)-C(30)	35.04(9)
C(7)-C(9)	1.534(4)	P(1)-Si(1)-C(30)	144.76(7)
C(7)-C(8)	1.535(4)	N(2)-Si(1)-Si(2)	110.16(8)
C(7)-C(10)	1.542(4)	N(1)-Si(1)-Si(2)	106.67(7)
C(11)-C(14)	1.526(4)	P(1)-Si(1)-Si(2)	104.80(4)
C(11)-C(12)	1.530(4)	C(30)-Si(1)-Si(2)	110.41(7)
C(11)-C(13)	1.538(4)	C(20)-Si(2)-C(21)	106.22(15)
C(15)-C(17)	1.523(4)	C(20)-Si(2)-C(19)	110.12(14)
C(15)-C(18)	1.530(3)	C(21)-Si(2)-C(19)	110.49(14)
C(15)-C(16)	1.549(4)	C(20)-Si(2)-Si(1)	113.19(10)
N(1)-C(30)	1.328(3)	C(21)-Si(2)-Si(1)	109.04(10)
N(1)-C(22B)	1.496(3)	C(19)-Si(2)-Si(1)	107.78(10)
N(1)-C(22A)	1.496(3)	C(30)-N(2)-C(26)	130.8(2)
C(22A)-C(23A)	1.519(4)	C(30)-N(2)-Si(1)	92.15(15)
C(22A)-C(25A)	1.527(4)	C(26)-N(2)-Si(1)	137.06(17)
C(22A)-C(24A)	1.541(4)	C(6)-C(1)-C(2)	116.6(2)

C(6)-C(1)-P(1)	125.83(19)
C(2)-C(1)-P(1)	116.94(18)
C(3)-C(2)-C(1)	118.6(2)
C(3)-C(2)-C(7)	117.2(2)
C(1)-C(2)-C(7)	124.2(2)
C(2)-C(3)-C(4)	123.6(2)
C(5)-C(4)-C(3)	115.8(2)
C(5)-C(4)-C(11)	123.8(2)
C(3)-C(4)-C(11)	120.3(2)
C(4)-C(5)-C(6)	123.4(2)
C(5)-C(6)-C(1)	118.7(2)
C(5)-C(6)-C(15)	114.6(2)
C(1)-C(6)-C(15)	126.6(2)
C(9)-C(7)-C(8)	110.2(2)
C(9)-C(7)-C(10)	107.2(2)
C(8)-C(7)-C(10)	105.4(2)
C(9)-C(7)-C(2)	111.1(2)
C(8)-C(7)-C(2)	112.0(2)
C(10)-C(7)-C(2)	110.6(2)
C(14)-C(11)-C(12)	107.7(2)
C(14)-C(11)-C(4)	112.1(2)
C(12)-C(11)-C(4)	109.3(2)
C(14)-C(11)-C(13)	108.8(2)
C(12)-C(11)-C(13)	108.8(2)
C(4)-C(11)-C(13)	110.0(2)
C(17)-C(15)-C(18)	109.5(2)
C(17)-C(15)-C(16)	108.2(2)
C(18)-C(15)-C(16)	104.6(2)
C(17)-C(15)-C(6)	110.4(2)
C(18)-C(15)-C(6)	115.3(2)
C(16)-C(15)-C(6)	108.4(2)
C(30)-N(1)-C(22B)	131.0(2)
C(30)-N(1)-C(22A)	131.0(2)
C(30)-N(1)-Si(1)	90.27(16)
C(22B)-N(1)-Si(1)	136.94(16)
C(22A)-N(1)-Si(1)	136.94(16)
N(1)-C(22A)-C(23A)	113.3(2)
N(1)-C(22A)-C(25A)	106.0(2)
C(23A)-C(22A)-C(25A)	109.4(2)
N(1)-C(22A)-C(24A)	110.7(2)
C(23A)-C(22A)-C(24A)	109.0(3)
C(25A)-C(22A)-C(24A)	108.2(3)
N(1)-C(22B)-C(25B)	106.1(8)
N(1)-C(22B)-C(24B)	109.6(7)
C(25B)-C(22B)-C(24B)	112.6(10)
N(1)-C(22B)-C(23B)	106.1(8)
C(25B)-C(22B)-C(23B)	113.8(10)
C(24B)-C(22B)-C(23B)	108.3(10)
N(2)-C(26)-C(27)	110.6(2)
N(2)-C(26)-C(29)	106.3(2)
C(27)-C(26)-C(29)	108.5(2)
N(2)-C(26)-C(28)	110.4(2)
C(27)-C(26)-C(28)	110.8(2)
C(29)-C(26)-C(28)	110.1(2)
N(1)-C(30)-N(2)	106.8(2)
N(1)-C(30)-C(31)	126.5(2)
N(2)-C(30)-C(31)	126.6(2)
N(1)-C(30)-Si(1)	54.69(13)
N(2)-C(30)-Si(1)	52.37(12)
C(31)-C(30)-Si(1)	174.08(19)
C(36)-C(31)-C(32)	119.5(2)
C(36)-C(31)-C(30)	120.0(2)
C(32)-C(31)-C(30)	120.5(2)
C(33)-C(32)-C(31)	120.0(3)
C(34)-C(33)-C(32)	120.3(3)
C(33)-C(34)-C(35)	120.0(3)
C(34)-C(35)-C(36)	120.1(3)
C(35)-C(36)-C(31)	120.1(3)

Crystal structure of 2.

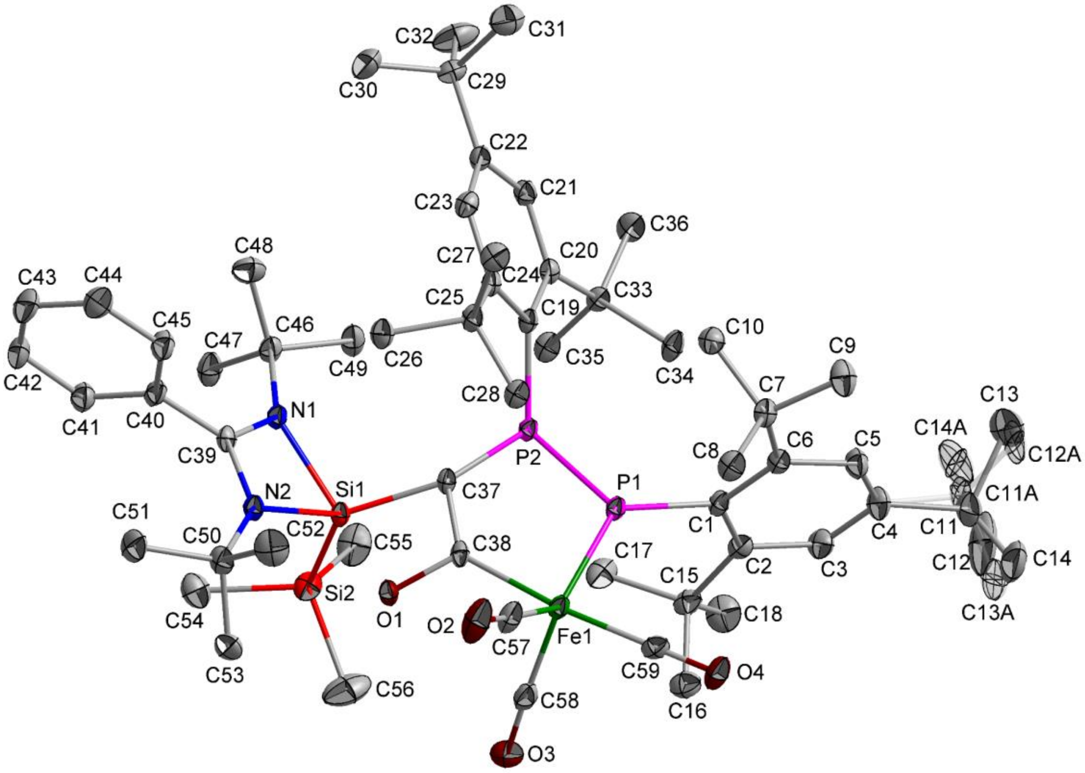

Figure S40: Asymmetric unit of **2** with thermal ellipsoids shown at 50% probability level. Hydrogen atoms are omitted for clarity. One *tert*-butyl group is disordered over two positions and was refined with distance restraints and restraints for the anisotropic displacement parameters. The occupancy of the minor component was refined to 0.364(6). Selected bond lengths (Å) and bond angles (°). Fe1–P1 2.1133(7), Fe1–C38 1.982(3), Fe1–C57 1.803(3), C57–O2 1.134(3), O1–Si1 1.8093(19), Si1–Si2 2.3453(10), C37–C38 1.371(4), C38-O1 1.366(3)Å, P1–P2 2.2028(9), C1–P1–P2 116.60(8).

Table S3. Bond length [Å] and angles [°] for **2.**

Fe(1)-P(1)	2.1133(7)	C(50)-C(51)	1.526(4)
Fe(1)-C(38)	1.982(3)	C(38)-C(37)	1.371(4)
Fe(1)-C(58)	1.779(3)	C(46)-C(48)	1.525(4)
Fe(1)-C(59)	1.805(3)	C(46)-C(49)	1.524(3)
Fe(1)-C(57)	1.803(3)	C(46)-C(47)	1.527(4)
Si(2)-Si(1)	2.3453(10)	C(1)-C(6)	1.419(4)
Si(2)-C(56)	1.862(4)	C(1)-C(2)	1.445(3)
Si(2)-C(55)	1.881(3)	C(6)-C(5)	1.393(3)
Si(2)-C(54)	1.888(3)	C(6)-C(7)	1.553(3)
O(1)-Si(1)	1.8093(19)	C(5)-C(4)	1.371(4)
O(1)-C(38)	1.366(3)	C(4)-C(3)	1.371(4)
N(1)-Si(1)	1.975(2)	C(4)-C(11)	1.553(7)
N(1)-C(39)	1.317(3)	C(4)-C(11A)	1.546(10)
N(1)-C(46)	1.479(3)	C(3)-C(2)	1.400(4)
C(40)-C(41)	1.388(4)	C(2)-C(15)	1.562(4)
C(40)-C(45)	1.393(4)	C(19)-C(24)	1.436(3)
C(40)-C(39)	1.497(3)	C(19)-C(20)	1.446(3)
Si(1)-N(2)	1.841(2)	C(24)-C(23)	1.388(4)
Si(1)-C(39)	2.369(3)	C(24)-C(25)	1.565(3)
Si(1)-C(38)	2.273(3)	C(23)-C(22)	1.381(4)
Si(1)-C(37)	1.888(2)	C(22)-C(21)	1.388(4)
O(3)-C(58)	1.154(4)	C(22)-C(29)	1.534(4)
N(2)-C(39)	1.340(3)	C(21)-C(20)	1.377(4)
N(2)-C(50)	1.489(3)	C(20)-C(33)	1.559(3)
C(41)-C(42)	1.390(4)	C(7)-C(9)	1.538(4)
P(1)-P(2)	2.2028(9)	C(7)-C(8)	1.535(3)
P(1)-C(1)	1.845(2)	C(7)-C(10)	1.536(4)
O(4)-C(59)	1.144(3)	C(11)-C(12)	1.523(9)
C(42)-C(43)	1.380(5)	C(11)-C(13)	1.511(9)
P(2)-C(37)	1.759(2)	C(11)-C(14)	1.523(9)
P(2)-C(19)	1.859(3)	C(15)-C(16)	1.523(4)
O(2)-C(57)	1.134(3)	C(15)-C(18)	1.547(4)
C(43)-C(44)	1.380(4)	C(15)-C(17)	1.547(4)
C(44)-C(45)	1.383(4)	C(25)-C(26)	1.540(3)
C(50)-C(52)	1.520(4)	C(25)-C(28)	1.531(4)
C(50)-C(53)	1.533(4)	C(25)-C(27)	1.530(4)

C(29)-C(30)	1.520(4)
C(29)-C(31)	1.535(4)
C(29)-C(32)	1.533(4)
C(33)-C(35)	1.528(4)
C(33)-C(36)	1.549(4)
C(33)-C(34)	1.533(4)
C(13A)-C(11A)	1.502(14)
C(11A)-C(12A)	1.512(13)
C(11A)-C(14A)	1.528(13)

C(38)-Fe(1)-P(1)	83.94(7)
C(58)-Fe(1)-P(1)	127.79(9)
C(58)-Fe(1)-C(38)	89.03(12)
C(58)-Fe(1)-C(59)	94.25(12)
C(58)-Fe(1)-C(57)	99.81(13)
C(59)-Fe(1)-P(1)	89.45(8)
C(59)-Fe(1)-C(38)	173.32(11)
C(57)-Fe(1)-P(1)	130.89(10)
C(57)-Fe(1)-C(38)	85.71(11)
C(57)-Fe(1)-C(59)	99.44(12)
C(56)-Si(2)-Si(1)	109.29(11)
C(56)-Si(2)-C(55)	105.53(17)
C(56)-Si(2)-C(54)	108.65(17)
C(55)-Si(2)-Si(1)	113.73(11)
C(55)-Si(2)-C(54)	110.44(14)
C(54)-Si(2)-Si(1)	109.05(10)
C(38)-O(1)-Si(1)	90.32(15)
C(39)-N(1)-Si(1)	89.76(16)
C(39)-N(1)-C(46)	128.8(2)
C(46)-N(1)-Si(1)	140.72(17)
C(41)-C(40)-C(45)	119.6(2)
C(41)-C(40)-C(39)	121.4(2)
C(45)-C(40)-C(39)	118.9(2)
Si(2)-Si(1)-C(39)	107.87(7)
O(1)-Si(1)-Si(2)	99.20(7)
O(1)-Si(1)-N(1)	157.84(9)
O(1)-Si(1)-N(2)	94.53(9)
O(1)-Si(1)-C(39)	127.78(9)
O(1)-Si(1)-C(38)	36.93(8)
O(1)-Si(1)-C(37)	73.81(9)
N(1)-Si(1)-Si(2)	99.84(7)
N(1)-Si(1)-C(39)	33.76(9)
N(1)-Si(1)-C(38)	140.64(10)
N(2)-Si(1)-Si(2)	110.97(7)
N(2)-Si(1)-N(1)	68.03(9)
N(2)-Si(1)-C(39)	34.28(9)
N(2)-Si(1)-C(38)	119.20(9)
N(2)-Si(1)-C(37)	130.35(10)
C(38)-Si(1)-Si(2)	110.91(7)
C(38)-Si(1)-C(39)	140.27(9)
C(37)-Si(1)-Si(2)	118.44(8)
C(37)-Si(1)-N(1)	106.45(10)
C(37)-Si(1)-C(39)	124.69(10)
C(37)-Si(1)-C(38)	37.04(10)
C(39)-N(2)-Si(1)	94.97(15)
C(39)-N(2)-C(50)	131.4(2)
C(50)-N(2)-Si(1)	133.62(18)
C(40)-C(41)-C(42)	119.6(3)
Fe(1)-P(1)-P(2)	111.04(3)
C(1)-P(1)-Fe(1)	127.14(9)
C(1)-P(1)-P(2)	116.60(8)
C(43)-C(42)-C(41)	120.5(3)
C(37)-P(2)-P(1)	92.89(9)
C(37)-P(2)-C(19)	113.80(11)
C(19)-P(2)-P(1)	119.93(8)
C(42)-C(43)-C(44)	120.0(3)
C(43)-C(44)-C(45)	120.1(3)
C(44)-C(45)-C(40)	120.2(3)
N(1)-C(39)-C(40)	126.4(2)
N(1)-C(39)-Si(1)	56.48(13)
N(1)-C(39)-N(2)	107.2(2)
C(40)-C(39)-Si(1)	176.58(19)
N(2)-C(39)-C(40)	126.3(2)
N(2)-C(39)-Si(1)	50.74(12)
N(2)-C(50)-C(52)	108.2(2)
N(2)-C(50)-C(53)	107.9(2)

N(2)-C(50)-C(51)	113.7(2)	C(19)-C(24)-C(25)	129.5(2)
C(52)-C(50)-C(53)	110.5(2)	C(23)-C(24)-C(19)	117.6(2)
C(52)-C(50)-C(51)	109.8(2)	C(23)-C(24)-C(25)	112.9(2)
C(51)-C(50)-C(53)	106.7(2)	C(22)-C(23)-C(24)	124.7(2)
Fe(1)-C(38)-Si(1)	169.68(14)	C(23)-C(22)-C(21)	116.3(3)
O(1)-C(38)-Fe(1)	119.63(18)	C(23)-C(22)-C(29)	121.6(2)
O(1)-C(38)-Si(1)	52.75(11)	C(21)-C(22)-C(29)	122.0(2)
O(1)-C(38)-C(37)	108.5(2)	C(20)-C(21)-C(22)	123.7(2)
C(37)-C(38)-Fe(1)	131.85(18)	C(19)-C(20)-C(33)	125.5(2)
C(37)-C(38)-Si(1)	56.02(13)	C(21)-C(20)-C(19)	118.7(2)
P(2)-C(37)-Si(1)	146.22(15)	C(21)-C(20)-C(33)	115.7(2)
C(38)-C(37)-Si(1)	86.93(16)	O(3)-C(58)-Fe(1)	176.8(3)
C(38)-C(37)-P(2)	118.66(18)	O(4)-C(59)-Fe(1)	177.8(2)
N(1)-C(46)-C(48)	113.8(2)	O(2)-C(57)-Fe(1)	176.9(3)
N(1)-C(46)-C(49)	105.9(2)	C(9)-C(7)-C(6)	112.9(2)
N(1)-C(46)-C(47)	109.9(2)	C(8)-C(7)-C(6)	111.1(2)
C(48)-C(46)-C(47)	109.8(2)	C(8)-C(7)-C(9)	105.1(2)
C(49)-C(46)-C(48)	108.2(2)	C(8)-C(7)-C(10)	112.1(2)
C(49)-C(46)-C(47)	109.1(2)	C(10)-C(7)-C(6)	109.9(2)
C(6)-C(1)-P(1)	120.75(18)	C(10)-C(7)-C(9)	105.6(2)
C(6)-C(1)-C(2)	119.5(2)	C(12)-C(11)-C(4)	111.8(5)
C(2)-C(1)-P(1)	119.57(19)	C(13)-C(11)-C(4)	110.2(6)
C(1)-C(6)-C(7)	125.7(2)	C(13)-C(11)-C(12)	108.8(7)
C(5)-C(6)-C(1)	118.6(2)	C(13)-C(11)-C(14)	106.7(7)
C(5)-C(6)-C(7)	115.7(2)	C(14)-C(11)-C(4)	111.8(5)
C(4)-C(5)-C(6)	123.2(3)	C(14)-C(11)-C(12)	107.3(6)
C(5)-C(4)-C(11)	118.1(4)	C(16)-C(15)-C(2)	108.3(2)
C(5)-C(4)-C(11A)	123.5(5)	C(16)-C(15)-C(18)	106.4(2)
C(3)-C(4)-C(5)	117.9(2)	C(16)-C(15)-C(17)	109.6(2)
C(3)-C(4)-C(11)	123.5(4)	C(18)-C(15)-C(2)	111.9(2)
C(3)-C(4)-C(11A)	118.0(5)	C(17)-C(15)-C(2)	116.3(2)
C(4)-C(3)-C(2)	124.0(3)	C(17)-C(15)-C(18)	103.9(2)
C(1)-C(2)-C(15)	129.0(2)	C(26)-C(25)-C(24)	108.7(2)
C(3)-C(2)-C(1)	116.8(2)	C(28)-C(25)-C(24)	116.9(2)
C(3)-C(2)-C(15)	114.1(2)	C(28)-C(25)-C(26)	108.8(2)
C(24)-C(19)-P(2)	126.14(18)	C(27)-C(25)-C(24)	109.5(2)
C(24)-C(19)-C(20)	118.0(2)	C(27)-C(25)-C(26)	108.3(2)
C(20)-C(19)-P(2)	113.91(18)	C(27)-C(25)-C(28)	104.3(2)

C(22)-C(29)-C(31)	108.6(2)	C(36)-C(33)-C(20)	111.3(2)
C(22)-C(29)-C(32)	112.4(2)	C(34)-C(33)-C(20)	111.0(2)
C(30)-C(29)-C(22)	110.6(2)	C(34)-C(33)-C(36)	104.7(2)
C(30)-C(29)-C(31)	109.4(3)	C(4)-C(11A)-C(12A)	109.9(9)
C(30)-C(29)-C(32)	108.3(3)	C(13A)-C(11A)-C(4)	105.0(8)
C(32)-C(29)-C(31)	107.4(2)	C(13A)-C(11A)-C(12A)	111.1(11)
C(35)-C(33)-C(20)	112.1(2)	C(13A)-C(11A)-C(14A)	109.4(10)
C(35)-C(33)-C(36)	105.9(2)	C(14A)-C(11A)-C(4)	111.4(8)
C(35)-C(33)-C(34)	111.3(2)	C(14A)-C(11A)-C(12A)	109.9(10)

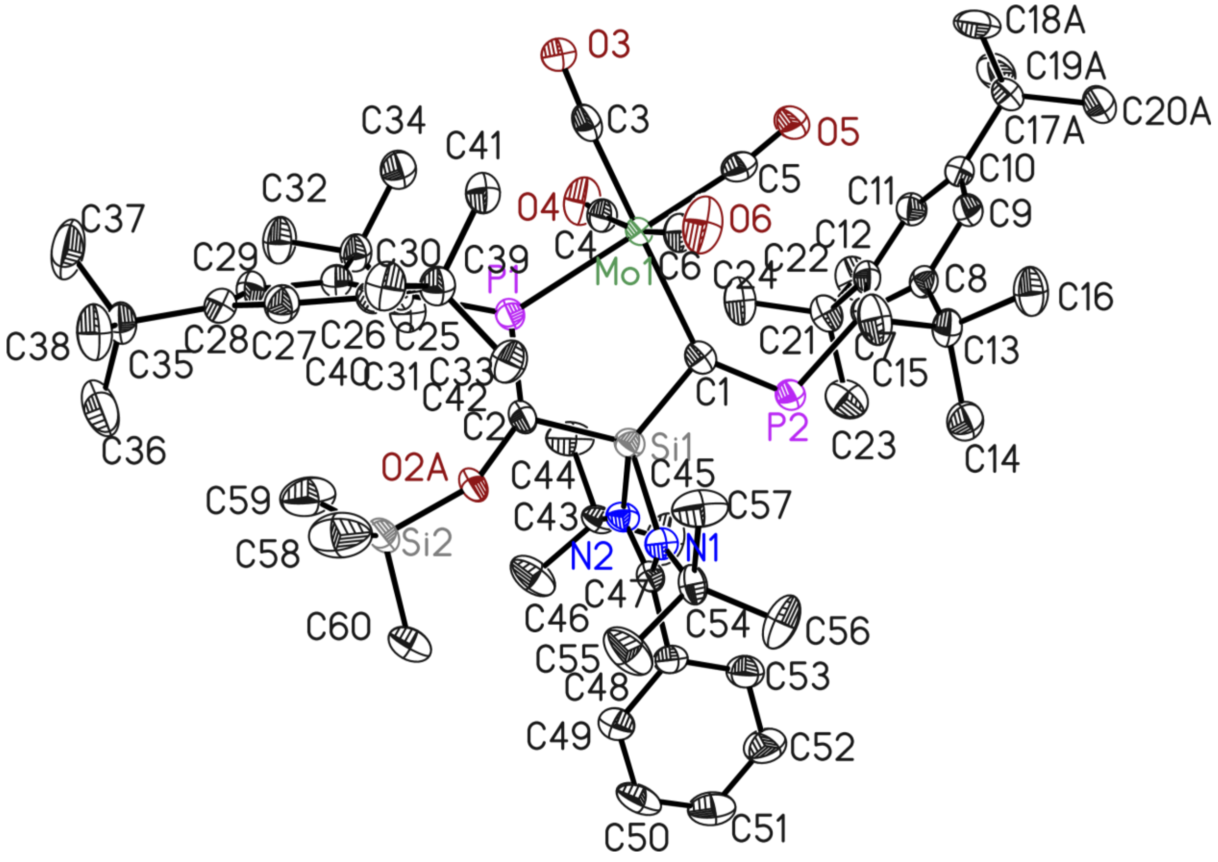

Figure S41: Molecular structure of **3** with thermal ellipsoids shown at 50% probability level. Hydrogen atoms are omitted for clarity.

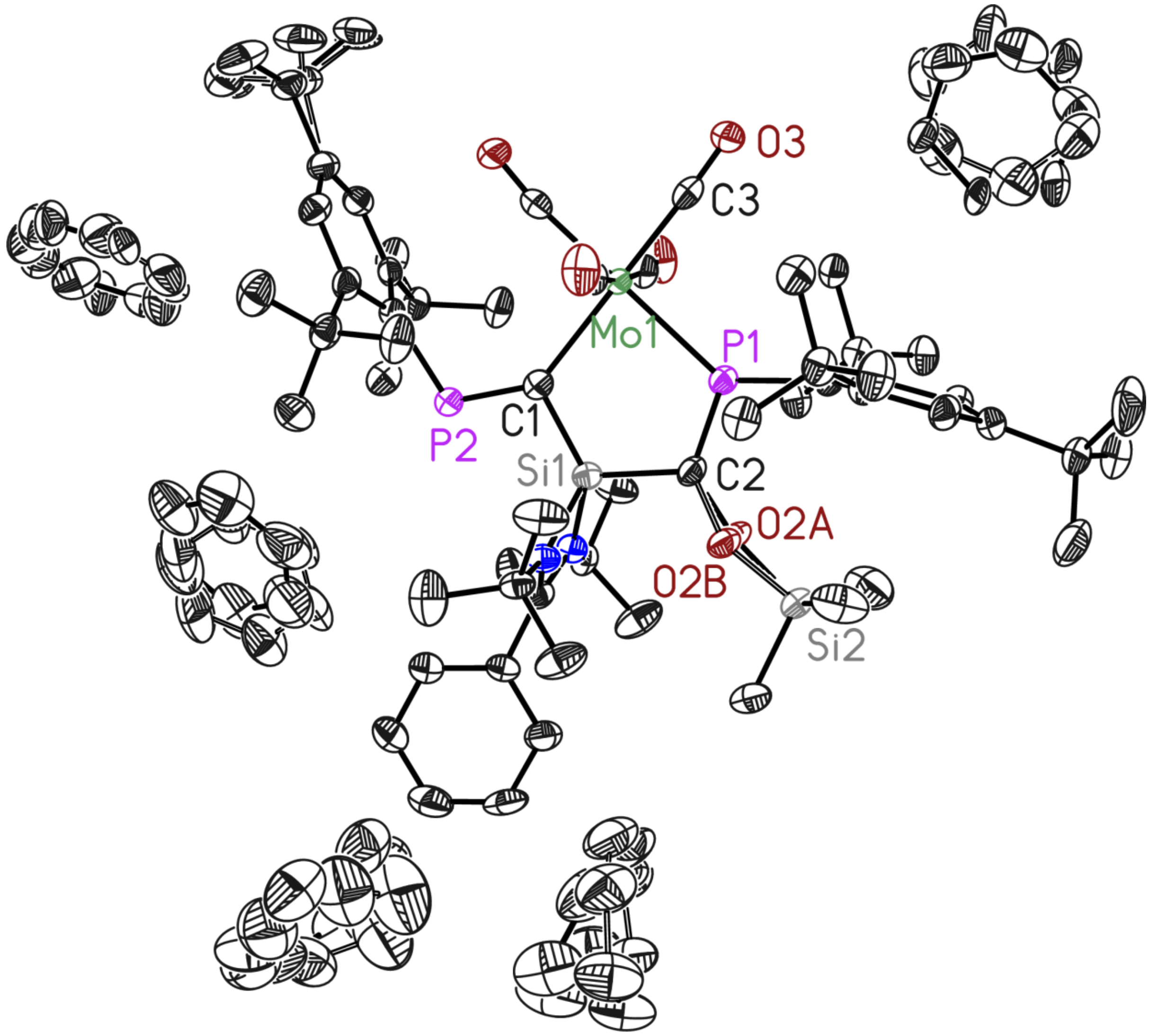

Figure S42: Asymmetric unit of **3** with thermal ellipsoids shown at 50% probability level. Hydrogen atoms are omitted for clarity. The structure was refined as an inversion twin with a fractional contribution of the minor component of 0.47(3). One *tert*-butyl group is disordered over two positions. The occupancy of the minor component refined to 0.286(16). The occupancy of the disordered oxygen atom refined to 0.50(4). For the five disordered benzene molecules the occupancies of the minor position refined to 0.264(13), 0.419(15), 0.42(2), 0.29(3) and 0.408(15). All disordered groups were refined with distance restraints and restraints for the anisotropic parameters. Selected bond lengths (Å) and bond angles (°). Mo1–P1 2.5051(11), Mo1 –C1 2.323(4), C1 –P2 1.679(4), C2-P1 1.678(4), C1–Si1 1.817(4), C2-Si1 1.868(4), C2-O2A 1.401(10), O2a–Si2 1.653(9), P1-Mo1-C1 86.24(10), C1-Si1-C2 115.66(18), P1-C2-Si1 112.7(2), Mo1-C1-Si1 111.93(19), Mo1-P1-C2 113.43(14).

Table S4. Bond length [Å] and angles [°] for **3.**

Mo(1)-C(3)	1.956(5)	C(27)-C(28)	1.391(6)
Mo(1)-C(5)	1.987(5)	C(28)-C(29)	1.380(6)
Mo(1)-C(6)	2.036(5)	C(28)-C(35)	1.536(6)
Mo(1)-C(4)	2.037(5)	C(29)-C(30)	1.397(6)
Mo(1)-C(1)	2.323(4)	C(30)-C(39)	1.553(6)
Mo(1)-P(1)	2.5051(11)	C(31)-C(32)	1.537(6)
P(2)-C(1)	1.679(4)	C(31)-C(33)	1.540(7)
P(2)-C(7)	1.884(4)	C(31)-C(34)	1.544(7)
C(7)-C(8)	1.424(6)	C(35)-C(37)	1.517(7)
C(7)-C(12)	1.430(6)	C(35)-C(36)	1.528(7)
C(8)-C(9)	1.397(6)	C(35)-C(38)	1.529(7)
C(8)-C(13)	1.559(7)	C(39)-C(42)	1.535(6)
C(9)-C(10)	1.387(6)	C(39)-C(41)	1.539(7)
C(10)-C(11)	1.382(6)	C(39)-C(40)	1.549(6)
C(10)-C(17B)	1.542(17)	Si(1)-C(1)	1.817(4)
C(10)-C(17A)	1.543(8)	Si(1)-N(1)	1.820(4)
C(11)-C(12)	1.398(6)	Si(1)-N(2)	1.821(4)
C(12)-C(21)	1.546(6)	Si(1)-C(2)	1.868(4)
C(13)-C(15)	1.529(6)	Si(1)-C(47)	2.278(4)
C(13)-C(16)	1.532(6)	N(2)-C(47)	1.338(5)
C(13)-C(14)	1.534(7)	N(2)-C(43)	1.489(5)
C(17A)-C(19A)	1.522(10)	C(43)-C(45)	1.508(7)
C(17A)-C(18A)	1.524(10)	C(43)-C(46)	1.513(6)
C(17A)-C(20A)	1.541(10)	C(43)-C(44)	1.517(6)
C(17B)-C(19B)	1.514(18)	C(47)-N(1)	1.343(5)
C(17B)-C(20B)	1.520(18)	C(47)-C(48)	1.475(5)
C(17B)-C(18B)	1.539(18)	C(48)-C(53)	1.386(6)
C(21)-C(24)	1.523(6)	C(48)-C(49)	1.394(6)
C(21)-C(23)	1.545(7)	C(49)-C(50)	1.386(6)
C(21)-C(22)	1.545(6)	C(50)-C(51)	1.360(7)
P(1)-C(2)	1.678(4)	C(51)-C(52)	1.387(7)
P(1)-C(25)	1.851(4)	C(52)-C(53)	1.381(6)
C(25)-C(30)	1.428(6)	N(1)-C(54)	1.505(5)
C(25)-C(26)	1.441(6)	C(54)-C(55)	1.511(7)
C(26)-C(27)	1.401(6)	C(54)-C(56)	1.513(7)
C(26)-C(31)	1.549(6)	C(54)-C(57)	1.535(6)

C(2)-O(2B)	1.384(10)	C(92C)-C(93C)	1.370(17)
C(2)-O(2A)	1.401(10)	C(93C)-C(94C)	1.381(16)
O(2A)-Si(2)	1.653(9)	C(94C)-C(95C)	1.411(16)
O(2B)-Si(2)	1.638(9)	C(90G)-C(91G)	1.368(13)
Si(2)-C(58)	1.820(6)	C(90G)-C(95G)	1.412(14)
Si(2)-C(59)	1.842(6)	C(91G)-C(92G)	1.369(14)
Si(2)-C(60)	1.858(5)	C(92G)-C(93G)	1.384(14)
O(3)-C(3)	1.176(5)	C(93G)-C(94G)	1.376(15)
O(4)-C(4)	1.146(5)	C(94G)-C(95G)	1.387(15)
O(5)-C(5)	1.151(5)	C(90D)-C(95D)	1.376(12)
O(6)-C(6)	1.158(5)	C(90D)-C(91D)	1.379(12)
C(90A)-C(95A)	1.349(11)	C(91D)-C(92D)	1.371(12)
C(90A)-C(91A)	1.372(12)	C(92D)-C(93D)	1.393(11)
C(91A)-C(92A)	1.365(12)	C(93D)-C(94D)	1.395(11)
C(92A)-C(93A)	1.415(13)	C(94D)-C(95D)	1.388(11)
C(93A)-C(94A)	1.377(11)	C(90I)-C(95I)	1.378(18)
C(94A)-C(95A)	1.370(11)	C(90I)-C(91I)	1.383(17)
C(90J)-C(91J)	1.376(17)	C(91I)-C(92I)	1.385(17)
C(90J)-C(95J)	1.390(18)	C(92I)-C(93I)	1.383(17)
C(91J)-C(92J)	1.362(18)	C(93I)-C(94I)	1.385(17)
C(92J)-C(93J)	1.359(18)	C(94I)-C(95I)	1.379(18)
C(93J)-C(94J)	1.366(17)	C(90E)-C(91E)	1.352(14)
C(94J)-C(95J)	1.387(17)	C(90E)-C(95E)	1.388(14)
C(90B)-C(95B)	1.363(15)	C(91E)-C(92E)	1.381(13)
C(90B)-C(91B)	1.379(14)	C(92E)-C(93E)	1.384(11)
C(91B)-C(92B)	1.397(14)	C(93E)-C(94E)	1.391(11)
C(92B)-C(93B)	1.404(15)	C(94E)-C(95E)	1.409(13)
C(93B)-C(94B)	1.392(13)	C(90F)-C(91F)	1.367(16)
C(94B)-C(95B)	1.372(15)	C(90F)-C(95F)	1.376(15)
C(90H)-C(95H)	1.366(17)	C(91F)-C(92F)	1.377(17)
C(90H)-C(91H)	1.382(16)	C(92F)-C(93F)	1.361(16)
C(91H)-C(92H)	1.369(17)	C(93F)-C(94F)	1.409(16)
C(92H)-C(93H)	1.384(17)	C(94F)-C(95F)	1.384(15)
C(93H)-C(94H)	1.373(17)		
C(94H)-C(95H)	1.369(17)	C(3)-Mo(1)-C(5)	86.03(17)
C(90C)-C(91C)	1.390(17)	C(3)-Mo(1)-C(6)	86.6(2)
C(90C)-C(95C)	1.395(17)	C(5)-Mo(1)-C(6)	88.9(2)
C(91C)-C(92C)	1.382(17)	C(3)-Mo(1)-C(4)	87.3(2)

C(5)-Mo(1)-C(4)	87.6(2)	C(18A)-C(17A)-C(10)	107.3(7)
C(6)-Mo(1)-C(4)	173.15(17)	C(20A)-C(17A)-C(10)	110.7(7)
C(3)-Mo(1)-C(1)	178.8(2)	C(19B)-C(17B)-C(20B)	108.5(17)
C(5)-Mo(1)-C(1)	93.86(15)	C(19B)-C(17B)-C(18B)	107.7(16)
C(6)-Mo(1)-C(1)	92.19(18)	C(20B)-C(17B)-C(18B)	109.6(19)
C(4)-Mo(1)-C(1)	93.90(18)	C(19B)-C(17B)-C(10)	110.7(15)
C(3)-Mo(1)-P(1)	93.89(12)	C(20B)-C(17B)-C(10)	107.3(16)
C(5)-Mo(1)-P(1)	179.51(17)	C(18B)-C(17B)-C(10)	113.0(16)
C(6)-Mo(1)-P(1)	91.58(14)	C(24)-C(21)-C(23)	111.7(4)
C(4)-Mo(1)-P(1)	91.94(13)	C(24)-C(21)-C(22)	106.7(4)
C(1)-Mo(1)-P(1)	86.24(10)	C(23)-C(21)-C(22)	105.0(4)
C(1)-P(2)-C(7)	111.28(19)	C(24)-C(21)-C(12)	111.3(4)
C(8)-C(7)-C(12)	117.9(4)	C(23)-C(21)-C(12)	109.6(4)
C(8)-C(7)-P(2)	121.7(3)	C(22)-C(21)-C(12)	112.5(4)
C(12)-C(7)-P(2)	120.4(3)	C(2)-P(1)-C(25)	109.22(19)
C(9)-C(8)-C(7)	118.6(4)	C(2)-P(1)-Mo(1)	113.43(14)
C(9)-C(8)-C(13)	117.2(4)	C(25)-P(1)-Mo(1)	137.35(13)
C(7)-C(8)-C(13)	124.1(4)	C(30)-C(25)-C(26)	119.0(3)
C(10)-C(9)-C(8)	123.4(4)	C(30)-C(25)-P(1)	120.4(3)
C(11)-C(10)-C(9)	116.5(4)	C(26)-C(25)-P(1)	120.4(3)
C(11)-C(10)-C(17B)	125.6(9)	C(27)-C(26)-C(25)	117.3(4)
C(9)-C(10)-C(17B)	117.7(9)	C(27)-C(26)-C(31)	117.8(4)
C(11)-C(10)-C(17A)	121.5(5)	C(25)-C(26)-C(31)	124.7(4)
C(9)-C(10)-C(17A)	121.8(5)	C(28)-C(27)-C(26)	123.7(4)
C(10)-C(11)-C(12)	123.6(4)	C(29)-C(28)-C(27)	116.8(4)
C(11)-C(12)-C(7)	118.4(4)	C(29)-C(28)-C(35)	122.9(4)
C(11)-C(12)-C(21)	117.2(4)	C(27)-C(28)-C(35)	120.3(4)
C(7)-C(12)-C(21)	124.3(4)	C(28)-C(29)-C(30)	123.7(5)
C(15)-C(13)-C(16)	106.8(4)	C(29)-C(30)-C(25)	118.1(4)
C(15)-C(13)-C(14)	110.5(4)	C(29)-C(30)-C(39)	117.4(4)
C(16)-C(13)-C(14)	106.1(4)	C(25)-C(30)-C(39)	124.3(4)
C(15)-C(13)-C(8)	111.4(4)	C(32)-C(31)-C(33)	106.1(4)
C(16)-C(13)-C(8)	112.1(4)	C(32)-C(31)-C(34)	106.4(4)
C(14)-C(13)-C(8)	109.8(4)	C(33)-C(31)-C(34)	109.7(4)
C(19A)-C(17A)-C(18A)	108.4(8)	C(32)-C(31)-C(26)	112.2(4)
C(19A)-C(17A)-C(20A)	109.1(7)	C(33)-C(31)-C(26)	114.3(4)
C(18A)-C(17A)-C(20A)	107.8(8)	C(34)-C(31)-C(26)	107.8(4)
C(19A)-C(17A)-C(10)	113.4(7)	C(37)-C(35)-C(36)	110.3(5)

C(37)-C(35)-C(38)	108.2(4)	C(49)-C(48)-C(47)	122.2(4)
C(36)-C(35)-C(38)	108.0(4)	C(50)-C(49)-C(48)	119.6(4)
C(37)-C(35)-C(28)	110.2(4)	C(51)-C(50)-C(49)	120.3(5)
C(36)-C(35)-C(28)	108.2(4)	C(50)-C(51)-C(52)	120.7(5)
C(38)-C(35)-C(28)	111.9(4)	C(53)-C(52)-C(51)	119.7(5)
C(42)-C(39)-C(41)	110.8(4)	C(52)-C(53)-C(48)	119.9(4)
C(42)-C(39)-C(40)	105.9(4)	C(47)-N(1)-C(54)	129.9(4)
C(41)-C(39)-C(40)	106.0(4)	C(47)-N(1)-Si(1)	90.8(2)
C(42)-C(39)-C(30)	113.5(4)	C(54)-N(1)-Si(1)	139.3(3)
C(41)-C(39)-C(30)	108.6(4)	N(1)-C(54)-C(55)	110.6(4)
C(40)-C(39)-C(30)	111.8(4)	N(1)-C(54)-C(56)	110.2(4)
C(1)-Si(1)-N(1)	121.51(19)	C(55)-C(54)-C(56)	113.1(4)
C(1)-Si(1)-N(2)	121.05(19)	N(1)-C(54)-C(57)	104.1(3)
N(1)-Si(1)-N(2)	72.07(14)	C(55)-C(54)-C(57)	109.7(4)
C(1)-Si(1)-C(2)	115.66(18)	C(56)-C(54)-C(57)	108.7(4)
N(1)-Si(1)-C(2)	109.54(19)	O(2B)-C(2)-P(1)	133.0(7)
N(2)-Si(1)-C(2)	109.48(19)	O(2A)-C(2)-P(1)	129.0(8)
C(1)-Si(1)-C(47)	128.95(17)	O(2B)-C(2)-Si(1)	113.0(6)
N(1)-Si(1)-C(47)	36.14(15)	O(2A)-C(2)-Si(1)	115.6(6)
N(2)-Si(1)-C(47)	35.96(15)	P(1)-C(2)-Si(1)	112.7(2)
C(2)-Si(1)-C(47)	115.39(17)	C(2)-O(2A)-Si(2)	146.5(15)
C(47)-N(2)-C(43)	130.0(4)	C(2)-O(2B)-Si(2)	150.8(13)
C(47)-N(2)-Si(1)	91.0(2)	O(2B)-Si(2)-C(58)	119.9(8)
C(43)-N(2)-Si(1)	139.0(3)	O(2A)-Si(2)-C(58)	99.6(11)
N(2)-C(43)-C(45)	111.2(4)	O(2B)-Si(2)-C(59)	102.6(10)
N(2)-C(43)-C(46)	111.1(4)	O(2A)-Si(2)-C(59)	120.4(7)
C(45)-C(43)-C(46)	110.3(4)	C(58)-Si(2)-C(59)	112.2(3)
N(2)-C(43)-C(44)	105.4(3)	O(2B)-Si(2)-C(60)	104.3(5)
C(45)-C(43)-C(44)	109.9(4)	O(2A)-Si(2)-C(60)	106.9(5)
C(46)-C(43)-C(44)	108.7(4)	C(58)-Si(2)-C(60)	108.8(3)
N(2)-C(47)-N(1)	106.0(3)	C(59)-Si(2)-C(60)	108.1(3)
N(2)-C(47)-C(48)	126.6(4)	O(3)-C(3)-Mo(1)	177.0(4)
N(1)-C(47)-C(48)	127.3(4)	O(4)-C(4)-Mo(1)	174.7(4)
N(2)-C(47)-Si(1)	53.1(2)	O(5)-C(5)-Mo(1)	173.0(4)
N(1)-C(47)-Si(1)	53.0(2)	O(6)-C(6)-Mo(1)	173.4(4)
C(48)-C(47)-Si(1)	175.2(3)	P(2)-C(1)-Si(1)	108.0(2)
C(53)-C(48)-C(49)	119.8(4)	P(2)-C(1)-Mo(1)	140.1(2)
C(53)-C(48)-C(47)	118.1(4)	Si(1)-C(1)-Mo(1)	111.93(19)

C(95A)-C(90A)-C(91A)	120.8(11)	C(91G)-C(90G)-C(95G)	119.9(13)
C(92A)-C(91A)-C(90A)	119.2(10)	C(90G)-C(91G)-C(92G)	121.4(13)
C(91A)-C(92A)-C(93A)	120.8(10)	C(91G)-C(92G)-C(93G)	119.7(14)
C(94A)-C(93A)-C(92A)	118.0(9)	C(94G)-C(93G)-C(92G)	119.6(14)
C(95A)-C(94A)-C(93A)	120.0(9)	C(93G)-C(94G)-C(95G)	121.5(14)
C(90A)-C(95A)-C(94A)	121.1(10)	C(94G)-C(95G)-C(90G)	117.8(13)
C(91J)-C(90J)-C(95J)	120(2)	C(95D)-C(90D)-C(91D)	120.7(10)
C(92J)-C(91J)-C(90J)	121(2)	C(92D)-C(91D)-C(90D)	121.2(10)
C(93J)-C(92J)-C(91J)	117(2)	C(91D)-C(92D)-C(93D)	119.1(9)
C(92J)-C(93J)-C(94J)	123(2)	C(92D)-C(93D)-C(94D)	119.5(9)
C(93J)-C(94J)-C(95J)	119.3(18)	C(95D)-C(94D)-C(93D)	120.8(9)
C(94J)-C(95J)-C(90J)	117.4(18)	C(90D)-C(95D)-C(94D)	118.7(10)
C(95B)-C(90B)-C(91B)	119.8(13)	C(95I)-C(90I)-C(91I)	121(2)
C(90B)-C(91B)-C(92B)	118.8(13)	C(90I)-C(91I)-C(92I)	119(2)
C(91B)-C(92B)-C(93B)	121.1(13)	C(93I)-C(92I)-C(91I)	119(2)
C(94B)-C(93B)-C(92B)	118.3(13)	C(92I)-C(93I)-C(94I)	122(2)
C(95B)-C(94B)-C(93B)	119.3(13)	C(95I)-C(94I)-C(93I)	118(2)
C(90B)-C(95B)-C(94B)	122.3(13)	C(90I)-C(95I)-C(94I)	120(2)
C(95H)-C(90H)-C(91H)	122.1(18)	C(91E)-C(90E)-C(95E)	120.1(12)
C(92H)-C(91H)-C(90H)	114.8(18)	C(90E)-C(91E)-C(92E)	121.3(13)
C(91H)-C(92H)-C(93H)	122.1(19)	C(91E)-C(92E)-C(93E)	119.3(11)
C(94H)-C(93H)-C(92H)	122.7(19)	C(92E)-C(93E)-C(94E)	121.0(10)
C(95H)-C(94H)-C(93H)	114.2(18)	C(93E)-C(94E)-C(95E)	117.9(11)
C(90H)-C(95H)-C(94H)	122.9(19)	C(90E)-C(95E)-C(94E)	120.3(12)
C(91C)-C(90C)-C(95C)	117.6(18)	C(91F)-C(90F)-C(95F)	119.8(17)
C(92C)-C(91C)-C(90C)	121.7(19)	C(90F)-C(91F)-C(92F)	120.5(18)
C(93C)-C(92C)-C(91C)	121.4(19)	C(93F)-C(92F)-C(91F)	120.8(18)
C(92C)-C(93C)-C(94C)	118.2(18)	C(92F)-C(93F)-C(94F)	119.1(16)
C(93C)-C(94C)-C(95C)	121.2(17)	C(95F)-C(94F)-C(93F)	119.4(16)
C(90C)-C(95C)-C(94C)	119.9(17)	C(90F)-C(95F)-C(94F)	120.2(16)

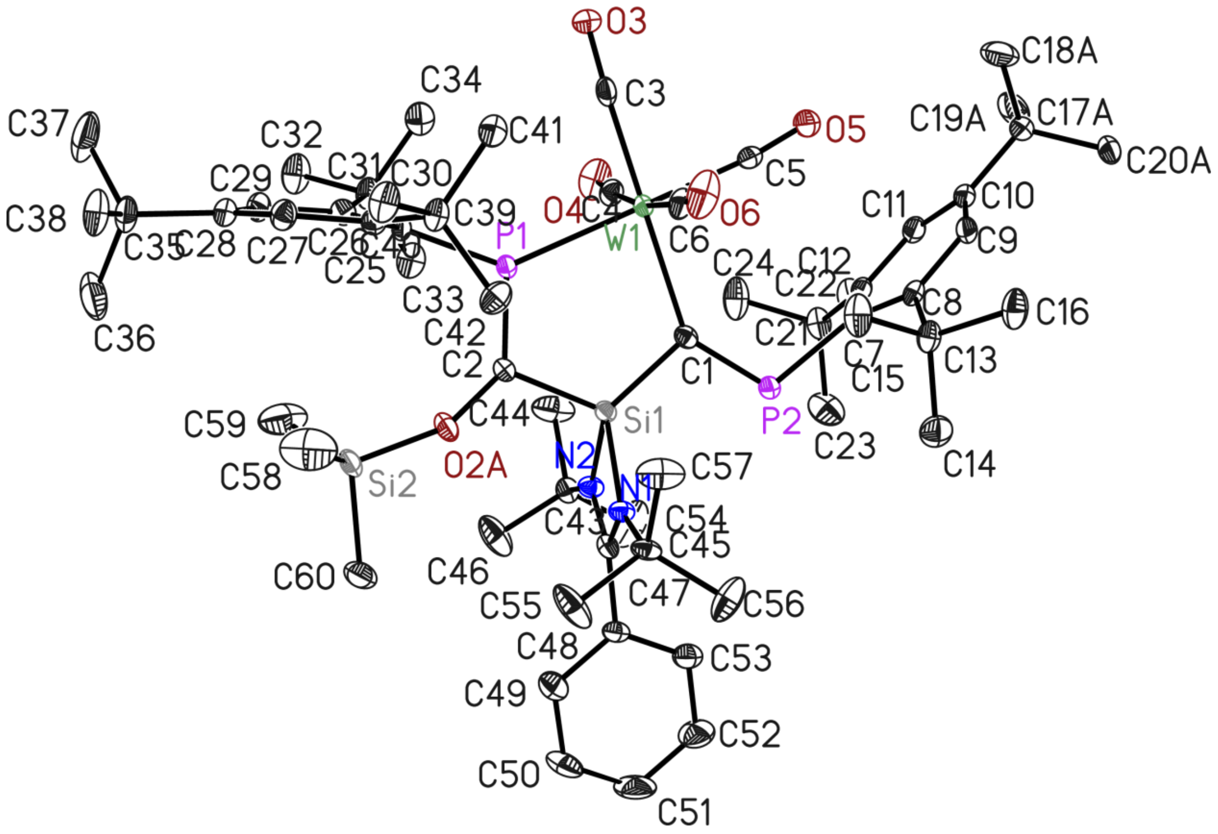

Figure S43: Molecular structure of **4** with thermal ellipsoids shown at 50% probability level. Hydrogen atoms are omitted for clarity.

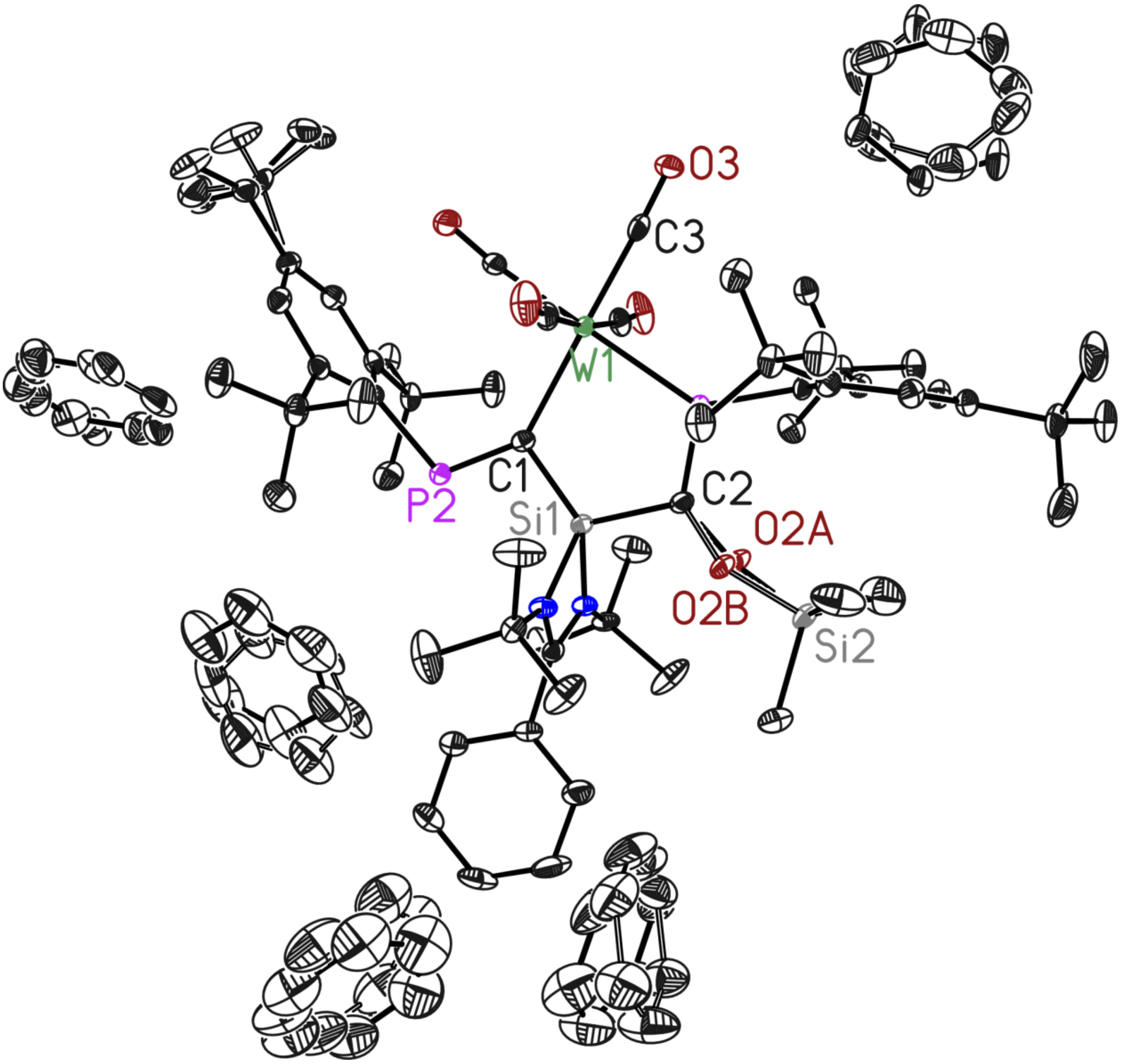

Figure S44: Asymmetric unit of **4** with thermal ellipsoids shown at 50% probability level. Hydrogen atoms are omitted for clarity. One *tert*-butyl group is disordered over two positions. The occupancy of the minor component refined to 0.271(12). The occupancy of the minor component of the disordered oxygen atom refined to 0.44(4). For the five disordered benzene molecules the occupancies of the minor position refined to 0.251(9), 0.339(8), 0.439(17), 0.47(3) and 0.376(13). All disordered groups were refined with distance restraints and restraints for the anisotropic parameters. Selected bond lengths (Å) and bond angles (°). W1–P1 2.4933(7), W1–C1 2.294(2), C1–P2 1.690(3), C2-P1 1.686(3), C1–Si1 1.821(2), C2-Si1 1.861(3), C2-O2A 1.389(9), O2A–Si2 1.657(8), P1-W1-C1 86.12(6), C1-Si1-C2 115.01(11), P1-C2-Si1 112.28(13), W1-C1-Si1 112.86(12), W1-P1-C2 113.65(9).

Table S5. Bond length [Å] and angles [°] for **4.**

W(1)-C(3)	1.968(3)	C(27)-C(28)	1.386(4)
W(1)-C(5)	1.995(3)	C(28)-C(29)	1.380(4)
W(1)-C(4)	2.029(3)	C(28)-C(35)	1.535(4)
W(1)-C(6)	2.039(3)	C(29)-C(30)	1.401(4)
W(1)-C(1)	2.294(2)	C(30)-C(39)	1.554(4)
W(1)-P(1)	2.4933(7)	C(31)-C(34)	1.536(4)
P(2)-C(1)	1.690(3)	C(31)-C(33)	1.537(4)
P(2)-C(7)	1.881(2)	C(31)-C(32)	1.545(4)
C(7)-C(12)	1.428(4)	C(35)-C(36)	1.525(5)
C(7)-C(8)	1.430(4)	C(35)-C(38)	1.527(5)
C(8)-C(9)	1.402(4)	C(35)-C(37)	1.530(5)
C(8)-C(13)	1.546(4)	C(39)-C(42)	1.537(4)
C(9)-C(10)	1.388(4)	C(39)-C(41)	1.540(4)
C(10)-C(11)	1.380(4)	C(39)-C(40)	1.544(4)
C(10)-C(17A)	1.544(7)	Si(1)-N(1)	1.820(2)
C(10)-C(17B)	1.568(16)	Si(1)-N(2)	1.821(2)
C(11)-C(12)	1.398(4)	Si(1)-C(1)	1.821(2)
C(12)-C(21)	1.549(4)	Si(1)-C(2)	1.861(3)
C(13)-C(14)	1.535(4)	Si(1)-C(47)	2.278(2)
C(13)-C(15)	1.539(4)	N(2)-C(47)	1.343(3)
C(13)-C(16)	1.542(4)	N(2)-C(43)	1.488(3)
C(17A)-C(19A)	1.526(8)	C(43)-C(46)	1.509(4)
C(17A)-C(18A)	1.535(8)	C(43)-C(44)	1.511(4)
C(17A)-C(20A)	1.536(7)	C(43)-C(45)	1.522(5)
C(17B)-C(19B)	1.513(17)	C(47)-N(1)	1.343(3)
C(17B)-C(20B)	1.525(17)	C(47)-C(48)	1.479(3)
C(17B)-C(18B)	1.543(16)	C(48)-C(53)	1.385(4)
C(21)-C(23)	1.534(5)	C(48)-C(49)	1.387(4)
C(21)-C(24)	1.535(4)	C(49)-C(50)	1.391(4)
C(21)-C(22)	1.538(4)	C(50)-C(51)	1.361(5)
P(1)-C(2)	1.686(3)	C(51)-C(52)	1.386(5)
P(1)-C(25)	1.852(3)	C(52)-C(53)	1.390(4)
C(25)-C(30)	1.426(4)	N(1)-C(54)	1.485(3)
C(25)-C(26)	1.434(4)	C(54)-C(56)	1.514(4)
C(26)-C(27)	1.399(4)	C(54)-C(57)	1.522(4)
C(26)-C(31)	1.557(4)	C(54)-C(55)	1.540(4)

C(2)-O(2B)	1.375(6)	C(92C)-C(93C)	1.353(15)
C(2)-O(2A)	1.389(9)	C(93C)-C(94C)	1.380(13)
O(2A)-Si(2)	1.657(8)	C(94C)-C(95C)	1.409(14)
O(2B)-Si(2)	1.644(6)	C(90G)-C(91G)	1.372(12)
Si(2)-C(58)	1.835(4)	C(90G)-C(95G)	1.395(12)
Si(2)-C(59)	1.844(4)	C(91G)-C(92G)	1.359(13)
Si(2)-C(60)	1.856(3)	C(92G)-C(93G)	1.371(12)
O(3)-C(3)	1.172(3)	C(93G)-C(94G)	1.390(13)
O(4)-C(4)	1.151(3)	C(94G)-C(95G)	1.373(14)
O(5)-C(5)	1.144(3)	C(90D)-C(95D)	1.383(13)
O(6)-C(6)	1.151(3)	C(90D)-C(91D)	1.396(12)
C(90A)-C(91A)	1.353(9)	C(91D)-C(92D)	1.370(11)
C(90A)-C(95A)	1.361(9)	C(92D)-C(93D)	1.378(11)
C(91A)-C(92A)	1.378(10)	C(93D)-C(94D)	1.379(12)
C(92A)-C(93A)	1.401(11)	C(94D)-C(95D)	1.384(13)
C(93A)-C(94A)	1.369(9)	C(90I)-C(95I)	1.384(14)
C(94A)-C(95A)	1.371(8)	C(90I)-C(91I)	1.396(12)
C(90J)-C(91J)	1.364(16)	C(91I)-C(92I)	1.367(13)
C(90J)-C(95J)	1.399(17)	C(92I)-C(93I)	1.393(13)
C(91J)-C(92J)	1.331(17)	C(93I)-C(94I)	1.372(14)
C(92J)-C(93J)	1.356(17)	C(94I)-C(95I)	1.374(15)
C(93J)-C(94J)	1.367(15)	C(90E)-C(91E)	1.367(12)
C(94J)-C(95J)	1.374(15)	C(90E)-C(95E)	1.373(12)
C(90B)-C(95B)	1.358(15)	C(91E)-C(92E)	1.384(11)
C(90B)-C(91B)	1.388(15)	C(92E)-C(93E)	1.380(8)
C(91B)-C(92B)	1.366(16)	C(93E)-C(94E)	1.384(8)
C(92B)-C(93B)	1.358(16)	C(94E)-C(95E)	1.400(9)
C(93B)-C(94B)	1.385(16)	C(90F)-C(91F)	1.372(14)
C(94B)-C(95B)	1.356(16)	C(90F)-C(95F)	1.378(11)
C(90H)-C(91H)	1.359(11)	C(91F)-C(92F)	1.365(16)
C(90H)-C(95H)	1.378(11)	C(92F)-C(93F)	1.392(15)
C(91H)-C(92H)	1.409(12)	C(93F)-C(94F)	1.382(15)
C(92H)-C(93H)	1.394(11)	C(94F)-C(95F)	1.379(13)
C(93H)-C(94H)	1.377(10)		
C(94H)-C(95H)	1.353(11)	C(3)-W(1)-C(5)	86.02(10)
C(90C)-C(95C)	1.382(15)	C(3)-W(1)-C(4)	87.38(12)
C(90C)-C(91C)	1.384(15)	C(5)-W(1)-C(4)	87.72(13)
C(91C)-C(92C)	1.366(15)	C(3)-W(1)-C(6)	86.10(12)

C(5)-W(1)-C(6)	88.45(14)	C(18A)-C(17A)-C(10)	106.4(5)
C(4)-W(1)-C(6)	172.65(10)	C(20A)-C(17A)-C(10)	110.8(5)
C(3)-W(1)-C(1)	178.69(12)	C(19B)-C(17B)-C(20B)	108.7(15)
C(5)-W(1)-C(1)	94.23(9)	C(19B)-C(17B)-C(18B)	109.1(14)
C(4)-W(1)-C(1)	93.91(11)	C(20B)-C(17B)-C(18B)	107.2(17)
C(6)-W(1)-C(1)	92.62(11)	C(19B)-C(17B)-C(10)	111.2(12)
C(3)-W(1)-P(1)	93.65(7)	C(20B)-C(17B)-C(10)	106.8(14)
C(5)-W(1)-P(1)	179.38(10)	C(18B)-C(17B)-C(10)	113.7(13)
C(4)-W(1)-P(1)	91.74(8)	C(23)-C(21)-C(24)	111.9(3)
C(6)-W(1)-P(1)	92.06(9)	C(23)-C(21)-C(22)	105.0(3)
C(1)-W(1)-P(1)	86.12(6)	C(24)-C(21)-C(22)	106.3(3)
C(1)-P(2)-C(7)	111.17(11)	C(23)-C(21)-C(12)	110.5(3)
C(12)-C(7)-C(8)	118.1(2)	C(24)-C(21)-C(12)	110.5(3)
C(12)-C(7)-P(2)	120.6(2)	C(22)-C(21)-C(12)	112.6(3)
C(8)-C(7)-P(2)	121.3(2)	C(2)-P(1)-C(25)	109.58(12)
C(9)-C(8)-C(7)	118.3(3)	C(2)-P(1)-W(1)	113.65(9)
C(9)-C(8)-C(13)	117.6(3)	C(25)-P(1)-W(1)	136.77(8)
C(7)-C(8)-C(13)	124.0(3)	C(30)-C(25)-C(26)	119.0(2)
C(10)-C(9)-C(8)	123.4(3)	C(30)-C(25)-P(1)	120.2(2)
C(11)-C(10)-C(9)	116.7(2)	C(26)-C(25)-P(1)	120.5(2)
C(11)-C(10)-C(17A)	121.5(4)	C(27)-C(26)-C(25)	117.8(3)
C(9)-C(10)-C(17A)	121.5(4)	C(27)-C(26)-C(31)	117.6(3)
C(11)-C(10)-C(17B)	126.0(7)	C(25)-C(26)-C(31)	124.3(3)
C(9)-C(10)-C(17B)	116.8(7)	C(28)-C(27)-C(26)	123.3(3)
C(10)-C(11)-C(12)	123.5(3)	C(29)-C(28)-C(27)	117.1(2)
C(11)-C(12)-C(7)	118.6(3)	C(29)-C(28)-C(35)	122.6(3)
C(11)-C(12)-C(21)	117.2(3)	C(27)-C(28)-C(35)	120.3(3)
C(7)-C(12)-C(21)	124.2(3)	C(28)-C(29)-C(30)	123.4(3)
C(14)-C(13)-C(15)	111.2(3)	C(29)-C(30)-C(25)	117.9(3)
C(14)-C(13)-C(16)	105.7(3)	C(29)-C(30)-C(39)	117.0(3)
C(15)-C(13)-C(16)	106.2(2)	C(25)-C(30)-C(39)	124.9(3)
C(14)-C(13)-C(8)	110.3(2)	C(34)-C(31)-C(33)	109.8(3)
C(15)-C(13)-C(8)	111.4(2)	C(34)-C(31)-C(32)	106.2(3)
C(16)-C(13)-C(8)	111.9(2)	C(33)-C(31)-C(32)	106.1(3)
C(19A)-C(17A)-C(18A)	108.1(5)	C(34)-C(31)-C(26)	108.1(2)
C(19A)-C(17A)-C(20A)	109.3(5)	C(33)-C(31)-C(26)	114.3(3)
C(18A)-C(17A)-C(20A)	108.9(6)	C(32)-C(31)-C(26)	112.0(3)
C(19A)-C(17A)-C(10)	113.2(5)	C(36)-C(35)-C(38)	108.4(3)

C(36)-C(35)-C(37)	109.9(3)	C(49)-C(48)-C(47)	121.6(3)
C(38)-C(35)-C(37)	107.9(3)	C(48)-C(49)-C(50)	119.1(3)
C(36)-C(35)-C(28)	108.5(3)	C(51)-C(50)-C(49)	121.0(3)
C(38)-C(35)-C(28)	112.1(2)	C(50)-C(51)-C(52)	120.1(3)
C(37)-C(35)-C(28)	110.1(3)	C(51)-C(52)-C(53)	119.9(3)
C(42)-C(39)-C(41)	110.9(2)	C(48)-C(53)-C(52)	119.6(3)
C(42)-C(39)-C(40)	105.9(2)	C(47)-N(1)-C(54)	130.2(2)
C(41)-C(39)-C(40)	106.2(2)	C(47)-N(1)-Si(1)	90.85(15)
C(42)-C(39)-C(30)	113.4(2)	C(54)-N(1)-Si(1)	138.84(17)
C(41)-C(39)-C(30)	108.0(2)	N(1)-C(54)-C(56)	111.4(3)
C(40)-C(39)-C(30)	112.2(2)	N(1)-C(54)-C(57)	105.6(2)
N(1)-Si(1)-N(2)	72.22(9)	C(56)-C(54)-C(57)	109.2(3)
N(1)-Si(1)-C(1)	122.09(12)	N(1)-C(54)-C(55)	109.9(2)
N(2)-Si(1)-C(1)	121.65(12)	C(56)-C(54)-C(55)	111.7(3)
N(1)-Si(1)-C(2)	109.20(12)	C(57)-C(54)-C(55)	108.8(3)
N(2)-Si(1)-C(2)	109.45(13)	O(2B)-C(2)-P(1)	132.8(5)
C(1)-Si(1)-C(2)	115.01(11)	O(2A)-C(2)-P(1)	128.8(8)
N(1)-Si(1)-C(47)	36.12(9)	O(2B)-C(2)-Si(1)	113.8(4)
N(2)-Si(1)-C(47)	36.12(9)	O(2A)-C(2)-Si(1)	116.1(6)
C(1)-Si(1)-C(47)	130.08(11)	P(1)-C(2)-Si(1)	112.28(13)
C(2)-Si(1)-C(47)	114.91(10)	C(2)-O(2A)-Si(2)	147.4(13)
C(47)-N(2)-C(43)	130.4(2)	C(2)-O(2B)-Si(2)	151.1(8)
C(47)-N(2)-Si(1)	90.83(15)	O(2B)-Si(2)-C(58)	118.7(6)
C(43)-N(2)-Si(1)	138.71(17)	O(2A)-Si(2)-C(58)	99.2(10)
N(2)-C(43)-C(46)	111.2(2)	O(2B)-Si(2)-C(59)	104.0(8)
N(2)-C(43)-C(44)	105.9(2)	O(2A)-Si(2)-C(59)	121.0(6)
C(46)-C(43)-C(44)	109.2(3)	C(58)-Si(2)-C(59)	112.0(2)
N(2)-C(43)-C(45)	110.9(3)	O(2B)-Si(2)-C(60)	104.6(3)
C(46)-C(43)-C(45)	109.6(3)	O(2A)-Si(2)-C(60)	107.0(5)
C(44)-C(43)-C(45)	110.0(3)	C(58)-Si(2)-C(60)	108.52(19)
N(2)-C(47)-N(1)	106.1(2)	C(59)-Si(2)-C(60)	108.29(17)
N(2)-C(47)-C(48)	126.5(2)	O(3)-C(3)-W(1)	177.5(2)
N(1)-C(47)-C(48)	127.3(2)	O(4)-C(4)-W(1)	174.6(3)
N(2)-C(47)-Si(1)	53.05(12)	O(5)-C(5)-W(1)	173.8(2)
N(1)-C(47)-Si(1)	53.03(12)	O(6)-C(6)-W(1)	173.9(2)
C(48)-C(47)-Si(1)	175.21(19)	P(2)-C(1)-Si(1)	107.05(13)
C(53)-C(48)-C(49)	120.3(2)	P(2)-C(1)-W(1)	140.09(13)
C(53)-C(48)-C(47)	118.1(2)	Si(1)-C(1)-W(1)	112.86(12)

C(91A)-C(90A)-C(95A)	121.2(8)	C(91G)-C(90G)-C(95G)	118.7(11)
C(90A)-C(91A)-C(92A)	119.2(8)	C(92G)-C(91G)-C(90G)	120.3(10)
C(91A)-C(92A)-C(93A)	120.0(7)	C(91G)-C(92G)-C(93G)	121.3(11)
C(94A)-C(93A)-C(92A)	119.5(7)	C(92G)-C(93G)-C(94G)	119.8(11)
C(93A)-C(94A)-C(95A)	119.3(6)	C(95G)-C(94G)-C(93G)	118.7(10)
C(90A)-C(95A)-C(94A)	120.8(7)	C(94G)-C(95G)-C(90G)	121.2(10)
C(91J)-C(90J)-C(95J)	119.4(18)	C(95D)-C(90D)-C(91D)	121.4(12)
C(92J)-C(91J)-C(90J)	122.6(19)	C(92D)-C(91D)-C(90D)	118.4(11)
C(91J)-C(92J)-C(93J)	119.2(19)	C(91D)-C(92D)-C(93D)	120.8(11)
C(92J)-C(93J)-C(94J)	120.1(16)	C(92D)-C(93D)-C(94D)	120.5(12)
C(93J)-C(94J)-C(95J)	121.6(15)	C(93D)-C(94D)-C(95D)	119.8(14)
C(94J)-C(95J)-C(90J)	117.0(15)	C(90D)-C(95D)-C(94D)	119.0(14)
C(95B)-C(90B)-C(91B)	123.5(16)	C(95I)-C(90I)-C(91I)	120.3(15)
C(92B)-C(91B)-C(90B)	114.7(15)	C(92I)-C(91I)-C(90I)	119.2(13)
C(93B)-C(92B)-C(91B)	119.5(17)	C(91I)-C(92I)-C(93I)	120.3(13)
C(92B)-C(93B)-C(94B)	127.5(17)	C(94I)-C(93I)-C(92I)	120.1(14)
C(95B)-C(94B)-C(93B)	111.3(16)	C(93I)-C(94I)-C(95I)	120.2(16)
C(94B)-C(95B)-C(90B)	123.6(16)	C(94I)-C(95I)-C(90I)	119.7(16)
C(91H)-C(90H)-C(95H)	119.3(9)	C(91E)-C(90E)-C(95E)	121.1(8)
C(90H)-C(91H)-C(92H)	119.3(9)	C(90E)-C(91E)-C(92E)	120.4(9)
C(93H)-C(92H)-C(91H)	120.3(9)	C(93E)-C(92E)-C(91E)	119.1(9)
C(94H)-C(93H)-C(92H)	118.8(9)	C(92E)-C(93E)-C(94E)	121.0(7)
C(95H)-C(94H)-C(93H)	119.8(9)	C(93E)-C(94E)-C(95E)	119.1(7)
C(94H)-C(95H)-C(90H)	122.4(9)	C(90E)-C(95E)-C(94E)	119.4(7)
C(95C)-C(90C)-C(91C)	118.7(14)	C(91F)-C(90F)-C(95F)	120.8(13)
C(92C)-C(91C)-C(90C)	120.8(15)	C(92F)-C(91F)-C(90F)	121.1(15)
C(93C)-C(92C)-C(91C)	121.7(15)	C(91F)-C(92F)-C(93F)	119.1(16)
C(92C)-C(93C)-C(94C)	118.8(14)	C(94F)-C(93F)-C(92F)	119.3(13)
C(93C)-C(94C)-C(95C)	120.5(13)	C(95F)-C(94F)-C(93F)	121.4(12)
C(90C)-C(95C)-C(94C)	119.3(13)	C(90F)-C(95F)-C(94F)	118.2(12)

Computational Details

To understand the detailed atomic-level mechanisms for the formation of products **2'**, **3'**, and **4'** from the reaction of **1'** with $Fe(CO)_5$, $Mo(CO)_6$, and $W(CO)_6$, respectively, free energy profiles were mapped at the B3LYP-D3/6-31G* Mo, W (LanL2DZ) level of theory.[12-18] The B3LYP-D3 method has been shown in the literature to provide reliable results for understanding the mechanisms of similar systems.[19] To include the effect of the solvent, all the calculations were performed using the solvent toluene within the polarizable continuum model (PCM). [20] The thermodynamic properties for the reaction of 1' with $Fe(CO)_5$ were calculated at the room (experimental) temperature of 273.15 K. For the reaction of 1' with $Mo(CO)_6$ and $W(CO)_6$, all the thermodynamic properties were calculated at 333.15 K (experimental temperature). All the DFT calculations were performed using the Gaussian 16 software package. [21]

The free energy profile for the reaction of **1'** with $Fe(CO)_5$ is shown in (Figure S45). As can be seen from (Figure S45 (b)), the HOMO (-4.11 eV) molecular orbital of **1** corresponds to a Si=P π-type orbital, while the LUMO (-0.83 eV) is a π*-type orbital of the phenyl ring of the benzamidinate ligand. NBO analysis of **1** reveals that the HOMO orbital has a predominant electron density at the phosphorus atom, which is available for bond formation with incoming $Fe(CO)_5$. Initially, **1'** and $Fe(CO)_5$ combine to form the phosphasilene-iron complex, **Fe-int1**, releasing one CO molecule.

The CO molecule released from the previous step can be inserted into the P-Si bond of **Fe-int1** through the transition state **Fe-ts1'**, producing **Fe-int2'**. However, this process requires a high energy barrier of 66.88 kcal/mol, and hence is unfeasible under the experimental condition. An alternative scenario, in which **Fe-int1** reacts with a second **1'** molecule to generate **Fe-int2** while releasing another CO molecule, has been studied. It is worth noting that the *in situ* monitoring of the reaction by ^{31}P NMR spectroscopy showed the formation of Fe complex **2** together with a new peak at δ 200 ppm, which can be attributed to **Fe-int2** (Figure S38). The large downfield shift in the ^{31}P NMR resonance signal compared to phosphasilene **1** might be due to the coordination of P lone pair to the $Fe(CO)_3$ moiety which results in reduced electron density on the phosphorus atom. A similar trend is observed in the ^{31}P NMR spectrum for the M(0) complexes of phosphasilenes, further suggesting **Fe-int2** formation.

The two CO molecules released from the previous steps can then interact with the two Si atoms, Si(3) and Si(7) of **Fe-int2**, to form **Fe-int3**. One of the CO molecules forms a bond with Si(7) by overlapping the empty valence orbital of the silylium moiety with a π* orbital of the CO group, while the other CO molecule has a weak interaction with Si(3) at a distance of 3.5 Å due to steric crowding. During the Si(7)-C(3) bond formation, one of the C-N bonds of the amidinate group is stretched from 1.85 Å in **Fe-int2** to 2.03 Å in **Fe-int3**. The Si(7)-C(8) and Si(3)-C(2) distances for **Fe-int3** are 2.05 Å and 3.48 Å, respectively. The complex **Fe-int3** acts as the activated species from which the deoxygenative homo-coupling of two CO molecules happens as seen in the experiments. The energetics of the reaction are thus monitored with respect to **Fe-int3**. The activated species **Fe-int3** undergoes a cyclization step where the

overlap between the sp^2 hybridized orbitals of C(2) and C(8) results in the C-C bond formation (Figure **5**(c)) *via* the transition state **Fe-ts1**, forming a 7-membered ring structure **Fe-int4**. The energy barrier for this cyclization was determined to be 10.44 kcal/mol, and **Fe-int4** was stabilized by 10.13 kcal/mol relative to **Fe-int3**. In the subsequent step, the rearrangement of the CO group associated with the C(2) atom leads to the formation of a ketene moiety, **Fe-int5** (-19.83 kcal/mol), *via* the transition state **Fe-ts2**, with an energy barrier of 12.71 kcal/mol. This rearrangement is facilitated by the formation of a Si(3)-O(9) bond involving the oxygen atom attached to C(8), accompanied by the breaking of the Si(3)-C(2) bond. In the following step, the ketene moiety facilitates the formation of a strained four-membered ring through the formation of the P(6)-C(2) bond *via* **Fe-ts3** with an energy of -10.07 kcal/mol, leading to the intermediate **Fe-int6**. In the next step, this strain is relieved by breaking the P(6)-C(2), P(6)-Si(7), and C(8)-O(9) bonds, along with the simultaneous formation of the C(8)-P(6) bond to form **Fe-int7** *via* **Fe-ts4**. This process involves the insertion of the ketene group (C(8)=C(2)=O(1)) into the P(6)-Si(7) bond and the opening of the seven-membered ring. The energies of **Fe-ts4** and **Fe-int7** are 18.04 and -29.05 kcal/mol, respectively. In the subsequent step, the formation of Fe(5)-C(2) and Si(7)-O(1) bond happens *via* a bicyclic transition state **Fe-ts5**, having an energy of -9.53 kcal/mol and results in the four-membered cyclic structure **Fe-int8**. NBO calculations reveal that the interaction between P(4)-Fe(5) π^* and C(2)-O(1) π^* orbitals and between C(8)-Si(7) σ and C(2)-O(1) π^* orbitals favour this rearrangement. Finally, from **Fe-int8**, the elimination of silanone (**S**) which can undergoes the polymerization, occurs *via* the breaking of the P(4)-Si(3) bond, resulting in the final product **2'** through the ring closure transition state **Fe-ts6**, with an energy of -9.76 kcal/mol. Product **2'** is stabilized by 47.9 kcal/mol compared to activated species **Fe-int3.**Using the energy-span model,**46** the free energy profile indicates that the reaction from **int3** involves an overall barrier of δE = 18.04 kcal/mol associated with **Fe-ts4** and is the rate determining transition state for the deoxygenative homo-coupling of two CO molecules.

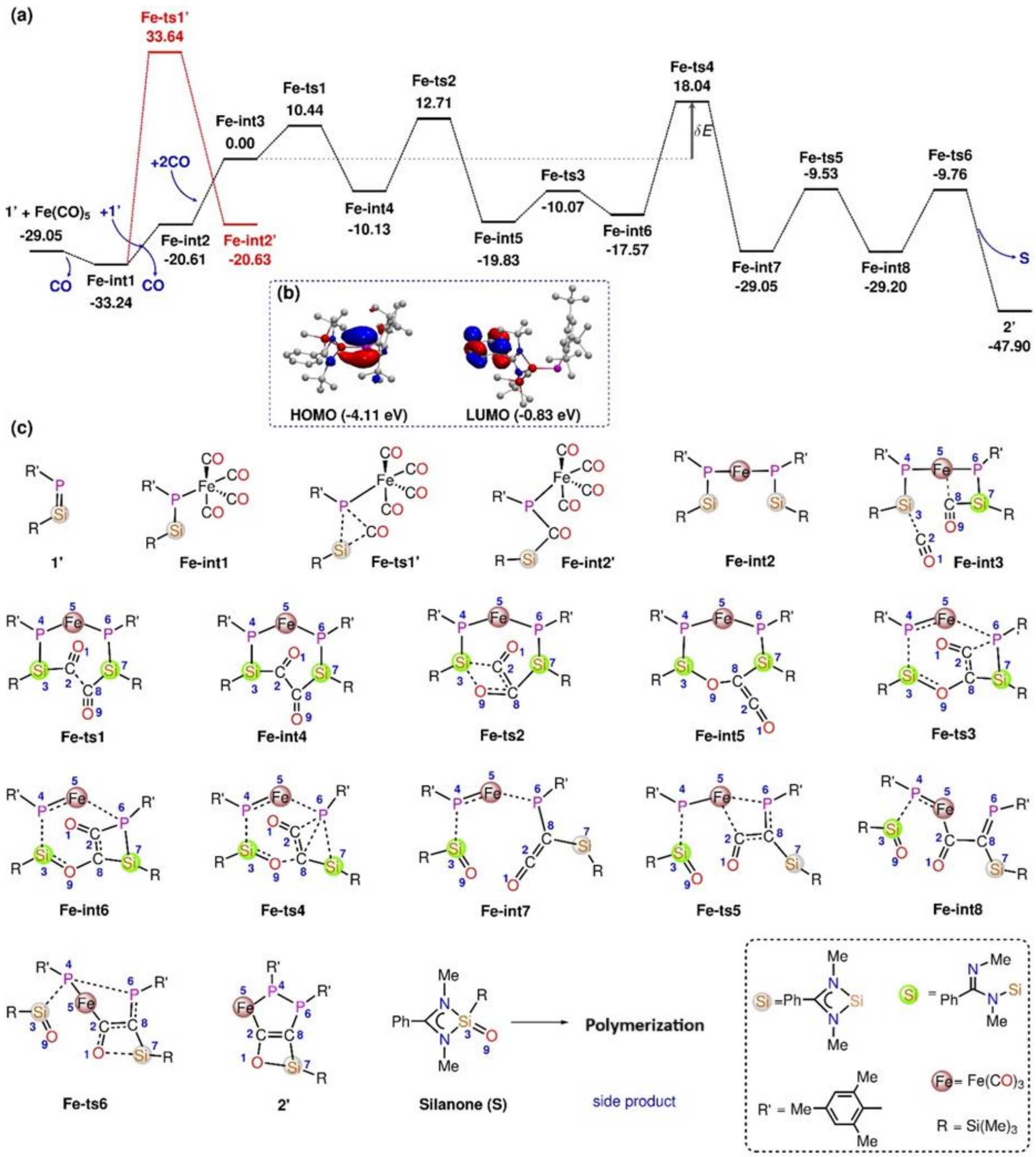

Figure. 45. (a) The Gibbs free energy profile for the reaction of 1' with $Fe(CO)_5$ was obtained at the B3LYP-D3/6-31G* level of theory. (b) Plot of the HOMO and LUMO orbitals and their corresponding energies of 1. (c) Different stationary point structures existing along the reaction pathway. The substituents connected to Si, Fe, and P are omitted in the images for clarity. The energies are given in kcal/mol

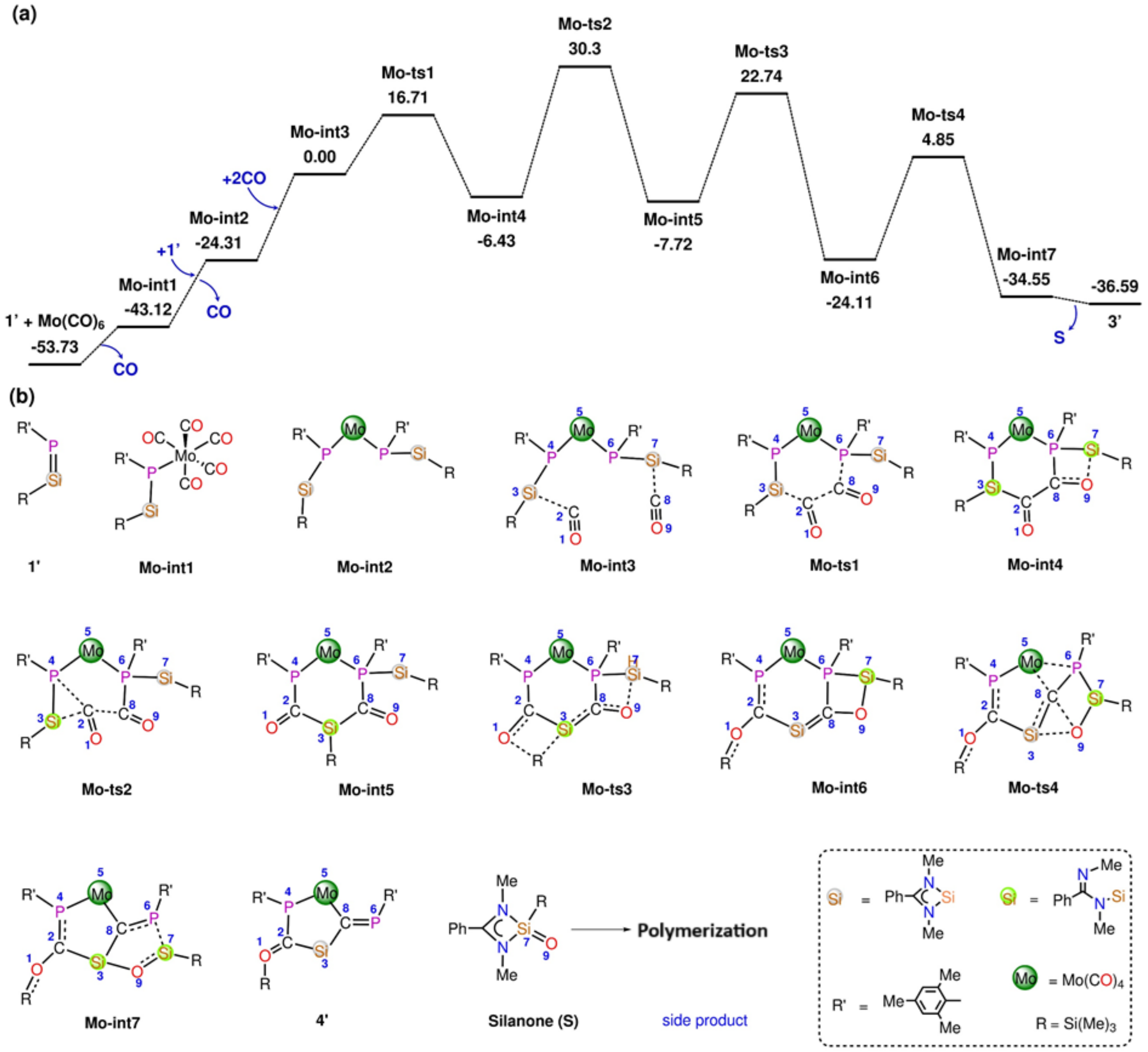

Figure S46. (a) The Gibbs free energy profile for the reaction of 1' with $Mo(CO)_6$ obtained at the B3LYP-D3/6-31G* level of theory. (b) Different stationary point structures existing along the reaction pathway. The substituents connected to Si, Mo, and P, are omitted in the images for clarity. The energies are given in kcal/mol.

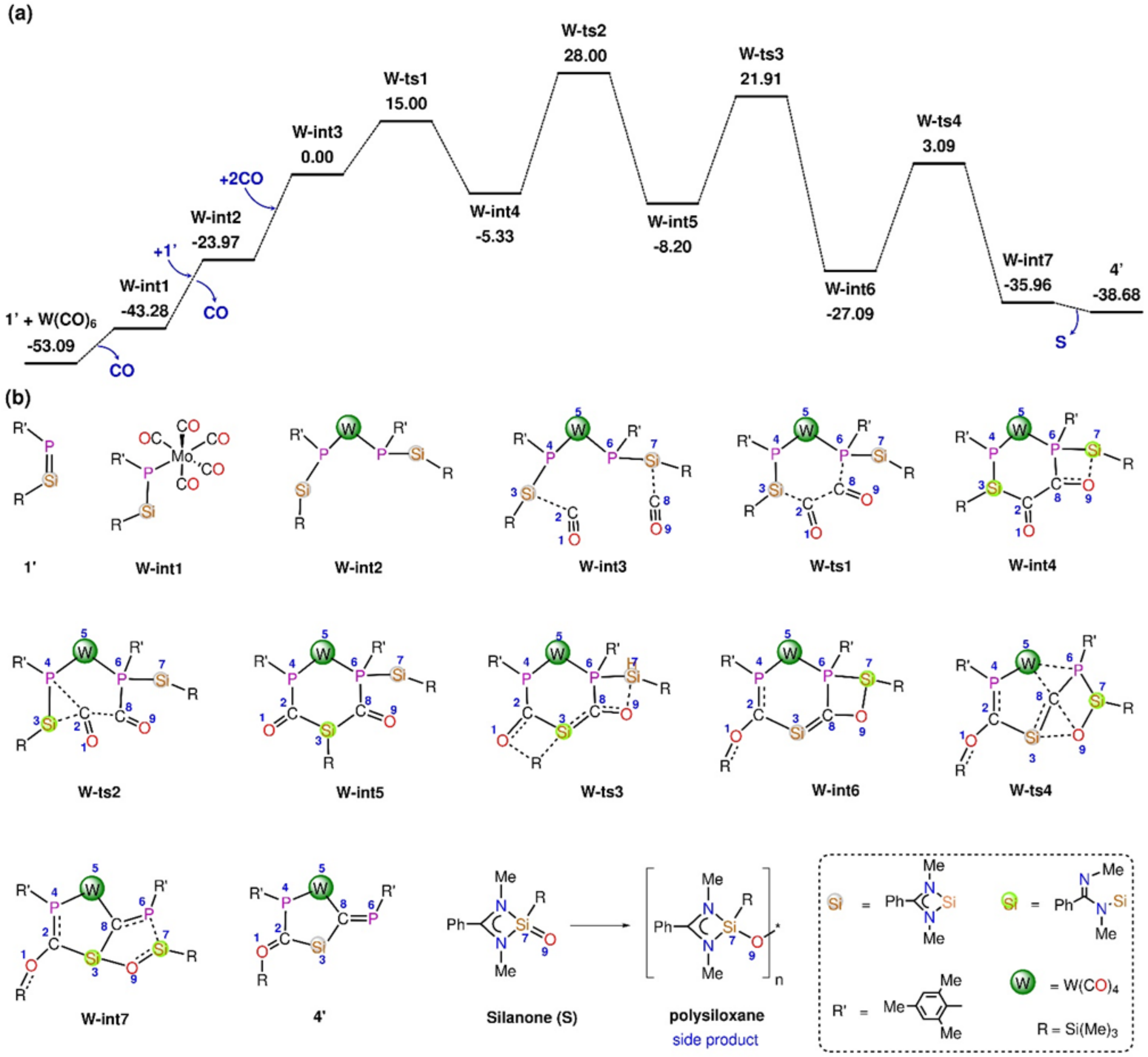

Figure S47. (a) The Gibbs free energy profile for the reaction of 1' with $W(CO)_6$ obtained at the B3LYP-D3/6-31G* level of theory. (b) Different stationary point structures existing along the reaction pathway. The substituents connected to Si, W, and P, are omitted in the images for clarity. The energies are given in kcal/mol.

Table S6. Geometrical parameters of Fe-int3 and W-int3 obtained at the B3LYP/6-31G* W (LanL2DZ) level of theory.
(X = Fe, W)

Angles	Fe-int3	W-int3
< P(4)X(5)P(6)	170.16	77.28
< X(5)P(4)Si(3)	123.20	122.06
< X(5)P(4)Si(7)	97.08	112.04
< P(6)X(5)P(4)Si(3)	98.45	-118.07
< P(4)X(5)P(6)Si(7)	-84.43	165.65

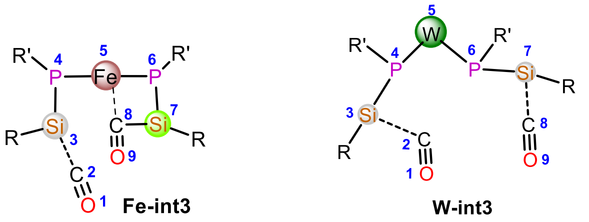

XYZ Coordinates of the stationary point structures obtained for the reaction of 1' with $Fe(CO)_5$ at the B3LYP-D3/6-31G* level of theory.

1

102

Si	-1.09488800	1.33538700	0.16315300
N	-1.20406100	-0.11272600	-1.01279400
C	-2.89423900	-1.95783600	-0.64051300
P	0.67040700	2.12378300	1.06635000
Si	-2.39668400	3.10815800	-0.70520900
N	-2.37091200	0.04181500	0.79579000
C	-4.07218500	-1.90087400	-1.39572700
H	-4.43224500	-0.94336300	-1.76009500
C	-4.76688300	-3.07411500	-1.69129200
H	-5.67963200	-3.02543800	-2.27790300
C	-4.28805500	-4.30534200	-1.23693100
H	-4.83035200	-5.21771200	-1.46781600

C	-3.11134200	-4.36259100	-0.48591900
H	-2.73656900	-5.31769500	-0.12969700
C	-2.41490700	-3.19236300	-0.18500800
H	-1.51030400	-3.22861500	0.41395000
C	-2.17067200	-0.70677200	-0.29155700
C	-0.49302300	-0.57576000	-2.22922600
C	-1.26985400	4.25312500	-1.71071900
H	-1.84419500	5.08810300	-2.13276300
H	-0.78252900	3.72543100	-2.53817600
H	-0.48063900	4.66220000	-1.07127100
C	-3.42133800	-0.02056400	1.84045400
C	2.05571400	0.84620100	0.88739200
C	3.10455700	1.12971800	-0.05783300
C	3.86705400	0.06208500	-0.55165700
H	4.60848000	0.26615700	-1.30807300
C	3.71284400	-1.25558500	-0.11966500
C	2.85582900	-1.45114400	0.95944000
H	2.79654500	-2.44405000	1.38820500
C	2.07883700	-0.42990700	1.52959000
C	-3.19322100	4.15459000	0.66595000
H	-3.70987800	5.01782400	0.22670700
H	-2.42424300	4.53548900	1.34792700
H	-3.92277500	3.59787100	1.26328600
C	-3.73330000	2.40769400	-1.86428900
H	-4.37936500	3.21362000	-2.23471400
H	-4.37132900	1.66802400	-1.36970500
H	-3.27174300	1.92498600	-2.73329700
C	3.52638200	2.56433500	-0.52046900
C	4.51970800	-2.43093000	-0.69551300
C	1.36935200	-0.82284100	2.86196200
C	4.95021200	2.56852800	-1.13325600
C	2.59032400	3.15712300	-1.59907300
C	3.60565600	3.51008600	0.70302700
C	5.32773500	-2.02939500	-1.94384500

C	5.50745800	-2.94079700	0.38053500
C	3.56850600	-3.58192200	-1.09547500
C	0.10827500	-1.65421400	2.55845100
C	0.99845900	0.37081600	3.76381300
C	2.32787200	-1.69353300	3.72420500
C	-4.76172600	0.49543800	1.28302400
C	-2.94538400	0.89470900	2.98192700
C	-3.62362900	-1.44350800	2.39781400
C	0.46477500	0.55238800	-2.63524500
C	-1.47463100	-0.84431400	-3.38454100
C	0.33116100	-1.83532300	-1.90953100
H	1.19336000	0.74510300	-1.84659800
H	1.00806000	0.26385300	-3.54131000
H	-0.07826500	1.47998000	-2.84538200
H	-2.10000900	0.03498400	-3.57300600
H	-0.90878900	-1.06326500	-4.29669700
H	-2.12609400	-1.69683200	-3.18226300
H	1.00184500	-1.64896900	-1.06602900
H	-0.31301800	-2.68595200	-1.67161400
H	0.93868000	-2.10817900	-2.77972100
H	-4.67032500	1.52277600	0.92308200
H	-5.10911200	-0.13385700	0.45793700
H	-5.52592500	0.47641400	2.06819100
H	-2.77009300	1.91592300	2.62941400
H	-3.70700000	0.93224000	3.76764500
H	-2.01386200	0.52292800	3.41711900
H	-4.27242700	-1.38611600	3.27861100
H	-4.10326400	-2.10529700	1.67432100
H	-2.67399600	-1.88920000	2.70314100
H	-0.61927000	-1.05993700	2.00490900
H	0.35706100	-2.53931400	1.95943800
H	-0.36425400	-1.99752200	3.48858400
H	0.60941000	-0.00465800	4.71876200
H	1.87976800	0.98791300	3.97444600

H	0.24955900	1.01939300	3.31260000
H	1.87965300	-1.85584700	4.71127600
H	2.51962800	-2.68277700	3.29915400
H	3.29232200	-1.19344500	3.86577700
H	4.98091500	-3.28518700	1.27716200
H	6.10109400	-3.77908300	-0.00598200
H	6.19610200	-2.14345700	0.68275000
H	6.08877700	-1.27578900	-1.71276400
H	5.84586300	-2.90627000	-2.34900800
H	4.67775500	-1.62868800	-2.73045200
H	2.87917200	-3.26144300	-1.88363700
H	4.14308200	-4.43785500	-1.47089200
H	2.96887300	-3.93023100	-0.24811800
H	2.92526600	4.16802200	-1.86681300
H	2.61559400	2.54502800	-2.50837100
H	1.56057300	3.21376000	-1.24627800
H	5.25067300	3.60594600	-1.31913300
H	5.68964800	2.12112500	-0.45928500
H	4.99950900	2.04789200	-2.09593200
H	2.63289600	3.65944200	1.17453600
H	4.29519900	3.10703000	1.45437700
H	3.97892600	4.49338000	0.39024700

1'

57

Si	1.65615500	-0.46114800	0.15924300
N	0.46095800	0.39856400	-1.00762500
C	-1.14848000	2.11697000	-0.13979200
P	1.27773700	-2.51958600	0.59136800
Si	3.79998500	0.41635300	-0.24171600
N	0.68845800	0.89506900	1.06533900
C	-1.02886200	3.44671300	0.28736300
H	-0.08102800	3.80629400	0.67648800

C	-2.12205100	4.30720100	0.19199700
H	-2.02509700	5.34006300	0.51356000
C	-3.33863400	3.84026400	-0.31275600
H	-4.19042600	4.51119900	-0.37996300
C	-3.46199900	2.51145600	-0.72823800
H	-4.40943400	2.14531000	-1.11334800
C	-2.37002400	1.64895200	-0.64920200
H	-2.46487900	0.60911800	-0.94611700
C	-0.01676600	1.17748800	-0.03157800
C	0.03194000	0.35424200	-2.38917400
C	4.33035200	0.00744200	-2.01709300
H	5.32585600	0.41842500	-2.22861800
H	3.63069600	0.42956500	-2.74834600
H	4.36925300	-1.07591300	-2.17656700
C	0.56612000	1.49293800	2.37540400
C	-0.58924500	-2.32021700	0.37657600
C	-1.19278000	-2.55842700	-0.88458200
C	-2.55921200	-2.30029000	-1.06665400
H	-3.00163300	-2.47710800	-2.04604800
C	-3.36965500	-1.82995100	-0.03115800
C	-2.78335600	-1.66988000	1.22660200
H	-3.40337800	-1.34265000	2.06004600
C	-1.42453400	-1.91991400	1.45204100
C	5.05766900	-0.29488300	0.98375100
H	6.06118400	0.10206100	0.78409000
H	5.10440400	-1.38716600	0.90820100
H	4.79359600	-0.04327100	2.01724200
C	3.67872500	2.30146500	-0.03717400
H	4.62900400	2.78889000	-0.28865300
H	3.42332300	2.57045500	0.99447100
H	2.90369800	2.71666000	-0.69262100
C	-0.40550800	-3.10482700	-2.05559300

C	-4.82489700	-1.49196800	-0.25664700
C	-0.89669800	-1.78926400	2.86284700
H	1.00252500	0.81300800	3.11109100
H	1.10367900	2.44798700	2.43811900
H	-0.48361300	1.66481900	2.63744800
H	-0.80819500	-0.33533500	-2.52603400
H	-0.26889800	1.34885900	-2.73488200
H	0.86599000	0.00705200	-3.00453600
H	-1.67884900	-1.43857400	3.54520700
H	-0.52964500	-2.75516800	3.23061800
H	-0.04969400	-1.10331700	2.92043500
H	-1.05045700	-3.24156200	-2.93101200
H	0.42446900	-2.44852600	-2.33457400
H	0.04672700	-4.07207600	-1.80782500
H	-5.45820000	-1.87992800	0.54986100
H	-4.97871000	-0.40407700	-0.28638000
H	-5.19282000	-1.90266800	-1.20288300

2'

88

Fe	-2.14369000	-1.94811900	-1.15530700
P	-2.34036500	0.06694300	-0.46802700
Si	1.82392400	-1.70833200	0.36570700
O	0.68952800	-2.70582300	-0.73298500
N	2.97448200	-1.18574500	-0.95557000
C	4.49023900	0.82589000	-0.77721700
P	-0.80214600	0.45297500	1.07254100
Si	2.40804800	-3.44132200	1.82656400
O	-2.50536100	-3.95390000	0.91100800
N	3.04810100	-0.23915400	0.96808800
C	5.54728100	1.29420400	0.01770800
H	5.71326300	0.86143400	0.99883700

O	-1.04543400	-3.56328800	-3.36721500
C	6.38762000	2.30126700	-0.45423300
H	7.20663900	2.65444400	0.16552400
O	-4.94321900	-1.82500400	-2.07843300
C	6.17545900	2.85460900	-1.71978300
H	6.82741100	3.64347100	-2.08419800
C	5.12627100	2.39024000	-2.51770200
H	4.95667000	2.81978600	-3.50080800
C	4.28994100	1.37729800	-2.05253200
H	3.46075100	1.03583500	-2.66183100
C	3.55199500	-0.18370800	-0.24902500
C	3.29630300	-1.67614200	-2.27991800
C	-0.30452100	-1.83250100	-0.42517000
C	0.18460600	-0.86913500	0.44419000
C	0.80419900	-4.07974000	2.61228900
H	0.98802400	-4.96253800	3.23757500
H	0.07997100	-4.35250900	1.83704200
H	0.33896000	-3.31012300	3.23905500
C	3.12027000	0.76492900	2.00586300
C	-3.84578300	1.02018800	-0.04286900
C	-4.01924800	2.29895000	-0.62345200
C	-5.16835300	3.03292000	-0.31910800
H	-5.30209200	4.01401700	-0.77046700
C	-6.14729700	2.54299900	0.55020900
C	-5.95146100	1.28371400	1.12235600
H	-6.70073300	0.89152100	1.80712300
C	-4.81957800	0.50888000	0.84690600
C	0.15420800	2.00909100	0.68293100
C	0.82816700	2.25220600	-0.54433700
C	1.70846300	3.33468100	-0.62975700
H	2.24251900	3.50039400	-1.56273100
C	1.94029700	4.19883000	0.44347600

C	1.21849900	3.98745100	1.61802400
H	1.35929800	4.66575900	2.45702300
C	0.32852700	2.91445200	1.75750900
C	3.59252600	-2.82717500	3.17809800
H	3.86666900	-3.63434100	3.86907600
H	3.13189600	-2.02363500	3.76525200
H	4.51438700	-2.42858700	2.73829500
C	3.23170000	-4.83706500	0.83611800
H	3.45935800	-5.69713500	1.47868800
H	4.16975800	-4.50399700	0.37583300
H	2.56668200	-5.17874300	0.03472200
C	-2.36501800	-3.18416500	0.06158200
C	-1.48645200	-2.94915600	-2.50049300
C	-3.83814700	-1.86964800	-1.75272900
C	-2.98527600	2.89445100	-1.55532300
C	-7.36470700	3.36923800	0.89040100
C	-4.68129800	-0.84154300	1.51372900
C	0.63493300	1.40699000	-1.78027600
C	2.93744500	5.32505200	0.31221900
C	-0.39478700	2.75869900	3.08148300
H	-7.16645600	4.02424700	1.74936700
H	-7.65983300	4.01146100	0.05369400
H	-8.21866400	2.73634700	1.15383200
H	-3.67989100	-0.98914300	1.93066500
H	-5.40874700	-0.94893200	2.32405500
H	-4.85528200	-1.65770900	0.80340400
H	-0.20182200	3.62248500	3.72567800
H	-1.47808600	2.67306100	2.94777700
H	-0.07178100	1.86059000	3.62128500
H	3.91268100	4.94420700	-0.01552100
H	2.61186600	6.05988300	-0.43499800
H	3.07891000	5.85256100	1.26077200

H	1.10527100	0.42595300	-1.67786600
H	-0.42496000	1.22050100	-1.97548100
H	1.05997100	1.90749700	-2.65596700
H	3.26248200	1.77155600	1.59856400
H	2.17667700	0.75636000	2.55902100
H	3.92747700	0.54941300	2.71798900
H	3.15740600	-2.75976300	-2.30121000
H	2.63627600	-1.24125600	-3.04173400
H	4.33366000	-1.44628700	-2.54361300
H	-2.82061000	2.26182100	-2.43584300
H	-3.29922700	3.88247800	-1.90626300
H	-2.01506400	3.01002300	-1.05675100

Optimized structure of the side product Silanone (S)

37

Si	1.516143000	-0.985974000	0.736244000
O	2.195461000	-2.086649000	1.582276000
N	-0.015236000	-1.354146000	-0.326576000
C	-2.138575000	-0.079149000	0.087675000
Si	2.634807000	0.655286000	-0.533648000
N	0.004813000	-0.057276000	1.393184000
C	-2.474978000	1.285285000	0.081264000
H	-1.705494000	2.031319000	0.288597000
C	-3.779745000	1.683121000	-0.215482000
H	-4.033467000	2.745770000	-0.229341000
C	-4.758937000	0.723694000	-0.494994000
H	-5.781115000	1.036664000	-0.721888000
C	-4.430249000	-0.636091000	-0.484632000
H	-5.195066000	-1.386561000	-0.698229000
C	-3.123705000	-1.038300000	-0.200929000
H	-2.865552000	-2.099136000	-0.185100000
C	-0.752321000	-0.499945000	0.389024000

C	-0.323719000	-1.965582000	-1.595616000
C	4.018856000	1.451316000	0.480717000
H	4.557955000	2.207354000	-0.115026000
H	4.743844000	0.690252000	0.810434000
H	3.621537000	1.946786000	1.381461000
C	-0.357601000	0.767805000	2.516170000
C	1.371246000	1.987640000	-1.027807000
H	1.827247000	2.742193000	-1.690697000
H	0.982924000	2.510122000	-0.138091000
H	0.512971000	1.545743000	-1.560278000
C	3.366525000	-0.103845000	-2.108887000
H	3.920099000	0.656710000	-2.685466000
H	2.578687000	-0.513145000	-2.762355000
H	4.063668000	-0.921721000	-1.866183000
H	-1.445210000	0.758434000	2.689340000
H	0.141007000	0.388007000	3.421630000
H	-0.044272000	1.818309000	2.374452000
H	0.586571000	-2.004050000	-2.213699000
H	-0.678879000	-3.003237000	-1.469132000
H	-1.092269000	-1.402746000	-2.150239000

Fe-int1

66

P	-1.12617300	-0.63447700	0.82014500
Si	-0.18605600	1.27811400	0.35290100
Si	-1.41206300	3.24798000	0.75578700
N	1.51096000	1.27166800	1.07185000
C	0.34317400	-1.76587700	0.59212700
C	0.95270600	-2.23908800	1.78309300
C	2.15605000	-2.95455200	1.70931500
H	2.61898500	-3.29934700	2.63188500
C	2.77111000	-3.24514400	0.49081100

C	2.12674000	-2.82992000	-0.67640200
H	2.57314200	-3.07136300	-1.63935600
C	0.92587300	-2.11180900	-0.65448500
C	0.34128600	-2.01992100	3.15295100
C	4.08583900	-3.98522000	0.42239900
C	0.28518100	-1.78137300	-1.98054900
C	-2.98541800	3.25398700	-0.28954500
H	-3.48287800	4.22894700	-0.21269400
H	-3.67945000	2.48593600	0.06624100
H	-2.77796300	3.05175500	-1.34482200
C	-0.27637600	4.69390500	0.29368100
H	-0.76634000	5.65177600	0.50792400
H	-0.03000100	4.67344700	-0.77431400
H	0.66312300	4.66215200	0.85755200
C	-1.85269900	3.27089400	2.59577500
H	-2.40127500	4.18819600	2.84434000
H	-0.96273300	3.22638100	3.23413400
H	-2.49337500	2.41642000	2.84093500
N	1.06179100	1.48067300	-1.01693700
C	1.14980900	1.63237700	-2.45777500
C	2.16641300	1.07055500	2.34880700
C	2.07008200	1.34057100	-0.14525100
C	3.50167100	1.20306000	-0.46004500
C	4.20480800	0.10284300	0.05676300
H	3.68991900	-0.62995500	0.66908700
C	5.55076600	-0.06723600	-0.26135100
H	6.09043700	-0.92477300	0.12972900
C	6.20063200	0.85959700	-1.08095900
H	7.25116700	0.72656400	-1.32293500
C	5.50165100	1.95588200	-1.59350600
H	6.00685000	2.67667100	-2.22937000
C	4.15108900	2.12565200	-1.29279600

H	3.60637600	2.97795700	-1.68662000
H	-0.09338000	-0.76251000	-2.02120900
H	0.99561400	-1.91832600	-2.80203800
H	-0.57204600	-2.43847100	-2.16313800
H	4.45825600	-4.23967800	1.41977900
H	3.98970400	-4.91678400	-0.14855800
H	4.85207500	-3.38097700	-0.08059500
H	0.29445000	-0.96128200	3.42756700
H	-0.68825600	-2.39226400	3.19080400
H	0.92122100	-2.54458600	3.91970500
H	1.53960100	1.49801100	3.13503100
H	2.31292700	0.00621100	2.55910000
H	3.13919200	1.57123300	2.36127900
H	1.35509900	2.67350800	-2.73409800
H	1.94051600	0.99572200	-2.86729100
H	0.19541700	1.34342600	-2.89741700
Fe	-3.12463300	-0.97148700	-0.46362200
C	-2.51581000	0.07546900	-1.73649600
C	-3.90824500	-0.18402200	0.93118300
C	-4.65562700	-1.25221700	-1.28953600
C	-2.67094700	-2.69051200	-0.47346100
O	-5.65982500	-1.43617100	-1.83624800
O	-2.32374000	-3.79797700	-0.52383100
O	-2.10752400	0.79419100	-2.56399900
O	-4.37984300	0.41779900	1.80743500

Fe-ts1'

68

P	1.45849800	0.52380800	0.08735600
Si	-0.92202700	1.48162500	0.17771100
Si	-1.38299700	3.69258900	0.90280800

N	-1.85482500	0.95196600	-1.33162500
C	0.64931000	-0.84338900	1.14637200
C	0.05365900	-1.98647900	0.56943400
C	-0.45722000	-2.97841500	1.41531900
H	-0.91097700	-3.86330800	0.97083100
C	-0.37498900	-2.88329700	2.80847400
C	0.22107500	-1.74299200	3.35553800
H	0.29487200	-1.65075300	4.43767700
C	0.73424800	-0.71797200	2.55195700
C	0.24976900	4.57580300	1.24759500
H	0.06220900	5.57614400	1.65714600
H	0.83902400	4.68284300	0.33192000
H	0.85501500	4.02169600	1.97400900
C	-2.37701900	3.52617000	2.51049700
H	-2.57706200	4.51981400	2.93094100
H	-1.82688500	2.95313300	3.26655600
H	-3.34145800	3.03370800	2.34280000
C	-2.39694900	4.58743700	-0.42027200
H	-2.67201500	5.59514000	-0.08402600
H	-3.31966700	4.04281800	-0.65074500
H	-1.81789800	4.68093700	-1.34523400
N	-2.26397100	0.34007700	0.68740700
C	-2.86246800	-0.17666600	1.90302800
C	-1.58833100	0.88055300	-2.76060100
C	-2.62035900	0.12862100	-0.59177900
H	-1.89342000	1.81031700	-3.24995100
H	-0.51848000	0.73203200	-2.93447200
H	-2.12867300	0.04419400	-3.20977000
H	-3.89978500	-0.47298100	1.72391600
H	-2.29940000	-1.03756100	2.27355500
H	-2.84886400	0.60451900	2.66738800
C	2.47455800	-0.44545200	-2.36697500
C	3.31412400	-1.94630900	-0.03184600
C	4.93846500	-0.73123200	-1.71223400

C	4.19541800	0.82447300	0.19538100
O	5.92729700	-0.94708200	-2.27591800
O	4.69885000	1.55339900	0.94855000
O	1.80780300	-0.55091000	-3.31798400
O	3.24967000	-2.98650100	0.47786300
C	1.12212600	1.85704000	-0.98064100
O	0.67795700	2.81437400	-1.53523400
C	-3.61989100	-0.83196300	-1.07453500
C	-3.66554600	-2.12451400	-0.52539700
C	-4.52489800	-0.46773600	-2.08451000
C	-4.60343100	-3.04270400	-0.99204200
H	-2.94970200	-2.40958200	0.23897900
C	-5.46694300	-1.38863300	-2.53787600
H	-4.49779700	0.53704800	-2.49477000
C	-5.50518800	-2.67610600	-1.99505800
H	-4.62853300	-4.04527100	-0.57559800
H	-6.17087100	-1.10228600	-3.31342800
H	-6.23716900	-3.39367900	-2.35421400
C	-0.88618900	-3.99484600	3.69349700
C	1.37409100	0.48629200	3.21141200
C	-0.01145600	-2.20674400	-0.92400000
H	0.80030800	1.40435800	3.02850700
H	1.44179100	0.34585900	4.29464600
H	2.38331600	0.67071700	2.82713300
H	-1.72526000	-4.52461100	3.22942200
H	-0.09905600	-4.73634900	3.88331200
H	-1.21658400	-3.61533800	4.66634400
H	-0.81143200	-2.90694600	-1.18409100
H	-0.17238600	-1.27962500	-1.47828000
H	0.93350500	-2.62422200	-1.29012400
Fe	3.42651400	-0.38788700	-0.86735100

Fe-int2'

68

P	1.37643000	-0.42466700	-0.69588300
Si	-1.19669100	1.48831400	0.15499900
Si	-1.26339400	3.55301700	1.27536400
N	-2.33556400	1.25784700	-1.25630700
C	0.72622600	-1.35442900	0.76820100
C	-0.03715400	-2.52433400	0.52434500
C	-0.46600000	-3.30567100	1.60097400
H	-1.04341100	-4.20651400	1.40184200
C	-0.17690800	-2.96315200	2.92491600
C	0.56289100	-1.80196200	3.15332000
H	0.80497600	-1.52178800	4.17667000
C	1.03024000	-0.99625500	2.10469200
C	-0.42068600	-2.95450000	-0.87603800
C	-0.68078700	-3.80876900	4.07010500
C	1.89095200	0.19522700	2.45598200
C	0.41186800	3.79750300	2.11045800
H	0.48272000	4.80978100	2.52727200
H	1.22880500	3.66484500	1.39279300
H	0.56233700	3.08578500	2.92971100
C	-2.67325100	3.46772200	2.53686500
H	-2.75491600	4.41262800	3.08797900
H	-2.50635200	2.66734100	3.26720300
H	-3.63583900	3.28155000	2.04635400
C	-1.58204600	4.88675300	-0.02587900
H	-1.58934800	5.88126000	0.43740700
H	-2.54552000	4.74081500	-0.52788600
H	-0.79255400	4.86636100	-0.78439800
N	-2.45121100	0.22647500	0.62141900
C	-2.74446900	-0.68854100	1.70831900
C	-2.49338000	1.75105100	-2.61065100

C	-3.00677600	0.29672000	-0.60187500
C	-4.08726400	-0.54961600	-1.12730500
C	-3.95177800	-1.14462700	-2.39246900
H	-3.05148700	-0.97962500	-2.97562200
C	-4.95947500	-1.97442000	-2.87983400
H	-4.84675600	-2.44317900	-3.85255100
C	-6.10805100	-2.20284100	-2.11762000
H	-6.89416600	-2.84585700	-2.50246700
C	-6.24714600	-1.60890700	-0.86013900
H	-7.14091000	-1.78445900	-0.26921000
C	-5.23712600	-0.79058200	-0.35904900
H	-5.34625900	-0.32352900	0.61447300
H	1.64417100	1.08670900	1.87641100
H	1.80366500	0.44007300	3.51932000
H	2.94463100	-0.02075700	2.24651200
H	-0.62584400	-4.87658400	3.83142600
H	-0.10363600	-3.63363400	4.98365800
H	-1.73162700	-3.58196800	4.29597700
H	-1.08471600	-2.22178100	-1.35063000
H	0.45373500	-3.05253600	-1.52800700
H	-0.94190000	-3.91713500	-0.85693900
H	-2.33866900	2.83262500	-2.61429800
H	-1.75487400	1.29630200	-3.28005000
H	-3.49814500	1.53390000	-2.98264800
H	-3.52163200	-0.28288700	2.36687500
H	-3.07016200	-1.65838600	1.32369800
H	-1.83278400	-0.84009900	2.28781000
C	3.56205900	1.35920900	-0.22810400
C	3.44495000	-0.61854800	-2.67647600
C	5.44750900	-0.09493200	-1.16968800
O	6.59148500	0.01882900	-1.31087300
O	3.45822200	2.39221700	0.29479800
O	3.27752400	-0.86488600	-3.79833800
C	0.50639000	1.10951500	-0.56567400

O	0.70480100	2.19173300	-1.16980200
C	3.87343900	-1.59402700	0.21114900
O	3.98228200	-2.43368200	1.00721000
Fe	3.70803000	-0.26028200	-0.94178500

Fe-int2

121

P	2.36306900	0.21042600	-1.64233900
Si	3.21179900	1.40203500	-0.03795100
Si	2.60662700	3.66956400	0.08222600
N	3.54828800	0.72720500	1.67628600
C	1.32146300	4.00044100	-1.26067600
H	0.89751800	5.00800400	-1.16866300
H	0.51036700	3.26926700	-1.19797300
H	1.76200600	3.89935500	-2.25862100
C	4.15719900	4.73176000	-0.18368100
H	3.91477000	5.79883000	-0.10039800
H	4.58514000	4.56236200	-1.17857700
H	4.92957500	4.50228800	0.55971100
C	1.90825100	3.96572900	1.81973700
H	1.49178900	4.97649800	1.91138300
H	2.69276700	3.85135400	2.57745500
H	1.11339900	3.24656100	2.04123100
N	5.01371900	1.03069300	0.13966800
C	4.83217900	0.51408200	1.36483600

Fe	0.02808500	0.06047800	-1.54932500
C	-0.01772300	1.06338700	-3.02285000
C	0.12693600	-1.67750700	-1.76093100
C	0.00183300	0.66689000	0.06877200
O	0.19274200	-2.84471800	-1.82541200
O	0.00474800	1.04290300	1.19816800
O	-0.04203800	1.76816700	-3.95571000
P	-2.29974900	-0.08338000	-1.68467200
C	-3.17332000	-1.66419400	-1.17904200
C	-4.16798200	-2.15478500	-2.06390300
C	-2.95315100	-2.35834000	0.03931800
C	-4.96066600	-3.24841900	-1.68751500
C	-3.76964700	-3.44545700	0.37301300
C	-4.78999000	-3.89945700	-0.46492300
H	-5.72806000	-3.60251400	-2.37378000
H	-3.60273000	-3.95227200	1.32230800
Si	-3.34168600	1.27180600	-0.34799700
N	-3.70691600	0.88475000	1.45272000
N	-5.10763900	0.75103200	-0.16687200
Si	-2.98596400	3.58495500	-0.55440900
C	-4.94459300	0.48480300	1.13712900
C	-6.26663500	0.49466100	-0.99609200
C	-3.03500400	0.78794900	2.73390700
H	-6.22744800	-0.50697800	-1.43694900

H	-7.18599100	0.58780600	-0.40968400
H	-6.29016200	1.22973200	-1.80464700
H	-1.95886400	0.83409800	2.56119300
H	-3.31458400	1.61891600	3.39327700
H	-3.27726600	-0.15514000	3.23533800
C	-5.90875500	-0.19412000	2.01983000
C	-6.21451200	0.32156200	3.28754700
C	-6.49093800	-1.39812600	1.59183300
C	-7.10932700	-0.35532400	4.11494600
H	-5.76580000	1.25439800	3.61462300
C	-7.37335900	-2.07615100	2.43076700
H	-6.22138600	-1.81576400	0.62765300
C	-7.68700000	-1.55445300	3.68872900
H	-7.35332800	0.05107900	5.09208300
H	-7.81282600	-3.01347700	2.10202100
H	-8.37870100	-2.08317600	4.33848200
C	-1.83406600	-2.00992200	0.99073600
C	-5.66211500	-5.06413500	-0.05821900
C	-4.40541800	-1.54419000	-3.43078100
H	-0.88122600	-2.37470200	0.59525000
H	-1.71128600	-0.93801200	1.12534400
H	-1.99744800	-2.47091400	1.97103000
H	-6.49983000	-5.20113600	-0.74998800
H	-5.09223600	-6.00193700	-0.03713900

H	-6.07389900	-4.91873700	0.94864300
H	-5.15313300	-2.12177000	-3.98571300
H	-4.75086400	-0.50716200	-3.36945800
H	-3.48217000	-1.52409900	-4.02010800
C	5.82970200	-0.21229200	2.16917200
C	6.56184400	-1.25249100	1.57445700
C	6.02260500	0.09566700	3.52390500
C	7.47926900	-1.97526200	2.33487700
H	6.38159700	-1.51647500	0.53813200
C	6.95284800	-0.62239500	4.27386900
H	5.45687000	0.90202300	3.98044400
C	7.67946900	-1.65874600	3.68119500
H	8.03429600	-2.78810000	1.87552000
H	7.10811100	-0.37563700	5.32003900
H	8.39863000	-2.22127300	4.26984900
C	6.20585800	1.00699800	-0.68182300
C	2.84186000	0.37101600	2.88969000
H	6.20208500	1.88576100	-1.33183000
H	7.10347900	1.03695500	-0.05652900
H	6.23919000	0.10889900	-1.30809700
H	2.91897300	1.16866900	3.63928500
H	1.78798400	0.23064300	2.64623500
H	3.24071900	-0.55364300	3.31863900
C	3.39356600	-1.33723600	-1.38965900

C	3.27064000	-2.22851000	-0.29464900
C	4.41391500	-1.58341800	-2.34725500
C	4.19390500	-3.27224900	-0.14391700
C	5.31207000	-2.64061100	-2.15634700
C	5.22963700	-3.49141400	-1.05105600
H	4.09750200	-3.93132700	0.71810000
H	6.09542400	-2.80562100	-2.89421100
C	4.57256000	-0.73381200	-3.59208600
C	6.20804900	-4.62563200	-0.85366800
C	2.16233200	-2.15718700	0.73136600
H	1.63663300	-1.20581400	0.71998100
H	1.41724700	-2.93127700	0.51525100
H	2.55045300	-2.34182700	1.73930700
H	7.11521600	-4.48329100	-1.45072300
H	6.50329200	-4.71783400	0.19859100
H	5.76823000	-5.58733200	-1.14904400
H	5.32812600	-1.16511100	-4.25821400
H	3.63052900	-0.65694400	-4.14515000
H	4.87316100	0.29302800	-3.35647400
C	-2.36577900	3.99501700	-2.29131300
C	-4.64035000	4.45611600	-0.22099700
C	-1.73202300	4.06697100	0.78118200
H	-5.38803000	4.19622400	-0.97923900
H	-5.04410500	4.17485300	0.75886900

H	-4.51405900	5.54617100	-0.23264500
H	-1.50231200	3.38691900	-2.57625600
H	-3.15274700	3.81629000	-3.03315600
H	-2.07547100	5.05163100	-2.35441400
H	-1.37880200	5.09524000	0.63538700
H	-2.18374000	4.00077100	1.77827000
H	-0.86704700	3.39965100	0.76463400

Fe-int3

125

C	-6.19183100	0.28210300	0.11113400
N	-5.03151500	-0.58675800	0.12770000
O	0.14044900	-0.26245200	-2.00844100
C	6.10034900	0.81936200	-0.36339300
Si	-3.27320200	-0.62560300	-0.48365600
C	-4.92212900	-1.83091900	0.60423900
N	4.85076000	0.22140000	-0.77984200
Si	2.86911300	0.58291100	-1.04801600
N	-3.67904300	-2.24875200	0.31126500
C	0.85888200	0.22033500	-1.19415000
C	4.65117400	-1.03192100	-1.11900200
C	-3.00240600	-3.42570300	0.82706800
N	3.34901500	-1.16269000	-1.46801300
C	2.61725600	-2.39250500	-1.70527400
C	-5.96415800	-2.60161700	1.30296200
C	-6.20465200	-3.93779300	0.94657000
C	-6.72066200	-2.00278300	2.32177500
C	-7.19943900	-4.66329200	1.59889200
H	-5.62435000	-4.39484700	0.15099600

C	-7.70567600	-2.73763000	2.97895700
H	-6.51939000	-0.97643800	2.61112200
C	-7.94830900	-4.06508400	2.61605400
H	-7.38968500	-5.69397500	1.31481000
H	-8.28176000	-2.27529300	3.77484600
H	-8.71978100	-4.63402800	3.12692500
C	5.65118600	-2.12246000	-1.09795600
C	5.89288800	-2.89840400	-2.24059900
C	6.36548300	-2.37579400	0.08254200
C	6.84514300	-3.91670800	-2.20300100
H	5.34389100	-2.69184100	-3.15489300
C	7.30853900	-3.40237600	0.11703200
H	6.16408400	-1.78018500	0.96853700
C	7.55129200	-4.17134500	-1.02423900
H	7.03557000	-4.51068000	-3.09237700
H	7.85383300	-3.60148000	1.03532200
H	8.28976900	-4.96780600	-0.99574500
H	-1.97468600	-3.16320800	1.08380500
H	-3.50915700	-3.79462600	1.72318100
H	-2.98058600	-4.22765900	0.08000700
H	-6.11352800	0.95955100	-0.74008300
H	-7.11504600	-0.29996700	0.02556400
H	-6.23670700	0.89709000	1.01546600
H	6.10156000	1.87304300	-0.65409800
H	6.21181400	0.77447700	0.72577300
H	6.96217300	0.32583300	-0.82833600
H	3.29560700	-3.24935600	-1.72241100
H	1.87411900	-2.55759300	-0.91904700
H	2.08933000	-2.34332300	-2.66214500
P	-2.23859000	0.67073100	0.99079500
P	2.45976200	1.48269900	0.97208000
Fe	0.10651200	0.89847600	1.08874500

C	0.06755400	1.20139200	2.82229700
C	-0.02975300	2.56883300	0.43642600
C	0.18636100	-0.85761800	0.98412100
O	-0.12277700	3.66718900	0.07281100
O	0.09642300	1.36685200	3.97108900
O	0.14229100	-2.02436000	0.87603500
C	3.76390900	0.87755100	2.15822100
C	3.98569100	-0.47227400	2.53200600
C	4.64191700	1.86693800	2.67901500
C	5.07453800	-0.80392000	3.34819100
C	5.71274100	1.49038000	3.50119100
C	5.95964600	0.15934500	3.83708300
H	5.22712800	-1.84796000	3.61852400
H	6.37775600	2.26298200	3.88317700
C	-3.20222100	2.18273500	0.41829500
C	-4.10932600	2.73254600	1.36905300
C	-3.14274600	2.76835600	-0.87036700
C	-4.94953300	3.78529500	1.00177800
C	-4.02457600	3.81086600	-1.19949400
C	-4.93685500	4.33180300	-0.28639600
H	-5.64081200	4.18959800	1.73936300
H	-3.97494300	4.23481900	-2.20152900
C	3.06443300	-1.58784400	2.11026600
C	7.15023300	-0.23084900	4.68074500
C	4.49323300	3.34069700	2.35985300
C	-4.20659700	2.20576600	2.78615000
C	-2.14435200	2.37701000	-1.93757800
C	-5.85392700	5.47458200	-0.65186700
H	-4.89306300	2.82015500	3.37878600
H	-4.56151800	1.16887400	2.81032500
H	-3.23219100	2.20558900	3.28670800
H	-5.96058900	5.57345100	-1.73719500

H	-6.85304700	5.34040100	-0.22125700
H	-5.46518500	6.42845000	-0.27144500
H	-1.41686900	3.18276900	-2.07625100
H	-1.57943200	1.47950500	-1.69103500
H	-2.63932400	2.22450300	-2.90273200
H	2.09544500	-1.49863300	2.61122100
H	2.86259200	-1.57235500	1.03883500
H	3.48628100	-2.56593600	2.36482100
H	6.93022400	-1.10264100	5.30712800
H	8.01293000	-0.49272900	4.05268700
H	7.46366400	0.58860200	5.33651200
H	5.21669000	3.93181900	2.93258900
H	4.65690500	3.54037400	1.29452300
H	3.48770400	3.70756100	2.58795700
Si	-3.68911800	-0.87467100	-2.81981200
Si	3.07169200	2.06136300	-2.87178000
C	3.64257100	1.08226000	-4.39913300
C	4.32852300	3.43153300	-2.48379600
C	1.38472200	2.85905000	-3.21885200
C	-4.44812600	-2.60756000	-2.99985600
C	-5.01182300	0.40797500	-3.29301900
C	-2.21841100	-0.70349500	-3.99368200
H	4.27730500	4.23284700	-3.23199900
H	4.12860200	3.87416400	-1.50079800
H	5.35313200	3.04294700	-2.47915600
H	4.59901700	0.58210600	-4.20466500
H	2.90976700	0.31017800	-4.66197000
H	3.77311000	1.73613500	-5.27091600
H	1.44726700	3.57827900	-4.04523600
H	0.63739600	2.10116400	-3.47857700
H	1.02150600	3.38900200	-2.33101500
H	-5.31655100	-2.72852400	-2.34200700

H	-4.78157800	-2.77412000	-4.03206500
H	-3.72230100	-3.38832700	-2.74759100
H	-5.99759500	0.09718100	-2.92953800
H	-4.80363200	1.40485600	-2.89106400
H	-5.07207600	0.49014800	-4.38556600
H	-1.46024200	-1.47325800	-3.83290400
H	-2.58843600	-0.79598800	-5.02342500
H	-1.72178600	0.26388100	-3.89047000
C	-1.05261500	-3.01798900	-1.67805800
O	-0.24380600	-3.64257500	-2.17721600

Fe-ts1

125

C	-1.76260300	-1.95767600	2.60308700
N	-2.53994200	-1.61646800	1.42182500
O	0.57021500	-1.31114500	-2.55738300
C	5.63841900	0.95942900	0.24140200
Si	-2.18921700	-1.40051000	-0.38525100
C	-3.89182400	-1.63455000	1.30723400
N	4.65697600	-0.02257600	-0.16518300
Si	2.91914900	-0.17748000	-1.11007500
N	-4.19459700	-1.48212100	0.03925900
C	0.99609900	-0.78166400	-1.57516300
C	4.58460600	-1.29519500	0.18662000
C	-5.51695900	-1.32781900	-0.52590700
N	3.41669700	-1.76673400	-0.29122400
C	2.97111700	-3.14517200	-0.35179700
H	-5.90651300	-2.28875300	-0.88816000
H	-6.22529900	-0.91838100	0.20279300
H	-5.46172600	-0.63953000	-1.37115300
H	-1.14113500	-1.11072400	2.90910600
H	-2.42617700	-2.22638300	3.42869900

H	-1.10082300	-2.80285300	2.39492100
H	6.43632700	1.06107900	-0.50577300
H	5.14318900	1.92708500	0.34915200
H	6.09452900	0.69486400	1.20183800
H	2.33314300	-3.39706700	0.50199100
H	2.38079300	-3.28888300	-1.26029400
H	3.82315500	-3.83191300	-0.37867100
P	-2.19092800	0.55863300	-1.50284500
P	2.10310000	1.84404800	-0.44018200
Fe	-0.16838500	1.54933200	-0.75532400
C	-0.82288300	3.15866100	-0.65649000
C	0.12707200	1.70586700	-2.54572800
C	-0.27199900	1.31966300	1.01299500
O	0.28543000	1.89352600	-3.67036700
O	-1.25668900	4.22770300	-0.56538600
O	-0.31624000	1.21970600	2.16348700
Si	-2.25791700	-3.46101900	-1.53309500
Si	3.91793700	-0.09708600	-3.25845300
C	5.26619700	-1.44020000	-3.26997900
C	4.71981800	1.60772300	-3.47653400
C	2.72897800	-0.42482100	-4.69470200
C	-0.55080000	-4.29084500	-1.59369300
C	-3.46073200	-4.62714700	-0.62928800
C	-2.88204200	-3.12703600	-3.29187100
H	5.22569200	1.68305700	-4.44766800
H	3.96388000	2.39876000	-3.41985700
H	5.45930800	1.80309600	-2.69157700
H	5.98694200	-1.28704300	-2.45881600
H	4.83182200	-2.43918500	-3.14651700
H	5.81725500	-1.42850400	-4.21878800
H	3.27544900	-0.39502000	-5.64637800
H	2.25230800	-1.40522000	-4.59825700

H	1.93184800	0.32383100	-4.73670100
H	-0.09718700	-4.33868000	-0.59651900
H	-0.62898200	-5.31475300	-1.98031100
H	0.12655000	-3.72881100	-2.24365700
H	-3.20707400	-4.71119500	0.43440600
H	-4.49035000	-4.25936900	-0.69796900
H	-3.43033800	-5.63481200	-1.06281000
H	-2.20934900	-2.43138100	-3.80594500
H	-2.94204000	-4.05018200	-3.88204100
H	-3.87954100	-2.67141300	-3.27192700
C	-0.18843400	-1.15778100	-0.08659100
O	0.52275900	-1.60357000	0.76354500
C	2.61164200	2.44175100	1.24302200
C	2.77723600	3.85690800	1.33202400
C	2.85279800	1.66459400	2.40014800
C	3.19795500	4.43399500	2.53094800
C	3.28561300	2.29327800	3.57792900
C	3.47192500	3.66888100	3.66937700
H	3.31737900	5.51499800	2.57910000
H	3.46847500	1.67460200	4.45559600
C	-3.78510600	1.46142500	-1.20121500
C	-4.60447500	1.67225900	-2.34464000
C	-4.24519000	1.93431400	0.05301700
C	-5.84733600	2.29999700	-2.20360800
C	-5.49905400	2.55069800	0.14559200
C	-6.32168900	2.73707300	-0.96556800
H	-6.46444100	2.44842300	-3.08790800
H	-5.83932000	2.90032100	1.11889800
C	-3.42418100	1.84418700	1.31596700
C	-4.19645100	1.22140100	-3.73319000
C	-7.68732500	3.36762500	-0.83044900
C	2.66958300	0.16778000	2.47552500

C	2.49993800	4.77837100	0.16214000
C	3.95915400	4.32054800	4.94163800
H	-7.97380300	3.90353200	-1.74193200
H	-8.45762000	2.60642800	-0.64566800
H	-7.72257000	4.07430800	0.00579900
H	-2.65893200	2.62885600	1.33803900
H	-4.05528500	1.98194400	2.20018300
H	-2.90372200	0.89188000	1.41139800
H	-3.22629600	1.63502400	-4.02733900
H	-4.09431900	0.13170500	-3.79362800
H	-4.94241000	1.53459700	-4.47181700
H	3.09546300	4.50773400	-0.71663800
H	1.45036900	4.73663200	-0.15037500
H	2.72512500	5.81643100	0.42859100
H	5.00725100	4.63574500	4.85022700
H	3.37540600	5.21742200	5.18041600
H	3.89380000	3.63667400	5.79438700
H	3.63867200	-0.34301400	2.52442600
H	2.11895700	-0.10075800	3.38438100
H	2.12023700	-0.23819700	1.62996500
C	-4.84216900	-1.76114800	2.43389300
C	-5.87155600	-2.71159000	2.38026300
C	-4.73709700	-0.91246000	3.54635500
C	-6.78179000	-2.81598500	3.43135800
H	-5.94537000	-3.37225100	1.52174300
C	-5.65628800	-1.01285500	4.58975600
H	-3.94759100	-0.16825300	3.57954900
C	-6.67707800	-1.96563400	4.53508400
H	-7.57262000	-3.55933000	3.38816200
H	-5.57600100	-0.34718100	5.44429300
H	-7.39032800	-2.04447600	5.35073700
C	5.56716000	-2.06536900	0.97108100

C	5.13313600	-2.89619200	2.01627600
C	6.93801000	-1.96417500	0.68918500
C	6.06102800	-3.61105200	2.77147200
H	4.07394000	-2.96263000	2.24359700
C	7.86097800	-2.69115000	1.43913000
H	7.27391700	-1.33030800	-0.12541300
C	7.42461100	-3.51226400	2.48221300
H	5.72020900	-4.24453900	3.58520700
H	8.92001200	-2.61615800	1.21019800
H	8.14606200	-4.07375100	3.06897800

Fe-int4

125

C	2.45253300	-3.15383400	-1.28010400
N	3.19721800	-2.18864400	-0.46518700
O	0.14963200	0.07509600	2.42576100
C	-5.92514900	0.28691500	0.61745600
Si	2.51743000	-0.96644400	0.66327500
C	4.48732500	-1.81544300	-0.78452400
N	-4.61045600	-0.31279500	0.64263900
Si	-2.23184700	-0.38102400	1.23368300
N	4.85484900	-0.68388300	-0.27672300
C	-0.32702700	-0.72886700	1.62029500
C	-4.36333100	-1.56517200	0.44654200
C	6.22691200	-0.21724600	-0.27025700
N	-3.03738700	-1.89467900	0.65688900
C	-2.46152900	-3.15988700	0.20387400
C	5.34116200	-2.72475500	-1.60514200
C	5.77637900	-2.32748400	-2.87544400
C	5.73373200	-3.96980400	-1.09497100
C	6.59391400	-3.16973800	-3.63050900

H	5.46991100	-1.36147700	-3.26766000
C	6.55651700	-4.80650100	-1.84881600
H	5.39443800	-4.27226000	-0.10808500
C	6.98555500	-4.40871400	-3.11809800
H	6.92462400	-2.85831900	-4.61737800
H	6.86153000	-5.76858100	-1.44671400
H	7.62368000	-5.06280200	-3.70574200
C	-5.36184300	-2.60585800	0.06557000
C	-5.64839400	-3.66888300	0.93349000
C	-6.05353300	-2.50083100	-1.14835600
C	-6.60702000	-4.62003400	0.58518800
H	-5.12557900	-3.73818800	1.88339200
C	-7.00714900	-3.45774600	-1.49803000
H	-5.84562800	-1.66387700	-1.80813900
C	-7.28368000	-4.51929900	-0.63353800
H	-6.82670200	-5.43865000	1.26491300
H	-7.53578700	-3.37194300	-2.44336400
H	-8.02748500	-5.26312100	-0.90555400
H	6.65899900	-0.35672500	0.73004500
H	6.87732900	-0.72356700	-0.99430100
H	6.23714800	0.85802800	-0.47561900
H	2.10221200	-2.70541600	-2.21820700
H	3.08170000	-4.01389200	-1.52046600
H	1.59174500	-3.50671800	-0.71548900
H	-6.13370700	0.74557100	1.59242700
H	-5.93954200	1.08638500	-0.13131800
H	-6.73429800	-0.41995900	0.39394600
H	-2.34916800	-3.18816600	-0.88741800
H	-1.47898100	-3.28505800	0.65575900
H	-3.08605800	-4.00435300	0.50650600
P	1.97261400	0.94519700	-0.44750900
P	-1.84154800	1.44875300	-0.10698600
Fe	0.11642900	1.25768900	-1.42613600
C	-0.50051600	1.54915900	-3.05885100

C	0.26393100	3.02837900	-1.09819600
C	-0.17063300	-0.49316700	-1.54069800
O	0.32222700	4.15275900	-0.86217000
O	-0.80494200	1.75297000	-4.15410800
O	-0.35073800	-1.62817700	-1.66176500
C	-3.31694600	1.79138300	-1.18524500
C	-3.84472000	0.91960400	-2.17193200
C	-3.93592100	3.05453700	-0.99686200
C	-4.98225900	1.29791700	-2.89230200
C	-5.07139600	3.39061700	-1.74622500
C	-5.62107300	2.52365400	-2.69017200
H	-5.37799000	0.61426000	-3.64186300
H	-5.53878900	4.35975700	-1.58286000
C	3.07772500	2.33010000	0.02555300
C	4.12174900	2.68891000	-0.85912600
C	2.87252400	3.06527600	1.21446800
C	4.98707500	3.72588500	-0.49597200
C	3.75917100	4.10043300	1.52771300
C	4.83308100	4.43417500	0.69913100
H	5.79608100	3.99516600	-1.17234300
H	3.60349900	4.66184400	2.44675000
C	-3.21887700	-0.40880600	-2.52059200
C	-6.87325200	2.88345700	-3.45341700
C	-3.41579700	4.07934400	-0.00830600
C	4.29819300	2.01722200	-2.20271600
C	1.71327400	2.77824300	2.13733600
C	5.80608700	5.52116100	1.08888300
H	5.20961700	2.36772900	-2.69766600
H	4.35347100	0.92867800	-2.10423500
H	3.44980200	2.23538000	-2.86353900
H	5.32302300	6.29248700	1.69818900
H	6.63558400	5.11119300	1.68063100
H	6.24257300	6.00478800	0.20840500
H	0.75040700	2.94975800	1.64178400

H	1.69606600	1.73804800	2.47020700
H	1.75216700	3.41865800	3.02415900
H	-2.36246300	-0.27680800	-3.19103700
H	-2.86348100	-0.94343500	-1.64251600
H	-3.93262900	-1.05264000	-3.04399900
H	-6.84084200	2.50193700	-4.48011800
H	-7.76346500	2.45204400	-2.97596200
H	-7.01979800	3.96780500	-3.49718400
H	-3.98674900	5.01062800	-0.08770100
H	-3.49097200	3.72376700	1.02406200
H	-2.36021700	4.31270000	-0.18182400
Si	3.70598700	-0.95085400	2.71128600
Si	-3.06609400	0.20326200	3.37614000
C	-4.43251600	-1.03840000	3.83752700
C	-3.75649600	1.96743400	3.32625400
C	-1.75878800	0.10934400	4.74909000
C	2.50947100	-1.26892000	4.14403300
C	4.90333800	-2.42187600	2.60752000
C	4.64851300	0.66399200	3.00040700
H	-4.25326100	2.22540500	4.27018000
H	-2.94331800	2.68375400	3.16258700
H	-4.47879800	2.09029600	2.51308000
H	-5.31006800	-0.94943600	3.19130500
H	-4.06138400	-2.06753100	3.75942800
H	-4.75121800	-0.87746500	4.87531900
H	-2.23350600	0.27745400	5.72493500
H	-1.26884500	-0.87060100	4.77493200
H	-0.97817100	0.86066800	4.60240700
H	1.95067500	-2.19920800	3.98815600
H	3.06394200	-1.36059100	5.08678000
H	1.78106100	-0.45933100	4.24113100
H	4.36299900	-3.34728600	2.37749400
H	5.66734900	-2.27548500	1.83856100
H	5.40912500	-2.55849700	3.57185500

H	3.97736500	1.45961000	3.33706800
H	5.40895000	0.51352200	3.77749800
H	5.14548600	1.01406700	2.09194300
C	0.74888400	-1.71917200	1.12199900
O	0.57904000	-2.92246400	1.28368100

Fe-ts2

125

C	-4.14458700	1.32082000	-2.87709800
N	-3.77504500	1.40764600	-1.48032100
O	-0.75893500	1.18208000	0.90529600
C	5.41161800	-0.21702100	1.81974800
Si	-2.18758700	1.41523600	-0.45386900
C	-4.51077400	1.13245700	-0.39583200
N	4.29330000	0.42961100	1.17135000
Si	1.93902000	0.84225600	0.73937000
N	-3.65915000	1.05460200	0.63522500
C	0.21209500	1.54568200	0.14842100
C	4.39114800	1.42388100	0.35097400
C	-3.96853800	1.06232200	2.05015700
N	3.16495700	1.87422800	-0.08855800
C	3.02499900	2.91496700	-1.10424300
H	-4.88597600	1.62399100	2.25323200
H	-4.08247000	0.04327800	2.43399500
H	-3.14006300	1.53780500	2.58161400
H	-3.43395200	0.67129600	-3.40023200
H	-5.15031000	0.90772900	-2.99737800
H	-4.11251400	2.30997000	-3.34657800
H	5.22698100	-0.26057100	2.89988000
H	5.49924800	-1.24751000	1.45810600
H	6.36962000	0.29336700	1.65821300
H	2.01482300	2.90069500	-1.51160400

H	3.21601500	3.91370800	-0.69222300
H	3.72639000	2.74751200	-1.92910300
P	-1.82159800	-0.98217300	-0.84713800
P	1.62466100	-1.39289700	0.28700000
Fe	0.05213700	-1.84277200	-1.39578100
C	0.99050700	-2.88049700	-2.48228500
C	-0.41449700	-3.28291900	-0.43694700
C	0.56544400	-0.45046500	-2.41566400
O	-0.66566900	-4.22160200	0.18301600
O	1.56242000	-3.58858100	-3.19564600
O	0.96664800	0.27927000	-3.21300300
Si	-2.36263900	3.88520200	-0.16939400
Si	1.87645300	1.25212000	3.07441400
C	3.17471100	2.58287800	3.47158200
C	2.25523800	-0.36175500	3.99152000
C	0.19802400	1.87940800	3.70689800
C	-0.85485400	4.71566400	0.64075300
C	-2.61637400	4.63663600	-1.89346600
C	-3.88948700	4.29529100	0.89377600
H	2.32006700	-0.19217000	5.07389400
H	1.45916500	-1.09150200	3.80586100
H	3.19650500	-0.80552500	3.65458400
H	4.18331000	2.24474500	3.21565200
H	2.97481200	3.50252900	2.90824700
H	3.15422400	2.83426800	4.53962600
H	0.26339700	2.08231300	4.78402000
H	-0.10856400	2.80395300	3.20671200
H	-0.58832500	1.13674000	3.54141000
H	0.04335900	4.56531800	0.03136000
H	-1.01762300	5.79653900	0.74299400
H	-0.66079200	4.31087300	1.64029100
H	-1.85728600	4.27092100	-2.59277300

H	-3.60544900	4.37931000	-2.28981800
H	-2.54368100	5.73135000	-1.85568600
H	-3.75720600	3.97276500	1.93226000
H	-4.05704600	5.38051100	0.89928200
H	-4.79758700	3.81895300	0.50657400
C	-0.43299400	1.98088900	-1.17633000
O	-0.00403700	2.66675400	-2.08803200
C	-5.97051300	0.95980700	-0.33024900
C	-6.51132100	-0.08072600	0.44099600
C	-6.82287200	1.82550200	-1.03337400
C	-7.89235700	-0.25621800	0.49733600
H	-5.85131000	-0.76396700	0.96305300
C	-8.20402300	1.65121700	-0.96293300
H	-6.40140100	2.64033800	-1.61433100
C	-8.73935900	0.60882000	-0.20099900
H	-8.30591400	-1.07139000	1.08413300
H	-8.86157000	2.32817900	-1.50039000
H	-9.81593400	0.47165700	-0.15170800
C	5.65595100	2.05376600	-0.12474900
C	6.59054100	1.28200400	-0.82930800
C	5.93534700	3.39956700	0.14803800
C	7.78309400	1.85655300	-1.27005300
H	6.37708600	0.23464100	-1.02237400
C	7.13277700	3.96839800	-0.28524500
H	5.21885900	3.99325400	0.70840300
C	8.05542600	3.19975900	-0.99995800
H	8.50024900	1.25439500	-1.82102500
H	7.34531900	5.01104300	-0.06585300
H	8.98543000	3.64578400	-1.34159200
C	3.26593300	-2.18259500	-0.09072200
C	3.64683200	-3.24659600	0.76861800
C	4.13472600	-1.82923500	-1.15416900

C	4.87953000	-3.88622900	0.58057300
C	5.36221000	-2.48603400	-1.29268200
C	5.75994500	-3.51499000	-0.43531200
H	5.16062300	-4.69237700	1.25561000
H	6.02331800	-2.19329300	-2.10735000
C	-3.05867400	-1.98580500	0.08100200
C	-4.15054000	-2.55863100	-0.61453500
C	-2.94832700	-2.17879400	1.48068100
C	-5.11575100	-3.27925100	0.09588800
C	-3.94643400	-2.89619100	2.15050000
C	-5.03917300	-3.45333500	1.48036400
H	-5.95652600	-3.70738200	-0.44660800
H	-3.85963500	-3.03220300	3.22705600
C	-4.30463500	-2.41172200	-2.11171000
C	-6.08725900	-4.24716700	2.22367400
C	-1.77020800	-1.65284000	2.26471100
C	2.76733700	-3.73216000	1.90373100
C	7.08001300	-4.22177400	-0.63004300
C	3.78571300	-0.79388200	-2.19611200
H	-4.39767400	-1.36116400	-2.40584800
H	-3.43415000	-2.81075100	-2.64495300
H	-5.19593300	-2.93837800	-2.46719900
H	-7.06820000	-4.16607300	1.74228700
H	-5.82773100	-5.31380600	2.25277500
H	-6.18627200	-3.90909900	3.26088300
H	-1.92721300	-1.78072000	3.34078100
H	-0.84324800	-2.17292200	1.99620000
H	-1.58124100	-0.59566000	2.06440900
H	1.76972900	-4.01613000	1.55250100
H	3.21668600	-4.60550700	2.38876300
H	2.62108800	-2.95805800	2.66374100
H	7.86352100	-3.52519300	-0.94949500

H	7.41386700	-4.71117900	0.29100300
H	7.00260400	-4.99722800	-1.40388900
H	3.15708600	-1.23479200	-2.97763300
H	3.24229800	0.05456100	-1.78416500
H	4.68664500	-0.41171000	-2.68695700

Fe-int5

125

C	-2.00464900	3.42410900	0.92317700
N	-2.73321500	2.27012500	0.38535800
O	-1.12510600	0.53130900	1.86918400
C	5.47099300	-1.28979100	1.10928600
Si	-2.56294500	0.58967800	0.96712600
C	-3.89738700	2.43849200	-0.34953500
N	4.26133600	-0.49867800	1.10132200
Si	1.72908700	0.06383400	1.27125500
N	-4.71812400	1.44252100	-0.30907400
C	0.15448600	1.10002400	1.71652300
C	4.24373800	0.78753000	1.06105900
C	-5.92236700	1.38484600	-1.11719200
N	2.98122100	1.36098100	1.12850100
C	2.78402100	2.76334000	0.74292900
C	-4.05192800	3.67997100	-1.16441500
C	-3.03238800	4.05384400	-2.05355800
C	-5.20980200	4.46359800	-1.07510500
C	-3.17892200	5.18750500	-2.85118000
H	-2.12960600	3.45326200	-2.11034600
C	-5.34815900	5.60578600	-1.86455400
H	-5.99454900	4.18252200	-0.37908600
C	-4.33575900	5.96644500	-2.75667600

H	-2.38903500	5.46577900	-3.54328800
H	-6.24540400	6.21287900	-1.78276900
H	-4.44632700	6.85330600	-3.37457000
C	5.43637500	1.68091800	0.95604000
C	5.72137700	2.60320500	1.97203900
C	6.28485500	1.59182000	-0.15582700
C	6.84439200	3.42468100	1.87832300
H	5.06282600	2.66796300	2.83386000
C	7.40187300	2.42299900	-0.25227400
H	6.06061200	0.87270200	-0.93871400
C	7.68395300	3.33887400	0.76400000
H	7.06344500	4.13285800	2.67271900
H	8.05241900	2.35326600	-1.11985600
H	8.55559000	3.98335600	0.68933600
H	-6.78001700	1.77895700	-0.55499900
H	-5.85622700	1.93994200	-2.06275400
H	-6.14706000	0.33881000	-1.34387600
H	-2.56453000	4.34168500	0.73325500
H	-1.88842200	3.33017200	2.00705100
H	-1.01525200	3.52155600	0.47005800
H	5.43325600	-1.99761800	1.94677000
H	5.52069200	-1.87881900	0.18540800
H	6.39575200	-0.70478300	1.19925200
H	3.50074700	3.05913500	-0.02700600
H	1.78205200	2.88966100	0.32905100
H	2.89287800	3.44233600	1.59498100
P	-2.21777300	-0.70360700	-0.86423500
P	1.40789300	-1.26894400	-0.56251400
Fe	-0.36571000	-0.61971800	-1.93046800
C	0.42840500	-0.39895800	-3.50006100
C	-0.52024000	-2.40599600	-2.18401100
C	-0.15472500	1.10808800	-1.54319400

O	-0.59923600	-3.54558400	-2.32036200
O	0.86806100	-0.26171500	-4.55950000
O	0.01992500	2.24087500	-1.40065700
C	2.98929300	-1.39451000	-1.53568600
C	3.64123200	-0.33153000	-2.20341300
C	3.53905800	-2.70132700	-1.64452200
C	4.82210200	-0.57827400	-2.91606200
C	4.71789000	-2.89965200	-2.37143500
C	5.38591000	-1.85035800	-3.00697900
H	5.30892900	0.25487400	-3.42150100
H	5.12795200	-3.90549200	-2.44073200
C	-3.08099700	-2.29520300	-0.52635600
C	-4.19355200	-2.64417900	-1.32335600
C	-2.65729900	-3.16341500	0.50894800
C	-4.90792400	-3.80906400	-1.02460600
C	-3.40418500	-4.31609200	0.77048300
C	-4.54261800	-4.64861300	0.02980400
H	-5.77164500	-4.06679700	-1.63437700
H	-3.08155700	-4.97752800	1.57210900
C	3.12046000	1.08493700	-2.22213000
C	6.68284000	-2.08320800	-3.74430200
C	2.89958500	-3.91051300	-0.99322400
C	-4.59933100	-1.82225600	-2.52585100
C	-1.39687500	-2.91026000	1.30192500
C	-5.35897500	-5.87209900	0.37117900
H	-5.61351500	-2.07283700	-2.85317700
H	-4.55309600	-0.74923800	-2.32453400
H	-3.92031900	-2.01040300	-3.36832300
H	-4.73009800	-6.67579100	0.76923100
H	-6.11177800	-5.63917300	1.13610600
H	-5.89210100	-6.25693300	-0.50458400
H	-0.50797300	-2.97844900	0.66330400

H	-1.36959300	-1.91335700	1.74909900
H	-1.28941800	-3.64176200	2.10913400
H	2.57928100	1.29004700	-3.15385700
H	2.44282400	1.28060300	-1.39772400
H	3.94439400	1.80436600	-2.16132100
H	6.84076600	-1.33322000	-4.52676600
H	7.54052000	-2.02767600	-3.06021900
H	6.70632100	-3.07377900	-4.21164900
H	3.44478400	-4.82218700	-1.26018400
H	2.89534800	-3.82599100	0.09802500
H	1.85640900	-4.03542800	-1.30112200
Si	-4.18412600	-0.10570400	2.52999400
Si	2.04448500	-1.22087400	3.22790200
C	3.36186100	-0.34810900	4.28594400
C	2.53913300	-3.01240500	2.86416000
C	0.44494900	-1.24573200	4.25324600
C	-3.37306700	-1.11230300	3.91604800
C	-4.85225800	1.52633800	3.23570500
C	-5.58854500	-1.12278300	1.77905200
H	2.69318300	-3.57192100	3.79565500
H	1.75121400	-3.51637000	2.29285000
H	3.46134400	-3.05973600	2.27825200
H	4.34328800	-0.36726100	3.80348300
H	3.08937800	0.70084900	4.45505800
H	3.44595000	-0.83292200	5.26697300
H	0.63762200	-1.67938800	5.24321800
H	0.05110500	-0.23337400	4.39495900
H	-0.33904700	-1.83688000	3.77234100
H	-2.47924000	-0.61689800	4.30712400
H	-4.07943200	-1.25604000	4.74382800
H	-3.07536700	-2.10266500	3.55476300
H	-4.06229000	2.10384700	3.73057900

H	-5.27467000	2.14232400	2.43469200
H	-5.64050800	1.33547300	3.97493300
H	-5.22325900	-2.07368400	1.37962300
H	-6.33652700	-1.34244700	2.55233000
H	-6.07914700	-0.57745100	0.96886100
C	0.40574000	2.18313300	2.43649100
O	0.68608300	3.17234800	3.01467600

Fe-ts3

125

C	-2.34357200	-3.08837100	1.81612300
N	-2.89414600	-1.99517100	1.01708200
O	-0.69022700	-2.08835100	-0.56379200
C	5.79734800	0.24548900	-1.79293000
Si	-2.21669200	-1.33395200	-0.52305000
C	-4.21321600	-1.61501300	1.07324000
N	4.55016900	-0.35423200	-1.35483500
Si	1.94714500	-0.85275700	-0.77587000
N	-4.55568400	-0.86567800	0.07450500
C	0.40699200	-1.48389000	0.08579800
C	4.55027200	-1.38393900	-0.58085400
C	-5.88199500	-0.32163700	-0.12151300
N	3.31545100	-1.88838600	-0.22315200
C	3.21254400	-3.00250000	0.71865800
H	-6.41935400	-0.13845500	0.81811600
H	-5.79965500	0.62212900	-0.66350400
H	-6.49224300	-1.00269800	-0.73252500
H	-3.06420700	-3.90869500	1.90783600
H	-1.45105200	-3.46409000	1.31326800
H	-2.05224300	-2.75741200	2.81707300
H	5.62200300	0.77168100	-2.73666900

H	6.15000800	0.98633400	-1.06291200
H	6.60664500	-0.48171700	-1.94735500
H	3.65533100	-2.75656100	1.69249700
H	2.15788000	-3.23482100	0.86841100
H	3.71401300	-3.89720700	0.33426000
P	-1.66958400	0.91866500	-0.10666900
P	1.73323600	0.84924600	0.66217500
Fe	0.02161100	2.35107000	0.01296000
C	-1.17838500	3.66740400	-0.14356300
C	0.75366900	2.61015100	-1.55096300
C	1.01689300	3.44598000	1.01993000
O	1.16604400	2.80572100	-2.61244000
O	-1.81887800	4.61744500	-0.24580300
O	1.61993900	4.18771400	1.66161500
Si	-3.29869400	-2.35611500	-2.36417400
Si	1.68078100	-0.89686400	-3.11979200
C	-0.03324800	-0.25711500	-3.61885700
C	1.81622900	-2.73988900	-3.56215000
C	2.96727100	0.09065500	-4.09151100
C	-2.05360300	-3.37212500	-3.37525400
C	-4.54737500	-3.59242400	-1.63252400
C	-4.21365600	-1.14469900	-3.49732000
H	1.68561900	-2.89665900	-4.64061000
H	2.79738200	-3.13696400	-3.27667200
H	1.04969100	-3.32296000	-3.03978400
H	-0.24382900	-0.51522200	-4.66476200
H	-0.81024400	-0.70186700	-2.99525100
H	-0.09949800	0.82848600	-3.51935500
H	3.95636700	-0.36679200	-4.00887000
H	2.68338000	0.12811100	-5.15151200
H	3.03741100	1.11941300	-3.72376100
H	-1.49982600	-4.05617300	-2.72249200

H	-2.58154400	-3.97194000	-4.12815700
H	-1.32261900	-2.74577100	-3.89348800
H	-4.12174200	-4.15136500	-0.79155200
H	-5.44921700	-3.08494200	-1.27874500
H	-4.84139800	-4.31655600	-2.40286300
H	-3.52241700	-0.60969500	-4.15650300
H	-4.92113500	-1.69364100	-4.13174400
H	-4.77546400	-0.40348900	-2.91972800
C	0.29431000	-1.16409900	1.39732600
O	-0.03041100	-1.16122500	2.53952500
C	3.35988500	1.48550800	1.28169700
C	3.74466600	1.05241400	2.57534300
C	4.24036500	2.31047600	0.54200600
C	4.99324600	1.43153400	3.08402500
C	5.47782100	2.66184600	1.09052900
C	5.88072900	2.22519900	2.35513600
H	5.28101700	1.08899500	4.07623800
H	6.14780100	3.29357500	0.51008600
C	-3.26003800	1.83602400	-0.32624600
C	-3.99374100	2.20070500	0.82724500
C	-3.68229800	2.28746900	-1.59393300
C	-5.13764600	2.98879400	0.68478300
C	-4.84300100	3.06544800	-1.69170500
C	-5.58504600	3.42792700	-0.56604700
H	-5.70065500	3.26385300	1.57482400
H	-5.16403400	3.40816200	-2.67356300
C	-5.07825600	-2.04403000	2.20764700
C	-6.28392500	-2.71738500	1.97111100
C	-4.69729000	-1.75343500	3.52607100
C	-7.09618300	-3.09956800	3.03914300
H	-6.57572300	-2.95082500	0.95179600
C	-5.51715100	-2.12413300	4.59088800

H	-3.76413700	-1.22959900	3.71061800
C	-6.71608300	-2.80057300	4.34948300
H	-8.02534100	-3.62900300	2.84769100
H	-5.21928600	-1.88763900	5.60851300
H	-7.35171600	-3.09389300	5.18040100
C	5.78173700	-2.01937300	-0.01275000
C	6.32199800	-3.18793500	-0.56213000
C	6.42004600	-1.40087200	1.07044500
C	7.49128200	-3.73557400	-0.03095600
H	5.82779900	-3.66338700	-1.40545900
C	7.58443800	-1.95497500	1.60339800
H	5.99988500	-0.49185700	1.49076600
C	8.12245800	-3.12165400	1.05381700
H	7.90845600	-4.64095100	-0.46352700
H	8.07188600	-1.47366900	2.44727500
H	9.03093900	-3.55060600	1.46795400
C	3.88309300	2.83355200	-0.82531400
C	7.24728400	2.56970500	2.89704300
C	2.86305900	0.16543200	3.42968100
C	-2.86503100	2.02773700	-2.83626600
C	-3.55751100	1.77581100	2.21077700
C	-6.84944900	4.24404300	-0.69075400
H	-6.94302700	4.96393800	0.13002200
H	-6.87942500	4.79787400	-1.63479400
H	-7.73880000	3.60035800	-0.65871500
H	-1.98967300	2.69033800	-2.87305400
H	-2.47897700	1.00754500	-2.86817700
H	-3.45200400	2.20104900	-3.74339900
H	-2.66350800	2.32586500	2.53172600
H	-4.34905700	1.95766300	2.94487700
H	-3.29553800	0.71421800	2.24400000
H	3.51322300	2.02963000	-1.46566400

H	3.10593400	3.60378900	-0.76540000
H	4.75215500	3.28554900	-1.31268000
H	8.00107200	1.85635700	2.53662900
H	7.56759600	3.56706700	2.57647200
H	7.26572600	2.53987300	3.99161200
H	2.79496200	-0.84887900	3.01929200
H	3.26522000	0.08711200	4.44524200
H	1.83511600	0.53546400	3.49176800

Fe-int6

125

C	-2.27118600	2.93829300	-1.92628000
N	-2.88684700	2.04733700	-0.94393500
O	-0.56856300	2.15408400	0.51063100
C	5.88081200	-0.66523100	1.60732300
Si	-2.07023100	1.37052600	0.49369700
C	-4.22410200	1.72444700	-0.94618700
N	4.64639200	0.05790000	1.35988500
Si	2.03273200	0.82343400	1.06000800
N	-4.58435600	0.96533700	0.03461000
C	0.53759900	1.38609200	0.12520900
C	4.66373300	1.20280900	0.76905600
C	-5.94748600	0.53541900	0.25480300
N	3.44455500	1.82817300	0.59036200
C	3.33819600	3.04744100	-0.21323700
H	-6.55227600	0.50019000	-0.66140100
H	-5.93170200	-0.46338300	0.69756700
H	-6.45466800	1.20420100	0.96608200
H	-2.90662400	3.81407800	-2.09761300
H	-1.30854600	3.26191600	-1.53332400
H	-2.08476400	2.42800600	-2.87427600
H	5.73002300	-1.33753400	2.45821700

H	6.14869600	-1.28485900	0.74048400
H	6.73861600	-0.01461000	1.82752500
H	3.76994500	2.91300900	-1.21251900
H	2.28139400	3.29315100	-0.32806200
H	3.84685000	3.88954600	0.26875400
P	-1.62147600	-0.95525200	0.17305800
P	1.65843300	-0.71878900	-0.55878100
Fe	-0.00449500	-2.37236100	-0.10341700
C	-1.21121600	-3.68136400	-0.01647500
C	0.88350100	-2.93916400	1.31526600
C	0.77410400	-3.20674800	-1.47922200
O	1.36892900	-3.31678600	2.29272400
O	-1.89752800	-4.60403600	0.01541700
O	1.24586700	-3.75448000	-2.37562700
Si	-3.09838700	2.18478000	2.46407200
Si	1.83578900	0.42420200	3.36510100
C	0.10473600	-0.27272700	3.70585900
C	2.03419200	2.11967600	4.19754100
C	3.11639700	-0.77623200	4.06526500
C	-1.77421300	3.00343100	3.55345300
C	-4.27029800	3.57210400	1.89447100
C	-4.06692500	0.91630900	3.48296000
H	1.93665200	2.03128100	5.28714400
H	3.02180400	2.54282600	3.97963000
H	1.27758900	2.83052500	3.84758100
H	-0.14415100	-0.19811600	4.77200300
H	-0.65839200	0.26751500	3.14038600
H	0.04732300	-1.32573500	3.41762200
H	4.12018600	-0.34720000	4.00708400
H	2.88763100	-1.00034500	5.11534200
H	3.11949400	-1.71910800	3.50923300
H	-1.14164800	3.66674200	2.95314500

H	-2.25017500	3.60279600	4.34045500
H	-1.12196900	2.27191000	4.03863000
H	-3.82876300	4.17675700	1.09470200
H	-5.21493800	3.16237900	1.52655500
H	-4.48840900	4.23725600	2.73958300
H	-3.40658100	0.33235800	4.13185500
H	-4.78755700	1.43706400	4.12631800
H	-4.61803600	0.22384500	2.84014300
C	0.51415800	0.70862100	-1.09983000
O	-0.27691200	0.73492800	-2.06347800
C	3.13548200	-1.16183500	-1.59291800
C	3.42066400	-0.48293800	-2.80319100
C	4.05895900	-2.12209900	-1.10557400
C	4.61042800	-0.77789600	-3.48306000
C	5.23350500	-2.37357600	-1.81940600
C	5.53506100	-1.70819500	-3.00964100
H	4.82099100	-0.24954300	-4.41061700
H	5.93228300	-3.11227100	-1.43175400
C	-3.26466100	-1.78251800	0.32094900
C	-4.00868200	-1.98187100	-0.86414500
C	-3.71424600	-2.33254700	1.53686300
C	-5.20312400	-2.70153800	-0.79923200
C	-4.92520600	-3.03570700	1.55818300
C	-5.68532400	-3.23046400	0.40318300
H	-5.77761600	-2.84868000	-1.71201100
H	-5.27283000	-3.45291700	2.50136200
C	-5.11526400	2.24928800	-2.02482200
C	-6.16264400	3.12330400	-1.70700900
C	-4.93061200	1.84943300	-3.35553400
C	-7.01090800	3.59772300	-2.70826700
H	-6.30422400	3.43493600	-0.67621600
C	-5.78636400	2.31587000	-4.35328200

H	-4.12132700	1.16968400	-3.60355500
C	-6.82561000	3.19300700	-4.03220300
H	-7.81643300	4.28113200	-2.45403700
H	-5.64003700	1.99646600	-5.38141600
H	-7.48871600	3.55936100	-4.81110100
C	5.89177800	1.85982600	0.21940000
C	6.49760700	2.94117900	0.86958700
C	6.45267800	1.35582500	-0.96161500
C	7.65742600	3.51326900	0.34365600
H	6.06167400	3.32952000	1.78635000
C	7.60624300	1.93610000	-1.48975400
H	5.97890500	0.51711700	-1.46245700
C	8.21148000	3.01373300	-0.83774200
H	8.12671200	4.34944500	0.85491000
H	8.03200400	1.54553300	-2.41041300
H	9.11179800	3.46301900	-1.24807300
C	3.82196700	-2.90492300	0.16241700
C	6.83753000	-1.95723500	-3.73099400
C	2.51888100	0.57122100	-3.40139500
C	-2.87724500	-2.26439500	2.79133600
C	-3.51733300	-1.47657100	-2.20155500
C	-7.00558700	-3.96172800	0.45002700
H	-7.16047400	-4.56591100	-0.45074800
H	-7.06849200	-4.62374200	1.31991200
H	-7.84405500	-3.25512200	0.51363900
H	-2.09096600	-3.03160100	2.77405200
H	-2.37139400	-1.30326200	2.89774500
H	-3.48420800	-2.43117400	3.68638800
H	-2.78398500	-2.17052100	-2.63420600
H	-4.34538800	-1.37749100	-2.91088500
H	-3.01206500	-0.50970400	-2.12158700
H	3.53242300	-2.24959500	0.98759000

H	3.03512400	-3.65473300	0.02590400
H	4.72809000	-3.43790900	0.46404400
H	7.64868900	-1.36466600	-3.28625100
H	7.13603300	-3.00950600	-3.66934200
H	6.77124200	-1.68133700	-4.78843900
H	1.47972000	0.24352600	-3.48327600
H	2.50213700	1.47661600	-2.78292100
H	2.87540900	0.85528900	-4.39622800

Fe-ts4

125

C	1.92285300	-3.57825100	0.58861400
N	2.67764600	-2.31982900	0.50030900
O	0.67523200	-1.27671000	2.05202500
C	-5.62495400	1.70476500	1.25627200
Si	2.09679900	-0.89187400	1.42416200
C	3.94252300	-2.32361300	-0.05961000
N	-4.51259100	0.77670800	1.18328000
Si	-2.12677100	-0.10852200	1.23907100
N	4.74729300	-1.37019000	0.26322400
C	-0.73298900	-1.03056700	0.48534500
C	-4.68867500	-0.50185000	1.17854800
C	6.06609800	-1.24132400	-0.32495400
N	-3.52005000	-1.23387100	1.20738200
C	-3.49101300	-2.66655700	0.92086200
H	6.17730900	-1.71145600	-1.31241800
H	6.30275100	-0.17797400	-0.42538800
H	6.82383800	-1.68067900	0.33979700
H	2.54824600	-4.37765100	1.00470200
H	1.07765200	-3.40910600	1.25668700
H	1.53798400	-3.89948900	-0.38210500
H	-5.30479500	2.60097900	1.79621900

H	-5.93139500	2.01588900	0.24914700
H	-6.50696300	1.29121500	1.76300300
H	-4.03012500	-2.89795400	-0.00537200
H	-2.45097000	-2.97384100	0.80376600
H	-3.93653300	-3.24894700	1.73404300
P	1.78268000	0.85340800	-0.27340900
P	-1.54119700	0.48812800	-0.84125200
Fe	0.22362800	1.96982000	-1.30338400
C	1.50920300	3.03560100	-1.92247500
C	-0.53041800	3.23324500	-0.33447200
C	-0.60662600	2.10460000	-2.88998200
O	-0.94285000	4.06850900	0.34692100
O	2.24070300	3.78526700	-2.39669400
O	-1.10195700	2.19500400	-3.92505600
Si	3.64396000	-0.37955300	3.14263000
Si	-1.67358700	0.93055400	3.29607500
C	-0.12172900	1.98577900	3.14325800
C	-1.40316100	-0.48662200	4.51725100
C	-3.13410200	2.01282900	3.81840300
C	2.70837300	0.28610200	4.65955100
C	4.29065200	-2.11470000	3.57483100
C	5.10593800	0.77825000	2.81313400
H	-1.11710000	-0.09714000	5.50269500
H	-2.30673400	-1.09507400	4.64045100
H	-0.59667500	-1.12131900	4.13644600
H	0.14377200	2.41784600	4.11575100
H	0.69679200	1.34435900	2.81493200
H	-0.24024700	2.80148100	2.42590500
H	-4.06264200	1.43708500	3.88039300
H	-2.93485000	2.45791800	4.80180200
H	-3.29157700	2.82698100	3.10282500
H	1.80677100	-0.30157900	4.86249300

H	3.35668900	0.23855500	5.54461800
H	2.40517100	1.33001300	4.52590500
H	3.46975000	-2.79594200	3.82653000
H	4.84122600	-2.53581600	2.72841000
H	4.96586600	-2.06357300	4.43909700
H	4.83045600	1.82950000	2.93841000
H	5.90398200	0.55379600	3.53364100
H	5.49827800	0.64237100	1.80440500
C	-0.29021300	-1.50991300	-0.65434000
O	0.32188800	-2.04304200	-1.53090600
C	-3.06166400	0.43345400	-1.91747100
C	-3.41459700	-0.71054700	-2.66888600
C	-3.94120800	1.54605900	-1.88769900
C	-4.64999300	-0.73740300	-3.32740000
C	-5.15777000	1.47303000	-2.57258500
C	-5.54292400	0.33379300	-3.28349500
H	-4.91717600	-1.62596500	-3.89545400
H	-5.82195000	2.33471100	-2.54951200
C	3.42140000	1.68740600	-0.44634400
C	4.25647700	1.34056600	-1.53076700
C	3.78883400	2.74548500	0.41265600
C	5.46178000	2.02558300	-1.70491900
C	5.00643600	3.39968900	0.20374800
C	5.86308000	3.05003000	-0.84248900
H	6.10536300	1.75005100	-2.53844600
H	5.29103800	4.20638400	0.87655300
C	4.24982600	-3.41340400	-1.04319000
C	5.33620900	-4.27505300	-0.84961400
C	3.45925400	-3.55070600	-2.19418200
C	5.63080100	-5.26206900	-1.79169800
H	5.94713500	-4.17107300	0.04278300
C	3.76332200	-4.52773100	-3.14151700

H	2.60156100	-2.89783600	-2.32997600
C	4.84782300	-5.38685800	-2.94150400
H	6.47187400	-5.93071900	-1.62880300
H	3.14960500	-4.62333700	-4.03332000
H	5.07990400	-6.15191400	-3.67766200
C	-6.00142300	-1.20782700	1.11976300
C	-6.44309400	-2.00795200	2.18129900
C	-6.80360400	-1.05784600	-0.01939200
C	-7.67995700	-2.64882300	2.10541800
H	-5.82041500	-2.11968100	3.06479500
C	-8.03381700	-1.71079300	-0.09711800
H	-6.45130900	-0.43983300	-0.83965900
C	-8.47456800	-2.50450300	0.96487700
H	-8.02214400	-3.26204600	2.93436800
H	-8.64850900	-1.59891800	-0.98628800
H	-9.43480400	-3.00934700	0.90488900
C	-3.59715000	2.83539700	-1.18277000
C	-6.89167400	0.25459200	-3.95695200
C	-2.51338100	-1.91464400	-2.80158700
C	2.87375200	3.22165100	1.51562300
C	3.84767800	0.29275600	-2.53904700
C	7.19359900	3.74041200	-1.02421000
H	7.47897200	3.79009000	-2.08071300
H	7.17433900	4.76103000	-0.62733800
H	7.99142600	3.20023300	-0.49695800
H	1.98267000	3.71836300	1.11098700
H	2.50953300	2.39651200	2.13345800
H	3.38263300	3.93357800	2.17271600
H	3.10596700	0.69636200	-3.24188300
H	4.70774400	-0.05268900	-3.12101000
H	3.39106400	-0.57836900	-2.06273100
H	-3.25522800	2.65911100	-0.16000900

H	-2.80648600	3.37611800	-1.71514600
H	-4.46610800	3.49763300	-1.13569500
H	-7.65134200	-0.13542200	-3.26545100
H	-7.23319000	1.24004200	-4.29081000
H	-6.86924200	-0.41329100	-4.82426900
H	-1.49449100	-1.63509300	-3.08193300
H	-2.43970800	-2.47360300	-1.86260600
H	-2.89831100	-2.59939900	-3.56328300

Fe-int7

125

C	3.33196800	-2.06469100	2.20060000
N	3.63237900	-1.09401800	1.14487100
O	1.53859000	0.47229800	2.14801000
C	-3.42240100	2.76180600	0.64625600
Si	2.72905800	0.46899500	1.10521000
C	4.65039700	-1.33114300	0.25274500
N	-3.45834000	1.42041400	1.19963100
Si	-2.37089300	0.26488500	2.10788900
N	4.90814000	-0.44346300	-0.65198800
C	-1.37005300	-0.70645200	0.95192400
C	-4.51920300	0.61142100	1.37345800
C	5.95946100	-0.66278300	-1.62705100
N	-4.09509300	-0.40818300	2.13764800
C	-4.70509700	-1.72162500	2.27873600
C	5.41068700	-2.62038800	0.40140800
C	5.00629600	-3.76633300	-0.29348400
C	6.54842000	-2.67531800	1.21630900
C	5.73053700	-4.95353000	-0.17187100
H	4.11902300	-3.72585200	-0.91795600
C	7.27324900	-3.86220400	1.33543900
H	6.86172700	-1.78520200	1.75624900
C	6.86498500	-5.00415400	0.64181200

H	5.40780000	-5.83953400	-0.71239200
H	8.15556400	-3.89515000	1.96931000
H	7.42839700	-5.92880900	0.73507600
C	-5.86224500	0.76906500	0.80548600
C	-6.99193100	0.40576400	1.55698000
C	-6.01574000	1.22341200	-0.51378100
C	-8.26127600	0.50325900	0.99339500
H	-6.87361400	0.06654200	2.58082500
C	-7.28729600	1.30491700	-1.07586300
H	-5.14347700	1.47538800	-1.10354600
C	-8.40924000	0.94719100	-0.32431500
H	-9.13396500	0.23116100	1.57919600
H	-7.40048400	1.64060200	-2.10211200
H	-9.39989500	1.01311400	-0.76477500
H	6.47692900	0.28640600	-1.81451500
H	6.71282100	-1.40731400	-1.33370000
H	5.53285800	-0.98664100	-2.58801700
H	3.00860000	-3.02475400	1.78492100
H	4.19996600	-2.23749400	2.84835200
H	2.51569000	-1.65619100	2.79481100
H	-2.75880100	3.38368900	1.25199900
H	-3.03448100	2.74110700	-0.37712600
H	-4.42194200	3.20433100	0.64957900
H	-5.28315600	-1.98411200	1.38826200
H	-3.90836800	-2.45976500	2.39702100
H	-5.35064800	-1.76904700	3.16198900
P	1.85581300	0.49657000	-1.07888600
P	-1.54465100	-0.40735200	-0.88952700
Fe	0.43816900	-0.83233600	-1.97292800
C	-0.16196900	-2.03571300	-3.12970900
C	0.08939800	0.49044900	-3.14013200
C	1.14812700	-2.17890800	-1.02369800

O	-0.12655000	1.30965900	-3.92257400
O	-0.56439500	-2.81078500	-3.88876200
O	1.64693800	-3.11707500	-0.57191200
C	-2.95837000	-1.50986400	-1.37217000
C	-3.24375100	-2.79701500	-0.85720800
C	-3.88726300	-0.92487500	-2.27911200
C	-4.48835000	-3.38651000	-1.12361700
C	-5.10859500	-1.55341500	-2.52487600
C	-5.44954900	-2.76926300	-1.92410700
H	-4.70122800	-4.36893600	-0.70458200
H	-5.81832800	-1.07900100	-3.20039300
C	1.88970000	2.25726100	-1.62755000
C	2.73274500	2.64623300	-2.69089400
C	1.08440500	3.22277600	-0.97920600
C	2.78079800	3.99246400	-3.06625700
C	1.17346400	4.55917900	-1.38214800
C	2.02091200	4.96781000	-2.41610400
H	3.43691100	4.28666500	-3.88334700
H	0.55659900	5.30073800	-0.87725500
C	-2.24398300	-3.60320700	-0.05989700
C	-6.80955000	-3.38523100	-2.14860100
C	-3.57433200	0.35641600	-3.02312700
C	3.57805100	1.63574000	-3.42919300
C	0.10952700	2.83670800	0.10880300
C	2.12969800	6.42401200	-2.80199300
H	4.29200800	2.12980100	-4.09597700
H	4.13190600	1.00445000	-2.72884400
H	2.95360400	0.96857700	-4.03734600
H	1.19082900	6.95909600	-2.62145000
H	2.90952300	6.92919800	-2.21632400
H	2.39095900	6.54084000	-3.85937700
H	0.59695200	2.31735700	0.94002600

H	-0.39878900	3.72211900	0.50523600
H	-0.64996200	2.14646200	-0.28178800
H	-1.21819200	-3.41359200	-0.38630500
H	-2.28267200	-3.38305200	1.01227200
H	-2.43955400	-4.67388800	-0.17895500
H	-6.84594200	-4.42224400	-1.79928300
H	-7.58668100	-2.82536200	-1.61080400
H	-7.08455100	-3.37506600	-3.20989000
H	-4.45119400	0.71721200	-3.57087500
H	-3.22090100	1.14855800	-2.35367100
H	-2.77177100	0.20293400	-3.75528500
Si	4.26646400	2.23727600	1.38257200
Si	-1.46420100	0.97645700	4.16352000
C	-2.93911800	1.30322900	5.31005000
C	-0.43881400	2.52548400	3.84835900
C	-0.34726200	-0.41436300	4.77422000
C	5.62781300	1.50923400	2.49988700
C	5.10041800	2.99428100	-0.14162300
C	3.36029300	3.63597100	2.30180400
H	-0.09593400	2.94364800	4.80357600
H	0.43467000	2.21928500	3.26128700
H	-0.98315300	3.31068400	3.31192900
H	-3.60014400	2.07750600	4.90276200
H	-3.53647000	0.39791400	5.46746800
H	-2.58575800	1.64823300	6.28981500
H	0.04776000	-0.16718600	5.76785100
H	-0.85310000	-1.38392700	4.84321200
H	0.49203300	-0.48803900	4.07247000
H	6.36062700	2.27236300	2.79260900
H	5.21014500	1.07074300	3.41384200
H	6.15910400	0.71716400	1.95878400
H	5.50563200	2.20906600	-0.78580100

H	4.39515300	3.58736400	-0.73126000
H	5.92400100	3.65136800	0.16895800
H	2.83184100	3.25476400	3.18242700
H	4.05492600	4.42181100	2.62631500
H	2.61593500	4.09625500	1.64020200
C	-0.48142100	-1.53227400	1.46459000
O	0.25041900	-2.33543100	1.89008600

Fe-int8

125

Fe	0.75144700	-2.41261500	1.56888100
P	1.58720200	-1.48335400	-0.15140800
Si	-2.70680700	0.27556500	1.50699500
O	-1.01675600	-0.27456600	2.40224400
N	-2.22146000	2.03057100	1.49746100
C	-3.05413900	3.58998800	-0.28017100
P	-2.00501500	-2.19554400	-0.74995300
Si	-4.15314500	-0.44184200	3.20915800
O	-1.29567200	-4.36040700	2.38506200
N	-3.80622400	1.25786900	0.27498100
C	-4.16444600	4.43787900	-0.35815300
H	-5.06381600	4.21091900	0.20701400
O	2.79914000	-4.52670700	1.30174600
C	-4.10242500	5.58206400	-1.15521400
H	-4.95878000	6.24814400	-1.21042600
O	1.64548100	-1.36970300	4.15073300
C	-2.94421300	5.86602700	-1.88351500
H	-2.90266900	6.75434300	-2.50798800
C	-1.84239700	5.00775800	-1.81501700
H	-0.94620400	5.22232300	-2.39099300
C	-1.88720400	3.86983700	-1.01082500
H	-1.05538900	3.16909200	-0.95611200

C	-3.06515900	2.33707600	0.50321300
C	-1.12373800	2.84165100	1.98404400
C	-0.73070500	-1.13376400	1.48558100
C	-1.84193800	-1.08961300	0.50984600
C	-3.56032600	-2.13193500	3.82837700
H	-4.19598700	-2.49927300	4.64344900
H	-2.53357400	-2.05925200	4.20453600
H	-3.56426700	-2.88460200	3.03237700
C	-5.02866500	1.21907900	-0.49966600
C	3.08734700	-2.14037300	-0.98110600
C	4.36746200	-2.10516800	-0.38714100
C	5.46602200	-2.55541600	-1.12642700
H	6.45346700	-2.52253400	-0.66884200
C	5.33657600	-3.04543100	-2.42838300
C	4.05736500	-3.09245000	-2.98907300
H	3.93277300	-3.47798200	-3.99922300
C	2.93214500	-2.64548900	-2.29264100
C	-3.58184000	-1.72687800	-1.58664500
C	-3.54563000	-0.78937500	-2.64218600
C	-4.71442600	-0.54891500	-3.37102600
H	-4.68744300	0.17897600	-4.17989500
C	-5.91582200	-1.20216300	-3.08033400
C	-5.93047400	-2.12162500	-2.02626200
H	-6.85845500	-2.63433200	-1.78016600
C	-4.78062700	-2.40702700	-1.28115900
C	-5.92463700	-0.55809000	2.53686400
H	-6.60428600	-0.96389600	3.29654400
H	-5.98453100	-1.20332800	1.65320100
H	-6.29623800	0.43188500	2.24798700
C	-4.08783000	0.81969600	4.62433000
H	-4.75121900	0.52285300	5.44636800
H	-4.39568200	1.81488300	4.28305200

H	-3.07084900	0.90309700	5.02467000
C	-0.49352600	-3.59260800	2.07392600
C	2.01956000	-3.68679200	1.40838300
C	1.26554200	-1.80733600	3.15490400
C	4.59741400	-1.63524600	1.03125000
C	6.54713200	-3.48125300	-3.21926700
C	1.57139500	-2.71204700	-2.95119600
C	-2.27335600	-0.04900000	-2.98000800
C	-7.15829500	-0.93976600	-3.89824700
C	-4.84388400	-3.42071400	-0.15877000
H	6.95720900	-2.64538300	-3.80197300
H	7.34636700	-3.84422800	-2.56403800
H	6.29819300	-4.27884800	-3.92771100
H	1.09800800	-1.72273000	-3.00595500
H	1.64766700	-3.09878400	-3.97248900
H	0.88104700	-3.35664000	-2.39370800
H	-5.83926700	-3.86940400	-0.08539200
H	-4.12178300	-4.23437900	-0.30774200
H	-4.60966500	-2.96712100	0.81119300
H	-7.18555700	0.09176200	-4.26582000
H	-7.19802900	-1.59830500	-4.77610200
H	-8.06814700	-1.11916500	-3.31557200
H	-1.83466200	0.45469600	-2.11006000
H	-1.50660400	-0.74062000	-3.35730000
H	-2.44473700	0.69910900	-3.75952500
H	-4.89472000	1.69697500	-1.47516100
H	-5.31275500	0.18116000	-0.66168200
H	-5.85099600	1.72225400	0.02681000
H	-0.80144200	2.43824200	2.94553400
H	-0.28842600	2.80545200	1.28376300
H	-1.45754500	3.87509200	2.12598400
H	4.50438500	-2.46873800	1.73902000

H	5.60571800	-1.22423800	1.14214900
H	3.88222000	-0.86819800	1.33060900
Si	1.33199600	0.79721100	-0.67545800
O	-0.17302300	1.15219800	-0.34386900
Si	2.07034200	1.53675000	-2.79360100
C	0.52854200	1.72254800	-3.89755800
C	2.72744100	3.31058000	-2.55367200
C	3.30425100	0.46929200	-3.76183900
H	0.78088400	2.27866800	-4.81038400
H	0.13393100	0.74748400	-4.20236900
H	-0.27578700	2.25788900	-3.38128700
H	1.97545600	3.93140000	-2.04986800
H	3.64329300	3.33996100	-1.95782700
H	2.93523600	3.76935100	-3.52934300
H	3.79223700	1.07182400	-4.53950600
H	4.06995600	0.04815500	-3.10881000
H	2.79075300	-0.35990500	-4.26086700
N	2.46978700	1.40065400	0.59999800
C	2.01228500	1.43795500	1.99351800
C	3.81396100	1.62028900	0.33704200
H	1.02294400	0.98641100	2.04818900
H	2.68309600	0.87195300	2.65092800
H	1.94243300	2.46100200	2.38048200
C	4.53855400	2.51731000	1.29917900
C	5.62107400	2.04944200	2.05329900
C	4.13301000	3.85212400	1.43748500
C	6.28852000	2.90237400	2.93432800
H	5.93415700	1.01460500	1.94994700
C	4.80618800	4.70750400	2.30896600
H	3.28755200	4.20976500	0.85554800
C	5.88406000	4.23290800	3.06215800
H	7.12390600	2.52774700	3.51978500

H	4.48874400	5.74254800	2.40400300
H	6.40528800	4.89745300	3.74592900
N	4.32862600	1.08758100	-0.71442800
C	5.67352000	1.37858600	-1.15996400
H	5.63217500	1.76706800	-2.18648300
H	6.23375300	2.09698700	-0.54560900
H	6.24235700	0.44005300	-1.20343000

Fe-ts6

125

Fe	-0.78814545	-2.61292921	-1.52992872
P	-1.52165848	-1.52661559	0.18731799
Si	2.76084537	-0.15242163	-1.59068972
O	1.00907630	-0.51793723	-2.34324136
N	2.46832715	1.63307365	-1.52858178
C	3.91477770	3.22146094	-0.23804590
P	1.78390917	-2.15343895	1.01789121
Si	3.99909492	-1.13732387	-3.32256263
O	1.14786733	-4.79887243	-1.72378728
N	4.15449597	0.73506579	-0.55919806
C	5.22118320	3.71732005	-0.35201854
H	5.97911621	3.13500463	-0.86703090
O	-3.11209942	-4.40527932	-1.24357863
C	5.53588399	4.96771526	0.17856682
H	6.54494428	5.35761699	0.08040461
O	-1.23220569	-1.92339548	-4.34800734
C	4.55463529	5.71390981	0.83701499
H	4.80393039	6.68522063	1.25553528
C	3.25601522	5.21201815	0.96144978

H	2.49685279	5.78843493	1.48262681
C	2.92481446	3.97032470	0.42046507
H	1.92358783	3.55896107	0.53209297
C	3.55047804	1.89629889	-0.76925926
C	1.55225098	2.57575939	-2.14493224
C	0.71941468	-1.41306971	-1.44310443
C	1.83034455	-1.35190660	-0.46620329
C	2.89677248	-2.40913412	-4.19122986
H	3.41192505	-2.86311632	-5.04673301
H	1.97185506	-1.94468516	-4.54917083
H	2.61536521	-3.21420874	-3.50231156
C	5.39554942	0.51104704	0.14972539
C	-3.09463116	-2.16128218	0.92989884
C	-4.36313594	-1.87812196	0.37052983
C	-5.51881668	-2.20305831	1.08691533
H	-6.48955723	-1.97301742	0.64938278
C	-5.46859826	-2.83058142	2.33494328
C	-4.21133677	-3.15893120	2.84752841
H	-4.14588093	-3.67438473	3.80457649
C	-3.03025673	-2.83989478	2.17119289
C	3.24743992	-1.51031940	1.94162071
C	3.18235932	-0.24350497	2.56493587
C	4.24936531	0.16625100	3.36998360
H	4.20109070	1.14456306	3.84474539
C	5.37329238	-0.63931465	3.57834965
C	5.41448458	-1.89098828	2.95636386
H	6.28359473	-2.52979576	3.10178011
C	4.36696680	-2.34592928	2.14813003
C	5.52141270	-2.00080539	-2.58845498
H	6.09211977	-2.51215080	-3.37370786
H	5.22832651	-2.75314039	-1.84646267
H	6.19185380	-1.28818601	-2.09565156

C	4.54947338	0.22020352	-4.52866299
H	5.17081464	-0.19539351	-5.33167711
H	5.13554600	0.99058556	-4.01347024
H	3.68504517	0.71064744	-4.99063647
C	0.36851268	-3.94484142	-1.67091619
C	-2.21721443	-3.68487584	-1.35331582
C	-1.04145669	-2.22706227	-3.25062904
C	-4.50330501	-1.27174514	-1.00567905
C	-6.73021170	-3.12772882	3.11061902
C	-1.70162219	-3.24143029	2.76914328
C	1.99535558	0.66606885	2.36436141
C	6.49879432	-0.18083544	4.47522262
C	4.46729283	-3.69788526	1.47572686
H	-6.98785958	-2.29849570	3.78392446
H	-7.58591194	-3.28101979	2.44356843
H	-6.61905919	-4.02431883	3.73064003
H	-1.01494526	-2.38878167	2.85184525
H	-1.82917837	-3.67588637	3.76612991
H	-1.18670626	-3.97795091	2.13874537
H	5.35692224	-4.24171228	1.80772987
H	3.59250226	-4.32581126	1.69139806
H	4.52461970	-3.60003406	0.38408538
H	6.60925701	0.90878130	4.45390911
H	6.31266833	-0.46802861	5.51867150
H	7.45460462	-0.62732517	4.18078880
H	1.91058614	1.02274243	1.33524206
H	1.04579730	0.15353073	2.56827782
H	2.04698594	1.54512772	3.01268587
H	5.50779700	1.21044256	0.98306747
H	5.39665727	-0.50086054	0.55431123
H	6.26055814	0.61882640	-0.51845209
H	1.03525184	2.05696073	-2.95445208

H	0.81231629	2.91987907	-1.42044225
H	2.09894452	3.42896560	-2.56156980
H	-4.22433863	-1.98988493	-1.78541354
H	-5.53880682	-0.96965805	-1.19256623
H	-3.85914636	-0.39951384	-1.13201611
Si	-1.47222883	2.10010404	0.94130703
O	0.02340432	2.42776830	0.68833635
Si	-2.36581886	2.04083342	3.12659550
C	-0.88090406	2.41622797	4.24701590
C	-3.61366911	3.45032675	3.35142765
C	-3.07096254	0.34328483	3.52684181
H	-1.21248773	2.48125742	5.29157290
H	-0.10185415	1.65044967	4.18436094
H	-0.41841756	3.37332995	3.97899976
H	-3.11719627	4.42411035	3.26672051
H	-4.42067415	3.41095234	2.61683874
H	-4.05767653	3.39019785	4.35345914
H	-3.38723102	0.29663004	4.57671423
H	-3.91782166	0.10508834	2.88005171
H	-2.30943647	-0.42408743	3.36445862
N	-2.50970004	1.98938957	-0.45320086
C	-1.90468131	1.81961490	-1.78885985
C	-3.91535465	2.04804911	-0.34260285
H	-1.00399037	1.20988923	-1.69925184
H	-2.60091000	1.29423764	-2.44733639
H	-1.64430725	2.77723690	-2.25232468
C	-4.61904028	2.73837664	-1.47080144
C	-5.68449251	2.13384938	-2.15049235
C	-4.20753517	4.02520535	-1.85003206
C	-6.32885492	2.80520426	-3.19059492
H	-5.99547329	1.13202840	-1.87435662
C	-4.86090496	4.70114725	-2.87867634

H	-3.37409643	4.48946469	-1.32991095
C	-5.92179394	4.09049137	-3.55377791
H	-7.14722023	2.32260879	-3.71753909
H	-4.54003817	5.70096897	-3.15787762
H	-6.42618908	4.61354972	-4.36154334
N	-4.45225270	1.56105482	0.71224414
C	-5.87364057	1.64908003	0.98060159
H	-6.01612439	1.86481904	2.04570750
H	-6.41781158	2.40136022	0.39368164
H	-6.32952817	0.66778629	0.79759106

XYZ coordinates obtained for the reaction of 1’ with $W(CO)_6$ at the B3LYP-D3/6-31G* W (LanL2DZ) level of theory.

1'

57

Si	1.65615400	-0.46126400	0.15919200
N	0.46116800	0.39864000	-1.00773500
C	-1.14821900	2.11708300	-0.13989000
P	1.27743900	-2.51965200	0.59126700
Si	3.80011100	0.41598300	-0.24165800
N	0.68857400	0.89501700	1.06526600
C	-1.02857900	3.44672700	0.28756500
H	-0.08078700	3.80615400	0.67694300
C	-2.12170700	4.30729500	0.19219700
H	-2.02475800	5.34008300	0.51400200
C	-3.33822700	3.84052800	-0.31286900
H	-4.18997100	4.51152400	-0.38007800
C	-3.46160100	2.51181500	-0.72865000
H	-4.40898500	2.14581100	-1.11402000
C	-2.36968900	1.64922400	-0.64959700
H	-2.46454100	0.60946400	-0.94677200
C	-0.01656700	1.17751200	-0.03166900

C	0.03213400	0.35431200	-2.38927100
C	4.33055600	0.00695400	-2.01698100
H	5.32608200	0.41791300	-2.22845800
H	3.63097200	0.42910100	-2.74829200
H	4.36940300	-1.07640300	-2.17643000
C	0.56620100	1.49280600	2.37535300
C	-0.58954300	-2.32008900	0.37657000
C	-1.19315900	-2.55817000	-0.88457800
C	-2.55961700	-2.30005900	-1.06651800
H	-3.00210400	-2.47679500	-2.04589800
C	-3.37000700	-1.82986400	-0.03091700
C	-2.78360700	-1.66984100	1.22680100
H	-3.40356500	-1.34268200	2.06032100
C	-1.42474800	-1.91981100	1.45210500
C	5.05767400	-0.29523700	0.98393800
H	6.06119400	0.10177700	0.78444500
H	5.10447000	-1.38751500	0.90832000
H	4.79344100	-0.04370200	2.01740500
C	3.67891500	2.30112200	-0.03726600
H	4.62918700	2.78849700	-0.28885400
H	3.42354700	2.57021700	0.99435900
H	2.90385800	2.71622500	-0.69273200
C	-0.40598300	-3.10444200	-2.05571000
C	-4.82528400	-1.49191800	-0.25623900
C	-0.89679300	-1.78908900	2.86286400
H	1.00253400	0.81281200	3.11101900
H	1.10380700	2.44782600	2.43813000
H	-0.48353800	1.66474500	2.63733900
H	-0.80815400	-0.33509600	-2.52607100
H	-0.26849200	1.34897000	-2.73505300
H	0.86610900	0.00689600	-3.00460600
H	-1.67895900	-1.43861300	3.54531800
H	-0.52945800	-2.75490900	3.23056700
H	-0.04997600	-1.10290800	2.92036100

H	-1.05102400	-3.24112400	-2.93107200
H	0.42392400	-2.44807000	-2.33473000
H	0.04631400	-4.07169200	-1.80807600
H	-5.45849000	-1.87995100	0.55030900
H	-4.97913100	-0.40403000	-0.28587500
H	-5.19329200	-1.90255400	-1.20246700

W(CO)6

13

C	-0.81154700	-1.80693200	0.59750500
C	-1.41182100	0.13532700	-1.50599700
C	-1.27703100	0.99812800	1.28581700
C	0.81066600	1.80727900	-0.59674800
O	-1.98813200	1.55365700	2.00059300
O	1.26236600	2.81259300	-0.92913800
O	-1.26385900	-2.81182800	0.93032100
O	-2.19703800	0.21061600	-2.34442600
W	-0.00010300	-0.00005900	0.00008100
C	1.41299600	-0.13546800	1.50503000
O	2.19950200	-0.21085300	2.34224600
C	1.27678900	-0.99844600	-1.28549100
O	1.98807700	-1.55355700	-2.00043700

W-int1

68

P	-0.53170400	-0.32342300	0.95038300
Si	0.72347000	1.40279000	0.57323700
Si	-0.01635500	3.59395600	1.00915600
N	2.42732900	1.01850000	1.16967500
C	0.68888200	-1.71638200	0.68016400
C	1.03610200	-2.47779200	1.83237500
C	2.02512700	-3.46179200	1.74427800
H	2.28702800	-4.02608400	2.63743300
C	2.68811100	-3.74292100	0.54443300
C	2.30785200	-3.02348300	-0.58595700
H	2.79366700	-3.23483600	-1.53749700

C	1.31738000	-2.02906800	-0.54905700
C	0.36631900	-2.26068300	3.17438200
C	3.74751900	-4.81737200	0.47254500
C	0.98889100	-1.37499000	-1.87378600
C	-1.16534800	4.12506100	-0.39974200
H	-1.40726100	5.19100400	-0.30203000
H	-2.10430700	3.56424400	-0.37640100
H	-0.70655800	3.96960100	-1.38235300
C	1.55368900	4.66103000	0.99965700
H	1.30305800	5.71410600	1.17850200
H	2.06616000	4.59719400	0.03231700
H	2.26009400	4.34469300	1.77559200
C	-0.89860000	3.69510000	2.67714600
H	-1.18860400	4.73228800	2.88843700
H	-0.25780000	3.34941600	3.49618200
H	-1.80785900	3.08543300	2.67190000
N	1.92630000	1.52770900	-0.85349300
C	1.95917500	1.87930700	-2.25830100
C	3.05655700	0.47186300	2.35569200
C	2.93419000	1.12936700	-0.06788300
C	4.30781500	0.80553300	-0.49040100
C	4.83866700	-0.45738900	-0.18108300
H	4.23469000	-1.18242500	0.35479300
C	6.12261500	-0.78993800	-0.61055500
H	6.52731200	-1.77163600	-0.38264500
C	6.88254200	0.13382600	-1.33366700
H	7.88447100	-0.12698400	-1.66227300
C	6.35500400	1.39167200	-1.63964400
H	6.94551300	2.10950500	-2.20099700
C	5.06585900	1.72717800	-1.22759300
H	4.65253400	2.70378400	-1.46064000
H	0.27510200	-0.56096400	-1.77503000
H	1.89569300	-0.98381300	-2.34806200
H	0.55695900	-2.11384500	-2.55803500

H	4.33179400	-4.86727400	1.39832600
H	3.29657500	-5.80708600	0.32132500
H	4.43855300	-4.64406600	-0.35971600
H	0.58109100	-1.26693200	3.58292900
H	-0.72429500	-2.33479400	3.09748200
H	0.70596700	-3.00861700	3.89879000
H	2.62819500	0.95514500	3.23729700
H	2.88439900	-0.60774900	2.43068200
H	4.13380300	0.66184900	2.33865200
H	2.17298900	2.94675800	-2.39608500
H	2.72192700	1.30007800	-2.78770100
H	0.98286800	1.66421700	-2.69644700
C	-2.00516300	0.77050800	-1.84187800
C	-3.41618900	1.26498200	0.63303900
C	-4.57969200	-0.51282700	-1.32220000
C	-2.17591100	-2.10848300	-1.33644800
O	-5.60296200	-0.57586800	-1.87591400
O	-1.81985000	-3.07767500	-1.85839200
O	-1.49440500	1.44214300	-2.64206100
O	-3.75894200	2.20697200	1.21856500
C	-3.45901800	-1.57047600	1.18364700
O	-3.79097200	-2.22256600	2.07943000
W	-2.81460000	-0.40180800	-0.38628700

CO

2

C	0.00000000	0.00000000	-0.65022600
O	0.00000000	0.00000000	0.48766900

W-ts1'

70

P	0.57550700	0.21167500	-0.38285700
Si	-1.61677900	1.47556100	0.21136300
Si	-1.71108900	3.57342900	1.29979200
N	-2.72942900	1.19539700	-1.23432400
C	-0.24621900	-1.13033600	0.71448400

C	-0.89227500	-2.21328000	0.07234100
C	-1.39071600	-3.26592600	0.85016900
H	-1.88255900	-4.10093400	0.35297000
C	-1.25641600	-3.28806800	2.24333900
C	-0.63131200	-2.20045900	2.85893400
H	-0.52737300	-2.19323000	3.94246900
C	-0.13301900	-1.11691500	2.12373900
C	0.06189200	4.18484900	1.53099700
H	0.07668300	5.08089000	2.16393400
H	0.51119200	4.43790500	0.56554700
H	0.69747100	3.42673800	2.00241300
C	-2.52705600	3.25731100	2.98433900
H	-2.55674500	4.18650900	3.56734500
H	-1.97272700	2.51675400	3.57321000
H	-3.55721400	2.90041200	2.87093700
C	-2.74225000	4.79519300	0.28755100
H	-2.82427200	5.75821600	0.80756400
H	-3.75593900	4.41554300	0.11528000
H	-2.27407300	4.96953000	-0.68708400
N	-3.00207000	0.37594600	0.73418000
C	-3.53398200	-0.25242700	1.92821700
C	-2.60454500	1.26972600	-2.68088200
C	-3.48887200	0.34634500	-0.51628300
H	-2.81412900	2.28541200	-3.02751100
H	-1.58467100	1.00626000	-2.97766400
H	-3.29845500	0.57508900	-3.15983100
H	-4.61472100	-0.39607400	1.83725200
H	-3.05434200	-1.21862400	2.10518700
H	-3.33635600	0.39374100	2.78724700
C	3.25943700	1.02893500	-2.04839200
C	2.74540300	-1.79730900	-1.70845200
C	5.14142600	-0.54967300	-0.51782300
C	3.03297200	-1.53502000	1.15003700
O	6.29220100	-0.72140300	-0.56512700

O	2.96595500	-2.26384100	2.04699800
O	3.30075300	1.74306100	-2.95791900
O	2.47018800	-2.66726100	-2.42343200
C	0.29158100	1.81955000	-1.02136800
O	-0.25006700	2.79739100	-1.47599200
W	3.16140100	-0.24408100	-0.43795600
C	3.32070800	1.35293300	0.83861300
O	3.33891500	2.25611900	1.56423500
C	-4.60217700	-0.47968100	-1.00158800
C	-4.67186500	-1.83268500	-0.62867200
C	-5.58672400	0.06963100	-1.83879200
C	-5.71645800	-2.62658800	-1.09792500
H	-3.89382900	-2.25906700	-0.00295600
C	-6.63434600	-0.72880300	-2.29403400
H	-5.53676900	1.11907600	-2.11258300
C	-6.69879900	-2.07625800	-1.92656400
H	-5.76210500	-3.67526000	-0.81961600
H	-7.39972500	-0.30045200	-2.93414000
H	-7.51383500	-2.69713900	-2.28700500
C	-1.75127200	-4.46351500	3.05221800
C	0.48865100	0.04105300	2.87439500
C	-1.05867200	-2.27826400	-1.43247100
H	-0.14497300	0.93741400	2.83178800
H	0.63078800	-0.20890400	3.93006500
H	1.45643700	0.32921700	2.45820200
H	-2.62554900	-4.93223200	2.58695500
H	-0.97494700	-5.23553600	3.13562400
H	-2.02365400	-4.16547400	4.07038400
H	-1.68537800	-3.12988100	-1.71658100
H	-1.52409700	-1.37041200	-1.83039400
H	-0.09389700	-2.38835000	-1.93943600

W-int2'

70

P	-0.65577100	-0.37606000	0.32622200

Si	1.98864200	1.55226300	-0.16244800
Si	2.22690200	3.69103700	-1.10832300
N	2.91194100	1.20404000	1.37692000
C	0.08663800	-1.28361500	-1.10398900
C	0.71262900	-2.53019700	-0.84787000
C	1.21870800	-3.28211500	-1.91272600
H	1.69180300	-4.23983900	-1.70403300
C	1.13451600	-2.84032200	-3.23616200
C	0.51491700	-1.61225100	-3.47701400
H	0.42577000	-1.25811600	-4.50209500
C	-0.01885500	-0.82880900	-2.44309200
C	0.85634500	-3.07986900	0.55638200
C	1.71903400	-3.65706500	-4.36429500
C	-0.72522600	0.45493100	-2.82005200
C	0.64504500	4.06028300	-2.07301000
H	0.61886500	5.11715200	-2.36613800
H	-0.24103400	3.85207900	-1.46340800
H	0.57922700	3.45771800	-2.98573000
C	3.74687900	3.66861300	-2.23792000
H	3.90304800	4.65550200	-2.69086200
H	3.62850300	2.94246200	-3.05064300
H	4.65451800	3.40779400	-1.68105700
C	2.45537800	4.90964700	0.32062700
H	2.53758900	5.93485100	-0.06190700
H	3.36124300	4.69147500	0.89822900
H	1.59587600	4.86409400	0.99777100
N	3.24325700	0.25205200	-0.51896800
C	3.59902600	-0.67699100	-1.57658200
C	2.88075600	1.61115600	2.76797600
C	3.60139400	0.22061600	0.77873200
C	4.50602000	-0.74780500	1.41369000
C	4.11667300	-1.38595100	2.60322300
H	3.15317000	-1.16029000	3.04876500
C	4.95451500	-2.33455100	3.18704400

H	4.64606900	-2.83597400	4.09920500
C	6.18453800	-2.63961600	2.59771400
H	6.83769300	-3.37520000	3.05796700
C	6.57534400	-2.00389000	1.41547500
H	7.53175100	-2.24033700	0.95912200
C	5.73622500	-1.06623600	0.81683500
H	6.03806300	-0.56824900	-0.09906300
H	-0.36162100	1.32174300	-2.26361100
H	-0.60663300	0.66397700	-3.88790900
H	-1.79659200	0.38680400	-2.61015400
H	1.56295000	-4.72947300	-4.20315000
H	1.27322800	-3.38881600	-5.32764700
H	2.80270400	-3.49735000	-4.44765300
H	1.47138500	-2.42247600	1.18243700
H	-0.11284000	-3.17666900	1.05892800
H	1.32707000	-4.06833700	0.53934800
H	2.80938100	2.70038600	2.81796500
H	2.00961600	1.18588200	3.27888100
H	3.79117200	1.29066500	3.28134200
H	4.50378200	-0.34922200	-2.10173400
H	3.76139900	-1.68075500	-1.17552700
H	2.77371800	-0.72653700	-2.28839500
C	-3.01483100	1.62718200	-0.46543900
C	-2.92154900	0.83769900	2.30193900
C	-5.22006700	0.06114100	0.69530800
C	-3.24096800	-1.95081000	1.52080200
O	-6.37419400	0.19459600	0.78317300
O	-3.20795100	-2.96612700	2.07890400
O	-2.83769200	2.62044200	-1.03367900
O	-2.74704800	1.38584300	3.30551300
C	0.20841400	1.15836600	0.32191500
O	-0.07067300	2.22043400	0.93920300
W	-3.23561300	-0.15692100	0.52690300
C	-3.44592500	-1.16603100	-1.24201100

O	-3.53699400	-1.73540800	-2.24727000

W-int2

123

C	-5.45680700	-1.68861600	-1.42567600
N	-4.93357300	-0.93000900	-0.30384300
C	5.35650000	-0.57090000	-2.19352100
Si	-3.45286300	-0.96306600	0.83845800
C	-5.60315700	-0.13903000	0.53981800
N	4.93516800	-0.41240300	-0.81377300
Si	3.66471900	-1.12109200	0.36140800
N	-4.81613200	0.04252100	1.61084400
C	5.63822100	0.09245800	0.20294700
C	-4.89973900	1.12904900	2.57230700
N	5.01653800	-0.27980500	1.33109900
C	5.16589400	0.34157500	2.63588900
C	-6.93942500	0.44595100	0.32720400
C	-7.96522200	0.22292200	1.25672400
C	-7.17152900	1.24448100	-0.80226900
C	-9.22347900	0.78676700	1.04734700
H	-7.77563100	-0.39954300	2.12651500
C	-8.42923700	1.81322100	-0.99929900
H	-6.35900700	1.44129100	-1.49424100
C	-9.45567800	1.58150500	-0.07906500
H	-10.02103700	0.60697600	1.76224200
H	-8.60657800	2.43987800	-1.86839900
H	-10.43546200	2.02294000	-0.23765300
C	6.84788200	0.93085500	0.10772400
C	8.04546500	0.51946100	0.70895500
C	6.78302800	2.15548800	-0.57270300
C	9.17896700	1.32687600	0.61616300
H	8.08574200	-0.43261000	1.23047500
C	7.91781900	2.96198100	-0.65223500
H	5.84007500	2.48082700	-1.00066600
C	9.11569200	2.54697600	-0.06327100

H	10.10972400	1.00472500	1.07381100
H	7.86577600	3.91493200	-1.17062600
H	9.99904500	3.17558900	-0.13070500
H	-4.42877600	0.81183300	3.50592600
H	-4.38110000	2.01889600	2.19825700
H	-5.94336600	1.38475300	2.77464300
H	-4.62569900	-1.94622800	-2.08570600
H	-5.93771600	-2.61551500	-1.08756400
H	-6.18805800	-1.10108100	-1.98734300
H	4.47190000	-0.77551500	-2.79893100
H	5.84285900	0.33712300	-2.56098500
H	6.05520400	-1.41069900	-2.30088700
H	6.22156800	0.51634600	2.86246000
H	4.62840200	1.29542600	2.68020300
H	4.75258600	-0.33013000	3.39157000
P	-1.68017600	0.20732200	0.44744800
P	1.73583300	-0.18370700	0.63809200
C	1.44090000	-1.68023600	-2.39662100
C	0.09508000	-2.75744200	-0.10654000
C	-0.11335900	0.68076900	-2.27235800
O	0.17435000	-3.75402200	0.50078600
O	2.32220500	-1.98196900	-3.11946300
O	-0.21659800	1.65110300	-2.90311000
C	2.29595700	1.60320800	0.72061300
C	2.21948500	2.23742000	1.99349500
C	2.80523500	2.34846900	-0.37203600
C	2.66786400	3.55403200	2.14603700
C	3.25206300	3.66303500	-0.16568000
C	3.20306400	4.28409200	1.08055100
H	2.59912200	4.02289700	3.12620100
H	3.64193100	4.21785000	-1.01876700
C	-2.50568900	1.79873500	-0.12950700
C	-2.37162300	2.93141700	0.72401100
C	-3.27671000	1.94350800	-1.31042300

C	-3.04882800	4.11808600	0.42624400
C	-3.94419800	3.15326900	-1.56139200
C	-3.85783400	4.24840900	-0.70643600
H	-2.94190400	4.96683300	1.09971000
H	-4.54194700	3.23594900	-2.46870200
C	1.67073500	1.52984700	3.21709200
C	3.72951700	5.68579600	1.28078900
C	2.88608500	1.83363700	-1.79385500
C	-1.49006000	2.91432000	1.95474800
C	-3.40114400	0.88482800	-2.38264900
C	-4.57370100	5.54399200	-1.00759800
H	-1.53647600	3.87645600	2.47687800
H	-1.77407200	2.12466500	2.65762800
H	-0.44485900	2.73273900	1.68327000
H	-5.40719100	5.39025700	-1.70159800
H	-4.97090200	6.00404300	-0.09524000
H	-3.89461200	6.27442300	-1.46748500
H	-4.43665100	0.78852700	-2.72919700
H	-2.79327800	1.16319900	-3.25144500
H	-3.05915500	-0.08873400	-2.05118400
H	1.59203400	2.22668000	4.05925300
H	0.68176800	1.10124600	3.02879300
H	2.31276100	0.69624800	3.52362400
H	3.16713800	6.21903200	2.05541600
H	4.78201700	5.67208100	1.59606100
H	3.67422600	6.27149800	0.35665800
H	3.88962900	1.98511200	-2.20867500
H	2.64089100	0.77709800	-1.87309600
H	2.18710100	2.38318400	-2.43314300
Si	-3.38790400	-3.12538400	1.76164000
Si	4.01740200	-3.44805600	0.47218400
C	5.90965200	-3.63868400	0.48911200
C	3.30279800	-4.35832900	-1.02168100
C	3.27140200	-4.10774800	2.08235300

C	-2.00886100	-3.27608100	3.04590500
C	-5.08336300	-3.36715100	2.58760000
C	-3.20316100	-4.37869900	0.35328900
H	3.63256700	-5.40528600	-1.00828700
H	2.20973200	-4.34334900	-1.00194700
H	3.62139800	-3.90429900	-1.96480000
H	6.35028100	-3.27142400	-0.44572800
H	6.36028900	-3.07761000	1.31590300
H	6.19122700	-4.69343600	0.59949600
H	3.48379100	-5.17915000	2.19114700
H	3.67851900	-3.59080700	2.95913100
H	2.18463800	-3.97512400	2.07832700
H	-2.14662800	-2.55199900	3.85719300
H	-2.00750800	-4.28222000	3.48461100
H	-1.02811700	-3.10340000	2.59420600
H	-5.23133800	-2.65813300	3.41002200
H	-5.89789500	-3.21928500	1.86791200
H	-5.17482200	-4.38285600	2.99280100
H	-2.29049600	-4.20897600	-0.22367600
H	-3.16629900	-5.39914300	0.75556700
H	-4.05403700	-4.31843100	-0.33531900
W	0.00597400	-1.05804700	-1.20181700
C	-1.41011400	-1.88573800	-2.29370400
O	-2.27015300	-2.40982700	-2.90507500

W-int3

127

C	-6.10748800	0.56742100	-0.35425500
N	-5.01063900	0.22745600	0.53297700
O	-0.54814300	-2.52474000	2.97851600
C	5.58498800	-0.88546300	-1.51691500
Si	-3.55477300	-0.92615700	0.66688400
C	-4.74025700	0.75134900	1.73071500
N	4.86130200	-0.37071000	-0.36833200
Si	3.39599800	-0.84707600	0.69637400

N	-3.78430600	-0.01076700	2.29188800
C	-1.28372300	-2.07906300	2.23597100
C	5.31259700	0.45843100	0.57568800
C	-2.85951100	0.41175400	3.33106100
N	4.47644800	0.35582200	1.62000000
C	4.29936800	1.34084700	2.67398600
C	-5.31217400	1.99627700	2.27231000
C	-5.79184300	2.06362100	3.58832200
C	-5.33557700	3.13720600	1.45277100
C	-6.30406300	3.26463000	4.07782700
H	-5.77386600	1.17551500	4.21311400
C	-5.83833400	4.33683100	1.95388600
H	-4.93921100	3.08176100	0.44234800
C	-6.32578000	4.40036100	3.26268700
H	-6.68498100	3.31488000	5.09355800
H	-5.84737000	5.22147100	1.32407300
H	-6.72100000	5.33573000	3.64861000
C	6.48875800	1.34284300	0.48185000
C	7.52590600	1.24400500	1.41989200
C	6.55227800	2.29754400	-0.54356800
C	8.63011900	2.09038000	1.32209400
H	7.46777000	0.49842900	2.20774300
C	7.65491300	3.14684400	-0.62893800
H	5.72538800	2.38936400	-1.24070300
C	8.69494200	3.04103300	0.29914300
H	9.43838000	2.00847800	2.04288900
H	7.70041300	3.89260800	-1.41714800
H	9.55457500	3.70125100	0.22727200
H	-1.86987900	0.59145400	2.89610700
H	-3.20239800	1.33254500	3.80956000
H	-2.77041900	-0.36745800	4.09417800
H	-6.57615200	-0.35009700	-0.72068000
H	-6.86033100	1.15434500	0.17881600
H	-5.74581300	1.13944200	-1.21329200

H	4.85949700	-1.25547300	-2.24294900
H	6.19116200	-0.10155500	-1.97916300
H	6.24472200	-1.71431200	-1.22897300
H	5.26169700	1.77465400	2.95958000
H	3.63061100	2.14473700	2.34569000
H	3.86013700	0.84800000	3.54310300
P	-1.85678900	-0.01198100	-0.39326600
P	1.41111200	-0.06159800	0.32712900
C	1.77093400	-2.13376900	-2.30046500
C	0.12771700	-3.06556100	-0.24660700
C	0.00629900	0.03733600	-2.87542500
O	0.17886500	-4.03339100	0.41291500
O	2.79518000	-2.46958800	-2.77815300
O	-0.06236700	0.87242800	-3.67866200
C	1.87374800	1.71805000	-0.06775600
C	1.46501100	2.71362100	0.86411700
C	2.60718700	2.13555000	-1.20811100
C	1.84237400	4.04795000	0.67997300
C	2.97024900	3.48491100	-1.34364100
C	2.61739600	4.45566100	-0.40910300
H	1.51761500	4.79097900	1.40647300
H	3.53864100	3.78100600	-2.22495900
C	-2.82308200	1.04029400	-1.61285400
C	-2.68410500	2.45068400	-1.52107100
C	-3.64492600	0.51538000	-2.64103000
C	-3.35568700	3.27954700	-2.42858900
C	-4.28379900	1.38123800	-3.53990400
C	-4.15457300	2.76763700	-3.45416000
H	-3.23723500	4.35837100	-2.34018000
H	-4.90724600	0.95479600	-4.32416500
C	0.60100800	2.38464600	2.06263600
C	3.06357300	5.89073200	-0.55988500
C	3.00707500	1.23035000	-2.35366000
C	-1.81348500	3.10728500	-0.47272500

C	-3.92646800	-0.96142100	-2.77401400
C	-4.82540300	3.68182000	-4.45225800
H	-1.85264000	4.19868500	-0.56241900
H	-2.12012300	2.83383500	0.54266400
H	-0.76775000	2.80164800	-0.57409800
H	-5.73059700	3.22723800	-4.87003500
H	-5.10323000	4.63943400	-3.99709800
H	-4.15615500	3.90571900	-5.29384400
H	-4.29697300	-1.20806400	-3.77439000
H	-3.04106600	-1.56079900	-2.57249800
H	-4.70134000	-1.28119100	-2.06191500
H	0.20468700	3.30123700	2.51417900
H	-0.23866200	1.74280700	1.77789600
H	1.16459000	1.84865600	2.83248100
H	2.32914700	6.58629900	-0.13858300
H	4.01377700	6.06643700	-0.03681300
H	3.21783200	6.15504000	-1.61181000
H	4.06746600	1.35440300	-2.60201300
H	2.82592600	0.18040700	-2.14710500
H	2.43310200	1.49153300	-3.24964800
Si	-4.33824900	-3.15554200	0.78443100
Si	3.85481900	-3.05301400	1.38865900
C	5.66598100	-2.99035500	1.96720400
C	3.69084000	-4.23489400	-0.07922300
C	2.73701700	-3.60539000	2.80887100
C	-4.37622000	-3.72409400	2.59589000
C	-6.11426100	-3.14849900	0.10782500
C	-3.25676300	-4.30149600	-0.25607900
H	4.10089900	-5.21746700	0.18812100
H	2.64011900	-4.36612600	-0.35251300
H	4.21737400	-3.86956100	-0.96651400
H	6.33227900	-2.66816400	1.15780100
H	5.79239100	-2.29219800	2.80265600
H	5.99803400	-3.98225500	2.29873600

H	3.05077400	-4.59356200	3.16926500
H	2.77545500	-2.90514200	3.64982100
H	1.69914400	-3.68133100	2.47240700
H	-4.96684600	-3.03820300	3.21412600
H	-4.82552200	-4.72253800	2.67127800
H	-3.36902200	-3.78075500	3.02310500
H	-6.76045100	-2.46738700	0.67343800
H	-6.14287600	-2.85082500	-0.94666100
H	-6.54410100	-4.15592200	0.17686900
H	-2.26526000	-4.42586100	0.18830300
H	-3.72424500	-5.29231200	-0.32594000
H	-3.11267800	-3.91921500	-1.27196900
C	1.54571500	-0.46799400	4.11638300
O	0.49778000	-0.20934100	4.47910100
W	0.09249100	-1.48930500	-1.50672300
C	-0.94982400	-2.64238300	-2.72308400
O	-1.52856600	-3.37947900	-3.43370700

W-ts1

127

N	4.58984100	0.85140300	-0.52052900
O	1.52352400	-2.40245800	-2.79390700
Si	3.19224400	-0.51178300	-0.90336100
C	4.33155200	1.43237200	-1.67717900
N	-4.85896300	-0.65333400	-0.24592600
Si	-3.24715500	-0.83180200	-1.12780900
N	3.34211400	0.73783100	-2.27616400
C	1.67195200	-1.49499300	-2.08469900
C	-5.14982300	0.45398500	-0.94700000
N	-4.16584400	0.59156400	-1.85038400
P	1.68451200	-0.02601600	0.64469600
P	-1.40693300	-0.07628800	-0.17630800
C	-1.91030900	-2.62172100	1.87757100
C	0.03794500	-3.14210500	-0.06188500
C	-0.36074600	-0.46871600	2.97890400

O	0.16504800	-4.02995000	-0.81231000
O	-2.96100700	-3.09297000	2.13340800
O	-0.42112000	0.22812200	3.90553200
C	-2.00071400	1.60103000	0.42188900
C	-1.55074300	2.77664600	-0.23884800
C	-2.90306100	1.73963300	1.50827800
C	-2.05050600	4.02526600	0.14997500
C	-3.37482000	3.01363700	1.85531000
C	-2.97549400	4.16887100	1.18691000
H	-1.69968800	4.91331300	-0.37212500
H	-4.08320900	3.09515200	2.67823200
Si	4.64172900	-2.34386300	-1.36197900
Si	-3.31647700	-2.83494600	-2.36933300
W	-0.21474500	-1.74962100	1.38652100
C	0.83801800	-2.96381400	2.55777800
O	1.42352000	-3.69955400	3.25515500
C	-0.12373900	-0.28529100	-2.23395700
O	-0.54032400	0.14197700	-3.23989400
C	2.62025100	0.55325200	2.14535000
C	2.31319300	1.84812400	2.64235100
C	3.54255700	-0.24314500	2.86578700
C	2.94286400	2.31425500	3.80198400
C	4.14565000	0.26196600	4.02459800
C	3.86408100	1.53887900	4.51112300
H	2.70083100	3.31144300	4.16631600
H	4.85683900	-0.36461800	4.56092100
C	3.94904500	-1.62145700	2.40565000
C	1.30695700	2.75284600	1.96384700
C	4.50102400	2.05258800	5.78129700
C	-3.39739300	0.60024100	2.37240300
C	-3.50310600	5.52482500	1.59222000
C	-0.52675400	2.75112200	-1.35107400
C	3.89642500	-3.93312300	-0.65017500
C	6.38246800	-2.03606400	-0.66274600

C	4.84075400	-2.50921900	-3.24734800
C	-3.37726600	-4.26143000	-1.12962700
C	-1.84645600	-3.00233000	-3.54107600
C	-4.94532900	-2.73035900	-3.33932700
C	-3.90587100	1.70181300	-2.74606900
C	-5.64618400	-1.32497200	0.77080100
C	5.39779100	1.38970000	0.55806200
C	2.96786200	0.79412400	-3.67886700
C	4.97164100	2.64461500	-2.23481400
C	4.17948600	3.74686700	-2.58805800
C	6.36158200	2.69856000	-2.41062300
C	4.77674100	4.89653800	-3.10471800
H	3.10386500	3.69912500	-2.44334000
C	6.95311400	3.84767600	-2.93531500
H	6.96921100	1.83728500	-2.14810300
C	6.16249300	4.94755900	-3.27987600
H	4.16150300	5.75148100	-3.36996100
H	8.02948800	3.88404500	-3.07590000
H	6.62569700	5.84272200	-3.68512800
C	-6.27263500	1.37664800	-0.71486200
C	-6.00381400	2.74249400	-0.52745700
C	-7.58990100	0.90053900	-0.63579800
C	-7.05211600	3.62189900	-0.26219300
H	-4.98090400	3.10236300	-0.54630800
C	-8.63400600	1.79026000	-0.38599900
H	-7.79465400	-0.15507800	-0.78516000
C	-8.36588600	3.14906700	-0.19675200
H	-6.84094800	4.67518200	-0.10241000
H	-9.65455500	1.42280600	-0.33553400
H	-9.18074400	3.83818800	0.00570400
H	7.05398800	-2.84986400	-0.96598400
H	6.79093300	-1.09653800	-1.05292300
H	6.40041600	-1.98133900	0.43029400
H	5.51594500	-3.34191300	-3.48355400

H	3.88324500	-2.70314300	-3.74072100
H	5.27207100	-1.59829200	-3.68028300
H	3.83542000	-3.88869700	0.44252400
H	2.88305700	-4.10000500	-1.03099400
H	4.50885400	-4.80256200	-0.92143200
H	-2.42354900	-4.36508600	-0.60532500
H	-4.15775400	-4.11907700	-0.37464000
H	-3.58004700	-5.20139700	-1.65874900
H	-1.98692000	-3.88967500	-4.17161800
H	-1.73899700	-2.13178200	-4.19616200
H	-0.91122600	-3.13132400	-2.98861800
H	-5.80115200	-2.60008900	-2.66638900
H	-4.94035900	-1.89125400	-4.04427000
H	-5.10588400	-3.65254800	-3.91185400
H	1.56641000	2.93635500	0.91592400
H	0.30538800	2.30980400	1.96061500
H	1.24273800	3.71771400	2.47926500
H	4.64830700	3.13820600	5.74632700
H	3.86967200	1.83967700	6.65461000
H	5.47413000	1.58254000	5.96220400
H	4.77064700	-1.56231100	1.68231900
H	4.29812900	-2.23350500	3.24375500
H	3.12377200	-2.14050200	1.91751700
H	6.12291600	2.11992300	0.18664500
H	4.76636900	1.86553500	1.31442800
H	5.94030900	0.57249800	1.04038600
H	3.43925800	1.65100000	-4.16701300
H	3.28141400	-0.11556300	-4.20512200
H	1.88330600	0.89189200	-3.77901400
H	-4.97082200	-1.88447100	1.42063500
H	-6.20414500	-0.59998700	1.37061600
H	-6.35609500	-2.02773000	0.31751700
H	-3.28481500	1.34245800	-3.56991100
H	-4.84340700	2.09678500	-3.14894300

H	-3.36927400	2.50798800	-2.23432700
H	-2.93494400	0.65930500	3.36363200
H	-4.48125500	0.66922500	2.51387400
H	-3.16114500	-0.38042700	1.97015300
H	-3.36139400	6.26566600	0.79817900
H	-4.57205600	5.48005600	1.83192800
H	-2.98798200	5.89855100	2.48677000
H	-0.93429900	2.32931800	-2.27469600
H	-0.18186600	3.76679000	-1.57336900
H	0.34281400	2.14585200	-1.07235300

W-int4

127

C	-2.50083100	2.54933600	2.27004700
N	-3.02253500	1.25341700	1.83674600
O	1.94330100	-0.78529400	2.20144600
C	5.74453100	0.07755800	-1.14020900
Si	-2.10375100	-0.27540500	1.60476800
C	-4.36813800	1.07624300	1.57648600
N	4.79724200	0.22292200	-0.04977800
Si	3.38529600	-0.70361700	1.04890000
N	-4.73440000	-0.11678100	1.25504900
C	0.95603400	-0.24933000	1.47676300
C	4.83242100	1.18003800	0.85932900
C	-6.11675800	-0.43701600	0.96250100
N	3.95603300	0.84262600	1.83001500
C	3.50736000	1.65639000	2.94883400
C	-5.27786800	2.26346800	1.64602000
C	-5.55730300	2.97745800	0.47351000
C	-5.87890500	2.64332100	2.85247600
C	-6.42526100	4.07010600	0.51293400
H	-5.10091900	2.66788000	-0.46287800
C	-6.74842300	3.73534200	2.88734200
H	-5.66480400	2.08145700	3.75799700
C	-7.02055300	4.45151200	1.71849200

H	-6.63926000	4.62110100	-0.39910100
H	-7.21358000	4.02544800	3.82555500
H	-7.69725100	5.30127500	1.74693000
C	5.63845200	2.41459300	0.82933200
C	6.38771900	2.81235700	1.94630500
C	5.62227400	3.21230800	-0.32540400
C	7.12952400	3.99221700	1.90032400
H	6.39875700	2.19252800	2.83760000
C	6.35272700	4.39892100	-0.35839100
H	5.01315000	2.91727400	-1.17316800
C	7.11035200	4.78708200	0.75049900
H	7.71836900	4.29306400	2.76179300
H	6.32938300	5.02039600	-1.24904400
H	7.68306100	5.70967800	0.72047500
H	-6.34950800	-1.42333300	1.38084500
H	-6.83936900	0.28612800	1.36457500
H	-6.26817200	-0.49728800	-0.12293300
H	-2.45770400	3.27256500	1.44486900
H	-3.12498600	2.97852200	3.05966000
H	-1.50067900	2.40995500	2.67675500
H	5.71224000	-0.95222000	-1.49872600
H	5.49665000	0.73479200	-1.98009000
H	6.76706000	0.30327200	-0.81531800
H	2.51266300	1.31848700	3.24822900
H	4.17654200	1.55247100	3.81096400
H	3.46367200	2.71332500	2.66683300
P	-1.87586800	-0.71155500	-0.56415800
P	1.51258500	-0.31144000	-0.25651500
C	1.71057200	-2.95689700	-1.96680700
C	-1.02278400	-3.40389300	-2.49906700
C	-0.12546400	-3.18597800	0.22867100
O	-1.61583400	-4.19022700	-3.11903700
O	2.73836400	-3.45556700	-2.21066300
O	-0.26702700	-3.86361400	1.15870100

C	1.84514600	1.31068800	-1.09408300
C	1.50023000	2.58080900	-0.56967400
C	2.59436700	1.23569700	-2.29796100
C	1.90012300	3.73139600	-1.25826100
C	2.96828700	2.42149600	-2.94317400
C	2.63659300	3.68038000	-2.44266300
H	1.62814200	4.70114700	-0.84730000
H	3.54148500	2.35087300	-3.86526700
C	-3.19247200	0.02891200	-1.60598500
C	-3.09185100	1.35987100	-2.06900500
C	-4.29915700	-0.76608500	-1.98900600
C	-4.11053200	1.87827900	-2.87878200
C	-5.29310000	-0.20343100	-2.79592600
C	-5.22508900	1.11950600	-3.24348900
H	-4.02752900	2.90420800	-3.23290700
H	-6.14563000	-0.81675500	-3.08171800
C	0.70680900	2.77170500	0.69857300
C	3.09396800	4.94638100	-3.12580600
C	3.03149300	-0.06271600	-2.94186800
C	-1.92777200	2.24873900	-1.70120400
C	-4.43467400	-2.20158700	-1.53993600
C	-6.33769000	1.71628700	-4.07238400
H	-1.96050200	3.19163600	-2.25659500
H	-1.94837700	2.48955100	-0.63351200
H	-0.96405900	1.77168700	-1.90567700
H	-6.78852900	0.97029300	-4.73606300
H	-7.13942100	2.10536400	-3.43000300
H	-5.97906800	2.54830900	-4.68789200
H	-3.64761300	-2.83081500	-1.96903100
H	-4.35186700	-2.27816800	-0.45133600
H	-5.40140000	-2.61700100	-1.84278100
H	-0.24740800	2.24780100	0.64737500
H	1.22771400	2.38920200	1.58019000
H	0.49956000	3.83289800	0.86594400

H	2.30775800	5.70937300	-3.12176400
H	3.96212000	5.37599300	-2.60745500
H	3.38610000	4.76151800	-4.16435200
H	3.84540900	0.12133300	-3.64898500
H	3.38360100	-0.80183300	-2.22019000
H	2.21641900	-0.53238500	-3.49630100
Si	-2.77504200	-1.91967400	3.15951700
Si	4.37385100	-2.79024400	1.48192400
C	5.55241200	-3.30135400	0.09160700
C	2.99095500	-4.06659100	1.64092000
C	5.31854400	-2.60101100	3.11679700
C	-1.24671600	-2.52906500	4.10610000
C	-3.97121100	-1.12161800	4.40100800
C	-3.61068900	-3.36997500	2.27203500
H	3.38841200	-5.02164000	2.00679800
H	2.20970500	-3.73016400	2.32927200
H	2.52284900	-4.24810300	0.66762600
H	5.03443600	-3.31707700	-0.87404500
H	6.41618900	-2.63211800	0.01681300
H	5.92911300	-4.31435900	0.28217600
H	5.80152000	-3.54664200	3.39379500
H	6.09791300	-1.83347000	3.04080000
H	4.64193700	-2.31634600	3.93068100
H	-0.75302100	-1.68996100	4.60693400
H	-1.53216200	-3.27245200	4.86178200
H	-0.51785300	-2.99211100	3.43398700
H	-3.50012400	-0.26142200	4.89091600
H	-4.87875500	-0.77447600	3.89770200
H	-4.25706500	-1.83988200	5.18041100
H	-2.94726600	-3.79950400	1.51344200
H	-3.86974800	-4.16514400	2.98275200
H	-4.52905700	-3.03987900	1.77588000
C	-0.27420600	0.08429300	2.16335200
O	-0.21390900	0.55788200	3.32451500

W	-0.00048600	-2.03708200	-1.45375900
C	-0.18530600	-0.82033900	-3.08772200
O	-0.36142000	-0.11595400	-3.99022700

W-int5

127

C	-2.49273900	4.49194800	-1.31247800
N	-1.38520100	3.67344100	-0.84825200
O	-1.00667500	0.26657000	-1.79324800
C	-4.08555100	-3.53574800	-0.08845100
Si	0.89435400	2.01642200	-0.89702500
C	-0.50395900	4.14742500	-0.02937500
N	-3.59387600	-2.20469300	-0.39580900
Si	-2.15527100	-1.27691700	-1.31259100
N	0.59322200	3.35601500	0.24720300
C	-0.38310600	0.54532900	-0.71241900
C	-4.31804000	-1.09411000	-0.42678400
C	1.47049800	3.65650400	1.38886400
N	-3.57544400	-0.16091800	-1.06256500
C	-3.83510500	1.26732200	-1.14921300
C	-0.57359500	5.49551800	0.61751400
C	-1.62432900	5.80410700	1.49111000
C	0.40052800	6.46435100	0.33882600
C	-1.69660600	7.06422600	2.08675100
H	-2.37892700	5.05171900	1.70419600
C	0.31857800	7.72842000	0.92306000
H	1.21467100	6.22294300	-0.33819100
C	-0.72682800	8.02875300	1.80117200
H	-2.50919900	7.29341100	2.77061300
H	1.07255100	8.47708400	0.69663600
H	-0.78506900	9.01121200	2.26139300
C	-5.65908900	-0.89004300	0.14337600
C	-6.66027900	-0.23413700	-0.58881100
C	-5.92139400	-1.33864200	1.44767100
C	-7.91965400	-0.04166900	-0.02285800

H	-6.45629000	0.10554100	-1.59939100
C	-7.17771100	-1.12803800	2.01289300
H	-5.13196100	-1.81303600	2.02077400
C	-8.17793000	-0.48475500	1.27780500
H	-8.69777200	0.45517000	-0.59452200
H	-7.37534100	-1.46529300	3.02627800
H	-9.15810800	-0.32670300	1.71841100
H	2.28817200	4.32891100	1.10967600
H	0.89729200	4.11484500	2.19833600
H	1.90603600	2.72651800	1.76040800
H	-3.40870300	4.25945700	-0.75031300
H	-2.32149900	5.57361000	-1.23559200
H	-2.69450900	4.25343200	-2.36276400
H	-3.43151800	-4.26942500	-0.56656800
H	-4.07809000	-3.72948000	0.98845300
H	-5.10541600	-3.67806300	-0.46204400
H	-2.88546800	1.79700900	-1.22938000
H	-4.44012000	1.50847200	-2.03071800
H	-4.36109700	1.61791200	-0.25544300
P	2.92887400	0.11644400	0.70082200
P	-0.60835500	-0.99609900	0.36842700
C	0.78736900	-3.86863600	-0.16147200
C	3.45203000	-2.97975600	-0.03539400
C	1.63729000	-1.67378100	-1.84637600
O	4.49139500	-3.48769000	-0.14033900
O	0.27146000	-4.91060200	-0.31269500
O	1.62371700	-1.41935200	-2.98323300
C	-1.60344100	-0.85005800	1.92486700
C	-2.04262700	0.38122100	2.46305100
C	-2.03389700	-2.06529800	2.51776900
C	-2.93967000	0.37591100	3.53615100
C	-2.93074600	-2.01204100	3.59195700
C	-3.41343200	-0.80693300	4.10536200
H	-3.27426700	1.32970400	3.93811500

H	-3.25883600	-2.94816800	4.03878800
C	4.74671100	-0.10509800	0.92914000
C	5.25334700	-0.15763900	2.25090000
C	5.63086900	-0.30669400	-0.15951900
C	6.61541800	-0.39979700	2.45779400
C	6.98645300	-0.54320900	0.09744300
C	7.50105800	-0.59189900	1.39443800
H	6.99488400	-0.43936000	3.47743800
H	7.65830500	-0.70366600	-0.74400800
C	-1.55463200	1.70556000	1.94379600
C	-4.43459300	-0.77937900	5.21631400
C	-1.55999200	-3.42955500	2.07231400
C	4.36273700	0.03359900	3.46176800
C	5.16397500	-0.28297000	-1.59489000
C	8.97444100	-0.82056100	1.63885900
H	4.95554900	0.03074600	4.38278300
H	3.81283800	0.98034800	3.41241300
H	3.61402400	-0.76180200	3.54833700
H	9.41667700	-1.45161900	0.85988300
H	9.52771900	0.12866100	1.64055200
H	9.15123300	-1.30123700	2.60744000
H	4.28281200	-0.91536100	-1.74182900
H	4.88023300	0.73235900	-1.88800200
H	5.95216100	-0.63382700	-2.26879500
H	-0.46360300	1.75940700	1.96820300
H	-1.86382400	1.89532900	0.91118100
H	-1.93751200	2.53221000	2.55069200
H	-4.24414600	0.04178100	5.91581400
H	-5.44401100	-0.62955300	4.80927400
H	-4.44147000	-1.71657000	5.78196100
H	-2.18325800	-4.21360000	2.51244400
H	-1.57898800	-3.56196800	0.98971000
H	-0.52973100	-3.60974400	2.38818300
Si	0.98370600	2.79135200	-3.12212600

Si	-2.06078000	-2.45755000	-3.35243100
C	-3.85797700	-2.94663900	-3.72241200
C	-0.97881700	-3.99658900	-3.20053800
C	-1.40428000	-1.30708000	-4.69798000
C	2.40774500	1.88116900	-3.97820700
C	-0.60849100	2.52761900	-4.11113100
C	1.37866000	4.64547300	-3.01457500
H	-1.05217200	-4.58677000	-4.12293500
H	0.07075100	-3.72473000	-3.05451100
H	-1.26965700	-4.63561100	-2.36133000
H	-4.23227000	-3.68323800	-3.00326200
H	-4.52611900	-2.07769900	-3.69546200
H	-3.92654000	-3.39035200	-4.72357300
H	-1.36348900	-1.83629500	-5.65841500
H	-2.04491300	-0.42736200	-4.82261300
H	-0.39451000	-0.96566600	-4.45199000
H	2.23084900	0.80036600	-4.00025800
H	2.52676200	2.23031200	-5.01197100
H	3.34326500	2.05644100	-3.43788200
H	-0.80461200	1.46105900	-4.24524300
H	-1.46869800	2.96732000	-3.59908500
H	-0.51900500	2.99014500	-5.10261400
H	2.28367900	4.80753700	-2.41836000
H	1.54868700	5.06566400	-4.01407700
H	0.55508600	5.19833500	-2.54960100
C	2.68736700	1.47554800	-0.42695500
O	3.51777300	2.31480600	-0.82706600
W	1.65035900	-2.11074100	0.13766400
C	1.71546600	-2.40608400	2.16297400
O	1.74619600	-2.57014500	3.31112000

W-ts3

127

C	0.84527300	4.38111800	1.73378100
N	-0.11606400	3.68735600	0.91443900

O	1.31238300	1.25798900	1.53177400
Si	-1.29840800	1.96077800	0.80544300
C	-0.78328500	4.16395000	-0.11421200
N	3.97570900	-1.47889000	1.04978500
Si	2.49570400	-0.32503000	1.49157400
N	-1.60415500	3.18677500	-0.56174500
C	0.40417000	1.00882400	0.67279300
C	4.66345700	-0.43220300	0.61116200
C	-2.58136700	3.28645700	-1.63014900
N	3.88353100	0.65093200	0.83321800
H	-2.86969600	4.32872600	-1.79668800
H	-2.19221800	2.87681100	-2.57156400
H	-3.47448600	2.72019600	-1.35704000
H	1.35803900	5.17958200	1.18300400
H	0.37350100	4.82820500	2.62017700
H	1.58628600	3.65624900	2.08427600
P	-2.60562800	-0.63347600	-0.93207900
P	0.90902000	-0.65377700	-0.14825400
C	0.43200000	-3.73558900	0.68248100
C	-2.36284100	-3.75233200	0.13279900
C	-1.25459500	-1.81459000	1.91363400
O	-3.22820100	-4.52502700	0.21019700
O	1.24246200	-4.48608500	1.08549500
O	-1.48317600	-1.49676200	3.01047900
C	1.90311800	-0.32228500	-1.70027500
C	1.90399000	0.91738200	-2.38400400
C	2.74012700	-1.36727500	-2.17321500
C	2.78141500	1.10860600	-3.45966900
C	3.59622300	-1.12114100	-3.25224700
C	3.64940000	0.11063300	-3.90314900
H	2.77434100	2.06969900	-3.96916400
H	4.24529800	-1.92711400	-3.58878700
C	-4.29272800	-1.34724300	-1.19702000
C	-4.65373900	-1.61984200	-2.54158700

C	-5.18311500	-1.71086400	-0.15820400
C	-5.89182500	-2.20632900	-2.81964600
C	-6.41331400	-2.29483500	-0.48689400
C	-6.79312100	-2.54364600	-1.80622700
H	-6.15849900	-2.40893200	-3.85554800
H	-7.08954000	-2.57452300	0.31921500
C	0.97493100	2.05203200	-2.03698900
C	2.74813000	-2.77610600	-1.62560600
C	-3.72639300	-1.31276400	-3.70155900
C	-4.86361300	-1.50891100	1.30275400
C	-8.14098600	-3.14393000	-2.13079800
H	-4.20491200	-1.56236900	-4.65458500
H	-3.44426200	-0.25392400	-3.72958300
H	-2.79368400	-1.88666600	-3.64010400
H	-8.50510600	-3.77720900	-1.31433500
H	-8.89371200	-2.36077200	-2.29580200
H	-8.10113000	-3.75194300	-3.04153800
H	-3.82252400	-1.75440600	1.52427600
H	-5.00932200	-0.46433800	1.59176300
H	-5.50331000	-2.13596500	1.93207300
H	-0.06088300	1.71326600	-1.95685800
H	1.23477300	2.53609000	-1.08913300
H	1.01001600	2.82599500	-2.81001000
H	3.75882300	-3.19411600	-1.66027000
H	2.38776800	-2.85071000	-0.60523800
H	2.10324100	-3.41802100	-2.23540000
Si	-3.49644100	2.46245900	2.17109300
Si	2.25577700	-0.79970800	3.78734100
C	-3.31875700	1.31143200	3.66687400
C	-2.85514200	4.22111600	2.64764100
C	-5.35015700	2.87970900	1.94100700
H	-3.37386000	0.25995700	3.37088000
H	-2.35291100	1.46412600	4.16181600
H	-4.11698200	1.50296700	4.39644200

H	-1.87012900	4.16914500	3.12292400
H	-2.77077700	4.87028900	1.76751100
H	-3.56536400	4.69664800	3.33858800
H	-5.95902600	1.97950000	1.80206800
H	-5.73168200	3.42954800	2.81107000
H	-5.50351800	3.51593200	1.05880900
C	-2.65751500	0.70564000	0.16915600
O	-3.73427400	1.25442700	0.66255600
C	-0.57746600	-2.94945800	-1.94344400
O	-0.42027100	-3.25107200	-3.05325900
C	4.60253800	0.33793400	-5.05156300
H	4.62813600	1.39003500	-5.35243600
H	5.62176900	0.03421700	-4.78279300
H	4.31282200	-0.25385300	-5.92885200
C	5.99149100	-0.43053700	-0.02908100
C	7.07589400	-1.09055500	0.56831400
C	6.15792300	0.21084300	-1.26736000
C	8.31844300	-1.10006400	-0.06490900
H	6.95138900	-1.57714600	1.53038500
C	7.39926400	0.18444800	-1.90074900
H	5.31296900	0.69286500	-1.74588100
C	8.48039000	-0.46672700	-1.30006700
H	9.15799500	-1.60354400	0.40492600
H	7.52056600	0.67172500	-2.86383600
H	9.44757000	-0.48188500	-1.79415100
C	4.43233400	-2.84336300	1.22493700
C	4.08598200	2.01557000	0.38607800
H	3.57041400	-3.51279100	1.19199600
H	5.13355300	-3.12974700	0.43485000
H	4.92821200	-2.97177400	2.19586200
H	3.54214300	2.68538800	1.05465500
H	5.14881800	2.27309800	0.41228900
H	3.71101200	2.16425900	-0.63368300
C	3.97994000	-0.51044100	4.53174600

C	0.98278900	0.36121100	4.56149200
C	1.76553500	-2.61481100	3.99063600
H	3.97034900	-0.72410700	5.60795200
H	4.72735500	-1.16444500	4.06696100
H	4.30948100	0.52623300	4.39712700
H	-0.01165300	0.18543000	4.13938900
H	0.92528300	0.19746900	5.64498000
H	1.24430500	1.40987900	4.38386900
H	0.77716800	-2.81094900	3.56620800
H	2.47570600	-3.28938900	3.50117600
H	1.73285300	-2.87197500	5.05707400
C	-0.69092300	5.52523600	-0.68668000
C	-0.77734400	6.64938000	0.14974800
C	-0.50116800	5.70589100	-2.06611300
C	-0.67989600	7.93272800	-0.38783600
H	-0.94125100	6.51360800	1.21401500
C	-0.39346100	6.99035500	-2.59791200
H	-0.43123300	4.83900400	-2.71594600
C	-0.48425300	8.10584800	-1.76058500
H	-0.75899600	8.79715600	0.26523400
H	-0.24131300	7.12055700	-3.66563400
H	-0.40508300	9.10614700	-2.17693300
W	-0.86999600	-2.41407600	0.01112300

W-int6

127

C	3.20736600	2.77940200	-1.70052100
N	2.41127400	2.74835700	-0.48938500
O	-1.32121100	2.03679000	0.97764000
C	-5.08084400	0.12537400	-2.00519800
Si	1.39973400	1.49271400	0.53832400
C	2.44777100	3.57736000	0.55215200
N	-4.34363800	0.55284100	-0.83131400
Si	-2.57807000	1.47428800	-0.14158000
N	1.82753600	2.94267400	1.57096000

C	-0.29598700	1.10759600	0.66634800
C	-4.87095300	0.84601400	0.33197900
C	1.17422800	3.53520700	2.72825700
N	-3.95174300	1.53287100	1.05623500
C	-3.93885500	1.63597600	2.50885500
C	3.03808900	4.92057400	0.59089600
C	2.88586200	5.78030100	-0.51004800
C	3.75084500	5.35298000	1.72209500
C	3.44087300	7.05791900	-0.47622100
H	2.31284300	5.45151900	-1.37136500
C	4.31490300	6.62691000	1.74292000
H	3.87634900	4.68186700	2.56550000
C	4.15909900	7.48063800	0.64650300
H	3.31253600	7.72384000	-1.32437900
H	4.87503600	6.95421900	2.61384700
H	4.59567200	8.47505400	0.66769200
C	-6.22599100	0.51099900	0.82672400
C	-7.10162100	1.52651600	1.23549000
C	-6.62460500	-0.83061200	0.90812400
C	-8.37331900	1.20122000	1.70757600
H	-6.78533400	2.56384000	1.17117600
C	-7.89328100	-1.15131000	1.39116900
H	-5.92786200	-1.61107900	0.62104600
C	-8.76964800	-0.13702000	1.78679700
H	-9.05326300	1.99051500	2.01535600
H	-8.19596300	-2.19225600	1.46107500
H	-9.75863900	-0.38865400	2.15956300
H	1.61696800	3.15485300	3.65519100
H	1.27264800	4.62424300	2.71357200
H	0.11202100	3.27256300	2.70557900
H	2.60657100	3.10562800	-2.55465800
H	4.07126200	3.44272600	-1.59195900
H	3.56052600	1.76473600	-1.91248200
H	-4.40159200	-0.39778100	-2.68254100

H	-5.90605800	-0.54769100	-1.74842500
H	-5.49828700	0.98815900	-2.54228500
H	-3.08811900	2.25786700	2.78927000
H	-4.86043400	2.09674400	2.87881300
H	-3.81872800	0.65235900	2.97972500
P	2.18122500	-1.32112800	-0.42511200
P	-1.13240100	-0.32690100	-0.06541600
C	-1.45375000	-1.60058800	-3.09355800
C	1.25059900	-2.29075700	-3.46779600
C	0.57648900	0.36418400	-2.76949500
O	1.83309900	-2.77520700	-4.35946600
O	-2.42757400	-1.64769800	-3.73909300
O	0.81349000	1.39119000	-3.26404200
C	-1.96786000	-1.35928400	1.28250500
C	-1.71225200	-1.23380800	2.67439500
C	-2.97547600	-2.27065400	0.86337400
C	-2.46665600	-1.99425100	3.58143600
C	-3.70047100	-2.99943100	1.81214000
C	-3.47142600	-2.87323300	3.18150400
H	-2.25768100	-1.88452600	4.64393800
H	-4.46856200	-3.68843300	1.46361500
C	3.18927000	-2.74652000	0.13393000
C	2.92048400	-3.36657200	1.37701300
C	4.22321600	-3.23843600	-0.69541800
C	3.72219200	-4.43941900	1.78165300
C	5.00188000	-4.30899800	-0.24296300
C	4.77451700	-4.91723200	0.99482400
H	3.51470000	-4.91785400	2.73674200
H	5.80443200	-4.67988200	-0.87741200
C	-0.68056300	-0.30658700	3.28093000
C	-4.30048000	-3.63942500	4.18431600
C	-3.31536900	-2.52992400	-0.58753100
C	1.80305800	-2.90187200	2.28175900
C	4.50034600	-2.66074000	-2.06642200

C	5.65275500	-6.04718300	1.47784600
H	1.63942800	-3.61353000	3.09685100
H	2.04729000	-1.93036400	2.72405400
H	0.85535200	-2.78838100	1.74333200
H	6.05663300	-6.62753200	0.64142100
H	6.50721400	-5.66261600	2.05103600
H	5.10243400	-6.73017400	2.13385700
H	3.75898200	-3.00332200	-2.79530600
H	4.46727500	-1.56645200	-2.06842400
H	5.48730800	-2.96810500	-2.42689700
H	0.31306300	-0.46738900	2.86259300
H	-0.93079900	0.74164800	3.09422400
H	-0.62962100	-0.46524100	4.36372300
H	-3.82253200	-3.66143900	5.16931800
H	-5.29069800	-3.18039700	4.30797600
H	-4.46333600	-4.67434200	3.86173800
H	-4.34553300	-2.88974600	-0.68473000
H	-3.21628200	-1.64213500	-1.20426600
H	-2.66207300	-3.29653100	-1.01474700
Si	5.02769900	0.17072900	1.81011400
Si	-2.46557600	3.02681800	-1.89759300
C	-3.97294100	4.17900800	-1.73964400
C	-2.54451400	2.16742500	-3.58809100
C	-0.86943600	4.03967300	-1.72857200
C	5.85696000	-0.09485300	0.14675900
C	5.26485200	1.95088800	2.37887300
C	5.59880100	-1.06811900	3.09672000
H	-2.28290800	2.87297200	-4.38705000
H	-1.85662000	1.31996800	-3.65268100
H	-3.55354300	1.79236000	-3.78905900
H	-4.90690600	3.60575400	-1.78887300
H	-3.96456200	4.72174700	-0.78708600
H	-3.98939500	4.91859200	-2.55045100
H	-0.83077200	4.84795100	-2.47034000

H	-0.80332600	4.48109500	-0.72762200
H	0.00584900	3.39841000	-1.86969200
H	5.31482000	0.42095900	-0.65427700
H	6.88080600	0.29852900	0.16597400
H	5.90163200	-1.15837200	-0.10421600
H	6.30796500	2.14190300	2.66007500
H	5.00061900	2.65371400	1.58059400
H	4.63146300	2.17262100	3.24561100
H	5.37365500	-2.08588600	2.75689900
H	6.67813100	-0.99298400	3.27616200
H	5.08495800	-0.90962900	4.05204100
C	2.46742600	-0.04037700	0.65695600
O	3.35880800	-0.10772000	1.68594700
W	0.19569800	-1.47786300	-1.98689100
C	-0.08911200	-3.33412000	-1.17339100
O	-0.22247700	-4.38908300	-0.70885200

W-ts4

127

N	-0.64619500	2.89923500	0.83009500
O	1.56386400	1.47053900	0.09489900
Si	-0.37494500	1.55684500	-0.47167900
C	-0.64889800	3.81918200	-0.12445500
N	4.06405200	0.22135400	-0.44330200
Si	2.61504000	0.26671800	0.61656500
N	-0.36305500	3.18097800	-1.27956900
C	0.74072800	0.33059900	-1.09507600
C	4.87763900	1.27252300	-0.05507300
N	4.34222600	2.06343900	0.81798200
P	-2.49599200	-0.64831200	-0.07770500
P	1.03523000	-1.23593100	-0.23258100
C	0.17906800	-3.55891200	2.10432800
C	-2.58447800	-3.03616400	1.95052600
C	-0.76048000	-0.86271000	2.49872200
O	-3.53787900	-3.46899600	2.46550900

O	0.82472800	-4.31603000	2.71533700
O	-0.67632100	-0.02659100	3.30700000
C	1.74379800	-2.44992200	-1.43735700
C	1.16594900	-2.61147400	-2.71860700
C	2.77549400	-3.32336500	-1.02450800
C	1.67436300	-3.59719800	-3.57241400
C	3.24476300	-4.29914900	-1.90978800
C	2.71806900	-4.44501400	-3.19546300
H	1.22687500	-3.71552100	-4.55757700
H	4.04096900	-4.96540000	-1.58281000
C	-4.30791500	-0.89611600	-0.03905200
C	-4.91273600	-1.73288500	-1.00210600
C	-5.08521100	-0.31273100	0.98710900
C	-6.29593900	-1.93678100	-0.94616200
C	-6.46184300	-0.55194800	1.00682100
C	-7.08839500	-1.35024400	0.04482500
H	-6.76433100	-2.57465100	-1.69291600
H	-7.06214000	-0.10421100	1.79628500
C	-0.00506300	-1.78039300	-3.19610300
C	3.39909400	-3.23164200	0.35050700
C	-4.10580800	-2.43293100	-2.07383300
C	-4.46486000	0.55682600	2.05683300
H	-4.76042200	-2.87958500	-2.82853900
H	-3.41704400	-1.75313800	-2.58783500
H	-3.49508600	-3.23658200	-1.64783200
H	-3.62902500	0.05014200	2.55353600
H	-4.07641400	1.48451200	1.62178900
H	-5.20077500	0.81936700	2.82306900
H	-0.82106300	-1.78437200	-2.46416600
H	0.27802900	-0.73285900	-3.34010300
H	-0.40015700	-2.17370500	-4.13841000
H	4.09738600	-4.05734200	0.51877900
H	3.95866800	-2.29637800	0.48088600
H	2.64323800	-3.27515600	1.13913400

Si	-4.05385600	2.32178100	-2.03366300
Si	3.20167800	-0.01490400	2.89033500
C	-2.19488400	0.97624600	-0.46519300
O	-3.18319100	1.90939300	-0.62873400
W	-0.95482800	-2.28854600	1.06649500
C	-1.10524900	-3.68381500	-0.42826900
O	-1.18301900	-4.48469600	-1.26316700
C	2.50149700	-1.57252000	3.71051200
C	5.09090100	-0.18380700	3.04231200
C	2.53685300	1.49337200	3.83171600
H	1.41138300	-1.61042100	3.69397000
H	2.87399200	-2.49069300	3.24732200
H	2.82046100	-1.57958300	4.76168700
H	5.34756900	-0.44845300	4.07615200
H	5.45417600	-0.99283200	2.39673000
H	5.63507600	0.72510600	2.77666800
H	1.45745300	1.37792800	3.98128400
H	3.00496500	1.57877400	4.82047200
H	2.70874900	2.42556500	3.28460800
C	4.17259400	-0.35324800	-1.79294500
C	5.07698800	3.08728700	1.53281400
C	-0.02895500	3.74158200	-2.57046800
C	-1.04258500	3.02291000	2.21447700
H	-0.56105900	4.68294500	-2.73929200
H	1.04905800	3.92541000	-2.65794500
H	-0.31577600	3.03243400	-3.35033600
H	-2.05986100	2.64181400	2.36614000
H	-0.36412300	2.43034500	2.83040700
H	-1.00221800	4.06725400	2.53636900
H	3.20721600	-0.29071700	-2.30017200
H	4.90726700	0.20636500	-2.37626500
H	4.48147600	-1.40156300	-1.76266100
H	4.85018300	3.01023600	2.60241900
H	6.16684700	3.03358300	1.40955800

H	4.74405400	4.08136200	1.20632200
C	-0.96320300	5.25331100	0.02149500
C	-0.07253000	6.22876100	-0.44877900
C	-2.17280900	5.63777800	0.61981200
C	-0.38949800	7.58045400	-0.31358100
H	0.86845500	5.92932600	-0.89982400
C	-2.48895500	6.99006600	0.74066300
H	-2.86666800	4.87649000	0.96185500
C	-1.59712900	7.96151300	0.27725200
H	0.30572400	8.33478600	-0.66989900
H	-3.43014100	7.28498100	1.19507600
H	-1.84352200	9.01477300	0.37616400
C	6.26692300	1.35207100	-0.59225700
C	6.75498000	2.53951600	-1.15428700
C	7.10406900	0.22821200	-0.52080300
C	8.06317000	2.60329400	-1.63664800
H	6.10274500	3.40542400	-1.22080800
C	8.41434100	0.29710100	-0.99166600
H	6.71968300	-0.69218100	-0.09089500
C	8.89528000	1.48425000	-1.55262700
H	8.43158000	3.52512600	-2.07805100
H	9.05918600	-0.57468900	-0.92474400
H	9.91466500	1.53553200	-1.92508000
C	-5.85324000	1.85187600	-1.78061900
C	-3.89935800	4.18796100	-2.20885300
C	-3.28483300	1.41620000	-3.49120100
C	-8.58481500	-1.55263000	0.06444600
C	3.26545600	-5.48030000	-4.14960800
H	-9.09646500	-0.73874000	-0.46682600
H	-8.87012900	-2.49116400	-0.42220400
H	-8.97337600	-1.56666200	1.08852100
H	2.49463000	-5.83342400	-4.84331700
H	4.08364600	-5.06706300	-4.75524900
H	3.66448700	-6.34842700	-3.61367500

H	-3.73072200	1.75309200	-4.43478800
H	-2.20424600	1.59040900	-3.54519400
H	-3.44118000	0.33545600	-3.41290800
H	-6.47081600	2.24514500	-2.59836600
H	-5.98480800	0.76655700	-1.73760500
H	-6.23421800	2.26820300	-0.84106400
H	-4.27678800	4.69993600	-1.31635800
H	-2.85872900	4.49604100	-2.34832400
H	-4.47947300	4.54509400	-3.06877500

W-int7

127

N	0.75838500	3.51919900	0.41379800
O	0.69340100	0.71752400	1.11252300
Si	-0.14641200	0.99275900	-0.32681000
C	1.30012300	3.21949200	-0.70847200
N	3.37579100	-0.22631500	0.45981400
Si	1.79865900	-0.52023300	1.26144700
N	0.80827300	2.08080800	-1.34690700
C	-0.30687000	-0.61034800	-1.21040900
C	4.12471900	0.70018000	1.18265500
N	3.49508400	1.26533400	2.15420100
P	-2.96626400	0.24856000	-0.08577900
P	0.65460500	-1.75416400	-0.40269500
C	-1.37181200	-3.98194100	0.18105100
C	-3.79494600	-2.67310000	0.42024800
C	-1.64835700	-1.59342900	1.93426600
O	-4.87351500	-3.02019300	0.67594900
O	-1.07990300	-5.10092700	0.29683700
O	-1.58029900	-1.28322200	3.05205200
C	1.45589900	-3.11430400	-1.34344700
C	1.55654200	-3.10559900	-2.75990300
C	2.00152000	-4.19657700	-0.61049200
C	2.15156300	-4.20427600	-3.39102300
C	2.58554900	-5.26678300	-1.29325700

C	2.66236800	-5.29681000	-2.68616300
H	2.22038000	-4.20153900	-4.47686500
H	2.98776300	-6.09825100	-0.71834500
C	-4.68784200	0.71473100	0.28205700
C	-5.68403700	0.42007400	-0.67975600
C	-5.03472600	1.34009200	1.50343200
C	-7.00286000	0.80450700	-0.42481000
C	-6.37003600	1.70619800	1.70853300
C	-7.36400400	1.46035100	0.75663800
H	-7.76805700	0.58557700	-1.16647500
H	-6.64209600	2.18818300	2.64523400
C	1.09120400	-1.96623300	-3.63885400
C	2.01688700	-4.21746500	0.89923200
C	-5.35650300	-0.30174500	-1.96919800
C	-4.02113100	1.61136700	2.59178200
H	-6.23247200	-0.34888700	-2.62304800
H	-4.54978600	0.19243600	-2.52261600
H	-5.03002300	-1.33035300	-1.77686300
H	-3.42887300	0.71999800	2.82641200
H	-3.31876200	2.39499900	2.28955600
H	-4.51912600	1.93554700	3.51064200
H	0.01040100	-1.82548600	-3.59671900
H	1.53079800	-1.01467600	-3.32481000
H	1.38371200	-2.15287900	-4.67726300
H	2.26657900	-5.21421600	1.27467900
H	2.76913500	-3.52146000	1.29336100
H	1.05530300	-3.93545900	1.33075600
Si	-2.96072200	4.04486600	-0.79076600
Si	2.00216000	-1.26520400	3.49437800
C	-1.92060400	1.58115300	-0.01035400
O	-2.30146700	2.84795400	0.22157000
W	-1.93425000	-2.06397300	-0.02577300
C	-2.26513900	-2.30039900	-2.04854900
O	-2.46776500	-2.40023800	-3.18144500

C	1.16020100	-2.91736700	3.91115300
C	3.84481200	-1.52701800	3.87785400
C	1.21842100	0.09544600	4.54931000
H	0.17479000	-3.04031000	3.45701400
H	1.78414600	-3.76950900	3.62441200
H	1.02603700	-2.95804000	5.00028700
H	3.95257800	-1.94851600	4.88548100
H	4.28714900	-2.24244200	3.17329900
H	4.42592200	-0.60334300	3.82740000
H	0.13502400	0.10102300	4.39100700
H	1.40968000	-0.07901800	5.61587500
H	1.60322600	1.08191500	4.27704500
C	3.68159000	-0.53555100	-0.94236000
C	4.13799900	2.10354900	3.14474400
C	0.90935100	1.93611800	-2.80210800
C	1.30763800	4.50382100	1.31641600
H	0.52616600	2.82410300	-3.32103800
H	1.94342700	1.77430900	-3.12920800
H	0.31484000	1.07048400	-3.09906100
H	0.55532600	5.27781800	1.51986900
H	1.52460800	4.01174800	2.27380200
H	2.22496800	5.00042900	0.96807300
H	2.96021400	-0.05856900	-1.61728600
H	4.67711700	-0.17171600	-1.20172100
H	3.65983300	-1.61333000	-1.11998300
H	3.82532700	1.77601500	4.14340700
H	5.23559300	2.09974800	3.10991700
H	3.79385400	3.13913200	3.02660200
C	2.44172600	3.93571300	-1.36130400
C	3.65170400	3.26671600	-1.58960900
C	2.32450500	5.28267900	-1.72848800
C	4.73240900	3.93453300	-2.16539500
H	3.74345100	2.22563000	-1.29900700
C	3.40135500	5.94845300	-2.31759100

H	1.38622100	5.80142300	-1.55415600
C	4.60738500	5.27697100	-2.53494000
H	5.67060900	3.40794100	-2.31726200
H	3.29937600	6.99165700	-2.60405900
H	5.44627700	5.79747100	-2.98887700
C	5.56825800	0.88527900	0.84312800
C	6.08480000	2.16294800	0.58892800
C	6.42720000	-0.22389300	0.80838200
C	7.43859900	2.32809500	0.29129800
H	5.42052600	3.02054000	0.60732000
C	7.78195400	-0.05567500	0.52406800
H	6.02434900	-1.21321100	1.00633100
C	8.28924500	1.22043200	0.26023500
H	7.82668100	3.32179200	0.08551200
H	8.44116900	-0.91904500	0.50528900
H	9.34355300	1.34989500	0.03197900
C	-4.16205300	4.96192100	0.32090300
C	-1.56995900	5.15588600	-1.39480300
C	-3.81950400	3.21790000	-2.24359400
C	-8.78835000	1.90360400	0.99081500
C	3.31417900	-6.45140100	-3.40845000
H	-8.95625900	2.90953900	0.58315800
H	-9.50284900	1.23178200	0.50328100
H	-9.02667200	1.94088800	2.05899700
H	2.84868200	-6.62695700	-4.38420600
H	4.37938000	-6.25181200	-3.58724600
H	3.24842400	-7.37655200	-2.82630100
H	-4.10849300	3.96869000	-2.98938000
H	-3.15657100	2.49585400	-2.73481600
H	-4.72173900	2.68720300	-1.92396800
H	-4.63811000	5.79738200	-0.20649900
H	-4.94783600	4.28113800	0.66868400
H	-3.64908800	5.36613200	1.20137500
H	-0.98170200	5.53328100	-0.55229500

H	-0.88985500	4.60114100	-2.05036900
H	-1.96856200	6.01006400	-1.95651100

4'

90

W	1.40904300	-1.22691900	-0.76947800
P	2.21465700	1.91806100	1.15566100
C	3.95474200	1.30080100	1.02297300
C	4.56073100	0.66209800	2.12806700
C	5.88113300	0.21274200	2.01772900
H	6.33823800	-0.29425300	2.86579300
C	6.62740100	0.39763500	0.85061700
C	6.02654700	1.07311500	-0.21546100
H	6.59672000	1.23836100	-1.12760500
C	4.70708700	1.52983500	-0.15099300
C	3.81454600	0.45124700	3.42925100
C	8.03633800	-0.13522800	0.73407500
C	4.11852000	2.26393100	-1.33728400
P	-0.87279800	-1.51798900	0.18750100
C	-2.04096600	-2.90883700	0.37097700
C	-2.48373000	-3.60967000	-0.77580700
C	-3.42359200	-4.63263100	-0.61206500
H	-3.77913900	-5.16255200	-1.49318700
C	-3.91767600	-4.99322300	0.64502300
C	-3.45076200	-4.29726700	1.76443800
H	-3.82192500	-4.56785400	2.75069500
C	-2.52014400	-3.25923700	1.65509200
C	-1.98076300	-3.29610700	-2.16874300
C	-4.90473500	-6.12611400	0.79335600
C	-2.06614200	-2.53776000	2.90436100
Si	-0.49303600	1.34755200	0.35752200
N	-1.14611600	2.50361500	-0.91648800
C	-1.49937300	2.50645500	-2.32492700
C	-1.47447600	3.39491600	0.03381200
C	-2.11121400	4.70310500	-0.17301400

C	-3.15013600	5.12273900	0.67423800
H	-3.49244200	4.47141500	1.47135600
C	-3.75889400	6.35859300	0.46589800
H	-4.56964000	6.67596300	1.11456300
C	-3.32624000	7.18540100	-0.57490600
H	-3.79867900	8.15072200	-0.73160700
C	-2.28749300	6.77401800	-1.41498900
H	-1.94754800	7.41912000	-2.21934700
C	-1.68374900	5.53304800	-1.22210500
H	-0.86900100	5.21509200	-1.86440900
N	-1.11013900	2.85152200	1.20661800
C	-1.03561700	3.47119500	2.51708400
C	-1.66405200	-0.07509100	0.60225600
O	-2.91342600	0.09221100	1.11000700
Si	-4.43651900	0.12582500	0.34824200
C	-5.54331800	-0.96672900	1.39540900
H	-6.57414000	-0.96251300	1.02054900
H	-5.56236800	-0.62500600	2.43696700
H	-5.17671800	-1.99941700	1.38417100
C	-4.28890900	-0.49687700	-1.41702200
H	-4.16673500	-1.58356200	-1.44159800
H	-3.43338400	-0.04844100	-1.93443300
H	-5.19319100	-0.24206000	-1.98308600
C	-4.99687300	1.92277800	0.38380600
H	-6.02235400	2.02866400	0.00909000
H	-4.34833700	2.55238000	-0.23633200
H	-4.97040500	2.31832900	1.40621400
O	1.47356200	-4.19191500	-1.88049400
O	-0.02612500	-0.21521100	-3.44430400
O	4.21039200	-0.82383400	-2.19296400
O	2.84940400	-2.28612600	1.90387300
C	1.45722900	-3.09362700	-1.47491300
C	0.51305500	-0.56165000	-2.47006300
C	3.19450200	-0.92602900	-1.63277000

C	2.32741500	-1.88726500	0.95005200
C	1.24535200	0.83991300	0.28283900
H	8.04147100	-1.13267400	0.27448300
H	8.66039400	0.51222900	0.10774100
H	8.51535200	-0.22576000	1.71522100
H	4.46366800	-0.00928100	4.18120300
H	3.44552700	1.39951100	3.84065100
H	2.94796300	-0.20397400	3.29552800
H	3.20556300	1.77798400	-1.69390300
H	3.85411800	3.29732100	-1.07597600
H	4.83015700	2.29739400	-2.16796200
H	-0.97423700	-3.69840600	-2.31943500
H	-2.63566900	-3.74244900	-2.92353000
H	-1.92826800	-2.22092800	-2.36504100
H	-4.38227800	-7.08291500	0.92477800
H	-5.55031800	-5.98461300	1.66664600
H	-5.54112300	-6.22229000	-0.09276600
H	-0.97259900	-2.50666500	2.97971000
H	-2.42689100	-1.50317600	2.90979600
H	-2.44857800	-3.03521100	3.80075000
H	-1.70922200	1.48354900	-2.64229600
H	-2.38219100	3.12725200	-2.50253800
H	-0.67082300	2.88002500	-2.93629400
H	-0.08009100	3.20704500	2.97786500
H	-1.09549900	4.55959200	2.43367100
H	-1.84981400	3.11739000	3.15952700

XYZ coordinates obtained for the reaction of 1’ with Mo(CO)$_6$ at the B3LYP-D3/6-31G* Mo (LanL2DZ) level of theory.

1'

57

Si	1.65615400	-0.46126400	0.15919200
N	0.46116800	0.39864000	-1.00773500

C	-1.14821900	2.11708300	-0.13989000
P	1.27743900	-2.51965200	0.59126700
Si	3.80011100	0.41598300	-0.24165800
N	0.68857400	0.89501700	1.06526600
C	-1.02857900	3.44672700	0.28756500
H	-0.08078700	3.80615400	0.67694300
C	-2.12170700	4.30729500	0.19219700
H	-2.02475800	5.34008300	0.51400200
C	-3.33822700	3.84052800	-0.31286900
H	-4.18997100	4.51152400	-0.38007800
C	-3.46160100	2.51181500	-0.72865000
H	-4.40898500	2.14581100	-1.11402000
C	-2.36968900	1.64922400	-0.64959700
H	-2.46454100	0.60946400	-0.94677200
C	-0.01656700	1.17751200	-0.03166900
C	0.03213400	0.35431200	-2.38927100
C	4.33055600	0.00695400	-2.01698100
H	5.32608200	0.41791300	-2.22845800
H	3.63097200	0.42910100	-2.74829200
H	4.36940300	-1.07640300	-2.17643000
C	0.56620100	1.49280600	2.37535300
C	-0.58954300	-2.32008900	0.37657000
C	-1.19315900	-2.55817000	-0.88457800
C	-2.55961700	-2.30005900	-1.06651800
H	-3.00210400	-2.47679500	-2.04589800
C	-3.37000700	-1.82986400	-0.03091700
C	-2.78360700	-1.66984100	1.22680100
H	-3.40356500	-1.34268200	2.06032100
C	-1.42474800	-1.91981100	1.45210500
C	5.05767400	-0.29523700	0.98393800
H	6.06119400	0.10177700	0.78444500
H	5.10447000	-1.38751500	0.90832000
H	4.79344100	-0.04370200	2.01740500
C	3.67891500	2.30112200	-0.03726600

H	4.62918700	2.78849700	-0.28885400
H	3.42354700	2.57021700	0.99435900
H	2.90385800	2.71622500	-0.69273200
C	-0.40598300	-3.10444200	-2.05571000
C	-4.82528400	-1.49191800	-0.25623900
C	-0.89679300	-1.78908900	2.86286400
H	1.00253400	0.81281200	3.11101900
H	1.10380700	2.44782600	2.43813000
H	-0.48353800	1.66474500	2.63733900
H	-0.80815400	-0.33509600	-2.52607100
H	-0.26849200	1.34897000	-2.73505300
H	0.86610900	0.00689600	-3.00460600
H	-1.67895900	-1.43861300	3.54531800
H	-0.52945800	-2.75490900	3.23056700
H	-0.04997600	-1.10290800	2.92036100
H	-1.05102400	-3.24112400	-2.93107200
H	0.42392400	-2.44807000	-2.33473000
H	0.04631400	-4.07169200	-1.80807600
H	-5.45849000	-1.87995100	0.55030900
H	-4.97913100	-0.40403000	-0.28587500
H	-5.19329200	-1.90255400	-1.20246700

Mo(CO)6

13

C	1.35582700	-0.65586800	1.43451800
C	1.46422000	-0.17947400	-1.46596500
C	-0.58620900	-1.96532500	-0.34489500
C	-1.35563300	0.65612500	-1.43393700
O	-0.90762900	-3.05219200	-0.53699700
O	-2.10541600	1.01501800	-2.22798000
O	2.10423600	-1.01708100	2.22881100
O	2.27733100	-0.27719700	-2.27267700
C	-1.46396600	0.17934200	1.46650400
O	-2.27681500	0.27763800	2.27341200
C	0.58652900	1.96547200	0.34518700

O	0.90711700	3.05332000	0.53306300
Mo	0.00011400	0.00005500	0.00024900

Mo-int1

68

P	-0.81402700	-0.40728200	0.88760300
Si	0.39982700	1.35015100	0.54785000
Si	-0.37790300	3.52635000	0.99565500
N	2.11125100	1.01922500	1.16138400
C	0.46448600	-1.75424000	0.63555500
C	0.82879600	-2.49928200	1.79360400
C	1.85518400	-3.44566000	1.71926500
H	2.12882300	-3.99655200	2.61730700
C	2.54200000	-3.70469600	0.52801300
C	2.14779900	-3.00223800	-0.60821800
H	2.65310200	-3.19621900	-1.55333100
C	1.11993400	-2.04606400	-0.58528000
C	0.14082400	-2.30226700	3.12946400
C	3.64289400	-4.73760400	0.47135300
C	0.78192100	-1.40953200	-1.91639800
C	-1.52346800	4.06617600	-0.41323700
H	-1.76694200	5.13116500	-0.30890900
H	-2.46198900	3.50419600	-0.39654200
H	-1.06130800	3.91824400	-1.39548400
C	1.17785700	4.61466800	1.01178700
H	0.91218700	5.66144500	1.20535100
H	1.69697600	4.57265700	0.04679800
H	1.88393100	4.29554500	1.78686200
C	-1.27299900	3.60128500	2.65882000
H	-1.57964300	4.63217400	2.87730400
H	-0.63195100	3.25804800	3.47873500
H	-2.17290200	2.97795500	2.64389500
N	1.61776000	1.51808400	-0.86583800
C	1.65713500	1.87584100	-2.26865200
C	2.74581500	0.49685600	2.35505200

C	2.62881300	1.15207200	-0.06894100
C	4.01860300	0.87781400	-0.47433900
C	4.59003300	-0.36544800	-0.15765800
H	4.00483000	-1.11150600	0.37007800
C	5.89081400	-0.65204900	-0.56918200
H	6.32693900	-1.61879700	-0.33533400
C	6.62742800	0.29834200	-1.28184300
H	7.64254300	0.07342600	-1.59621800
C	6.05949300	1.53648800	-1.59561500
H	6.63163600	2.27510500	-2.14902400
C	4.75355700	1.82569800	-1.20155500
H	4.30930800	2.78697200	-1.44123400
H	0.05735500	-0.60420100	-1.82529300
H	1.68313800	-1.01029900	-2.39492900
H	0.36032000	-2.16081800	-2.59395100
H	4.21857300	-4.76182700	1.40356400
H	3.23274700	-5.74473300	0.31875100
H	4.33588800	-4.54006000	-0.35388500
H	0.31630400	-1.29979100	3.53562300
H	-0.94579800	-2.41563400	3.04491400
H	0.50190200	-3.03478600	3.85933900
H	2.28673800	0.96187200	3.23110600
H	2.61561500	-0.58877700	2.42750500
H	3.81491000	0.72924800	2.35307000
H	1.83708900	2.95037100	-2.40048100
H	2.44501300	1.32394900	-2.79052900
H	0.69422800	1.63094600	-2.72062200
C	-2.29564200	0.68691400	-1.91755900
C	-3.74221300	1.13517700	0.56338300
C	-4.85998400	-0.64966900	-1.42878400
C	-2.41330100	-2.21141500	-1.43870300
O	-5.87336300	-0.72372300	-1.99407500
O	-2.03165000	-3.16706200	-1.96284700
O	-1.79099900	1.37342600	-2.70542800

O	-4.10465400	2.06237300	1.15597300
C	-3.73846800	-1.72173000	1.08992200
O	-4.05972400	-2.38698500	1.97698100
Mo	-3.10118900	-0.51855000	-0.47339900

CO

2

C	0.00000000	0.00000000	-0.65022600
O	0.00000000	0.00000000	0.48766900

Mo-ts1'

70

P	0.87498700	0.20763500	-0.39647800
Si	-1.30715000	1.46434000	0.20135700
Si	-1.37808100	3.54341700	1.32556300
N	-2.44548200	1.21169800	-1.22926300
C	0.04701600	-1.14207600	0.70245700
C	-0.62289500	-2.21254500	0.06391000
C	-1.11553400	-3.26878500	0.84090100
H	-1.62649500	-4.09353300	0.34567600
C	-0.95308800	-3.30651900	2.23074700
C	-0.30537200	-2.23096700	2.84425800
H	-0.18045400	-2.23553200	3.92565900
C	0.18946700	-1.14536900	2.10931700
C	0.39788400	4.15490100	1.53297800
H	0.42214600	5.04489400	2.17410100
H	0.83109800	4.41673600	0.56259700
H	1.04044200	3.39239100	1.98770300
C	-2.15866800	3.20245400	3.02213800
H	-2.17007600	4.12165700	3.62141000
H	-1.59558100	2.44876100	3.58558100
H	-3.19322100	2.85334200	2.92588600
C	-2.43068200	4.78004100	0.35402400
H	-2.50671800	5.73340300	0.89240800
H	-3.44599800	4.39979700	0.19285800
H	-1.98059200	4.97320800	-0.62558000

N	-2.69394400	0.36771300	0.73195400
C	-3.21381600	-0.26766100	1.92746700
C	-2.34173400	1.30288300	-2.67643300
C	-3.20166100	0.36050100	-0.51000100
H	-2.55079400	2.32375500	-3.00792400
H	-1.32771700	1.03792700	-2.99136100
H	-3.04629100	0.61762400	-3.15340100
H	-4.29643400	-0.40472500	1.84953000
H	-2.73723600	-1.23792500	2.08965600
H	-3.00143500	0.36990000	2.78937600
C	3.54558700	1.02791200	-2.13812000
C	2.97355900	-1.81022500	-1.88080900
C	5.43634600	-0.65507700	-0.71087400
C	3.34305600	-1.65337800	0.99363200
O	6.57859200	-0.85234900	-0.79519500
O	3.28264000	-2.40983500	1.86485000
O	3.57607300	1.77156600	-3.02132100
O	2.65743100	-2.64539800	-2.61658900
C	0.57584100	1.80780300	-1.04493500
O	0.03097500	2.79838400	-1.47236600
C	3.69643100	1.25073300	0.77008700
O	3.75397300	2.12666300	1.52281700
C	-4.33103600	-0.44764000	-0.98809700
C	-4.41081200	-1.80368800	-0.62836800
C	-5.32058200	0.12119400	-1.80611200
C	-5.47031800	-2.58111000	-1.09171500
H	-3.62949100	-2.24500000	-0.01724200
C	-6.38299300	-0.66082700	-2.25552300
H	-5.26233200	1.17285500	-2.06956600
C	-6.45742800	-2.01133000	-1.90134700
H	-5.52397000	-3.63208500	-0.82372700
H	-7.15205000	-0.21740100	-2.88079300
H	-7.28396100	-2.61947700	-2.25731800
C	-1.44356900	-4.48488700	3.03815300

C	0.84900500	-0.00638500	2.85789200
C	-0.82426200	-2.26004800	-1.43742700
H	0.27926400	0.92736200	2.76511900
H	0.93197200	-0.23664700	3.92445300
H	1.85088300	0.20806100	2.47753400
H	-2.32909300	-4.94309800	2.58381400
H	-0.67208200	-5.26357700	3.10255600
H	-1.69676900	-4.19315400	4.06311300
H	-1.47157700	-3.09821900	-1.71550300
H	-1.28293600	-1.34086900	-1.81633800
H	0.12650500	-2.38046900	-1.96772400
Mo	3.46355900	-0.30596800	-0.56304500

Mo-int2'

70

P	0.98446500	-0.41874200	-0.39237500
Si	-1.64005600	1.51674300	0.20886400
Si	-1.81938300	3.61043900	1.25830800
N	-2.61894700	1.26009700	-1.31395000
C	0.22868600	-1.37763700	0.99949500
C	-0.44350600	-2.58803400	0.69038800
C	-0.95987500	-3.37685400	1.72307200
H	-1.46900300	-4.30585800	1.47303600
C	-0.84319400	-3.00672300	3.06602900
C	-0.18323400	-1.81171500	3.35920300
H	-0.07244600	-1.51132900	4.39925200
C	0.36189300	-0.99376500	2.35808400
C	-0.63115000	-3.05642700	-0.73788800
C	-1.43825300	-3.86256100	4.15920600
C	1.10719000	0.25079200	2.78867000
C	-0.19120600	3.93721500	2.15924900
H	-0.14424900	4.98173100	2.49139600
H	0.66355800	3.74779700	1.50102600
H	-0.08624800	3.29931100	3.04394400
C	-3.27892500	3.53805000	2.46396700

H	-3.40436000	4.50076300	2.97486600
H	-3.12225400	2.77034700	3.23082500
H	-4.21664600	3.30948800	1.94392400
C	-2.12012900	4.89201300	-0.10063700
H	-2.18112800	5.89992200	0.32876000
H	-3.05517600	4.69736000	-0.63892600
H	-1.29767200	4.87643400	-0.82373500
N	-2.93408300	0.24564800	0.55161300
C	-3.28965200	-0.71583000	1.57942400
C	-2.63510500	1.74452500	-2.67992400
C	-3.33634900	0.28704100	-0.73167900
C	-4.31378400	-0.60405600	-1.37266700
C	-4.00483100	-1.20443900	-2.60455200
H	-3.04798300	-1.00806500	-3.07750200
C	-4.91434800	-2.07858300	-3.19728000
H	-4.66823600	-2.55123900	-4.14317000
C	-6.13620200	-2.34629200	-2.57378400
H	-6.84522000	-3.02370700	-3.04063900
C	-6.44722800	-1.74783200	-1.34918400
H	-7.39744300	-1.95495100	-0.86636300
C	-5.53648000	-0.88508200	-0.74256900
H	-5.77643800	-0.41552800	0.20607400
H	0.76387500	1.15124000	2.27413300
H	1.00053300	0.41352900	3.86592900
H	2.17556500	0.16265000	2.56897000
H	-1.27931300	-4.92872200	3.96229600
H	-1.00231000	-3.62809600	5.13574800
H	-2.52297300	-3.70738800	4.23666700
H	-1.22751300	-2.34165500	-1.31749800
H	0.32516100	-3.16453600	-1.26225000
H	-1.14200500	-4.02459200	-0.76475200
H	-2.53487200	2.83275000	-2.67388500
H	-1.79843700	1.32637100	-3.25060400
H	-3.57469300	1.47700600	-3.17058900

H	-4.15759100	-0.37489600	2.15609400
H	-3.51294000	-1.69203500	1.14093600
H	-2.44028400	-0.83344500	2.25399500
C	3.37319000	1.55331700	0.43083200
C	3.18077000	0.91343100	-2.39144500
C	5.54917000	0.06941300	-0.90384000
C	3.55884000	-1.92458400	-1.77240500
O	6.69590500	0.21957600	-1.02352800
O	3.51858200	-2.90609200	-2.38285300
O	3.21120800	2.50991400	1.05899800
O	2.96996700	1.50982600	-3.35706200
C	0.11240400	1.10337700	-0.33363300
O	0.36124100	2.18878600	-0.93159100
C	3.85189500	-1.28397600	1.03691200
O	3.98383400	-1.90564100	2.00341000
Mo	3.56989800	-0.17857000	-0.67508700

Mo-int2

123

C	5.50190500	-1.68976200	1.55337400
N	4.96987300	-0.96334700	0.41486100
C	-5.38313900	-0.60887600	2.27258900
Si	3.48527400	-1.03855400	-0.72661800
C	5.63510900	-0.19524100	-0.45296900
N	-4.96334500	-0.46578700	0.89108400
Si	-3.68803800	-1.18360900	-0.27824500
N	4.84650500	-0.04775100	-1.52706100
C	-5.67053500	0.02241300	-0.13076000
C	4.92450800	1.01058200	-2.51966600
N	-5.04876100	-0.35960500	-1.25485300
C	-5.20462700	0.24377800	-2.56707500
C	6.97042900	0.39912700	-0.25994900
C	7.99584500	0.14915000	-1.18295500
C	7.20200800	1.23303600	0.84373600
C	9.25320900	0.72172500	-0.99254500

H	7.80642400	-0.50043800	-2.03273700
C	8.45877100	1.81014900	1.02172100
H	6.38956300	1.44985700	1.52988500
C	9.48486800	1.55176000	0.10822400
H	10.05046900	0.52133500	-1.70229400
H	8.63568800	2.46408800	1.87059700
H	10.46390400	1.99992100	0.25194300
C	-6.88521900	0.85490400	-0.04402200
C	-8.08276000	0.42649000	-0.63325900
C	-6.82508400	2.09098200	0.61577500
C	-9.22080700	1.22847800	-0.54895200
H	-8.11927900	-0.53405300	-1.13927500
C	-7.96448200	2.89180600	0.68698300
H	-5.88221300	2.42894800	1.03409000
C	-9.16225800	2.45994300	0.11008400
H	-10.15142300	0.89329100	-0.99746900
H	-7.91608700	3.85349300	1.18937000
H	-10.04915700	3.08423900	0.17086000
H	4.45337700	0.66459400	-3.44293900
H	4.40238700	1.90871200	-2.17095600
H	5.96682800	1.26472700	-2.73090000
H	4.67460200	-1.93852900	2.22151400
H	5.99025800	-2.62092900	1.23807000
H	6.22911400	-1.08285000	2.09964300
H	-4.49629100	-0.78707400	2.88319500
H	-5.88623100	0.29621000	2.62464300
H	-6.06659200	-1.45932000	2.39426400
H	-6.26153100	0.41397600	-2.79167500
H	-4.66869400	1.19771800	-2.62657800
H	-4.79332100	-0.43730800	-3.31548400
P	1.70603800	0.12413100	-0.36902500
P	-1.76324000	-0.24994200	-0.55512100
C	-1.44828600	-1.72894900	2.50191000
C	-0.09542200	-2.83369600	0.21268700

C	0.10543700	0.65103200	2.34577600
O	-0.17515700	-3.83093200	-0.38761700
O	-2.32577500	-2.01946200	3.22788400
O	0.20828000	1.63292700	2.95323700
C	-2.33528400	1.53380600	-0.67127100
C	-2.25936700	2.14785900	-1.95433600
C	-2.84713900	2.29720400	0.40799900
C	-2.71089600	3.46057100	-2.12927100
C	-3.29812200	3.60670600	0.17917300
C	-3.24964700	4.20707900	-1.07709300
H	-2.64301600	3.91271700	-3.11735600
H	-3.69037700	4.17469400	1.02248300
C	2.52614900	1.73688300	0.15839300
C	2.38043600	2.84549000	-0.72473200
C	3.29902700	1.92093100	1.33293600
C	3.05205800	4.04386400	-0.46392700
C	3.96126400	3.14092000	1.54622300
C	3.86618900	4.21051100	0.66040000
H	2.93690300	4.87271500	-1.16047900
H	4.56125600	3.25333700	2.44891300
C	-1.71151400	1.42088800	-3.16661600
C	-3.78021700	5.60349900	-1.30194700
C	-2.92363900	1.80841800	1.83948200
C	1.49164500	2.78835900	-1.94894800
C	3.42739500	0.89774500	2.43952000
C	4.57751600	5.51756100	0.92007100
H	1.53153600	3.73406700	-2.50080800
H	1.77410100	1.97774300	-2.62816900
H	0.44846900	2.61186700	-1.66557500
H	5.40699000	5.39035600	1.62417400
H	4.97976400	5.94578300	-0.00555000
H	3.89426100	6.26269300	1.34920200
H	4.46185000	0.82292200	2.79452900
H	2.81220400	1.19885900	3.29578600

H	3.09805500	-0.09029500	2.13844100
H	-1.64081300	2.10185400	-4.02236400
H	-0.71912500	1.00179700	-2.97561500
H	-2.35003400	0.57769000	-3.45353300
H	-3.21308100	6.12811200	-2.07904700
H	-4.82973300	5.58098300	-1.62658000
H	-3.73562900	6.20259700	-0.38585200
H	-3.92338900	1.97537600	2.25781300
H	-2.68736000	0.75124900	1.93572400
H	-2.21624700	2.36353700	2.46507900
Si	3.46925300	-3.22903800	-1.58592600
Si	-4.04843500	-3.51071500	-0.36540700
C	-5.94109100	-3.69798000	-0.38296600
C	-3.34109500	-4.41439800	1.13698600
C	-3.30213700	-4.19344300	-1.96610400
C	2.13008200	-3.44906300	-2.90232400
C	5.19216100	-3.46786000	-2.35465300
C	3.25794300	-4.45106600	-0.15371100
H	-3.67666300	-5.45959900	1.12987800
H	-2.24774100	-4.40664700	1.11954500
H	-3.65987600	-3.95326600	2.07675500
H	-6.38263800	-3.31780500	0.54629000
H	-6.38900900	-3.14637800	-1.21750600
H	-6.22468700	-4.75346800	-0.48039800
H	-3.51858900	-5.26522400	-2.06255300
H	-3.70548100	-3.68525600	-2.84969900
H	-2.21475200	-4.06502600	-1.96290300
H	2.26002700	-2.73391400	-3.72269800
H	2.17555200	-4.46169600	-3.32345100
H	1.13298800	-3.30491800	-2.47637900
H	5.35440500	-2.77884900	-3.19124300
H	5.98290800	-3.28832100	-1.61585600
H	5.30995600	-4.49263800	-2.72897500
H	2.29587500	-4.31964800	0.34850200

H	3.30931100	-5.48236500	-0.52588300
H	4.04880700	-4.32261600	0.59432200
C	1.41542100	-1.92210900	2.40352800
O	2.27990200	-2.42683500	3.01784300
Mo	-0.00954800	-1.11707200	1.29900300

Mo-int3

127

C	-5.47651800	-1.69762100	-1.67729100
N	-4.88142900	-1.02123400	-0.53943900
C	5.48250900	-0.20659800	-2.40811900
Si	-3.32851800	-1.07265800	0.51054200
C	-5.49723900	-0.27720100	0.37721600
N	4.98532300	-0.21961100	-1.04610100
Si	3.65661400	-1.08303900	-0.04942100
N	-4.65650300	-0.13850100	1.41422500
C	5.63311400	0.13365500	0.06617100
C	-4.70373200	0.89343600	2.43743600
N	4.94453400	-0.37759300	1.09554900
C	5.00680600	0.06686600	2.47792700
C	-6.84793400	0.29526800	0.28148000
C	-7.75979500	0.07586200	1.32688700
C	-7.20415500	1.08468800	-0.82138600
C	-9.04132600	0.62076400	1.24778600
H	-7.44722400	-0.51261500	2.19598700
C	-8.48526200	1.63020100	-0.88925700
H	-6.47356000	1.28131400	-1.60009600
C	-9.40519300	1.39317500	0.13915500
H	-9.75142200	0.44893100	2.05126800
H	-8.76455100	2.24296100	-1.74137400
H	-10.40243500	1.81997300	0.08118800
C	6.84425200	0.96872000	0.15376200
C	7.99746000	0.48298900	0.78561600
C	6.82052200	2.26812400	-0.37367400
C	9.12901600	1.29300300	0.87658100

H	8.00498000	-0.52590300	1.18844900
C	7.95253400	3.07555100	-0.26986500
H	5.90778500	2.64595700	-0.82521100
C	9.10693000	2.58800200	0.35006800
H	10.02568800	0.91521300	1.35908100
H	7.93175000	4.08554900	-0.66841800
H	9.98819500	3.21842100	0.42671400
H	-4.17965800	0.52812400	3.32350200
H	-4.20965800	1.80732300	2.09257600
H	-5.73808400	1.12438300	2.70683200
H	-4.68057900	-1.92790300	-2.38945200
H	-5.96850300	-2.63250800	-1.37857900
H	-6.21442100	-1.05721300	-2.16832800
H	4.62659200	-0.30635200	-3.08119100
H	6.00280200	0.73023000	-2.62754700
H	6.17007800	-1.04270800	-2.59042800
H	6.04483400	0.14127100	2.81439700
H	4.52408100	1.04250500	2.59538700
H	4.48014300	-0.66471100	3.09492100
P	-1.63125900	0.18800300	0.09734800
P	1.71463000	-0.17761100	0.20214400
C	1.49380700	-1.33602500	-2.96875000
C	0.14570600	-2.72868600	-0.84711600
C	-0.11850200	0.97385400	-2.54787300
O	0.07250400	-3.77766300	-0.34460000
O	2.37775200	-1.47334600	-3.72968100
O	-0.33561500	2.07197100	-2.84829500
C	2.19126700	1.61236900	0.49409900
C	1.97634000	2.14588200	1.80264700
C	2.69228600	2.48358700	-0.51119800
C	2.31896300	3.47382900	2.07390400
C	3.04207100	3.80048100	-0.17666300
C	2.87508300	4.31467900	1.10497100
H	2.14708400	3.86448500	3.07532900

H	3.43147800	4.44959700	-0.96045300
C	-2.55861600	1.75135500	-0.36181700
C	-2.42753400	2.89477200	0.48013000
C	-3.40282700	1.84471900	-1.49067300
C	-3.18441300	4.04285700	0.21289500
C	-4.15939500	3.00648700	-1.70408000
C	-4.07556200	4.11434100	-0.86341200
H	-3.08365700	4.90438000	0.87099800
H	-4.82116300	3.04462800	-2.56891700
C	1.38020700	1.33637600	2.93712400
C	3.28423900	5.72951000	1.44120500
C	2.82997200	2.12010400	-1.97571400
C	-1.47213200	2.94711100	1.65341100
C	-3.48602800	0.76333500	-2.54696400
C	-4.88151500	5.36463100	-1.12436800
H	-1.51856400	3.92802200	2.14176300
H	-1.69287800	2.18517900	2.40569300
H	-0.43646800	2.78624500	1.33236300
H	-5.74296400	5.15931000	-1.76913800
H	-5.25205800	5.80488600	-0.19134100
H	-4.27365300	6.13130700	-1.62332200
H	-4.50996700	0.64421900	-2.92169500
H	-2.85444900	1.03372500	-3.40442300
H	-3.14168400	-0.20216400	-2.18755400
H	1.14235600	1.98515900	3.78698500
H	0.47329100	0.79784300	2.64130500
H	2.08402100	0.57359000	3.28706200
H	2.63061700	6.16596500	2.20471000
H	4.30916400	5.76158900	1.83585500
H	3.25478600	6.37721200	0.55819700
H	3.81925700	2.39987300	-2.36001700
H	2.67562400	1.06111700	-2.16415900
H	2.08955300	2.66699700	-2.56792700
Si	-3.21159100	-3.28845600	1.29749600

Si	4.10061800	-3.39127200	-0.28306800
C	5.99841800	-3.50175100	-0.23409900
C	3.46736600	-4.06459500	-1.93280200
C	3.38725700	-4.39568000	1.15197500
C	-1.76008900	-3.62015900	2.46047500
C	-4.84655600	-3.55480500	2.23341200
C	-3.14675900	-4.44391400	-0.20239000
H	3.85224200	-5.08063900	-2.08829400
H	2.37298200	-4.10596500	-1.94196700
H	3.78093800	-3.44210800	-2.77741800
H	6.45529000	-2.95962100	-1.07064200
H	6.39668000	-3.08122500	0.69634100
H	6.31897400	-4.54941400	-0.29863800
H	3.69481900	-5.44609900	1.06392500
H	3.72108000	-4.01181600	2.12422800
H	2.29687700	-4.36043400	1.14264600
H	-1.75227800	-2.96437500	3.33472500
H	-1.84074200	-4.65122400	2.83392800
H	-0.79778100	-3.51886200	1.96222900
H	-4.92661800	-2.88308300	3.09534800
H	-5.71127300	-3.36951500	1.57176100
H	-4.92153300	-4.58716300	2.59760700
H	-2.24375900	-4.28342300	-0.79228400
H	-3.14721600	-5.49036000	0.12994600
H	-4.01195900	-4.29624900	-0.85832800
C	-1.38024700	-1.61158600	-2.91262900
O	-2.39137700	-1.90399900	-3.43226400
Mo	0.02622700	-0.90611000	-1.71980900
C	-1.67215800	-0.35325600	3.97398700
C	2.07585900	-2.20708100	3.36789100
O	-0.66807500	-0.18732000	4.46252800
O	1.05611000	-2.66193000	3.54203000

Mo-ts1

127

N	4.58009900	0.71155900	-0.55048600
O	1.49603100	-2.79030000	-2.39581000
Si	3.17907800	-0.68959600	-0.75832500
C	4.30549400	1.15681800	-1.76196500
N	-4.87817200	-0.76804500	-0.07214000
Si	-3.25945500	-1.03345400	-0.92321100
N	3.30810900	0.40009600	-2.26482100
C	1.64896100	-1.80435300	-1.80057200
C	-5.16903300	0.25794900	-0.88780200
N	-4.18000900	0.30438400	-1.79414000
P	1.69298600	-0.06690300	0.75653000
P	-1.42574600	-0.19473100	-0.03684100
C	-1.92066100	-2.52442200	2.28750900
C	0.01695900	-3.25435000	0.39493600
C	-0.36029900	-0.24885100	3.14550100
O	0.13512100	-4.21826400	-0.25082300
O	-2.96949800	-2.96259200	2.58931200
O	-0.41500600	0.54925900	3.98317100
C	-2.03261400	1.53867300	0.36516400
C	-1.58302600	2.63677600	-0.41804600
C	-2.94385800	1.79300000	1.42337400
C	-2.09342000	3.91776900	-0.17500900
C	-3.42703700	3.09418100	1.62151500
C	-3.02884800	4.17018500	0.83120400
H	-1.74305600	4.74425100	-0.79027500
H	-4.14269500	3.26271400	2.42467900
Si	4.63796300	-2.55091300	-1.03540800
Si	-3.34687900	-3.14669300	-1.96545700
C	0.84672200	-2.79645400	2.98206600
O	1.44302500	-3.45233600	3.74206800
C	-0.14590800	-0.62501100	-2.06099700
O	-0.55576400	-0.29274200	-3.10513300
C	2.65302100	0.69987100	2.15677800
C	2.35030500	2.04708700	2.49261500

C	3.59627700	0.00548900	2.95310100
C	2.99993000	2.65841300	3.57104500
C	4.22020000	0.65575300	4.02538800
C	3.94034100	1.98274600	4.35307000
H	2.76034400	3.69339500	3.81020100
H	4.94733100	0.10541200	4.62077200
C	4.00386100	-1.41814300	2.66260200
C	1.33313600	2.85983200	1.72028800
C	4.60017200	2.65539100	5.53421200
C	-3.43167100	0.75516800	2.41072600
C	-3.56747000	5.55951600	1.07813500
C	-0.55064000	2.49464400	-1.51363600
C	3.91855800	-4.05830600	-0.14103600
C	6.38587900	-2.15886300	-0.39803300
C	4.81495200	-2.92595900	-2.89284600
C	-3.42396700	-4.45083100	-0.59764800
C	-1.87847400	-3.44364800	-3.11408600
C	-4.97485900	-3.12289700	-2.94234900
C	-3.92348300	1.31060500	-2.80589000
C	-5.66807500	-1.32727800	1.00807400
C	5.40316000	1.36957400	0.44711700
C	2.91999600	0.29466300	-3.66101700
C	4.93775000	2.29684300	-2.46298700
C	4.14074500	3.35241500	-2.92961400
C	6.32523800	2.32891000	-2.66216000
C	4.73063000	4.43486500	-3.58213200
H	3.06711000	3.32249000	-2.76658800
C	6.90940600	3.40995800	-3.32231100
H	6.93662800	1.50262200	-2.31084700
C	6.11394700	4.46412900	-3.78003900
H	4.11157300	5.25450900	-3.93535700
H	7.98385800	3.42882900	-3.48008500
H	6.57136000	5.30661300	-4.29102600
C	-6.29963100	1.19187100	-0.76356800

C	-6.04346200	2.57253300	-0.73001700
C	-7.61330900	0.71610500	-0.63632600
C	-7.10044300	3.46682200	-0.56949800
H	-5.02355400	2.93708500	-0.78527900
C	-8.66607000	1.61880600	-0.49182600
H	-7.80849600	-0.35140300	-0.66692300
C	-8.41044900	2.99264600	-0.45587500
H	-6.89911000	4.53331100	-0.52907100
H	-9.68368000	1.25019200	-0.40409700
H	-9.23214500	3.69279800	-0.33541000
H	7.05712000	-3.00091900	-0.61157000
H	6.78593200	-1.27071500	-0.90086100
H	6.41673300	-1.97553900	0.68070100
H	5.49540100	-3.77383100	-3.04498900
H	3.85274900	-3.18167200	-3.34763100
H	5.23065000	-2.06492600	-3.43048100
H	3.85455900	-3.88648000	0.93887500
H	2.90907600	-4.28568100	-0.50021600
H	4.54595500	-4.94319000	-0.30822100
H	-2.47468400	-4.51131600	-0.05848200
H	-4.21009300	-4.23510500	0.13399900
H	-3.62872100	-5.43509000	-1.03804300
H	-2.02585100	-4.38881700	-3.65227100
H	-1.76537700	-2.64408700	-3.85336800
H	-0.94276900	-3.52243700	-2.55269300
H	-5.82960900	-2.92654700	-2.28417900
H	-4.96619100	-2.35271600	-3.72193400
H	-5.14056200	-4.09339000	-3.42677900
H	1.58924100	2.92472700	0.65741200
H	0.33507200	2.41192900	1.76958000
H	1.26291600	3.87689200	2.12219700
H	4.73866600	3.72863300	5.35965200
H	3.98936600	2.55143300	6.44129300
H	5.58042200	2.21651400	5.75139100

H	4.77966000	-1.44998100	1.88892200
H	4.41461000	-1.90395100	3.55397200
H	3.16451500	-2.01143700	2.29913200
H	6.11787400	2.05660600	-0.01584300
H	4.78223600	1.92446500	1.15680400
H	5.95845700	0.61334800	1.00789800
H	3.38674500	1.08878600	-4.24953300
H	3.22826400	-0.67002600	-4.08194200
H	1.83464100	0.38152900	-3.76146200
H	-4.99604000	-1.82259600	1.71136000
H	-6.21961300	-0.54153300	1.53264900
H	-6.38414600	-2.06750800	0.63045700
H	-3.30193900	0.86542000	-3.58631100
H	-4.86222300	1.65688000	-3.24888300
H	-3.38869800	2.16896500	-2.38523600
H	-2.94669800	0.90905400	3.38106000
H	-4.51136800	0.85217900	2.56727600
H	-3.21631100	-0.26485300	2.10646100
H	-3.43681400	6.20345500	0.20189200
H	-4.63464700	5.53375400	1.32816800
H	-3.05118100	6.04037000	1.91931400
H	-0.95369300	1.98339100	-2.39310800
H	-0.19949500	3.48134700	-1.83475400
H	0.31438600	1.91758700	-1.16946700
Mo	-0.22327500	-1.71038100	1.69860500

Mo-int4

127

C	2.91554400	1.16346100	-2.93522100
N	3.31229300	0.17460600	-1.93145600
O	-1.84289200	-1.18121300	-2.04416900
C	-5.59889000	-0.63515900	1.37646600
Si	2.25086100	-1.02838500	-1.11672200
C	4.62587600	0.08879400	-1.51105200
N	-4.74671800	-0.32642100	0.24173300

Si	-3.21755000	-1.07693100	-0.81584900
N	4.90377600	-0.82927200	-0.65001400
C	-0.78908800	-0.69329600	-1.37844300
C	-5.09534900	0.37668300	-0.81934800
C	6.25288400	-1.00178400	-0.14632600
N	-4.21047500	0.08686100	-1.80132600
C	-3.94944500	0.88405200	-2.99080200
C	5.61633800	1.07578200	-2.04848100
C	5.87124500	2.24897400	-1.32672500
C	6.31910700	0.82078000	-3.23266900
C	6.81696500	3.16343600	-1.79374400
H	5.33166200	2.43669300	-0.40295000
C	7.26623000	1.73674200	-3.69519500
H	6.12221900	-0.09360300	-3.78642100
C	7.51494900	2.90999000	-2.97769100
H	7.01001000	4.07287900	-1.23067800
H	7.80954900	1.53394400	-4.61415000
H	8.25186200	3.62225700	-3.33892300
C	-6.22845900	1.31360100	-0.95277600
C	-7.16378100	1.15223200	-1.98489700
C	-6.35502500	2.38064500	-0.05129000
C	-8.22502300	2.04878100	-2.10635000
H	-7.06526600	0.31889400	-2.67438500
C	-7.41089900	3.28139200	-0.18609700
H	-5.60900100	2.51477200	0.72475200
C	-8.34760000	3.11458600	-1.21007700
H	-8.95422300	1.91694800	-2.90031200
H	-7.50119200	4.11336000	0.50635500
H	-9.17163800	3.81517400	-1.31093200
H	6.41394000	-2.06196200	0.07906900
H	7.03635400	-0.67654700	-0.84433100
H	6.38568000	-0.44287700	0.78984400
H	2.90393000	2.18205700	-2.52570400
H	3.60203000	1.15305400	-3.78723600

H	1.92432300	0.91339300	-3.30778100
H	-4.97375600	-0.91542800	2.22678800
H	-6.21554000	0.22222200	1.66209000
H	-6.26424100	-1.47885200	1.15135600
H	-3.17565200	0.37523600	-3.56721300
H	-4.84715300	0.97867000	-3.60845600
H	-3.59363900	1.88653400	-2.72573900
P	1.87874000	-0.37496600	0.97972900
P	-1.40607000	-0.10901300	0.23666600
C	-1.86202300	-1.54794100	3.12447400
C	0.87038700	-1.85069000	3.84644600
C	-0.08417300	-2.89014500	1.31547900
O	1.43128800	-2.29025100	4.76252500
O	-2.89571500	-1.80441200	3.59373800
O	-0.08084900	-3.93507200	0.81481700
C	-1.83553900	1.71411600	0.11859900
C	-1.47745300	2.55257400	-0.97233400
C	-2.65805800	2.24766700	1.14630100
C	-1.95637600	3.86962000	-1.00369200
C	-3.09853600	3.57342300	1.06216000
C	-2.77380200	4.40316500	-0.00821700
H	-1.67621700	4.49843500	-1.84569000
H	-3.72591400	3.96094700	1.86313000
C	3.13518800	0.82268200	1.57650200
C	3.00594900	2.20471900	1.31405600
C	4.22087900	0.35242700	2.35357700
C	3.98191100	3.08363900	1.80080100
C	5.17524900	1.26694000	2.80909200
C	5.08259800	2.63512600	2.53467700
H	3.87916500	4.14715100	1.59344400
H	6.01457200	0.89925800	3.39642300
C	-0.59619100	2.14012100	-2.12997400
C	-3.31933500	5.80715100	-0.10009800
C	-3.11003200	1.47709300	2.36705600

C	1.85576300	2.76056600	0.50913900
C	4.36685000	-1.10936500	2.70446000
C	6.15732900	3.59267600	2.99176700
H	1.86986900	3.85511500	0.50284300
H	1.91196100	2.42100400	-0.52932900
H	0.88383500	2.44045800	0.89865600
H	6.58620300	3.28707400	3.95240800
H	6.98137300	3.63005100	2.26612600
H	5.76964900	4.61138200	3.09925400
H	3.57267800	-1.43710900	3.38443400
H	4.30793100	-1.73428900	1.80820200
H	5.32710600	-1.30084500	3.19426600
H	0.37369400	1.78247500	-1.78654100
H	-1.03226400	1.34093800	-2.73576700
H	-0.42022900	2.99593300	-2.78895800
H	-2.72872000	6.42557500	-0.78354200
H	-4.35260400	5.79766600	-0.47248300
H	-3.33236600	6.29645000	0.88015800
H	-4.09453300	1.82821000	2.69336300
H	-3.18910700	0.40956000	2.19107300
H	-2.41918300	1.62183800	3.20333900
Si	2.92299800	-3.23498500	-1.61288800
Si	-3.83509800	-3.34480600	-0.72357500
C	-3.80250300	-3.96290000	1.06707900
C	-2.64140100	-4.35337300	-1.78457100
C	-5.60347300	-3.45100700	-1.41178400
C	3.60820600	-4.08107300	-0.06220900
C	1.42627300	-4.20910600	-2.25585400
C	4.23020400	-3.17106800	-2.98839000
H	-2.95035000	-5.40526900	-1.83038600
H	-2.58696200	-3.96157600	-2.80570600
H	-1.63167500	-4.31363300	-1.36403100
H	-2.78192400	-3.96421200	1.46272200
H	-4.41623300	-3.34905100	1.73386700

H	-4.17948000	-4.99271000	1.11144300
H	-5.95697500	-4.48972800	-1.40128000
H	-6.30435100	-2.85270200	-0.81802700
H	-5.64804900	-3.09041200	-2.44600600
H	2.86533000	-4.07844900	0.74403800
H	3.87539400	-5.12504200	-0.26978500
H	4.50138800	-3.56063900	0.29734000
H	0.70013700	-4.38718700	-1.45762600
H	0.92213400	-3.65730500	-3.05607200
H	1.74224600	-5.18462000	-2.64782500
H	5.12311900	-2.63511500	-2.65353100
H	4.52265100	-4.18506300	-3.29062800
H	3.83090800	-2.65736700	-3.87063500
C	0.51406200	-0.87487700	-1.96389500
O	0.63128600	-1.00969500	-3.20873700
C	0.05159000	0.77026900	3.16722200
O	0.19202700	1.80548800	3.66127900
Mo	-0.09594100	-1.07273200	2.26170800

Mo-int5

127

C	2.17247900	4.48128600	1.33255900
N	1.11248900	3.60362800	0.86636500
O	0.88172800	0.12821000	1.77558600
C	4.19776700	-3.42395000	-0.01154300
Si	-1.08174100	1.80993500	0.89873900
C	0.19820300	4.03260900	0.05954800
N	3.62578500	-2.13752800	0.34210700
Si	2.12294100	-1.32581700	1.26980700
N	-0.85033600	3.17970200	-0.22558900
C	0.25945700	0.40012500	0.68973300
C	4.28218400	-0.98778100	0.41988100
C	-1.74438400	3.44428300	-1.36362100
N	3.47714400	-0.12114600	1.07261900
C	3.65863000	1.31269500	1.23063200

C	0.17855700	5.39249400	-0.56671300
C	1.19938100	5.77989400	-1.44430700
C	-0.85168600	6.29393200	-0.26460600
C	1.18688600	7.05102200	-2.02057200
H	1.99768500	5.07987000	-1.67562100
C	-0.85472500	7.56943200	-0.82940700
H	-1.64320600	5.99133500	0.41469200
C	0.16134000	7.94828000	-1.71158100
H	1.97697700	7.34111200	-2.70770300
H	-1.65208000	8.26563100	-0.58486900
H	0.15343900	8.93934300	-2.15672900
C	5.61700700	-0.68823100	-0.12257200
C	6.57003700	-0.00327000	0.64621500
C	5.92107100	-1.07656100	-1.43708000
C	7.82429600	0.27751100	0.10623100
H	6.33371600	0.29042100	1.66420000
C	7.17132000	-0.77821800	-1.97557700
H	5.16688900	-1.57405200	-2.03755800
C	8.12436000	-0.10641200	-1.20420600
H	8.56579200	0.79677800	0.70592700
H	7.40098200	-1.06951000	-2.99640700
H	9.10020300	0.11988500	-1.62422500
H	-2.59933500	4.06460700	-1.07564800
H	-1.19911300	3.94519300	-2.16716500
H	-2.12619900	2.49545800	-1.74646500
H	3.09899700	4.30048900	0.76873600
H	1.94224300	5.55217100	1.25923400
H	2.38859600	4.25132600	2.38201600
H	3.59547100	-4.21324300	0.44570000
H	4.19475200	-3.58234300	-1.09443400
H	5.22731600	-3.51369500	0.35158500
H	2.68091800	1.79280900	1.28339500
H	4.20706300	1.54385900	2.15121000
H	4.21043200	1.72600900	0.38053200

P	-3.04561300	-0.15297400	-0.69787100
P	0.58221700	-1.09597200	-0.41987400
C	-0.68269800	-4.05606300	0.06990600
C	-3.40119600	-3.28378900	-0.01098500
C	-1.63906200	-1.94342300	1.81460000
O	-4.41399200	-3.83789200	0.09561400
O	-0.12215400	-5.07414500	0.19985100
O	-1.64197700	-1.72779500	2.95659300
C	1.58640100	-0.86062600	-1.96125600
C	1.97399700	0.40243700	-2.46503300
C	2.07958800	-2.03957000	-2.57844600
C	2.88160600	0.46447000	-3.52740000
C	2.98550300	-1.91910600	-3.63963900
C	3.41604900	-0.68086300	-4.11934700
H	3.17594500	1.44220500	-3.90248900
H	3.36217200	-2.82792700	-4.10419500
C	-4.85621700	-0.44032000	-0.92073100
C	-5.36773500	-0.49772200	-2.24106500
C	-5.72963400	-0.68389400	0.16863600
C	-6.72120400	-0.78565500	-2.44545700
C	-7.07720000	-0.96517700	-0.08577800
C	-7.59547400	-1.01926700	-1.38102700
H	-7.10341800	-0.82722400	-3.46404000
H	-7.73977500	-1.15642300	0.75667100
C	1.42100900	1.68967000	-1.91946400
C	4.44370300	-0.57791100	-5.21996100
C	1.66436800	-3.43517000	-2.17180100
C	-4.49141700	-0.26059000	-3.45422100
C	-5.26128500	-0.65440600	1.60341700
C	-9.06126600	-1.29541200	-1.62199800
H	-5.08940900	-0.27105100	-4.37186500
H	-3.97323100	0.70331400	-3.39502400
H	-3.71699000	-1.02920400	-3.55613300
H	-9.47908300	-1.94732900	-0.84670500

H	-9.64615800	-0.36533400	-1.61372000
H	-9.22570700	-1.77371600	-2.59393700
H	-4.35542100	-1.25235600	1.74512900
H	-5.01725800	0.36934000	1.90330400
H	-6.03415300	-1.04085700	2.27572400
H	0.32923300	1.69941300	-1.96331400
H	1.70201200	1.86277800	-0.87592100
H	1.78024600	2.54839000	-2.49544100
H	4.20173700	0.22939900	-5.91982500
H	5.43618400	-0.35729100	-4.80370400
H	4.52223600	-1.51055000	-5.78759300
H	2.32312700	-4.17885200	-2.62989200
H	1.68754100	-3.59636300	-1.09275300
H	0.64433500	-3.65268200	-2.49847000
Si	-1.17472700	2.53937900	3.13942600
Si	2.10713000	-2.55275000	3.28400900
C	3.93285900	-2.94552400	3.62981700
C	1.11373200	-4.14981600	3.12111700
C	1.40158900	-1.46272600	4.65561000
C	-2.52121400	1.52637900	4.00619700
C	0.44908800	2.35627300	4.09576000
C	-1.68906900	4.36605600	3.07774500
H	1.23784800	-4.75040800	4.03129000
H	0.04749900	-3.93812400	2.99809700
H	1.42815300	-4.75596200	2.26615400
H	4.33667500	-3.65860000	2.90292100
H	4.55286400	-2.04176500	3.59631800
H	4.03895600	-3.38753400	4.62842300
H	1.39721300	-2.01040400	5.60652300
H	1.99669900	-0.55309900	4.79147400
H	0.37323700	-1.16918200	4.42505300
H	-2.26520200	0.46160500	4.02652300
H	-2.65675600	1.86587400	5.04116300
H	-3.47217000	1.63217700	3.47480300

H	0.70262100	1.30049200	4.21952900
H	1.27453600	2.84209200	3.56867900
H	0.35595900	2.80888100	5.09148200
H	-2.62085700	4.47837000	2.51205900
H	-1.85425400	4.75787500	4.08955800
H	-0.91740500	4.97850300	2.59864200
C	-2.85605400	1.20229600	0.44105900
O	-3.71447900	2.00090100	0.86695000
C	-1.70796800	-2.60751800	-2.23008500
O	-1.74712800	-2.76157100	-3.37684100
Mo	-1.63595800	-2.33322600	-0.18966900

Mo-ts3

C	0.73268100	4.23462100	1.74013000
N	-0.21639500	3.52946400	0.91650500
O	1.23582500	1.11780200	1.52714100
Si	-1.38109500	1.78289700	0.79454000
C	-0.89096600	4.00168600	-0.10875100
N	3.96149700	-1.55107200	1.02394000
Si	2.45325800	-0.43287000	1.47199100
N	-1.69824000	3.01569100	-0.56233100
C	0.33750300	0.85778000	0.66005900
C	4.62643500	-0.48549000	0.59575900
C	-2.67403200	3.10699700	-1.63251700
N	3.82027300	0.57735300	0.82056000
H	-2.97915900	4.14553100	-1.79250400
H	-2.27633700	2.71029800	-2.57593400
H	-3.55818700	2.52413300	-1.36507100
H	1.23812100	5.04000900	1.19245300
H	0.25216700	4.67497100	2.62518600
H	1.48094500	3.51851100	2.09327100
P	-2.66766600	-0.81435200	-0.94828200
P	0.87349600	-0.79130600	-0.16620900
C	0.43503400	-3.88618500	0.68080000
C	-2.38175500	-3.93445100	0.16176700

C	-1.27188000	-1.98514000	1.92932500
O	-3.23967900	-4.71009700	0.25357500
O	1.25823500	-4.61954600	1.08011400
O	-1.49298300	-1.68285800	3.02923200
C	1.86922400	-0.42990700	-1.71235300
C	1.85095900	0.81271700	-2.38993500
C	2.72733300	-1.45709200	-2.18747800
C	2.73374100	1.02742200	-3.45711600
C	3.58857700	-1.18790000	-3.25651400
C	3.62540700	0.04970700	-3.89806400
H	2.71176400	1.99108000	-3.96133200
H	4.25461600	-1.97959900	-3.59377100
C	-4.35328700	-1.54298300	-1.19936400
C	-4.71745800	-1.82705400	-2.54161100
C	-5.23804800	-1.91104800	-0.15663400
C	-5.94879700	-2.43043300	-2.81267700
C	-6.46152200	-2.51341900	-0.47860800
C	-6.84239200	-2.77501900	-1.79483600
H	-6.21659700	-2.64065500	-3.84682800
H	-7.13192000	-2.79657300	0.33125100
C	0.89527900	1.92537700	-2.04472300
C	2.74863700	-2.87106700	-1.65296700
C	-3.80123400	-1.51215800	-3.70847600
C	-4.92534500	-1.69340800	1.30377700
C	-8.18307100	-3.39492200	-2.11229900
H	-4.28465400	-1.76657700	-4.65780500
H	-3.52895700	-0.45084900	-3.73898000
H	-2.86270700	-2.07731700	-3.65411700
H	-8.54089600	-4.02110100	-1.28761700
H	-8.94456200	-2.62284800	-2.28899000
H	-8.13534500	-4.01494900	-3.01459100
H	-3.87341900	-1.88569000	1.52590300
H	-5.12154200	-0.65685200	1.59207000
H	-5.53347500	-2.35020800	1.93432900

H	-0.13387000	1.56386800	-1.97657300
H	1.13631400	2.40788700	-1.09114400
H	0.92129500	2.70537200	-2.81205800
H	3.75851600	-3.28808500	-1.71381900
H	2.41396300	-2.95509000	-0.62426800
H	2.09052100	-3.50882500	-2.25323000
Si	-3.56624000	2.24954600	2.18280000
Si	2.25544200	-0.91091400	3.77105500
C	-3.37761300	1.08644800	3.66841500
C	-2.93717500	4.00666900	2.68462000
C	-5.42348200	2.66079000	1.96510700
H	-3.44819200	0.03773300	3.36605500
H	-2.40415500	1.22597600	4.15218300
H	-4.16428100	1.28097100	4.40971300
H	-1.94991400	3.95522600	3.15529600
H	-2.86114700	4.66982500	1.81415600
H	-3.64811600	4.46620600	3.38564300
H	-6.02909900	1.75980900	1.81726100
H	-5.80438300	3.19852000	2.84299600
H	-5.58290200	3.30697300	1.09123000
C	-2.72842500	0.51718300	0.15878700
O	-3.80653800	1.05132800	0.66888200
C	-0.61304100	-3.13725100	-1.95016800
O	-0.47016900	-3.44741300	-3.05679000
C	4.59083500	0.30415200	-5.03039900
H	4.55233900	1.34466800	-5.36771100
H	5.62108700	0.08291200	-4.72439700
H	4.36865800	-0.33695900	-5.89234700
C	5.95878700	-0.44746100	-0.03488600
C	7.05438400	-1.08608400	0.56522300
C	6.11931200	0.20914200	-1.26597100
C	8.30182300	-1.05921600	-0.05787600
H	6.93468000	-1.58406900	1.52203900
C	7.36602100	0.21956800	-1.88920600

H	5.26650600	0.67499500	-1.74661000
C	8.45806900	-0.41046900	-1.28571300
H	9.14982200	-1.54603000	0.41438300
H	7.48326100	0.71955600	-2.84627200
H	9.42931900	-0.39684600	-1.77179200
C	4.45150700	-2.90441300	1.19341200
C	3.99481800	1.94994000	0.38634400
H	3.60556400	-3.59414200	1.16333800
H	5.15507900	-3.17240900	0.39887000
H	4.95471900	-3.02461300	2.16164600
H	3.43373500	2.60199600	1.05813800
H	5.05163300	2.23046000	0.41945800
H	3.62091300	2.09914300	-0.63372400
C	3.98543800	-0.58258400	4.48572700
C	0.97071900	0.22444900	4.56411300
C	1.80749800	-2.73496300	3.99385900
H	3.99861000	-0.79102400	5.56289400
H	4.73827300	-1.22322300	4.01105400
H	4.29123200	0.45990900	4.34049300
H	-0.02680300	0.02646400	4.15916900
H	0.93490600	0.06317500	5.64889100
H	1.20586400	1.27802600	4.37891300
H	0.80987300	-2.95093400	3.60147300
H	2.51528900	-3.39736100	3.48456400
H	1.81283400	-2.98940700	5.06143900
C	-0.81918900	5.36764200	-0.67365700
C	-0.92389100	6.48551600	0.16903900
C	-0.63149600	5.55928400	-2.05186400
C	-0.84682200	7.77333400	-0.36112700
H	-1.08595100	6.34115400	1.23245700
C	-0.54409100	6.84833000	-2.57629900
H	-0.54756800	4.69733700	-2.70660300
C	-0.65332200	7.95745000	-1.73274000
H	-0.94020500	8.63262100	0.29684100

H	-0.39355600	6.98704900	-3.64318400
H	-0.59018600	8.96126500	-2.14334100
Mo	-0.89377400	-2.58352400	0.01416500

Mo-int6

127

C	-3.20379100	2.77191900	1.71027100
N	-2.39674100	2.68366600	0.50899600
O	1.31350500	1.86888400	-0.95990500
C	5.07515600	0.09194000	2.11751200
Si	-1.40481100	1.37339500	-0.47501800
C	-2.40822700	3.47978500	-0.55825900
N	4.33566400	0.46628400	0.92711900
Si	2.56569700	1.35364700	0.18585100
N	-1.80164600	2.79862400	-1.55453600
C	0.28552400	0.95971800	-0.60165800
C	4.86165800	0.69960500	-0.25015500
C	-1.13514700	3.33991300	-2.72943500
N	3.94300200	1.34791200	-1.00926500
C	3.93743700	1.38490300	-2.46488700
C	-2.95761400	4.83886800	-0.63811100
C	-2.76588600	5.72875100	0.43230400
C	-3.66629800	5.25736500	-1.77684100
C	-3.27842100	7.02247900	0.36094300
H	-2.19455900	5.40895500	1.29816300
C	-4.18780000	6.54821600	-1.83504500
H	-3.82192400	4.56347500	-2.59641100
C	-3.99316000	7.43182500	-0.76891800
H	-3.11940700	7.71143300	1.18519000
H	-4.74517100	6.86540700	-2.71148500
H	-4.39654000	8.43914500	-0.81938000
C	6.21690300	0.34072200	-0.72758100
C	7.09265900	1.33551200	-1.18418900
C	6.61558900	-1.00319500	-0.74438700
C	8.36446700	0.98804600	-1.63994000

H	6.77625400	2.37471200	-1.16972000
C	7.88436300	-1.34662900	-1.21130800
H	5.91877900	-1.76893800	-0.42024200
C	8.76078000	-0.35246000	-1.65500700
H	9.04441800	1.76171700	-1.98512900
H	8.18707500	-2.38973000	-1.23123900
H	9.74977700	-0.62168700	-2.01527500
H	-1.58436000	2.93715100	-3.64373600
H	-1.21367000	4.43042700	-2.75126300
H	-0.07825100	3.05846700	-2.69575100
H	-2.59668600	3.08141000	2.56604700
H	-4.03182500	3.47612900	1.58236200
H	-3.61138400	1.77975300	1.93279200
H	4.39297100	-0.38296700	2.82680500
H	5.88751500	-0.60783800	1.89282600
H	5.51082400	0.97512400	2.60468000
H	3.07308000	1.97189900	-2.77724400
H	4.84926600	1.85210600	-2.85101500
H	3.84471100	0.37851600	-2.89140300
P	-2.25064400	-1.36996500	0.61379200
P	1.11429000	-0.44297000	0.20213100
C	1.40941700	-1.56853900	3.30511800
C	-1.31916000	-2.19645700	3.71828300
C	-0.58124400	0.41546100	2.89622600
O	-1.90823900	-2.62084000	4.63209600
O	2.38431300	-1.59072000	3.94527500
O	-0.78543000	1.46433300	3.35203900
C	1.95806500	-1.52517000	-1.10432100
C	1.70927600	-1.45385600	-2.50122000
C	2.96692800	-2.41717600	-0.64664100
C	2.47530400	-2.24037500	-3.37599100
C	3.70367000	-3.17348500	-1.56420500
C	3.48465100	-3.09591600	-2.93901200
H	2.27218800	-2.17020200	-4.44300200

H	4.47292700	-3.84567700	-1.18663700
C	-3.27195200	-2.80674500	0.09697200
C	-3.00250300	-3.48236600	-1.11670600
C	-4.31936100	-3.25232000	0.93684200
C	-3.81667700	-4.55993100	-1.48398900
C	-5.10842400	-4.33067200	0.52326800
C	-4.88101100	-4.99156600	-0.68747000
H	-3.60731000	-5.08067200	-2.41633000
H	-5.91997800	-4.66530400	1.16640700
C	0.67339300	-0.55843300	-3.14717700
C	4.32792900	-3.88895900	-3.90854200
C	3.29288900	-2.62800700	0.81560700
C	-1.87006000	-3.07665400	-2.03131800
C	-4.60343900	-2.61012300	2.27777500
C	-5.77100700	-6.12880000	-1.13030500
H	-1.69705000	-3.83735300	-2.79882600
H	-2.10428900	-2.13337900	-2.53567400
H	-0.93010500	-2.93188000	-1.48754000
H	-6.17692800	-6.67793600	-0.27391400
H	-6.62447900	-5.75555000	-1.71240000
H	-5.22949600	-6.83793300	-1.76557400
H	-3.83679900	-2.87289600	3.01394500
H	-4.62505500	-1.51709700	2.21450400
H	-5.56920500	-2.94223300	2.67183400
H	-0.31830000	-0.70026500	-2.71775900
H	0.92235800	0.49805200	-3.01154600
H	0.61877400	-0.76823200	-4.22107400
H	3.85867500	-3.94772300	-4.89623700
H	5.31578200	-3.42675400	-4.03927500
H	4.49578700	-4.91124700	-3.55013600
H	4.31183700	-3.01311900	0.93230000
H	3.21763500	-1.71345400	1.39641900
H	2.61643400	-3.35887400	1.26921800
Si	-5.02158200	0.07518000	-1.76189600

Si	2.47795900	3.02313300	1.83348300
C	3.98206500	4.15753900	1.55759900
C	2.58628000	2.29990500	3.58478900
C	0.87908600	4.02489300	1.62302200
C	-5.20247700	1.86218500	-2.33026000
C	-5.57580500	-1.14596200	-3.07240900
C	-5.91009500	-0.17634900	-0.12710700
H	2.37834100	3.07772000	4.33083600
H	1.86854000	1.48934600	3.73918300
H	3.58686100	1.90269800	3.78495200
H	4.91679400	3.58797200	1.63004300
H	3.95317100	4.62528300	0.56645000
H	4.01620800	4.95711100	2.30870500
H	0.85804800	4.88748800	2.30169100
H	0.79050100	4.38884800	0.59315400
H	0.00601600	3.39909100	1.83187300
H	-4.54015700	2.07236600	-3.17798500
H	-6.23331600	2.07430400	-2.64032500
H	-4.94741600	2.55812600	-1.52328600
H	-6.64377300	-1.03921700	-3.29738100
H	-5.01701200	-1.00477600	-4.00485500
H	-5.39621200	-2.16936100	-2.72211600
H	-5.38802900	0.33907600	0.68760500
H	-6.92917900	0.22604000	-0.17936700
H	-5.97004100	-1.23816400	0.12831400
C	-2.49838700	-0.14544000	-0.54194700
O	-3.36752200	-0.25661300	-1.58638700
C	0.01538900	-3.38752800	1.47133300
O	0.13258300	-4.46250900	1.05823600
Mo	-0.24550600	-1.47843100	2.19698500

Mo-ts4

N	-0.79735900	2.75514200	0.83442900
O	1.45716300	1.38670600	0.10958800
Si	-0.49425400	1.38496900	-0.43128300

C	-0.81603800	3.64931300	-0.14461100
N	3.98580700	0.21158300	-0.42825800
Si	2.55051100	0.23069600	0.64682800
N	-0.52033800	2.98743100	-1.28289400
C	0.64850100	0.16846600	-1.01882000
C	4.79296300	1.28521500	-0.08496200
N	4.26716000	2.11013500	0.76078600
P	-2.55635000	-0.85865500	0.01475400
P	1.01604400	-1.35699500	-0.11083200
C	0.23394000	-3.61062200	2.32207300
C	-2.56205100	-3.17746400	2.16662400
C	-0.79774900	-0.93419300	2.62917000
O	-3.49751700	-3.61395500	2.70476800
O	0.91064600	-4.31687600	2.95496500
O	-0.73557700	-0.07674100	3.41284500
C	1.77133900	-2.57778400	-1.28408900
C	1.19786900	-2.80674000	-2.55767000
C	2.84221900	-3.39214500	-0.84895300
C	1.74530200	-3.79828400	-3.38013700
C	3.34944700	-4.37680100	-1.70309800
C	2.82497200	-4.58861400	-2.98028700
H	1.30102900	-3.96743600	-4.35935800
H	4.17535900	-4.99628600	-1.35824900
C	-4.36608600	-1.14495000	0.04798600
C	-4.94315300	-2.02446400	-0.89469500
C	-5.16894000	-0.54592600	1.04541500
C	-6.32231100	-2.25614200	-0.84792000
C	-6.54055800	-0.81412700	1.05736200
C	-7.13894900	-1.65565500	0.11471700
H	-6.76838600	-2.92665900	-1.57963500
H	-7.15964000	-0.35368000	1.82475300
C	-0.00594500	-2.04110600	-3.06290300
C	3.47215200	-3.22543200	0.51631800
C	-4.11046200	-2.73746100	-1.93832100

C	-4.58334600	0.37351300	2.09305100
H	-4.74835700	-3.24582100	-2.66791000
H	-3.45478400	-2.05211700	-2.48725500
H	-3.46303000	-3.49406200	-1.48113100
H	-3.74200000	-0.09512600	2.61693600
H	-4.21206000	1.29559500	1.63203700
H	-5.33539200	0.64311200	2.84102100
H	-0.82524200	-2.05736500	-2.33490600
H	0.23269200	-0.98746800	-3.23782500
H	-0.37920300	-2.47905800	-3.99444300
H	4.20592400	-4.01475100	0.70694600
H	3.99360500	-2.26370200	0.60638600
H	2.72632800	-3.27108300	1.31479100
Si	-4.17573700	2.02461900	-2.03469000
Si	3.18884800	0.07762500	2.91935900
C	-2.30183000	0.76344400	-0.41665200
O	-3.30869500	1.66881600	-0.61216800
C	-1.06224000	-3.88648000	-0.20545500
O	-1.11324000	-4.72149500	-1.00479900
C	2.53435700	-1.45266100	3.82703900
C	5.08255900	-0.04998100	3.05291400
C	2.50272600	1.61302800	3.79935900
H	1.44469800	-1.50506500	3.84526300
H	2.90732900	-2.38580600	3.39499300
H	2.88348200	-1.40807800	4.86769000
H	5.35839100	-0.24548500	4.09717100
H	5.44942000	-0.89258600	2.45389800
H	5.60786300	0.84844400	2.72216900
H	1.42465800	1.48882000	3.95386400
H	2.96841700	1.74550200	4.78404700
H	2.66111200	2.52387100	3.21378600
C	4.08630000	-0.39515900	-1.76547700
C	5.01321000	3.16204400	1.42148300
C	-0.18098800	3.52319500	-2.58298400

C	-1.20299400	2.91084400	2.21299900
H	-0.70699800	4.46492600	-2.76779100
H	0.89838000	3.69859900	-2.67088700
H	-0.47142100	2.80337900	-3.35173200
H	-2.20430200	2.49362100	2.37531500
H	-0.50299200	2.36982500	2.85188200
H	-1.20707600	3.96655000	2.49793500
H	3.10605100	-0.39051700	-2.24714600
H	4.77763300	0.18190900	-2.38384900
H	4.44494300	-1.42697700	-1.71685400
H	4.79578500	3.13325300	2.49560500
H	6.10206800	3.10119300	1.29128100
H	4.67955400	4.14187500	1.05478100
C	-1.15154400	5.08228400	-0.03482600
C	-0.26389400	6.05834600	-0.50927000
C	-2.37570700	5.46418000	0.53454700
C	-0.59881100	7.40868700	-0.40731100
H	0.68825800	5.76062700	-0.93755800
C	-2.70937200	6.81487100	0.62250900
H	-3.06603300	4.70161400	0.88102500
C	-1.82084800	7.78726000	0.15477600
H	0.09385100	8.16387000	-0.76681900
H	-3.66148400	7.10775000	1.05496300
H	-2.08096400	8.83931400	0.22808000
C	6.17713500	1.34547100	-0.64034600
C	6.65878600	2.50967600	-1.25405600
C	7.01583800	0.22548400	-0.53421400
C	7.96170700	2.55462600	-1.75270100
H	6.00564100	3.37241200	-1.34764000
C	8.32100800	0.27582400	-1.02127600
H	6.63629300	-0.67753900	-0.06497000
C	8.79541100	1.43996900	-1.63366100
H	8.32478900	3.45840800	-2.23408200
H	8.96691400	-0.59269700	-0.92710100

H	9.81072500	1.47670000	-2.01877700
C	-5.96624000	1.52147800	-1.78344600
C	-4.05793700	3.88819300	-2.25852800
C	-3.37613300	1.09555500	-3.46055400
C	-8.63108000	-1.88840400	0.12293100
C	3.41256600	-5.63086900	-3.90240200
H	-9.15199000	-1.10568300	-0.44494600
H	-8.89067000	-2.85003800	-0.33206700
H	-9.03322400	-1.87257700	1.14171600
H	2.65341200	-6.04455000	-4.57524800
H	4.20503800	-5.20184600	-4.53083900
H	3.85670400	-6.45943700	-3.34000800
H	-3.81998300	1.39780000	-4.41676100
H	-2.29849100	1.28812300	-3.51039300
H	-3.51311400	0.01466400	-3.35322000
H	-6.58475600	1.87406400	-2.61883800
H	-6.07188500	0.43507200	-1.70772500
H	-6.36642800	1.95730800	-0.86090200
H	-4.45698200	4.41552900	-1.38455400
H	-3.02182100	4.21322800	-2.39317600
H	-4.63413300	4.21071400	-3.13460200
Mo	-0.95190100	-2.42044900	1.24117000

Mo-int7

127

N	0.46355900	3.43217600	0.38924300
O	0.53720100	0.62239200	1.09577800
Si	-0.30611800	0.84506400	-0.35034300
C	1.02353200	3.15068600	-0.72859400
N	3.27636100	-0.17524100	0.47009900
Si	1.70842200	-0.55138900	1.25684100
N	0.59169700	1.98628600	-1.36477700
C	-0.35186700	-0.76116400	-1.24980100
C	3.96080100	0.79550100	1.19803500
N	3.28567400	1.32303800	2.16022200

P	-3.08721700	-0.04235100	-0.08968400
P	0.64967100	-1.84359500	-0.41822400
C	-1.25368200	-4.20291700	0.13035400
C	-3.76450700	-3.04351800	0.39001900
C	-1.69617200	-1.85702500	1.93504700
O	-4.81919200	-3.45920600	0.63318500
O	-0.89660200	-5.30257600	0.22613000
O	-1.66280400	-1.55009300	3.05278300
C	1.53093800	-3.16805800	-1.33991900
C	1.64416100	-3.16546600	-2.75589600
C	2.13454400	-4.21005400	-0.59367500
C	2.31137100	-4.23073500	-3.37217400
C	2.78925200	-5.24786400	-1.26205500
C	2.88159400	-5.28417000	-2.65380900
H	2.38985200	-4.23249100	-4.45736100
H	3.23548300	-6.04887500	-0.67648400
C	-4.82436500	0.36859400	0.29114800
C	-5.81884600	0.04195200	-0.66236400
C	-5.18375400	0.98226200	1.51510000
C	-7.14781800	0.38303300	-0.39736400
C	-6.52889300	1.30408600	1.73089500
C	-7.52174100	1.02623200	0.78688300
H	-7.91084900	0.13980800	-1.13375300
H	-6.80913000	1.77729200	2.66971000
C	1.11652800	-2.06668400	-3.65115900
C	2.13632100	-4.22149800	0.91613200
C	-5.47996000	-0.66603900	-1.95681900
C	-4.17263500	1.28984800	2.59620900
H	-6.36246800	-0.74788200	-2.59848100
H	-4.70215600	-0.13884400	-2.52097400
H	-5.11008700	-1.68111900	-1.77098100
H	-3.55328900	0.41783600	2.83346700
H	-3.49551100	2.09188200	2.28507800
H	-4.67500800	1.60524000	3.51580600

H	0.02765400	-2.00258700	-3.62993100
H	1.48149300	-1.08420100	-3.33671000
H	1.43993600	-2.23939400	-4.68282800
H	2.43833200	-5.20042500	1.29997400
H	2.84450200	-3.48223400	1.31309400
H	1.15646000	-3.99155700	1.33729900
Si	-3.26466200	3.75728700	-0.79996500
Si	1.93508700	-1.27590900	3.49531900
C	-2.10779100	1.34049400	-0.03337800
O	-2.54054400	2.59032500	0.20199000
C	-2.25880900	-2.53380000	-2.08183400
O	-2.45672800	-2.61690200	-3.21493100
C	1.19820800	-2.97619200	3.91855600
C	3.78652200	-1.42315100	3.89771300
C	1.05525600	0.03653300	4.53631400
H	0.22518900	-3.16681700	3.46137700
H	1.87761400	-3.78801900	3.64089400
H	1.06209400	-3.01755000	5.00746600
H	3.90888000	-1.85283400	4.90018900
H	4.28201500	-2.09782500	3.18828300
H	4.30843800	-0.46409800	3.86860900
H	-0.02691800	-0.05031300	4.39300300
H	1.27249600	-0.10150500	5.60321800
H	1.35274000	1.04659700	4.24156100
C	3.62097400	-0.47962700	-0.92395100
C	3.86214500	2.20291200	3.15539500
C	0.71641700	1.84152900	-2.81857800
C	0.95856900	4.45385600	1.28208300
H	0.30425900	2.71212000	-3.34445800
H	1.75996600	1.72026000	-3.13307200
H	0.16141600	0.95189400	-3.12048200
H	0.17245900	5.20005500	1.46106800
H	1.18063200	3.98863100	2.25156800
H	1.85983200	4.98215300	0.93823400

H	2.88363300	-0.05236800	-1.61477000
H	4.59769900	-0.06113200	-1.17224700
H	3.66413200	-1.55854600	-1.09066400
H	3.54819700	1.86647000	4.15061700
H	4.95883600	2.25866100	3.14107300
H	3.46488600	3.21738300	3.02172600
C	2.13104200	3.91880000	-1.38195500
C	3.37524700	3.31112100	-1.59707000
C	1.94676200	5.25432600	-1.76301000
C	4.42358100	4.02821300	-2.17342900
H	3.51856100	2.27883800	-1.29619400
C	2.99129900	5.96905600	-2.35296700
H	0.98199900	5.72563800	-1.59873700
C	4.23166200	5.35872800	-2.55709600
H	5.38865200	3.54940100	-2.31471300
H	2.83732600	7.00279200	-2.65037500
H	5.04524600	5.91740900	-3.01171300
C	5.39391700	1.06888200	0.87559700
C	5.83215500	2.37599700	0.62416000
C	6.32129800	0.01590000	0.85504200
C	7.17643000	2.62543600	0.34261700
H	5.11490300	3.18995300	0.63182000
C	7.66622300	0.26851600	0.58687600
H	5.97928100	-0.99634900	1.05152200
C	8.09537000	1.57342700	0.32527400
H	7.50367300	3.64111800	0.13848600
H	8.37868700	-0.55164000	0.57894000
H	9.14221100	1.76838400	0.10951500
C	-4.49669400	4.61631200	0.32450300
C	-1.93574800	4.93573400	-1.41616400
C	-4.09750900	2.89669400	-2.24843000
C	-8.95795900	1.42299100	1.03202800
C	3.60997900	-6.40218300	-3.36019900
H	-9.16159300	2.42329500	0.62676100

H	-9.65402600	0.72877800	0.54916900
H	-9.18947500	1.45170100	2.10200200
H	3.16559600	-6.61382700	-4.33862100
H	4.66255900	-6.13880900	-3.53094900
H	3.59561700	-7.32494100	-2.77075900
H	-4.41833700	3.63512500	-2.99354100
H	-3.40907700	2.20039900	-2.74183100
H	-4.97694900	2.33181300	-1.92429400
H	-5.01502800	5.43009800	-0.19697600
H	-5.24719000	3.89958900	0.67779300
H	-3.99477900	5.04208100	1.20117800
H	-1.36112900	5.34453300	-0.57910700
H	-1.23331600	4.41540100	-2.07635900
H	-2.38215100	5.76724400	-1.97601600
Mo	-1.94027900	-2.31751500	-0.04150400

3'

90

P	2.50924600	1.56867900	1.10392700
C	4.19394100	0.81603500	0.93376100
C	4.76654400	0.12272700	2.02400500
C	6.05066400	-0.41760600	1.89275300
H	6.48097700	-0.96404200	2.73031600
C	6.79424800	-0.27373800	0.71831900
C	6.22998500	0.45434600	-0.33306000
H	6.80011600	0.58949800	-1.25027500
C	4.94772500	1.00424600	-0.24697200
C	4.02455700	-0.04836900	3.33360600
C	8.16083100	-0.90271200	0.57867800
C	4.40305000	1.79608600	-1.41728400
P	-0.94036400	-1.55889400	0.11657800
C	-2.26789000	-2.80409300	0.30568900
C	-2.78637300	-3.45600500	-0.83859400
C	-3.83093700	-4.37136800	-0.67291400
H	-4.24354000	-4.85979700	-1.55319500

C	-4.35742700	-4.67740900	0.58544400
C	-3.81408900	-4.03658600	1.70303600
H	-4.20898300	-4.26619600	2.69051100
C	-2.77812100	-3.10362000	1.59101200
C	-2.25317700	-3.20255400	-2.23287000
C	-5.46039600	-5.69738400	0.73695400
C	-2.24825900	-2.43670700	2.84076100
Si	-0.24700300	1.25909400	0.31861000
N	-0.79236600	2.48952800	-0.93744600
C	-1.13793100	2.54912700	-2.34635300
C	-1.03002600	3.39682600	0.02506700
C	-1.52856100	4.76662800	-0.16350400
C	-2.51616900	5.28046000	0.69308600
H	-2.92167000	4.65754900	1.48314600
C	-2.99358200	6.57569600	0.50405100
H	-3.76543800	6.96644500	1.16005900
C	-2.47962400	7.36763600	-0.52683700
H	-2.84968800	8.37886200	-0.66861200
C	-1.49050400	6.86243800	-1.37561100
H	-1.08644800	7.48013000	-2.17182000
C	-1.01864700	5.56281500	-1.20192500
H	-0.24172300	5.17168000	-1.85069500
N	-0.72203500	2.80504800	1.18970700
C	-0.58911600	3.39835200	2.50746000
C	-1.56049300	-0.04018800	0.55084200
O	-2.78059600	0.27192200	1.06110200
Si	-4.29950700	0.45482900	0.31101300
C	-5.50750800	-0.53651500	1.34701900
H	-6.53453600	-0.41015700	0.98289300
H	-5.48081800	-0.21654500	2.39533800
H	-5.25784800	-1.60265900	1.31169500
C	-4.21567100	-0.15298300	-1.46334000
H	-4.21607000	-1.24589800	-1.50438600
H	-3.31186400	0.20452200	-1.96936000

H	-5.08240500	0.21088900	-2.02837600
C	-4.68954800	2.29546400	0.37663200
H	-5.70029100	2.50100100	0.00324800
H	-3.98502000	2.87453400	-0.23129300
H	-4.63032600	2.66872300	1.40601000
O	1.15549800	-4.43010200	-2.02117700
O	0.04398300	-0.30105400	-3.52435300
O	4.20734400	-1.32082600	-2.27091100
O	2.71519000	-2.68792000	1.81580300
C	1.24090700	-3.34483500	-1.59770900
C	0.54128100	-0.71357500	-2.55658800
C	3.18638100	-1.33939900	-1.71600800
C	2.23108800	-2.24578700	0.86347300
C	1.43491700	0.60222300	0.23050800
H	8.09135600	-1.88997700	0.10254500
H	8.82350500	-0.29023800	-0.04320100
H	8.64102700	-1.04361400	1.55326600
H	4.65226700	-0.55422500	4.07457000
H	3.72226400	0.91888000	3.75518800
H	3.11592300	-0.64559400	3.20770900
H	3.45755900	1.37975800	-1.77680500
H	4.20969200	2.84020300	-1.13692500
H	5.11150200	1.79716300	-2.25157900
H	-1.29316700	-3.70716000	-2.38171400
H	-2.94993600	-3.58312400	-2.98627400
H	-2.09071100	-2.13928400	-2.43450900
H	-5.04411000	-6.70503400	0.86764600
H	-6.08475000	-5.48675100	1.61177700
H	-6.10590800	-5.72460200	-0.14739100
H	-1.15710800	-2.51751100	2.91274600
H	-2.50076000	-1.37060100	2.85021300
H	-2.67706600	-2.89516100	3.73699700
H	-1.46050300	1.56062700	-2.67805700
H	-1.94550100	3.26651800	-2.51786600

H	-0.27094600	2.83608100	-2.95136600
H	0.32257500	3.01736300	2.97499900
H	-0.51667100	4.48694700	2.43598300
H	-1.44659500	3.13861500	3.13864600
Mo	1.37396600	-1.49013600	-0.86045700

References

1. J. Bresien, A. Schulz, A. Villinger. *Dalton Trans.*, **2016**, *45*(2), 498-501.
2. J. Bresien, K. Faust, C. Hering-Junghans, J. Rothe, A. Schulz, A. Villinger. *Dalton Trans.*, **2016**, *45*(5), 1998-2007.
3. Bruker AXS Inc., SAINT *v8.37* A, Madison, **2016**.
4. L. Krause, R. Herbst-Irmer, G. M. Sheldrick, D. Stalke, *J. Appl. Crystallogr.* **2015**, *48*, 3–10.
5. G. M. Sheldrick, *Acta Crystallogr.,* **2015**, *A71*, 3–8.
6. G. M. Sheldrick, *Acta Crystallogr. Sect.*, **2015**, *C71*, 3–8.
7. C. B. Hübschle, G. M. Sheldrick, B. Dittrich, *J. Appl. Cryst.,* **2011**, *44*, 1281–1284.
8. Stoe & Cie. X-RED32 and X-AREA. Stoe & Cie GmbH, Darmstadt, Germany, **2018**.
9. S. Ishida, T. Abe, F. Hirakawa, T. Kosai, K. Sato, M. Kira, T. Iwamoto, *Chem. Eur. J.* **2015**, *21*, 15100–15103.
10. M. M. Linden, H. P. Reisenauer, D. Gerbig, , M. Karni, A. Schäfer, T. Müller, Y. Apeloig, P. R Schreiner, *Angew. Chem., Int. Ed.* **2015**, *54*, 12404–12409.
11. D. Wendel, D. Reiter, A. Porzelt, P. J. Altmann, S. Inoue, B. Rieger. *J. Am. Chem. Soc.* **2017**, *139*, 17193-17198.
12. A. D. Becke, Density-functional thermochemistry. III. The role of exact exchange. *J. Chem. Phys.* **1993**, *98*, 5648–5652.
13. C. Lee,; W. Yang,; R. G. Parr, Development of the Colic-Salvetti correlation-energy formula into a functional of the electron density. *Phys. Rev. B* **1988**, *37*, 785–789.
14. W. J. Hehre, R. Ditchfield, J. A Pople, Self-Consistent Molecular Orbital Methods. XII. Further Extensions of Gaussian Type Basis Sets for Use in Molecular Orbital Studies of Organic Molecules. *J. Chem. Phys.* **2003**, *56*, 2257-2261.
15. S. Grimme, S. Ehrlich, L Goerigk, Effect of the damping function in dispersion corrected density functional theory. *J. Comput. Chem,* **2011**, *32*, 1456–1465.
16. S. Grimme, J. Antony, S. Ehrlich, H Krieg, A consistent and accurate ab initio parametrization of density functional dispersion correction (DFT-D) for the 94 elements H-Pu. *J. Chem. Phys.* **2010**, *132*, 154104.
17. M. Swart, M. Güell, J. M. Luis, M Solà, Spin-state corrected Gaussian-type orbital basis sets, *J. Phys. Chem. A*, **2010**, *114*, 7191–7197.
18. F. Weigend, R Ahlrichs, Balanced basis sets of split valence, triple zeta valence and quadruple zeta valence quality for H to Rn: Design and assessment of accuracy. *Phys. Chem. Chem. Phys.* **2005**, *7*, 3297–3305.
19. Y. Wang, A. Kostenko, T. J. Hadlington, M-P. Luecke, S. Yao, M. Driess, Silicon-Mediated Selective Homo- and Heterocoupling of Carbon Monoxide. *J. Am. Chem. Soc.* **2019**, *141*, 626–634
20. J. Tomasi, B. Mennucci, R. Cammi, Quantum mechanical continuum solvation models. *Chem. Rev.* **2005**, *105*, 2999–3094.
21. M. J. Frisch, *Gaussian 16, Revision A.03*; Gaussian, Inc.: Wallingford CT, **2016**.
